# Development of the Squaramide Scaffold for High Potential and Multielectron Catholytes for Use in Redox Flow Batteries

**DOI:** 10.1021/jacs.3c14776

**Published:** 2024-04-17

**Authors:** Jacob S. Tracy, Conor H. Broderick, F. Dean Toste

**Affiliations:** † Chemical Sciences Division, Lawrence Berkeley National Laboratory, Berkeley, California 94720, United States; ‡ Department of Chemistry, University of California, Berkeley, California 94720, United States; § Joint Center for Energy Storage Research (JCESR), Argonne, Illinois 60429, United States; ∥ Department of Chemistry, University of West Florida, Pensacola, Florida 32514, United States

## Abstract

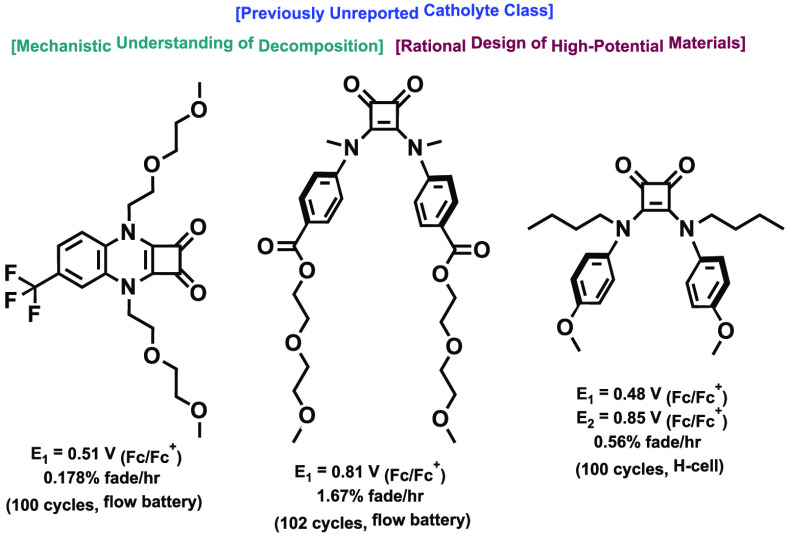

Nonaqueous organic redox flow batteries (N-ORFBs) are a promising technology for grid-scale storage of energy generated from intermittent renewable sources. Their primary benefit over traditional aqueous RFBs is the wide electrochemical stability window of organic solvents, but the design of catholyte materials, which can exploit the upper range of this window, has proven challenging. We report herein a new class of N-ORFB catholytes in the form of squaric acid quinoxaline (SQX) and squaric acid amide (SQA) materials. Mechanistic investigation of decomposition in battery-relevant conditions via NMR, HRMS, and electrochemical methods enabled a rational design approach to optimizing these scaffolds. Three lead compounds were developed: a highly stable one-electron SQX material with an oxidation potential of 0.51 V vs Fc/Fc^+^ that maintained 99% of peak capacity after 102 cycles (51 h) when incorporated into a 1.58 V flow battery; a high-potential one-electron SQA material with an oxidation potential of 0.81 V vs Fc/Fc^+^ that demonstrated negligible loss of redox active material as measured by pre- and postcycling CV peak currents when incorporated in a 1.63 V flow battery for 110 cycles over 29 h; and a proof-of-concept two-electron SQA catholyte material with oxidation potentials of 0.48 and 0.85 V vs Fc/Fc^+^ that demonstrated a capacity fade of just 0.56% per hour during static H-cell cycling. These findings expand the previously reported space of high-potential catholyte materials and showcase the power of mechanistically informed synthetic design for N-ORFB materials development.

## Introduction

**Figure 1:**
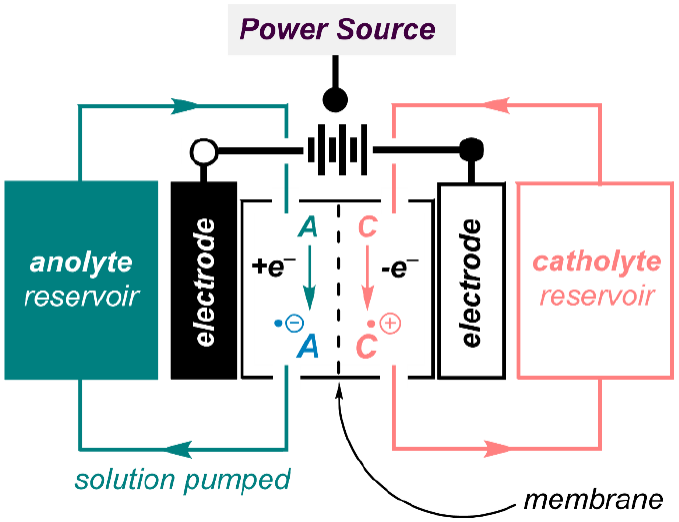
Generalized schematic of a redox flow battery during charging.

The intensifying effects of climate change necessitate rapid decarbonization of the electrical grid.^1,2^ This presents multiple challenges, one of which is the intermittency of renewable energy resources such as wind and solar power.^3^ A promising option for addressing the mismatch between supply and demand for renewable energy is the widespread incorporation of grid-scale electrochemical energy storage infrastructure.^4,5^ Redox-flow batteries (RFBs) are an early stage technology well suited for this purpose due to a unique architecture that decouples the power (J/s) from the storage capacity (*J*) of the battery.^6,7^ As shown in the schematic in Figure 1, redox-flow batteries store electroactive material (redoxmers) as solutes dissolved in tanks of solvent. These redoxmer solutions are then flowed through an electrochemical stack, where all electrochemical transformations take place. Within this process, the redoxmer that undergoes oxidation upon charging is referred to as the catholyte, while that is reduced upon charging is referred to as the anolyte. By storage of storing all active material in tanks that are decoupled from the location of the electrochemical processes, capacity can be increased simply by increasing the volume of active material solution. Power – how quickly the energy stored in the battery can be discharged – is in turn determined by the performance of the electrochemical stack. This is in comparison to lithium-ion batteries, the current incumbent technology for grid-scale electrochemical energy storage, which cannot readily decouple power from capacity due to their use of solid intercalation electrodes to store electroactive materials.^6,8^

When optimizing redox-flow batteries, two key terms must be considered: volumetric energy density and electrochemical stability. Commercial systems in particular are hindered by the limited thermodynamic electrochemical window (<1.5 V) of water, limiting the maximum achievable volumetric energy density.^9^ Nonaqueous organic RFBs (N-ORFBs), in contrast, can take advantage of the much wider electrochemical windows of organic solvents (>4 V), and the increased storage capacity benefits that they may enable.^10^ While the development of N-ORFB active materials has seen substantial progress in recent years, the synthesis of high-potential flow battery catholyte materials, which can take advantage of these expanded electrochemical windows while still exhibiting low capacity fade, high solubility, and ideally multielectron oxidations is an outstanding challenge.^11^

**Figure 2:**
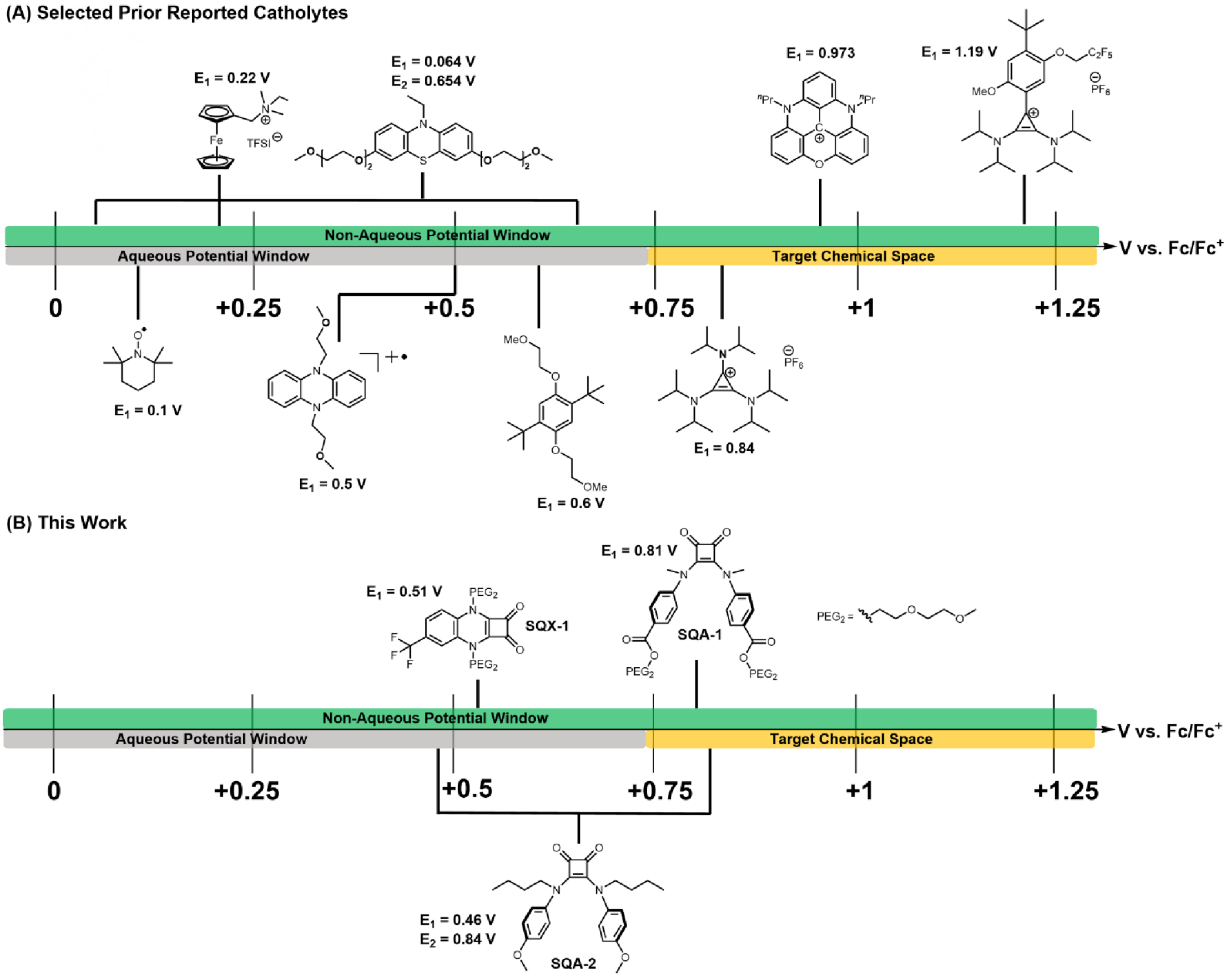
(a) Selected catholyte materials. (b) Structures and potentials of the top-performing squaramide materials described in this work.

The majority of previously reported catholyte scaffolds, including TEMPO derivatives, dialkoxybenzenes, ferrocenes, phenothiazines, and phenazines, as shown in Figure 2a, exhibit oxidation potentials below 0.6 V vs Fc/Fc^+^.^12−15^ N-ORFB catholyte materials, which exhibit potentials above 0.8 V vs Fc/Fc^+^, however, are primarily limited to the cyclopropenium scaffolds developed by the Sanford group, [4]helicinium scaffolds pioneered by the Gianetti group, and tetrathiafulvalene derivatives, which have seen use in hybrid Li/organic RFBs.^11,16−18^ We therefore aimed at the outset of our research to expand the chemical space of these high-oxidation potential catholyte scaffolds.

Beginning our search for candidate scaffolds, we discovered a 1977 report by Hünig and coworkers describing the cyclic voltammograms of squaric acid amides (SQAs) and squaric acid quinoxalines (SQXs), which displayed reversible one- and two-electron oxidations at voltages on the order of 0.3–0.5 V vs Fc/Fc^+^, respectively.^19^ At the time we initiated this work, these scaffolds had not been explored as active materials for electrochemical energy storage, and structure–property understanding was nonexistent. Very recently and near the conclusion of our efforts, an elegant 2023 report by the Hansmann group showed that SQXs were functional as the redox-active species in a polymeric cathode for Li/organic batteries.^20^ While these previously reported SQX derivatives indicate the promise of squaramide-derived materials for charge storage applications, their oxidation potentials remain substantially lower than reported best-in-class catholyte materials, the higher-oxidation potential SQA molecules investigated by the Hansmann group underwent rapid decomposition of the radical cation via an undefined mechanism, and multielectron cycling in conditions relevant to flow batteries has not been achieved.

In this work, we addressed each of these challenges through a strategy combining rigorous characterization of decomposition pathways with synthetic optimization to improve the stability and increase the oxidation potential of squaramide derivatives. Ultimately, this enabled us to develop classes of squaramide catholytes, which address each of the key parameters for volumetric energy density optimization–potential, solubility, and number of electrons – while achieving comparable oxidation potentials and stabilities to the initial reports on the best-in-class cyclopropenium catholytes developed by the Sanford group.^21^ We believe that squaramide-based catholyte materials hold substantial promise as high-oxidation potential catholytes and that by developing upon these scaffolds, elucidating their decomposition, and proving their utility in laboratory-scale flow batteries, this work will expand the horizons of future high-voltage N-ORFBs.

## Results and Discussion

**Figure 3:**
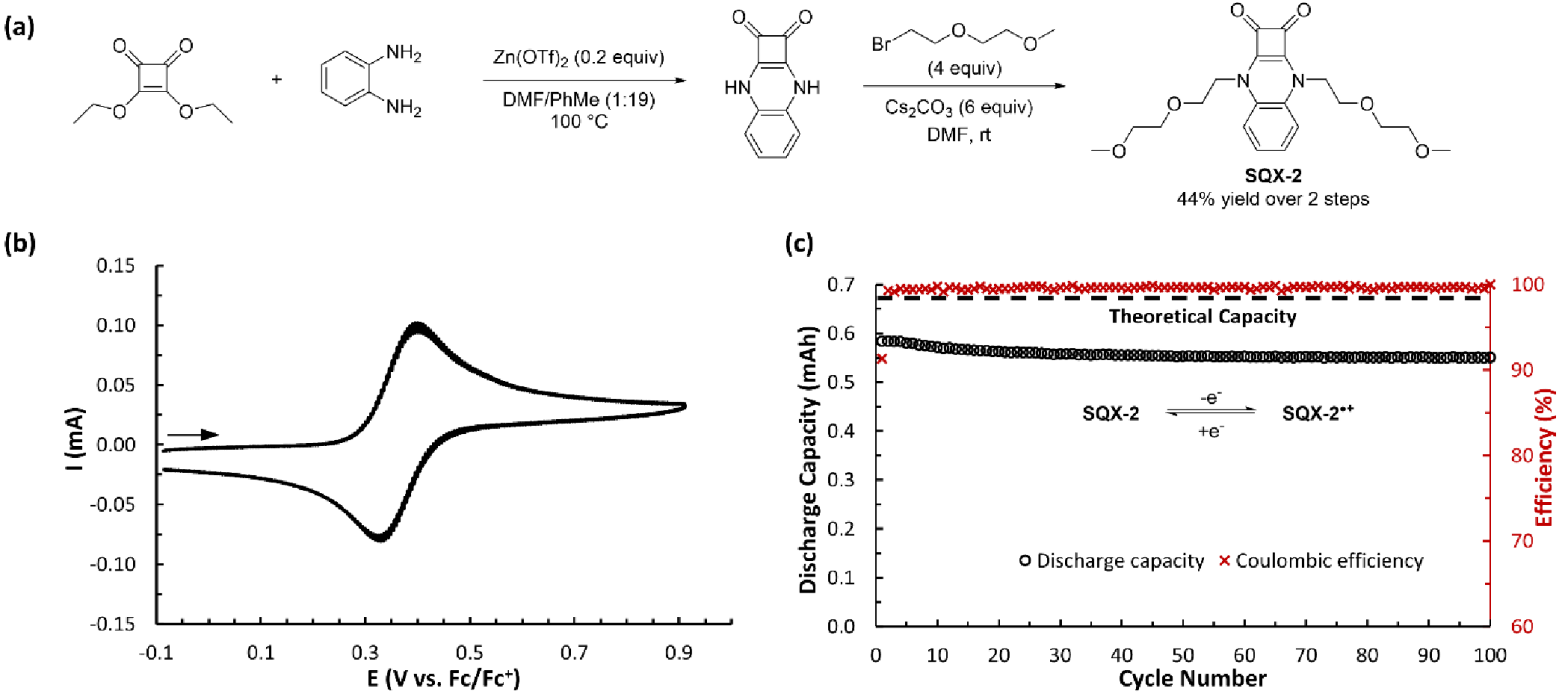
(a) Two-step synthesis of **SQX-2** from commercially available materials. (b) CV of **SQX-2** (5 mM in 0.5 M TBAPF_6_/MeCN) with a scan rate of 100 mV s^–1^. (c) Static oxidative H-cell cycling data between **SQX-2** and **SQX-2**^•**+**^, showing discharge capacity and Coulombic efficiency vs cycle number for 5 mM **SQX-2** in 0.5 M TBAPF_6_/MeCN.

To begin our studies, we performed a two-step synthesis of squaric acid quinoxaline **SQX-2** (Figure 3a), an analog of the original molecule reported by Hünig but with glycol ethers introduced for improved organic solubility.^19^ This molecule displayed reversible oxidation (0.35 V vs Fc/Fc^+^) across a range of scan rates in cyclic voltammetry studies using acetonitrile (MeCN) as the solvent and 0.5 M tetrabutylammonium hexafluorophosphate (TBAPF_6_) as the supporting electrolyte (Figure 3b). While this result was expected given the report by Hünig, the long-term stability of this class of radical cations was unknown at the time of our synthesis. To study this, we initiated galvanostatic charge–discharge cycling experiments (100 cycles, 5 mM active material in 0.5 M TBAPF_6_/MeCN,) in a 3-electrode static H-cell equipped with a glass-frit separator that allowed us to iteratively cycle both sides of the H-cell between **SQX-2** and **SQX-2**^•**+**^ (see Supporting Information for further details) using voltage cutoffs of typically ±350 mV from *E*_1/2_ to maximize the system’s state of charge (SOC).

**Figure 4:**
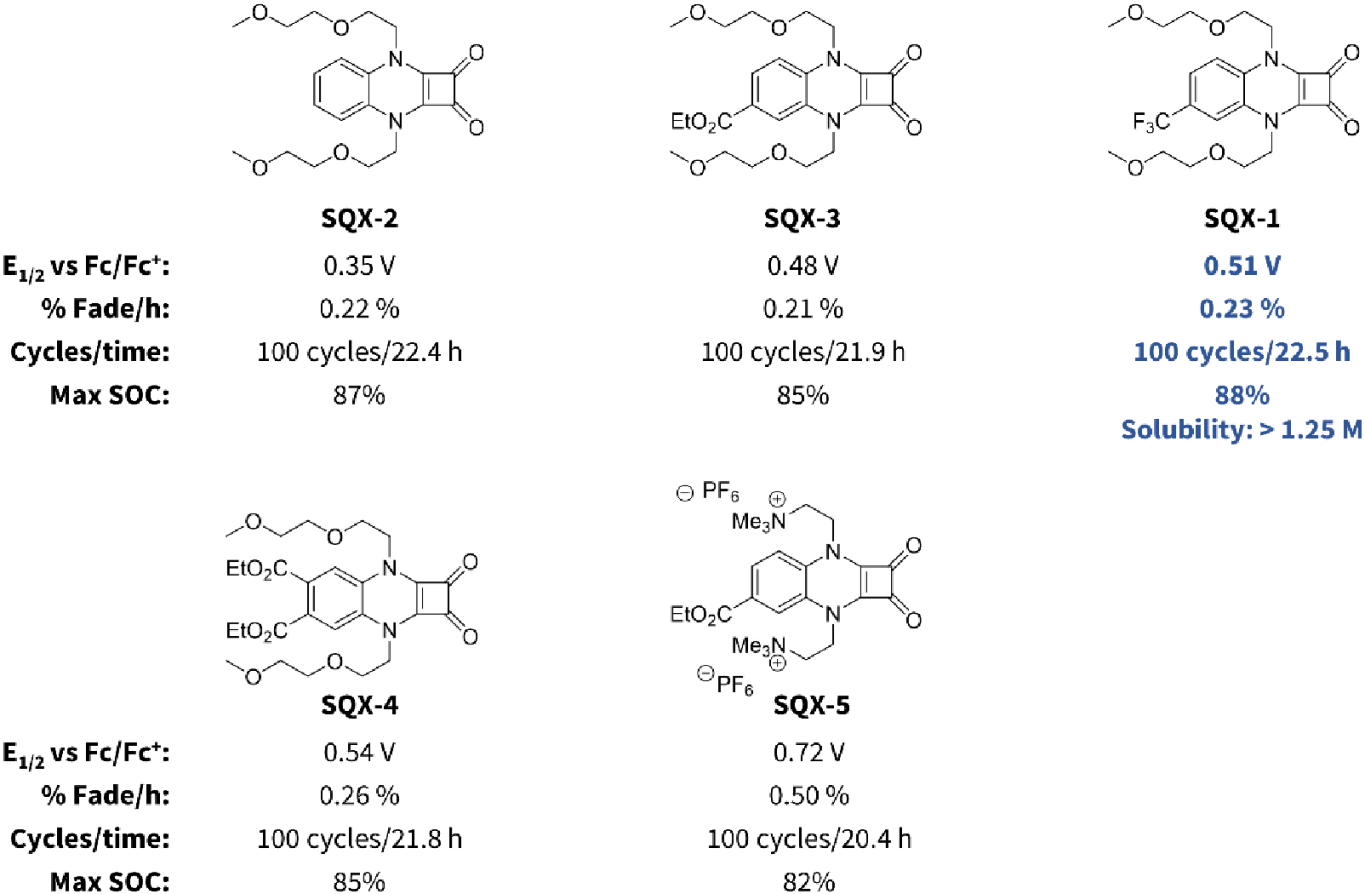
Oxidation potential and one-electron H-cell cycling stability of SQX derivatives **SQX-1** to **SQX-5**. Solubility for **SQX-1** was determined by a UV–vis calibration curve in an electrolyte solution of 500 mM TBAPF_6_ in MeCN, per Supporting Information page S25.

Over the course of 100 cycles (22.4 h), **SQX-2** reached a maximum SOC of 85% as measured by discharge capacity and showed excellent long-term electrochemical stability with a capacity fade normalized to a theoretical maximum of just 0.22% per hour (Figure 3c). Having established this class of ROM to have high electrochemical stability, we sought to improve oxidation potential by introducing electron-withdrawing groups at positions 4 and 5 of the arene (Figure 4). The introduction of a single ethyl ester to the 4-position (**SQX-3**) increased the oxidation potential by 130 mV to 0.48 V vs Fc/Fc^+^ without impacting electrochemical reversibility. Addition of a trifluoromethyl group to this same position (**SQX-1**) resulted in a further improvement in oxidation potential (0.51 V vs Fc/Fc^+^), again with no impact on the electrochemical stability of the corresponding radical cation. While a single ethyl ester (**SQX-3**) resulted in a 130 mV improvement in oxidation potential as compared to the parent **SQX-2**, adding ethyl esters to both the 4- and 5-positions (**SQX-4**) provided diminishing returns and resulted in an improvement of just 190 mV to 0.54 V vs Fc/Fc^+^. While this was 30 mV higher than what could be achieved by a single CF_3_ group, **SQX-1** was considered our lead **SQX** molecule at this point, as accessing the requisite diaminobenzene with the two esters required a low-yielding four-step synthesis, whereas the analogous 4-trifluoromethyl diaminobenzene is commercially available.

Our final attempt to improve the oxidation potential was to combine an ester at the 4-position with tetraalkyl ammonium solubilizing groups (**SQX-5**) in place of the glycol ethers. As expected, introducing two positive charges resulted in a major increase in oxidation potential to 0.72 V vs Fc/Fc^+^. However, formation of a tricationic radical during charging doubled the observed capacity fade to 0.50% per hour. While still showing reasonably high stability, a 2-fold decrease in long-term stability can have major implication in long-term battery performance, and we therefore decided to end our pursuit of this cationic strategy for improving oxidation potential.

**Figure 5:**
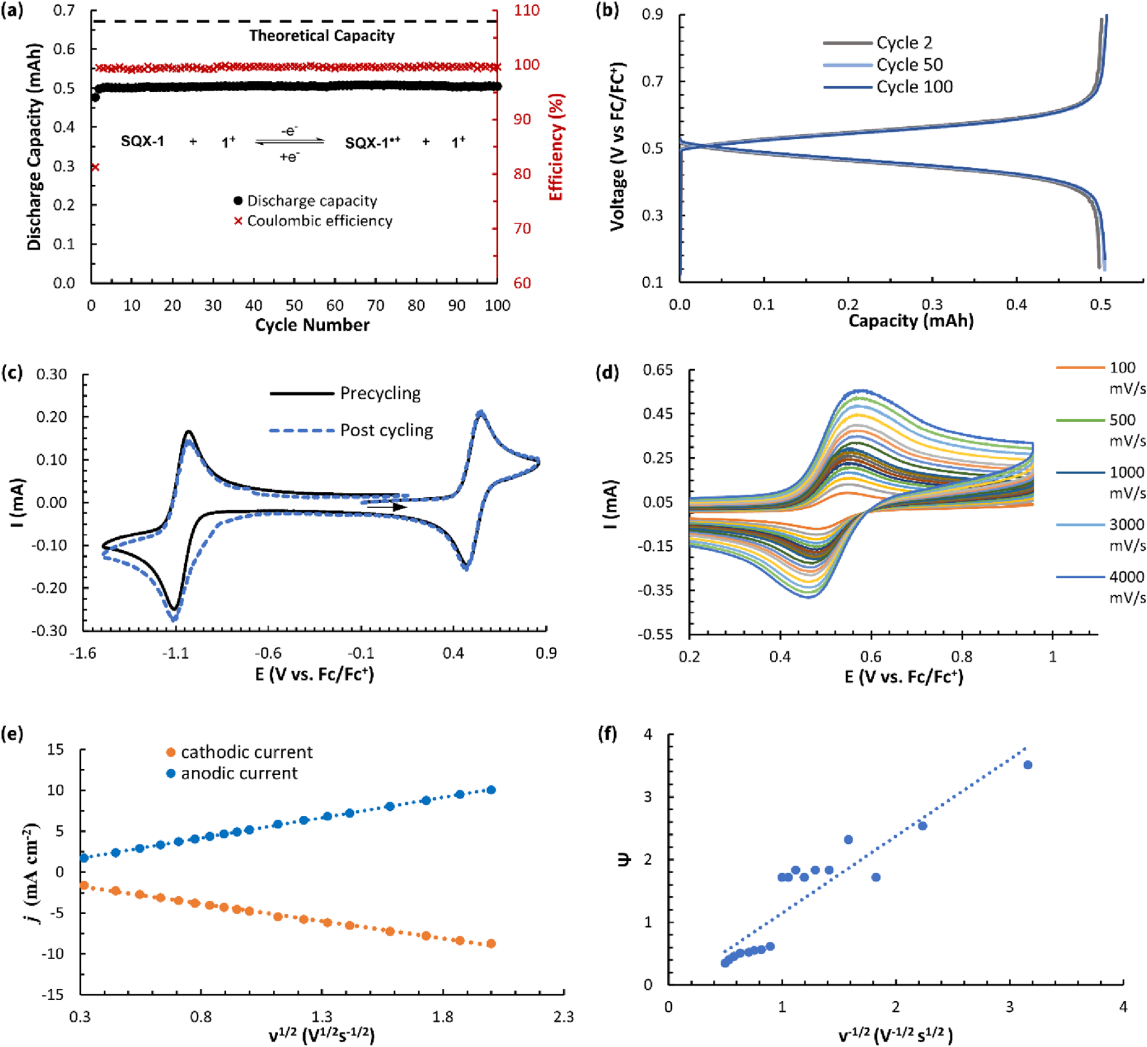
(a) Static oxidative H-cell cycling data between **SQX-1** and **SQX-1**^•**+**^ showing discharge capacity and Coulombic efficiency vs cycle number (100 cycles, 20.2 h) for the mixed solution of 5 mM **SQX-1** and 5 mM **1**^**+**^ in 0.5 M TBAPF_6_/MeCN. (b) Nernst curves showing potential versus capacity for the 2nd, 50th, and 100th cycles from the data shown in part (a). (c) CVs (500 mV s^–1^, glassy carbon electron) of the working side solution before and after the H-cell cycling of **SQX-1** and **SQX-1**^•**+**^ in a mixture of 5 mM **SQX-1** and 5 mM **1**^**+**^. (d) Variable scan CV (5 mM in 0.5 M TBAPF_6_/MeCN) of compound **SQX-1** alone from 100 to 4000 mV s^–1^. (e) Plots of anodic and cathodic peak current densities (*j*) vs the square root of the sweep rate (ν^1/2^) for **SQX-1**. (f) Nicholson’s dimensionless parameter Ψ vs inverse square root of the sweep rate (ν^–1/2^) for **SQX-1**.

Having identified **SQX-1** as having the best combination of oxidation potential, cycling stability, and synthetic accessibility, we used rate-dependent cyclic voltammetry to learn about its mass transport and electrokinetic properties (Figure 5d–f). Oxidation of **SQX-1** was found to be a transport-limited redox process with a diffusion coefficient (*D*) of 1.17 × 10^–5^ cm^2^ s^–1^ as determined from the Randles–Ševčík equation and with a heterogeneous electron-transfer rate (*k*^0^) of 7.4 × 10^–2^ cm s^–1^ as determined by the Nicholson method.^22,23^ Finally, the solubility of **SQX-1** was measured to be 2.23 ± 0.06 M in MeCN without supporting electrolyte and 1.25 M in supporting electrolyte (see Supporting Information for details).

The final test for **SQX-1** was to ascertain its performance under flow battery relevant conditions. To do so, we envisioned the use of a 50:50 mixed catholyte/anolyte flow battery design that minimizes the impacts of crossover by eliminating concentration gradients across the battery while it is in the fully discharged state. However, this battery design requires the catholyte and anolyte to be stable in the presence of each other across all charge states. For this purpose, we explored pyridinium **1**^**+**^, an anolyte first reported by the Sanford group,^24^ which has a reduction potential of −1.07 V vs Fc/Fc^+^. To determine its compatibility with squaramide **SQX-1**, static H-cell cycling experiments were conducted whereby 5 mM **SQX-1** and 5 mM **1**^**+**^ were mixed together in 0.5 M TBAPF_6_/MeCN. Cycling then took place between **SQX-1** and **SQX-1**^•**+**^ on both sides of the H-cell while pyridinium **1**^**+**^ remained in its electrochemically neutral form. Squaramide **SQX-1** showed improved cycling stability under these mixed redoxmer conditions with a slight *increase* in normalized discharge capacity of 1.5% being observed between cycles 1–10 (average 74.8% normalized discharge capacity) and cycles 91–100 (average 75.3% normalized discharge capacity) over the course of 20.2 h (Figure 5a). In addition, there were no major changes in amplitude between pre- and postcycling CVs of the working side of the H-cell (Figure 5c). In a subsequent experiment, H-cell cycling between **1**^**+**^ and **1**^•^ was conducted while squaramide **SQX-1** served as the neutral observer. Under these conditions, high levels of pyridinium cycling stability were observed without any significant decomposition of either the pyridinium anolyte or the squaramide catholyte (Figure SI-80).

**Figure 6:**
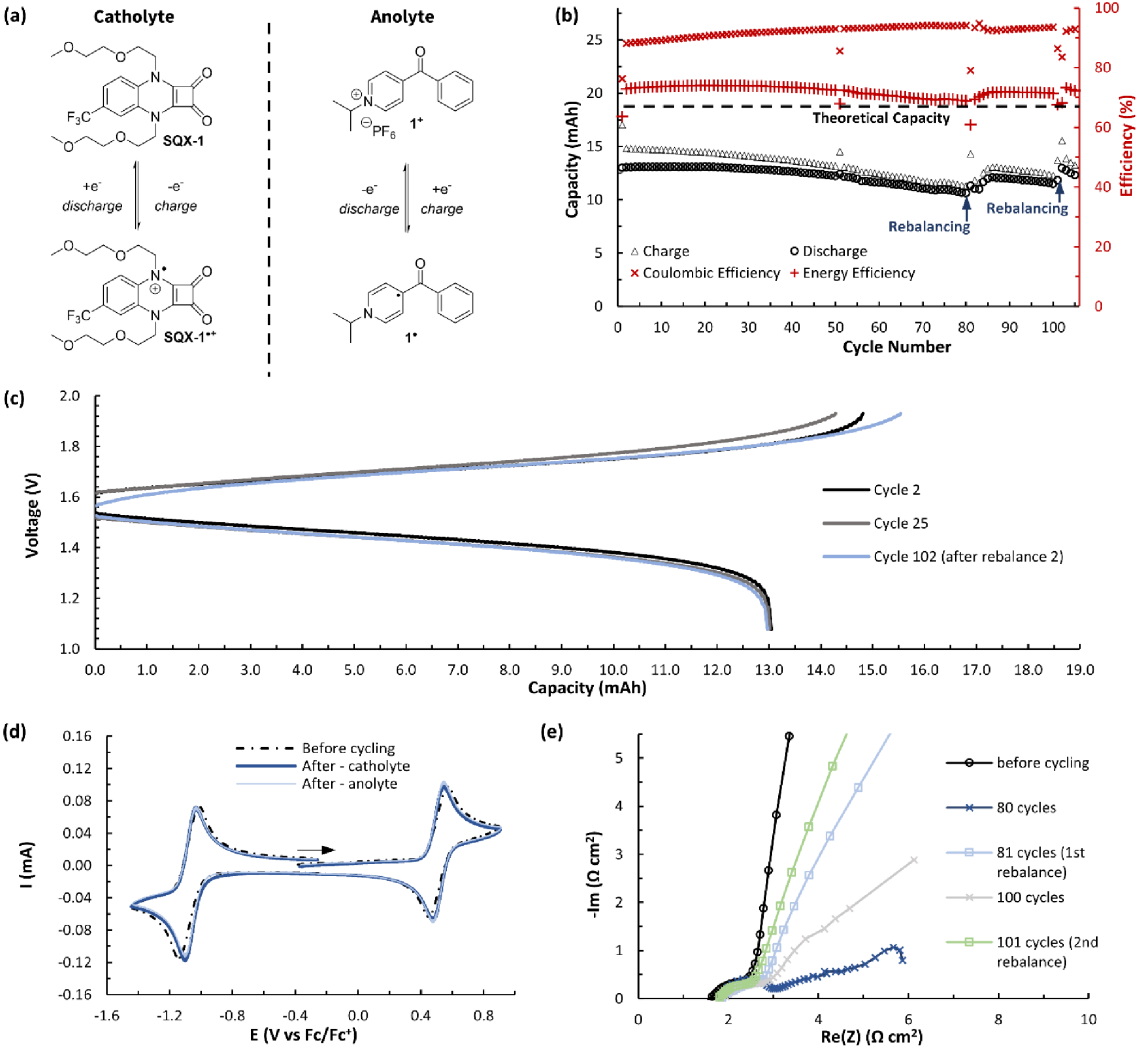
(a) Electrochemical reactions of the anolyte and catholyte during operation of a flow battery. (b) Flow cycling data showing charge and discharge capacities and Coulombic and energy efficiencies vs cycle number for a flow battery made with a mixture of **SQX-1** (100 mM) and **1**^**+**^ (100 mM) in 0.5 M TBAPF_6_/MeCN. Rebalancing of the cell was performed after 80 cycles and 100 cycles. (c) Nernst curves showing potential vs capacity for cycles 2, 25, and 102. (d) CVs (100 mV s^–1^) before and after flow cell cycling of **SQX-1** and **1**^**+**^ for both the anolyte and the catholyte sides of the battery. All solutions were diluted in a 1:19 ratio with 0.5 M TBAPF_6_/MeCN before acquisition. (e) Electrochemical impedance spectroscopy (EIS) on the flow cell before cycling, after 80 cycles, before cycle 81 (after first rebalancing), after cycle 100, and before cycle 101 (after second rebalancing).

With pyridinium (**1**^**+**^) identified as a compatible anolyte, the performance of squaramide **SQX-1** as a catholyte was explored using a flow battery design initially developed and described by Brushett and coworkers^14b,25^ and assembled with a Daramic AA-175 mesoporous separator. The anolyte and catholyte reservoirs were each filled with 7 mL of a mixed solution of 100 mM **SQX-1** and 100 mM **1**^**+**^ in 0.5 M TBAPF_6_/MeCN (Figure 6a). Charging and discharging were performed at flow rates of 20 mL/min and with constant current densities of 20 mA/cm^2^ until reaching voltage cutoffs of 1.93 and 1.08 V (+350 mV and −500 mV, respectively, from the cell’s theoretical Δ*E* of 1.58 V). The initial utilization rate based on discharge capacity was 69% during the first cycle and reached a peak of 70% during the 12th cycle (Figure 6b). After 80 cycles (40.5 h), a rebalance of the battery was performed by manually mixing the catholyte and anolyte reservoirs and by cycle 86, 92% of the peak utilization was recovered. Cycling continued for an additional 14 cycles (100 cycles total) at which point a second rebalancing took place by flowing the discharged cell for 7 h without any electrochemical inputs to allow for the dissipation of concentration gradients. Following this second rebalancing, 99% of the original peak utilized was recovered in cycle 102 following 51 h of active charging/discharging.

Over the course of 105 cycles (52.7 h), the average Coulombic efficiency was 92%, and the average energy efficiency was 72%. Postanalysis CVs of both the anolyte and catholyte reservoirs showed no evidence for the formation of any new electrochemically active species, and there was little change in peak currents as compared to the precycling CV (Figure 6d). In addition, Nernst curves showing potential versus capacity for cycles 2, 25, and 102 show very little deviation (Figure 6c). These data together are consistent with a highly stable battery system and provide evidence that the observed reversible capacity losses are not due to any decomposition of active material but are likely a result of built-up concentration gradients across the two sides of the flow cell, leading to increased cell resistance over time. Rebalancing the cell eliminated these gradients and reestablished the original utilization. This is further supported by Nyquist plots obtained through electrochemical impedance spectroscopy (EIS) that show an increase in electrochemical resistance across the cell electrodes over time that is significantly reduced immediately after rebalancing (Figure 6e).

**Figure 7:**
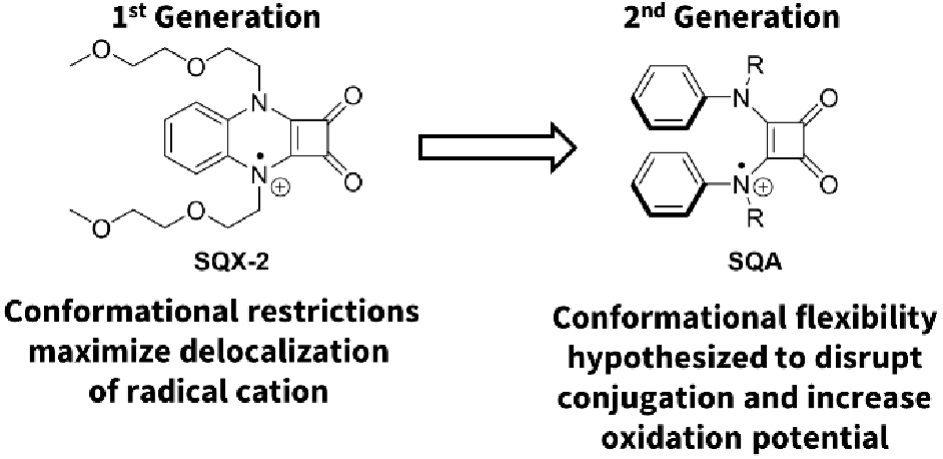
Strategy for the development of our second generation of high oxidation potential squaramides (SQA) based upon a two-aniline core.

Having established SQX catholytes to be an exceptional new class of molecules for nonaqueous redox flow batteries, we turned our attention to further improving their oxidation potential. To do so, we thought about breaking the planarity of the system by swapping out a single diamino benzene ring (**SQX**) for a two-aniline system (**SQA**, Figure 7). Not only would this introduce a second slightly electron withdrawing aromatic ring, but the steric environment of the two aromatic rings would also force them out of complete planarity with the squaramide ring system and might therefore limit radical cation delocalization and increase the corresponding oxidation potential. The second part of this theory was supported by the conformational work of Muthyala and coworkers showing that the electrochemically neutral ground state of bisalkylated diaryl squaramides favors a conformation that allows the two aryl groups to undergo intramolecular π-stacking and as a result, forces the aryl rings to be skewed with respect to the plan of the squaramide ring.^26^

**Figure 8:**
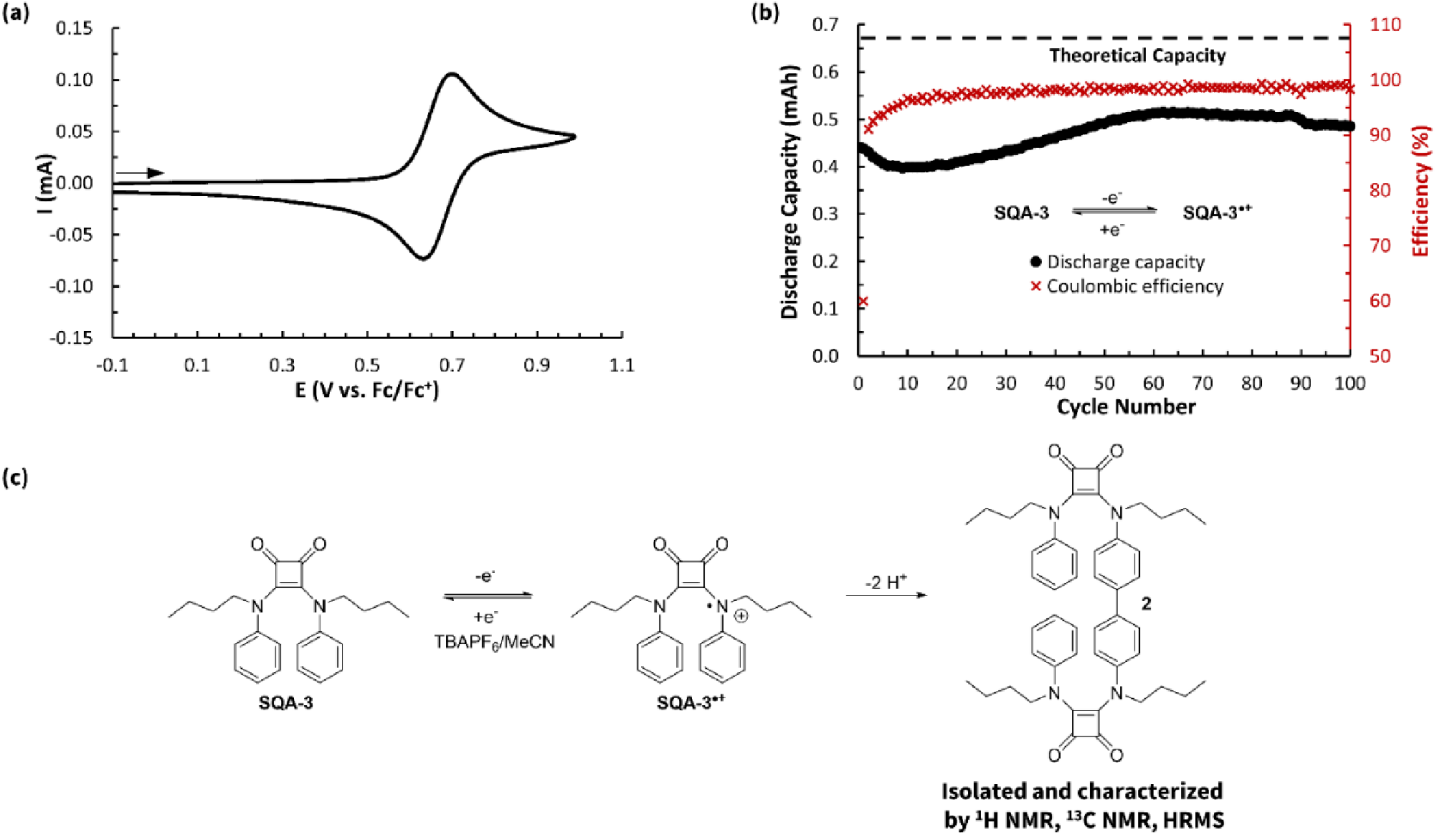
(a) CV of **SQA-3** (5 mM in 0.5 M TBAPF_6_/MeCN) with a scan rate of 100 mV s^–1^. (b) Static oxidative H-cell cycling data between **SQA-3** and **SQA-3**^•**+**^ showing discharge capacity and Coulombic efficiency vs cycle number for 5 mM **SQA-3** in 0.5 M TBAPF_6_/MeCN. (c) Proposed dimerization reaction of the radical cation **SQA-3**^•**+**^.

To test this theory, we synthesized **SQA-3** (Figure 7, R = *n-*butyl) using a similar strategy as for the SQX molecules involving condensation followed by N-alkylation. Consistent with the work of Hünig and Hansmann,^19,20^ this class of squaramide showed a reversible redox couple in cyclic voltammetry experiments with an improved oxidation potential of 0.67 V vs Fc/Fc^+^ (Figure 8a). To test the long-term stability of the resulting radical cation, we subjected this molecule to an oxidative H-cell cycling experiment (cycling between **SQA-3** and **SQA-3**^•**+**^) as previously described with the earlier class of SQX squaramides. Under these conditions, there was a significant initial 6% drop in normalized discharge capacity from 65% to 59% over the first 9 charge–discharge cycles. However, a continuation of cycling showed a subsequent steady increase in capacity, eventually reaching a normalized discharge capacity of 77% during cycle 62 followed by a relatively limited loss of capacity in subsequent cycles (Figure 8b).

This result, showing high levels of stability for what was assumed to be the radical cation of squaramide **SQA-3**, is seemingly at odds with the recent finding of Hansmann and coworkers, who found that the radical cation of a very similar squaramide (from Figure 7, R = Me) showed rapid decomposition in EPR studies with a half-life of around 300 min and which prompted those authors to end their exploration of this class of squaramides.^20^

While the result of Hansmann and coworkers was not public at the time of our finding, we were nonetheless intrigued by the unusual behavior that this molecule was showing in the H-cell cycling experiments and probed further. To ensure the validity of this observed cycling behavior and to ensure that it was not the result of an improper experimental setup or the presence of small impurities in the squaramide not visible via ^1^H NMR, the experiment was repeated two additional times across different batches of **SQA-3** all resulting in the same outcome. This led us to believe that we were observing a real phenomenon. To better understand what was happening, we conducted a postcycling analysis on the cycled material (see Supporting Information for details). This analysis showed almost no **SQA-3** present at the conclusion of the cycling experiments, and instead we identified the major constituent as the biaryl dimer **2** through ^1^H NMR, ^13^C NMR, and HRMS. We propose **2** forms through a biaryl coupling of two squaramide radical cations followed by loss of two protons (Figure 8c). Such a mechanism is well supported in the literature for the dimerization of simple aniline radical cations.^27^ As the relative discharge capacity remained high at cycle 100 when nearly all of the material was in the biaryl form, it can be concluded that the bis radical cation of **2** has high levels of stability. Additionally, a relatively slow formation of this biaryl species would explain the low initial discharge capacity, the apparent loss of capacity over cycles 1–9 that eventually reverses due to the two-electron cycling of biaryl **2**, and the low Coulombic efficiency over cycles 2–9 (91–96%) that eventually approaches 99% by cycle 40. Finally, formation of an initially EPR-silent dimer (**2**) would also explain the 300 min half-life reported by Hansmann for similar SQA molecules.

**Figure 9:**
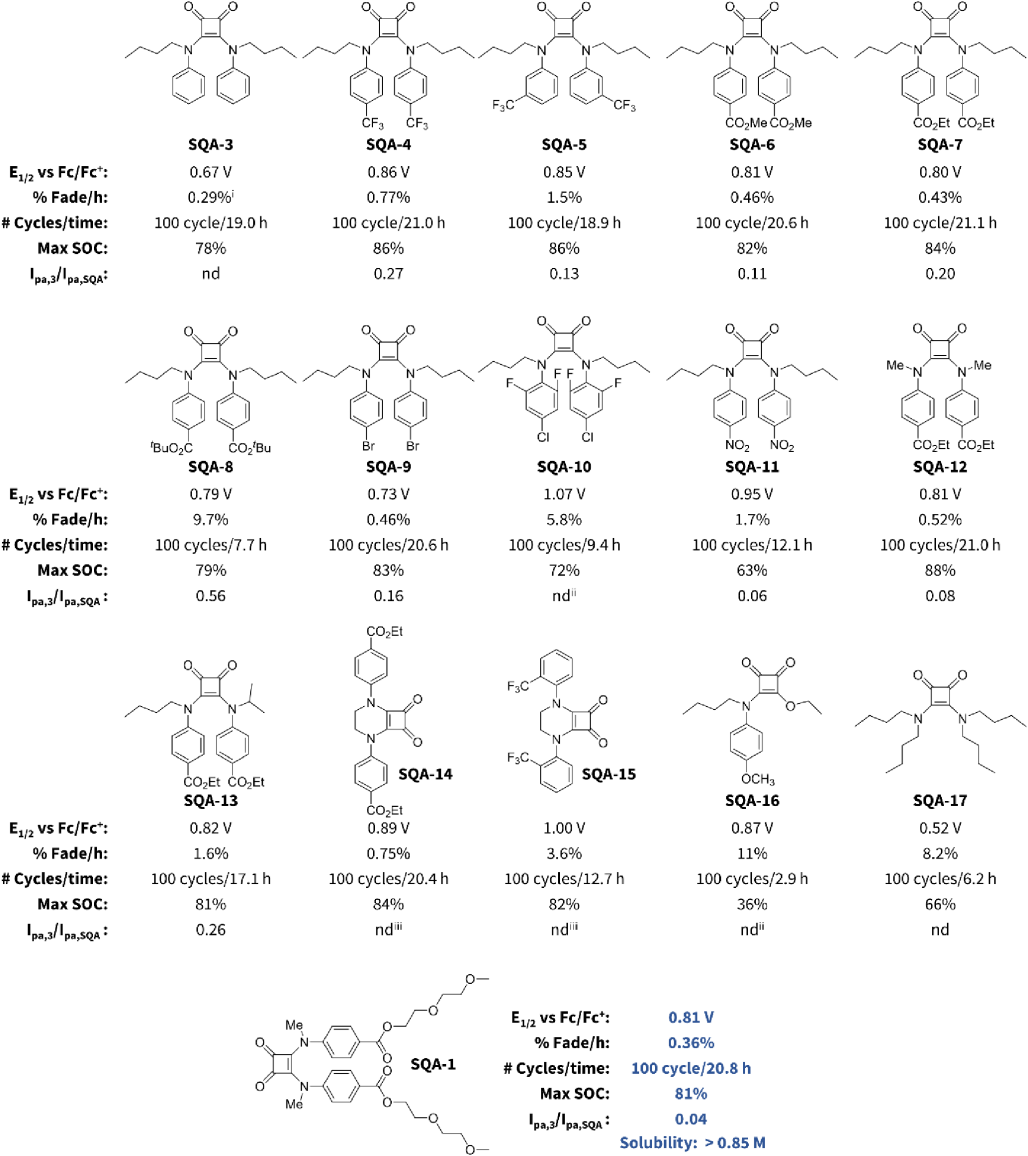
Oxidation potential, one-electron H-cell cycling stability, and ratio of *I*_pa,**3**_/*I*_pa,**SQA**_ after 100 cycles where *I*_cp,**3**_ is the peak anodic current corresponding to the byproduct **3**, and *I*_pa,**SQA**_ is the residual peak anodic current corresponding to the parent **SQA**. Solubility for **SQA-1** was determined by a UV–vis calibration curve in an electrolyte solution of 500 mM TBAPF_6_ in MeCN, per Supporting Information page S26. Notes: nd = not detected. (i) % fade/h following peak discharge capacity. (ii) Significant decomposition made it impossible to definitively detect via postcycling CV. (iii) None could be definitively detected via postcycling CV.

To overcome biaryl dimerization, electron-withdrawing substituents were installed at positions 3 and 4 of the aromatic rings to sterically block radical–radical coupling while simultaneously increasing oxidation potentials (Figure 9, **SQA-4** to **SQA-17**). From this small library, it was found that esters at the 4-position provided the best balance of improved oxidation potential along with high levels of cycling stability (0.80 V vs Fc/Fc^+^ and 0.43% fade/h for **SQA-7**). Within the class of esters, there was little difference between the methyl ester (**SQA-6**) and the ethyl ester (**SQA-7**). However, *t*-butyl ester (**SQA-8**) was observed to undergo spontaneous and rapid decomposition during the H-cell cycling and showed 9.7% capacity fade per hour, which we attribute to an autocatalytic decomposition process likely involving the loss of the *t*-butyl group.

**Figure 10:**
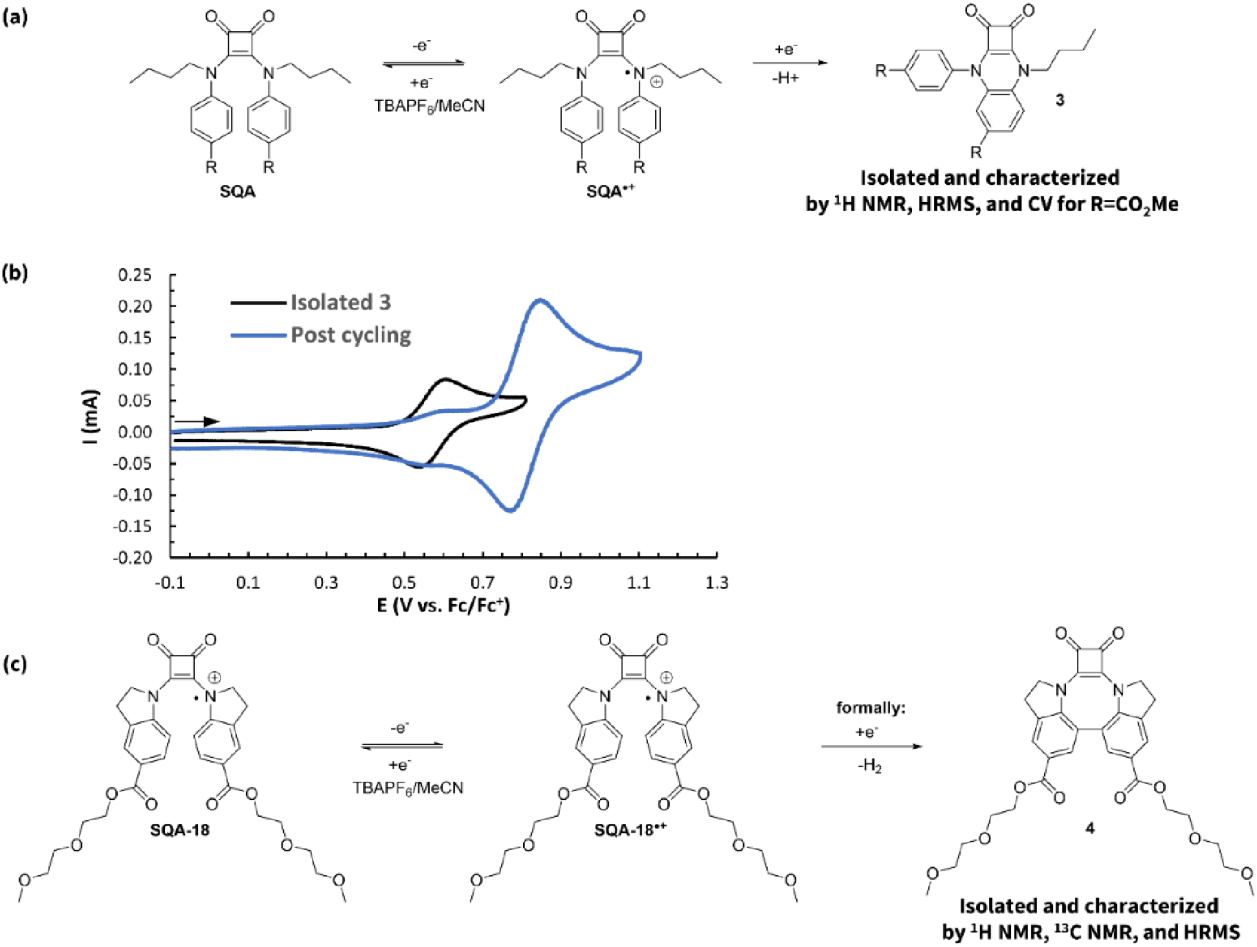
(a) Identification of intramolecular cyclization byproduct **3**. (b) CVs of **3R=CO**_**2**_**Me** (3.5 mM in 0.5 M TBAPF_6_/MeCN) overlaid with a CV of **SQA-6** taken from the working reservoir after 100 static H-cell charge–discharge cycles. Both CVs were acquired at a scan rate of 500 mV s^–1^. (c) Identification of intramolecular biaryl byproduct **4** from the radical cation of indoline squaramide **SQA-18**.

While introducing bulk at the 4-positions solved the issue of dimerization, we began to see a small amount of an alternative byproduct appearing in the Nernst curves during H-cell cycling and in postcycling CVs. The redox couple associated with this byproduct had a lower oxidation potential compared to the starting squaramide, and it appeared to be reversible both on the time-scale of CVs and on the time-scale of our H-cell cycling experiments (Figure 10a,b). To identify the structure of this decomposition product, we conducted a postcycling analysis following 100 H-cell charge–discharge cycles of **SQA-6**. From this, we were able to isolate a couple of milligrams of the byproduct, characterize it via ^1^H NMR and HRMS, and assign its structure to be that of **3**. Cyclic voltammetry confirmed that this isolated material had an oxidation potential that perfectly matched that of the observed byproduct (Figure 10b). Based upon the structure of **3**, we speculate that it forms from the charged radical cation state through an intramolecular cyclization of one squaramide nitrogen onto the aromatic ring of the other nitrogen, dealkylation, and subsequent formal loss of a hydrogen radical, potentially through a proton-coupled electron transfer or as a stepwise process. It should be noted that it is also possible that dealkylation precedes the intramolecular cyclization step and that we do not currently have strong evidence to support either order. As a way to easily quantify how much of this byproduct was being formed for each SQA, we established a parameter measured at the end of 100 H-cell charge–discharge cycles *I*_pa,**3**_/*I*_pa,**SQA**_, where *I*_pa,**3**_ is the peak anodic current corresponding to the byproduct **3**, and *I*_pa,**SQA**_ is the peak anodic current corresponding to the remaining SQA. A lower value of *I*_pa,**3**_/*I*_pa,**SQA**_ is desired and indicates less cyclization.

After identifying the structure of the byproduct with a hypothesized mechanism of formation, we sought to use molecular design to minimize its formation. This is important because while the byproduct itself appears to undergo a reversible oxidation and could continue to function in a battery setting, its lower oxidation potential would decrease the battery voltage over time, and its more 2-dimensional structure as compared to its parent SQA may present solubility issues when formed in higher concentrations. While questions remain regarding the exact mechanism of formation of the byproduct, it must undergo an intramolecular cyclization at some point in the process. We therefore theorized that the two *n*-butyl groups might be helping to promote this step through a Thorpe–Ingold-like effect. We hypothesized that by replacing the *n-*butyl groups with sterically smaller methyl groups, formation of this byproduct could be minimized. Gratifyingly, this was the case with dimethyl squaramide **SQA-12** having an *I*_pa,**3**_/*I*_pa,**SQA**_ of just 0.08 compared to 0.20 in the case of dibutyl squaramide **SQA-7**. To further support this hypothesis, we synthesized squaramide **SQA-13** with a bulky isopropryl group on one squaramide nitrogen and a *n*-butyl group on the other, with the expectation that a bulkier group would lead to more cyclization byproduct. Indeed, this squaramide resulted in an *I*_pa,**3**_/*I*_pa,**SQA**_ of 0.26 following 100 charge–discharge cycles. We also explored the idea of linking both squaramide nitrogen atoms together with an alkyl group to conformationally restrict cyclization (**SQA-14** and **SQA-15**). While this strategy was successful in eliminating any evidence of byproduct formation, these molecules had overall higher capacity fades and were qualitatively observed to have significantly reduced solubilities. As such, this class of molecules was not pursued further.

The final strategy we explored to minimize formation of the undesired byproduct was to reduce the bulk of the alkyl groups by trying them back in the form of an indoline (**SQA-18**, Figure 10c). While this change resulted in no observed byproduct **3**, postcycling CVs showed no evidence for the presence of the starting squaramide, and instead a new redox couple was observed at a higher oxidation potential (see Figure SI-67). Postcycling analysis including ^1^H NMR, ^13^C NMR, and HRMS allowed us to identify this new material as the intramolecular biaryl coupling product **4** (see Supporting Information for details). At present, we are unsure of what causes the indoline squaramides to behave so differently compared with all of the other squaramides tested.

We next sought to explore mixed squaramide/squarate molecules such as **SQA-16** for high oxidation potential catholytes. While their more limited π-system did result in a higher oxidation potential (0.87 V vs Fc/Fc^+^), the stability of its corresponding radical cation proved minimal, and a normalized capacity fade of 11% per hour was observed. As such, this class of molecule was not pursued further.

We further synthesized squaramide **SQA-17** as a control molecule starting from a dialkyl amine. The radical cation of this squaramide has the ability to delocalize charge around the cyclobutene ring and between the two squaramide nitrogens but lacks the ability for further delocalization. This substrate therefore allowed us to isolate the impact of radical cation delocalization around the expended π-system of an aromatic ring on the resulting radical cation stability. While this molecule displayed reversible electron-transfer in CV studies, its radical cation showed poor stability in H-cell charge–discharge studies and exhibited a capacity fade of over 8% per hour, highlighting the importance of extended charge delocalization in the long term cycling stability of squaramide catholytes.

In order to demonstrate the utility of these high oxidation potential squaramides under flow conditions, we synthesized a squaramide derivative (**SQA-1**) containing polar glycol esters at the 4-position of the aromatic rings for improved solubility and to block dimerization while continuing to incorporate the *N,N*′ methyl groups to reduce intramolecular cyclization. This molecule was found to have an oxidation potential of 0.81 V vs Fc/Fc^+^, excellent cycling stability (0.36% fade/h), and a significantly reduced *I*_pa,**3**_/*I*_pa,**SQA**_ of just 0.04 following 100 charge–discharge cycles over the course of 20.8 h.

**Figure 11:**
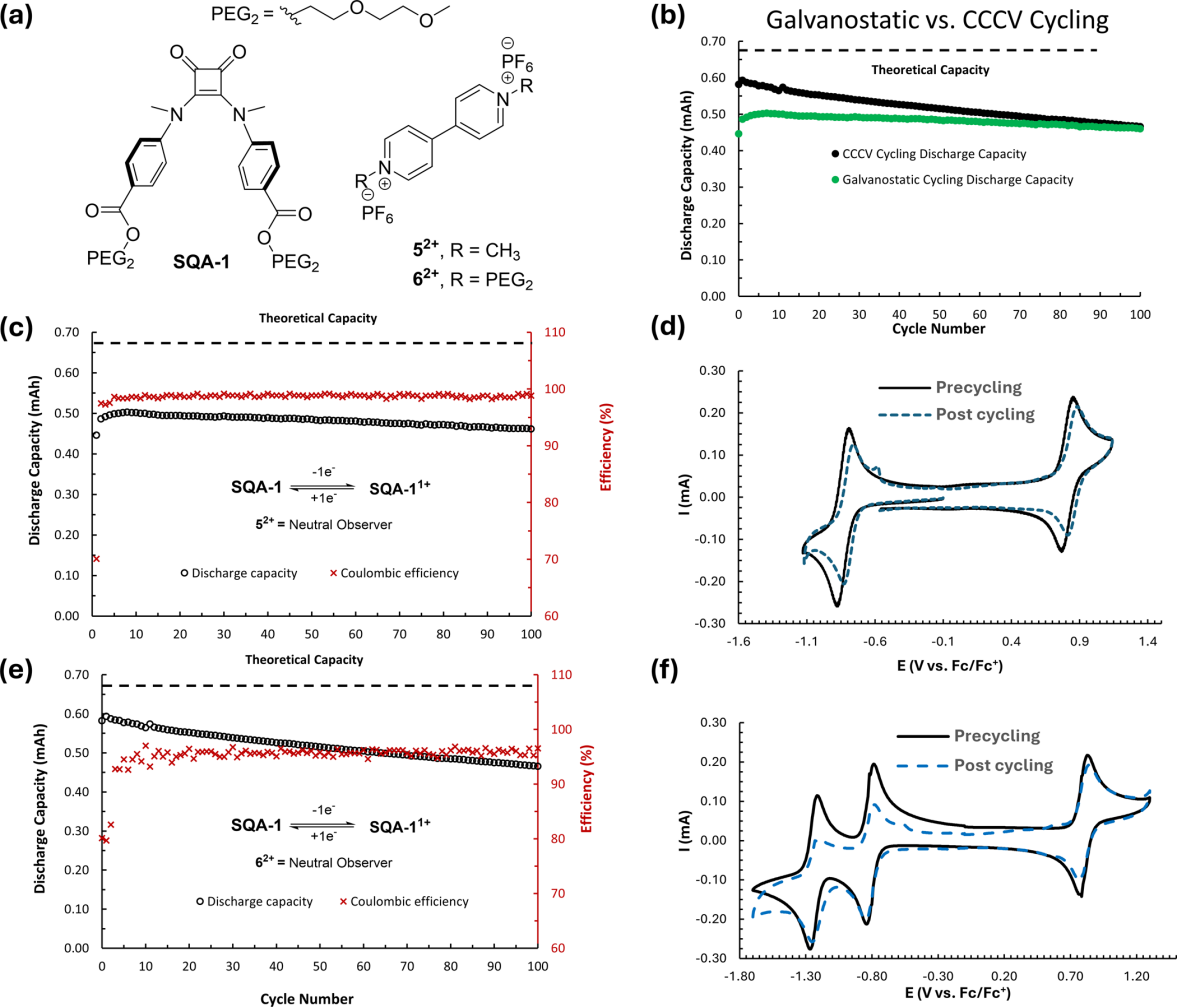
(a) Structures of **SQA-1** and viologens **5**^**2+**^ and **6**^**2+**^. (b) Comparison of discharge capacity vs cycle number for galvanostatic cycling of **SQA-1** and **SQA-1**^•**+**^ for the mixed solution of 5 mM **SQA-1** and 5 mM **5**^**2+**^ in 0.5 M TBAPF_6_/MeCN (100 cycles, 19.6 h), and CCCV cycling of **SQA-1** and **SQA-1**^**+**^ for the mixed solution of 5 mM **SQA-1** and 5 mM **6**^**2+**^ in 0.5 mM TBAPF_6_/MeCN (100 cycles, 29.67 h, cutoffs: + 200 mV from *E*_1/2_, 0.1 mA). (c) Discharge capacity and Coulombic efficiency vs cycle number for galvanostatic cycling from (b). (d) CVs (500 mV s^–1^, glassy carbon electrode) of the working side solution before and after galvanostatic H-cell cycling of **SQA-1** and **SQA-1**^•**+**^ in a mixture of 5 mM **SQA-1** and 5 mM **5**^**2+**^. (e) Discharge capacity and Coulombic efficiency vs cycle number for CCCV cycling from (b). (f) CVs (500 mV s^–1^, glassy carbon electron) of the working side solution before and after galvanostatic H-cell cycling of **SQA-1** and **SQA-1**^•**+**^ in a mixture of 5 mM **SQA-1** and 5 mM **6**^**2+**^. Maximum theoretical discharge capacity is 0.67 mA h

As before, a suitable anolyte was needed that would be compatible with high oxidation potential **SQA-1** in a 50:50 mixed analyte/catholyte flow battery setup. For this, we explored viologen **5**^**2+**^, which has a first reduction potential of −0.83 V vs Fc/Fc^+^.^28^ To ascertain compatibility, a mixture of 5 mM **SQA-1** and 5 mM **5**^**2+**^ in 0.5 M TBAPF_6_/MeCN was exposed to a static H-cell experiment where we first cycled between **SQA-1** with **SQA-1**^•**+**^, while viologen **5**^**+**^ served as an electrochemically neutral observer. Under these mixed conditions, **SQA-1** retained its high level of cycling stability and only small changes in peak currents were observed in postcycling CVs (Figure 11c,d). Similar results were found when viologen **5**^**2+**^ was reductively cycled all the way to its doubly reduced form with squaramide **SQA-1** serving as the electrochemically neutral observer (see Figure SI-86). Since it is possible that purely galvanostatic cycling can lead to resistance artifacts and artificially depress utilization, we also subjected **SQA-1** to a constant current/constant voltage (CCCV) cycling protocol with the higher-solubility **6**^**2+**^ viologen derivative as the electronically neutral observer. We indeed observed increased utilization, from a maximum SOC of 74.8% in purely galvanostatic mode to a maximum SOC of 88.50% in CCCV mode, albeit with substantially increased **6**^**2+**^ degradation as evinced by the postcycling CVs (Figure 11e,f).

**Figure 12:**
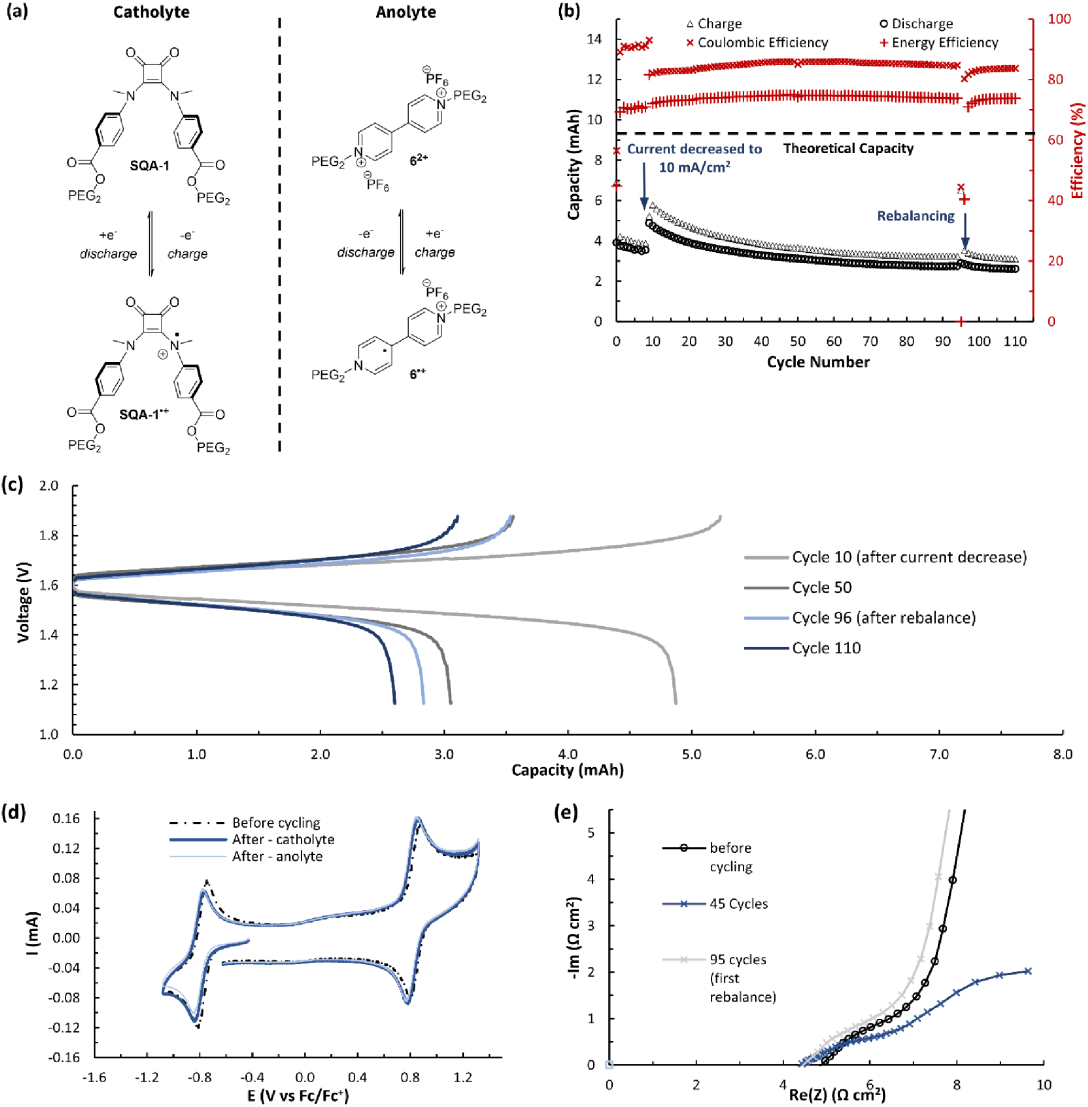
(a) Electrochemical reactions of the anolyte and catholyte during operation of a flow battery. (b) Flow cycling data showing charge and discharge capacities and Coulombic and energy efficiencies vs cycle number for a flow battery made with a mixture of **SQA-1** (50 mM) and **6**^**2+**^ (50 mM) in 0.5 M TBAPF_6_/MeCN. (c) Nernst curves showing potential versus capacity for cycles 10, 25, 95, and 110. (d) CVs (500 mV s^–1^) before and after flow cell cycling of **SQA-1** and **6**^**2+**^ for both the anolyte and catholyte sides of the battery. All solutions were diluted in a 1:19 ratio with 0.5 M TBAPF_6_/MeCN before acquisition. (e) Electrochemical impedance spectroscopy (EIS) on the flow cell before cycling, after 45 cycles, and before cycle 93 (after rebalancing).

Having found **SQA-1** and viologen anolytes to be compatible, we performed flow battery testing using squaramide **SQA-1** as the catholyte and higher solubility viologen **6**^**2+**^ as the one-electron anolyte to ensure solubility and compatibility in this proof-of-concept system (Figure 12).^29^ The flow battery was assembled as described above. The anolyte and catholyte reservoirs were each filled with 7 mL of a mixed solution of 50 mM **SQA-1** and 50 mM **6**^**2+**^ in 0.5 M TBAPF_6_/MeCN. Charging and discharging were performed at flow rates of 20 mL/min and with constant current densities of, initially, 20 mA/cm^2^ for ten cycles before being reduced to 10 mA/cm^2^ for the remaining hundred cycles until reaching voltage cutoffs of 1.88 and 1.38 V (+250 and −250 mV, respectively, from the cell’s theoretical Δ*E* of 1.63 V). Purely galvanostatic cycling was used due to the mesoporous nature of the membrane (Figure 12b). Peak utilization was reached with a charging rate of 10 mA/cm^2^ and reached 51% during cycle ten, immediately after the current was reduced. After 95 cycles (25 h), a rebalance of the battery was performed by manually mixing the catholyte and anolyte solvent/electrolyte mixtures. Cycling continued for an additional 15 cycles (110 cycles and 29 h in total).

Over the first ten cycles run at 20 mA/cm^2^, the average Coulombic efficiency was 92%, and the average energy efficiency was 72%. The subsequent 100 cycles at 10 mA/cm^2^ resulted in an average Coulombic efficiency of 84% and an average energy efficiency of 73% (Figure 12b). While the Nernst curves and cycling data show a decrease in capacity of the battery over time (Figure 12b,c), postanalysis CVs of both the anolyte and catholyte reservoirs, when compared to the initial precycling CVs, displayed near-identical catholyte peak current intensities and showed no evidence for the formation of new electrochemically active species (Figure 12d). This indicates that there was little to no decomposition of **SQA-1** under flow battery conditions and that the loss of battery capacity can be largely accounted for by a slow decomposition of viologen **6**^**2+**^. These results definitively show that **SQA-1** can serve as a high-potential catholyte material for N-ORFBs and that future work in this area should explore the identification of more compatible anolytes and the use of asymmetric battery designs.

**Figure 13:**
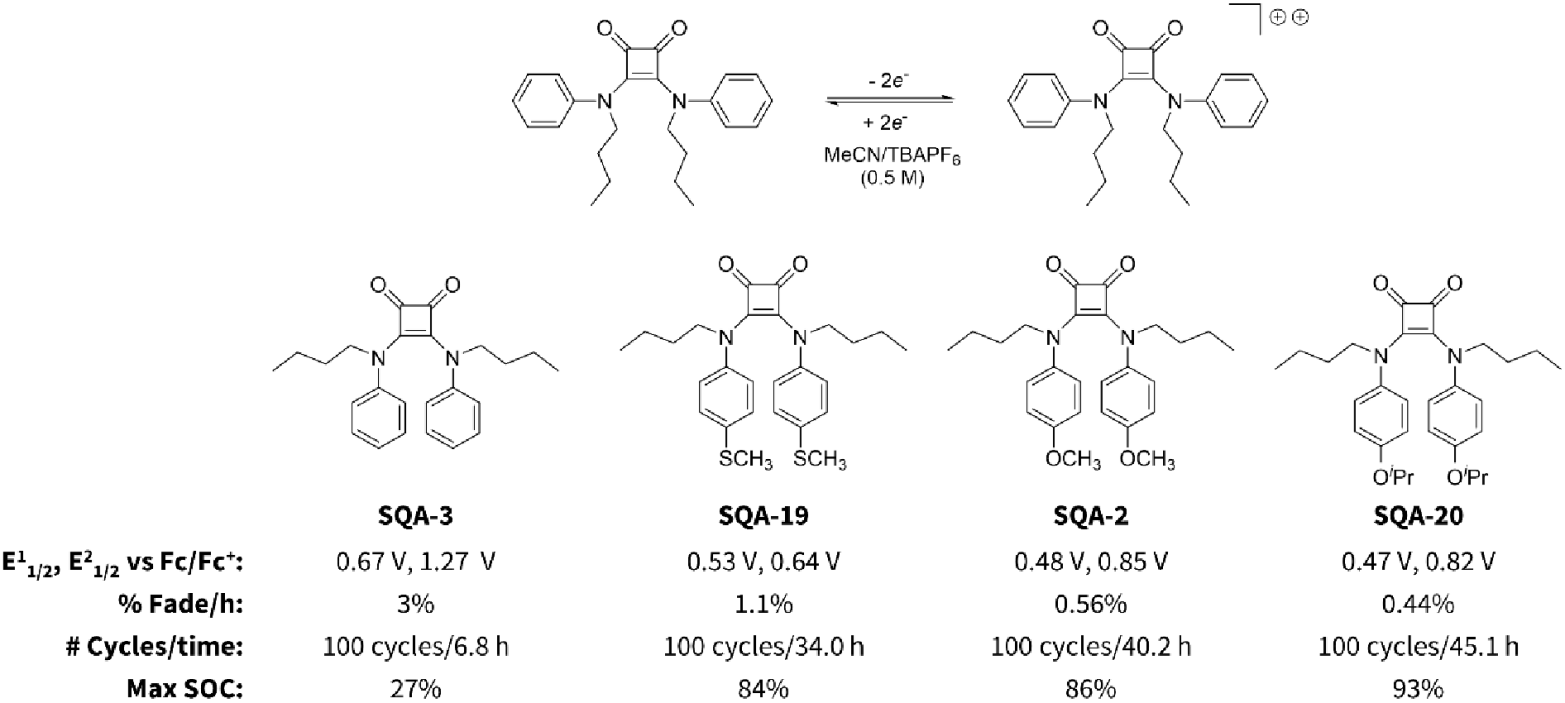
Oxidation potential and two-electron H-cell cycling stability of **SQA 2, 3, 19**, and **20**.

Improving the oxidation potential is just one of the important ways that properties of a high-performance catholyte can be improved. Another is to increase the number of electrons that can be stored within a molecule. Having already demonstrated that a properly substituted SQA molecule can be an excellent high oxidation potential alternative to the SQX class of catholytes, we sought to push the limits of this system further by developing SQAs into two-electron catholytes. While a second oxidation was visible via CV for **SQA-3**, the dicationic species was not electrochemically reversible on the time-scale of CVs or H-cell charge–discharge cycling (Figure 13, 3% fade per hour with a maximum SOC of only 27%). To help stabilize this dication, we sought to introduce electron-donating groups into the structure of the arene, a strategy that has previously proven fruitful in stabilizing doubly oxidized catholytes.^12,30^ Specifically, we sought to install the electron donating groups at the 4-position of the aromatic ring to simultaneously eliminate dimerization and increase stability in the dicationic state while in the singly charged radical cation state.

**Figure 14:**
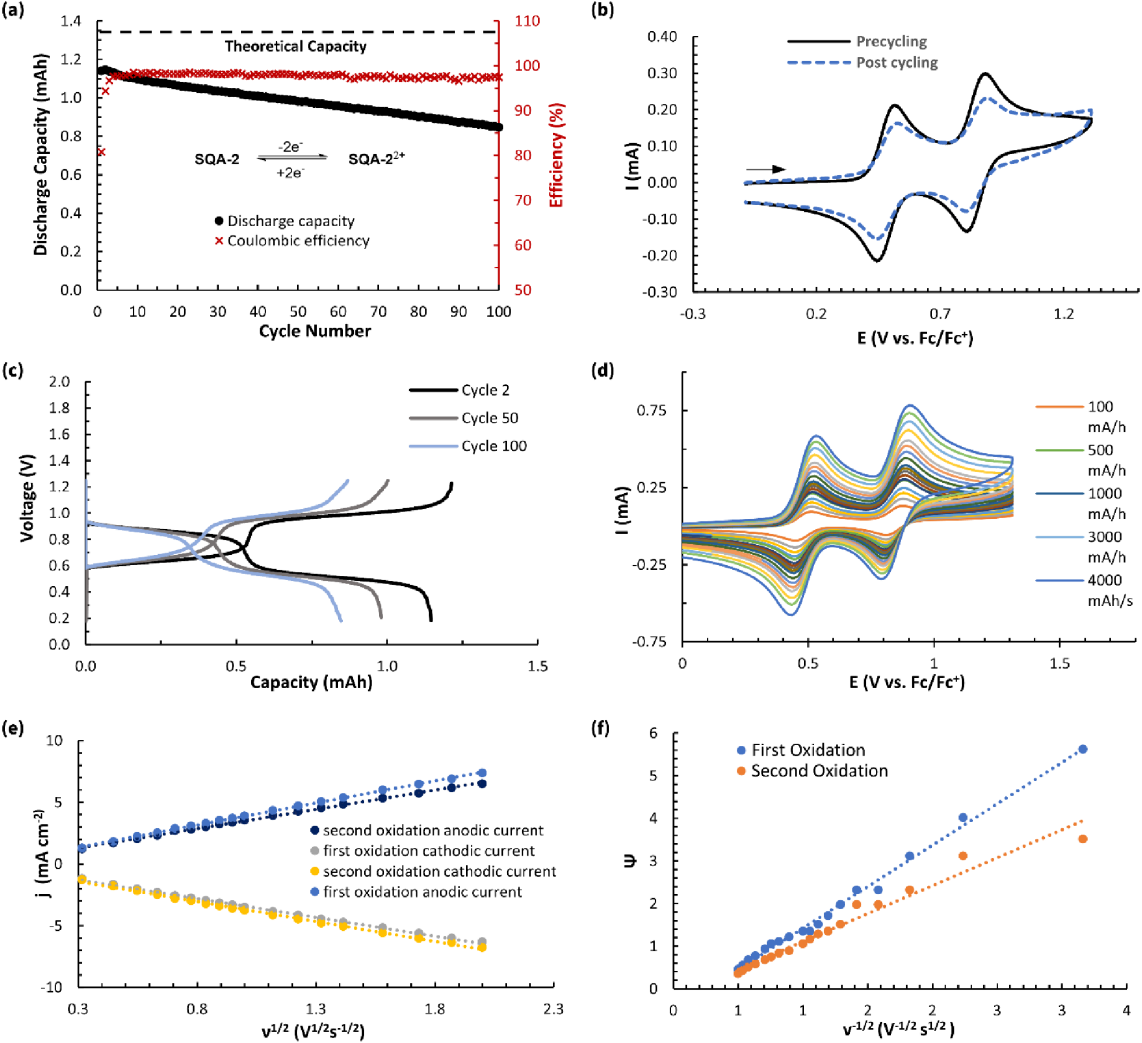
(a) Static oxidative two-electron H-cell cycling data between **SQA-2** and **SQA-2**^**2+**^ (5 mM in 0.5 M TBAPF_6_/MeCN) showing discharge capacity and Coulombic efficiency vs cycle number. (b) Pre- and postcycling static H-cell cycling CV overlay of **SQA-2** (5 mM in 0.5 M TBAPF_6_/MeCN) with a scan rate of 500 mV s^–1^. (c) Nernst curves showing potential vs capacity for cycles 2, 50, and 100. (d) Variable scan CV (5 mM in 0.5 M TBAPF_6_/MeCN) of compound **SQA-2** from 100 to 4000 mV s^–1^. (e) Plots of anodic and cathodic peak current densities (*j*) vs the square root of the sweep rate (ν^1/2^) for **SQA-2**. (f) Nicholson’s dimensionless parameter Ψ vs inverse square root of the sweep rate (ν^–1/2^) for **SQA-2**.

Installation of a thioether at the 4-position (**SQA-19**) resulted in significantly improved stability of the doubly charged dication. However, alkoxy ethers proved better suited for this purpose, with both methoxy-substituted **SQA-2** (0.56% fade/h, Figures 13 and 14a) and isopropoxy-substituted **SQA-20** (0.44% fade/h) showing very promising capacity fades across 100 static H-cell two-electron charge–discharge cycles. Although the percent fade per hour for both **SQA-2** and **SQA-20** were similar, a comparison of pre- and post H-cell cycling CVs showed that the second oxidation peak of the isopropoxy **SQA-20** had a significantly larger reduction in peak current than that observed in the first oxidation. In contrast, the first and second oxidations for methoxy **SQA-2** (Figure 14b) showed similar reductions in peak current, which might indicate less systemic decomposition across this molecule as compared to isopropoxy derivative **SQA-20**. As such, **SQA-2** was declared the lead candidate two-electron SQA catholyte. Subsequent variable scan rate CV studies for the first oxidation yielded a diffusion coefficient (*D*) of 6.1 × 10^–6^ cm^2^ s^–1^ as determined from the Randles–Ševčík equation and with a heterogeneous electron-transfer rate (*k*^0^) of 5.3 × 10^–2^ cm s^–1^ as determined by the Nicholson method while the second oxidation yielded 5.6 × 10^–6^ cm^2^ s^–1^ and 3.4 × 10^–2^ cm s^–1^, respectively (Figure 14 d–f).^22,23^ Taken together, these data clearly indicate that SQA molecules with electron donating groups at position 4 are an exciting new class of two-electron ROMs with potential use in redox flow batteries.

## Conclusion

In summary, we have demonstrated that SQX and SQA molecules are exceptional families of catholytes for redox flow batteries, as demonstrated through the design of three new classes of these molecules, representing both one-electron and two-electron systems. At the initiation of this project, there were no demonstrated functional applications of SQX or SQA molecules in the literature. While the Hansmann group has recently reported a polymeric SQX scaffold for energy storage, our report is the first in the field of flow batteries. Specifically, we designed **SQX-1** bearing a trifluoromethyl group at the 4-position that allowed for improved oxidation potential while glycol ethers imparted high solubility. This molecule showed exceptionally high levels of cycling stability when paired with pyridinium **1**^**+**^. In static H-cell cycling experiments, **SQX-1** showed no loss of discharge capacity over 100 cycles (20+ hours). In flow conditions featuring 100 mM **SQX-1** and **1**^**+**^, a 1.58 V battery was obtained that maintained 99% of peak capacity after 102 cycles (51 h) and reached a peak utilization of 70%.

In addition, we have for the first time demonstrated robust electrochemical stability and reversibility among the SQA class of molecules. Through the thoughtful construction of a small library of such catholytes, we identified three distinct decomposition pathways that reduced the stability of the corresponding radical cations. By using molecular design, we eliminated entirely or significantly minimized these degradation mechanisms, showcasing how detailed decomposition analysis is beneficial to developing and understanding new classes of high stability catholytes that can operate at extreme potentials. These efforts resulted in the design of **SQA-1**, which has an oxidation potential of 0.82 V vs Fc/Fc^+^. We then paired 50 mM **SQA-1** with 50 mM viologen **6**^**2+**^ in a flow battery setup to yield a 1.63 V battery, which over the course of 110 cycles and 29 h of operation underwent negligible amounts of **SQA-1** decomposition as measured by pre- and postcycling CV peak currents. Finally, by installing electron-donating groups at the 4-position of the SQA’s aromatic rings, we developed the first reported stable two-electron SQA catholytes. The most promising such two-electron SQA molecule (**SQA-2**) showcased oxidation potentials of 0.48 and 0.85 V vs Fc/Fc^+^, and its potential application in flow batteries was established through static H-cell cycling where a capacity fade of just 0.56% per hour was observed.

## Supporting Information Available

Experimental procedures and compound characterization; picture of custom H-cell; picture of flow cell during a representative run; H-cell oxidative cycling, two-electron H-cell reductive cycling, and cyclic voltammetry of **3****R=CO_2_Me**, **SQX-1**–**SQX-5**, **SQA-1**–**SQA-20**, **SQX-1** and **1**^+^, **SQA-1** and **5**^2+^ (PDF)
