# Supplementary material for: Development of the Squaramide Scaffold for High Potential and Multielectron Catholytes for Use in Redox Flow Batteries
Source: J Am Chem Soc. 2024 Apr 17;146(17):11740–55. doi: 10.1021/jacs.3c14776 (PMC11066874; doi:10.1021/jacs.3c14776)

Supporting Information

Development of the Squaramide Scaffold for High Potential and Multi-electron Catholytes for use in Redox Flow Batteries

Jacob S. Tracy,[a,b,c,d,†] Conor H. Broderick,[a,b,c,†] F. Dean Toste[a,b,c,*]

[a] Chemical Science Division, Lawrence Berkeley National Laboratory, University of California
Berkeley, CA 94720-1460, United States.
[b] Department of Chemistry, University of California, Berkeley
Berkeley, CA 94720-1460, United States.
[c] Joint Center for Energy Storage Research (JCESR)
Argonne, Illinois 60429, Unites States
[d] Department of Chemistry, University of West Florida
Pensacola, FL 32514, United States
[†]These authors contributed equally to this work.
*fdtoste@berkeley.edu

Table of Contents

I. General Information……………………………………..……...…S2
II. Experimental Procedures and Compound Characterization…....…S3
III. Electrochemical Investigations...……………………...……..…...S25
IV. SI References……..…………………………..…………..…S72
V. Copies of NMR Spectra……………………………………...S74

I. General Information

All reactions were carried out in oven- or flame-dried glassware sealed with rubber septa, kept under a positive pressure of nitrogen, and stirred with a Teflon-coated magnetic stir bar. Unless otherwise noted, reagents were obtained from commercial sources and used without further purification. Dry tetrahydrofuran (THF), toluene, dimethylfomamide (DMF) and acetonitrile (MeCN) used for synthesis were obtained by passing these previously degassed solvents through activated alumina columns under argon. Reactions were monitored by thin layer chromatography (TLC) on Supelco glass backed TLC plates (210-270 μm thickness, 60 Å porosity, F-254 indicator) and visualized by UV irradiation (254 nm) and/or aqueous potassium permanganate solution followed by heating. Solvents were removed under reduced pressure with a rotary evaporator and compounds dried on high vacuum on a Schlenk line.

^{1}H-NMR, ^{13}C-NMR, and ^{19}F-NMR spectra were acquired with Bruker spectrometers operating at 400 or 500 MHz for ^{1}H-NMR, 126 MHz for ^{13}C-NMR and 376 MHz for ^{19}F-NMR. Chemical shifts are reported relative to the residual solvent signal ($CDCl_3$: ^{1}H-NMR: δ = 7.26 ppm; ^{13}C-NMR δ = 77.16 ppm or DMSO: ^{1}H-NMR: δ = 2.50 ppm; ^{13}C-NMR: δ = 39.52 ppm). Multiplicities were indicated with s = singlet, d = doublet, t = triplet, q = quartet, h=heptet, m = multiplet, br = broad resonance. For flash column chromatography, the automatic chromatography system CombiFlash® NextGen 100 (UV light detection: 254 nm and 280 nm) with pre-packed columns (silica gel, 40 μm, 60 Å) was used. The eluting solvents along with the corresponding linear gradients are listed individually for each compound. High-resolution mass spectra (HRMS) were acquired on an Agilent 6230 LC-TOF (ESI) located in the Toste group at the University of California, Berkeley.

II. Experimental Procedures and Compound Characterization

General Synthetic Protocol A:

This procedure is based upon the reported conditions of Taylor and coworkers.[1]

An oven- or flamed-dried round-bottom flask equipped with a magnetic stir bar was charged with the corresponding diaminoarene (1.05 equiv) and zinc trifluoromethanesulfonate (0.20 equiv) and then placed under a nitrogen atmosphere. DMF and toluene in a 1:19 ratio (0.1 M overall in solvent) was then added followed by diethyl squarate via syringe (1.0 equiv). The resulting reaction mixture was heated to 100 °C (oil bath temperature) for 14-20 h overnight. At this point, the rection mixture was cooled to room temperature and the resulting solid material was filtered and washed with water and then a small amount of methanol before being dried under high vacuum. The resulting crude mixture was analyzed by crude ^1H NMR in DMSO-d_6 and then used crude in subsequent steps without purification.

General Synthetic Protocol B:

An oven-dried round-bottom flask equipped with a large magnetic stir bar was charged with the corresponding tricyclic squaramide (1.0 equiv) and dry Cs_2CO_3 (6.0 equiv) and placed under a nitrogen atmosphere. DMF (0.1 M) was added followed by the corresponding alkyl bromide (4.0 equiv). The reaction mixture was then stirred at room temperature for 14-20 h overnight (note: often-times the reaction was complete well before this time period). At this point, the reaction mixture was diluted with water (160 mL per mmol of the squaramide) and repeatedly extracted with toluene (200 mL per mmol of the squaramide) until TLC of the aqueous layer showed nearly all of the desired product had been extracted. This condition was typically found to be met after 2-4 toluene extractions. When significant amounts of water were observed in the combined organic layer, they were transferred to a separatory funnel and solid sodium chloride was added to the funnel which was subsequently shaken and any resulting aqueous layer was removed. An aqueous brine wash was typically avoided due to solubility of some of the alkylated products in aqueous media. The resulting combined organic layer was dried ($MgSO_4$), filtered, concentrated under reduced pressure, and purified via automated flash chromatography (SiO_2). In certain instances, further purification via preparatory TLC was required to obtain analytically pure product.

General Synthetic Protocol C:

This procedure is based upon the reported conditions of Taylor and coworkers and is nearly identical to general synthetic protocol A with modified equivalents.[1]

An oven- or flamed-dried round-bottom flask equipped with a magnetic stir bar was charged with the corresponding aniline (2.1 equiv) and zinc trifluoromethanesulfonate (0.20 equiv) and then placed under a nitrogen atmosphere. DMF and toluene in a 1:19 ratio (0.1 M overall in solvent) was then added followed by diethyl squarate via syringe (1.0 equiv). The resulting reaction mixture was heated to 100 °C (oil bath temperature) for 14-20 h overnight. At this point, the rection mixture was cooled to room temperature and

the resulting solid material was filtered and washed with water and then a small amount of methanol before being dried under high vacuum. The resulting crude mixture was analyzed by crude ^{1}H NMR in DMSO-d_6 and then used crude in subsequent steps without purification.

General Synthetic Protocol D:

Note: This is nearly the same protocol as general synthetic protocol B but with a slightly modified workup procedure.

An oven-dried round-bottom flask equipped with a large magnetic stir bar was charged with the corresponding tricyclic squaramide (1.0 equiv) and dry Cs_2CO_3 (6.0 equiv) and placed under a nitrogen atmosphere. DMF (0.1 M) was added followed by the corresponding alkyl bromide (4.0 or 6.0 equiv). The reaction mixture was then stirred at room temperature for 14-20 h overnight (note: often-times the reaction was complete well before this time period). At this point, the reaction mixture was diluted with water (160 mL per mmol of the squaramide) and repeatedly extracted with toluene (200 mL per mmol of the squaramide) until TLC of the aqueous layer showed nearly all of the desired product had been extracted. This condition was typically found to be met after 2-4 toluene extractions. The combined organic layer was then washed with brine (33% of the volume of the total organics), dried ($MgSO_4$), filtered, concentrated under reduced pressure, and purified via automated flash chromatography (SiO_2). In certain instances, further purification via preparatory TLC was required to obtain analytically pure product.

3,8-dihydrocyclobuta[b]quinoxaline-1,2-dione (**SI-1**)

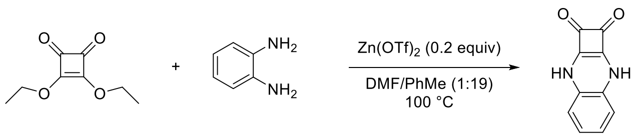

Synthesized according to general synthetic protocol A with diethyl squarate (0.60 mL, 4.0 mmol), *o*-phenylenediamine (450 mg, 4.2 mmol), zinc trifluoromethanesulfonate (290 mg, 0.80 mmol), DMF (0.4 mL), and toluene (7.6 mL). The product was afforded as an orange/brown solid (715 mg, 96% crude yield) and used without further purification.

While the compound was not analytically pure and was carried forward crude, the mixture was characterized by ^{1}H NMR and this crude data is included below and the spectrum is also provided for reference in the appropriate section: ^{1}H NMR (500 MHz, DMSO-d_6) δ 10.00 (s, 2H), 6.64 (dd, J = 5.8, 3.4 Hz, 2H), 6.35 (dd, J = 5.7, 3.4 Hz, 2H). Data are consistent with those reported in the literature.[1]

<u>3,8-bis(2-(2-methoxyethoxy)ethyl)-3,8-dihydrocyclobuta[b]quinoxaline-1,2-dione</u> (**SQX-2**)

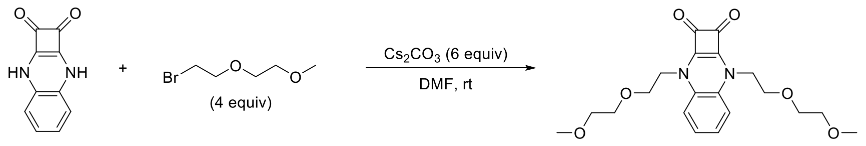

Synthesized according to general synthetic protocol B with squaramide (**SI-1**) (94.1 mg, 0.500 mmol), 1-bromo-2-(2-methoxyethoxy)ethane (0.27 mL, 2.0 mmol), Cs_2CO_3 (0.98 g, 3.0 mmol), and DMF (5 mL). The crude reaction mixture was purified via automated flash chromatography (40 g SiO_2, methanol in ethyl acetate 0 to 5%) followed by preparatory TLC (SiO_2, 1/1/0.1/0.3 toluene/ethyl acetate/methanol/DCM) to yield an orange solid (90 mg, 46% yield).

^{1}H NMR (400 MHz, $CDCl_3$) δ 6.73 (dd, *J* = 5.9, 3.4 Hz, 2H), 6.50 (dd, *J* = 5.9, 3.4 Hz, 2H), 3.81 – 3.73 (m, 4H), 3.73 – 3.69 (m, 4H), 3.68 – 3.64 (m, 4H), 3.54 – 3.48 (m, 4H), 3.35 (s, 6H). ^{13}C NMR (101 MHz, $CDCl_3$) δ 178.2, 174.0, 133.9, 125.3, 114.2, 72.0, 70.5, 67.4, 59.2, 45.6. HRMS (ESI) calculated for $C_{20}H_{26}N_2O_6$ (H+): 391.1864, found: 391.1875.

<u>ethyl 1,2-dioxo-1,2,3,8-tetrahydrocyclobuta[b]quinoxaline-5-carboxylate</u> (**SI-2**)

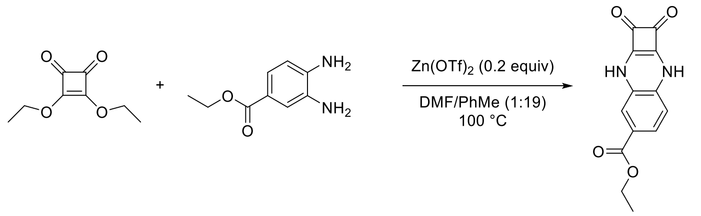

Synthesized according to general synthetic protocol A with diethyl squarate (0.60 mL, 4.0 mmol), ethyl 3,4-diaminobenzoate (757 mg, 4.2 mmol), zinc trifluoromethanesulfonate (290 mg, 0.80 mmol), DMF (0.4 mL), and toluene (7.6 mL). The product was afforded as a black solid (1.02 g, 99% crude yield) and used without further purification.

While the compound was not analytically pure and was carried forward crude, the mixture was characterized by ^{1}H NMR and this crude data is included below and the spectrum is also provided for reference in the appropriate section: ^{1}H NMR (400 MHz, DMSO-d_6) δ 10.33 (s, 1H), 10.16 (s, 1H), 7.26 (dd, *J* = 8.1, 1.8 Hz, 1H), 6.84 (d, *J* = 1.8 Hz, 1H), 6.38 (d, *J* = 8.1 Hz, 1H), 4.21 (q, *J* = 7.1 Hz, 2H), 1.25 (td, *J* = 7.1, 1.0 Hz, 3H).

ethyl 3,8-bis(2-(2-methoxyethoxy)ethyl)-1,2-dioxo-1,2,3,8-tetrahydrocyclobuta[b]quinoxaline-5-carboxylate (**SQX-3**)

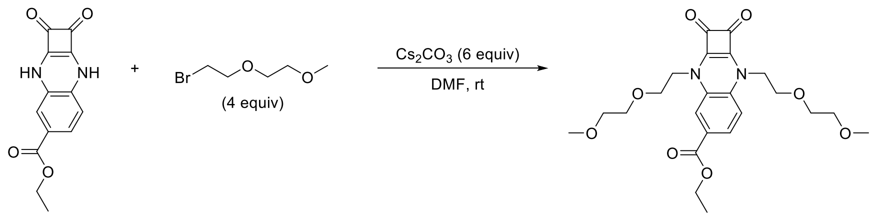

Synthesized according to general synthetic protocol B with squaramide (**SI-2**) (258 mg, 1.00 mmol), 1-bromo-2-(2-methoxyethoxy)ethane (0.54 mL, 4.0 mmol), Cs_2CO_3 (1.95 g, 6.00 mmol), and DMF (10 mL). The crude reaction mixture was purified via two rounds of automated flash chromatography (24 g SiO_2, methanol in ethyl acetate 0 to 5%) to yield an orange solid (123 mg, 27% yield).

^{1}H NMR (400 MHz, $CDCl_3$) δ 7.41 (dd, *J* = 8.3, 1.7 Hz, 1H), 7.03 (d, *J* = 1.8 Hz, 1H), 6.51 (d, *J* = 8.4 Hz, 1H), 3.81 – 3.68 (m, 8H), 3.68 – 3.60 (m, 4H), 3.53 – 3.46 (m, 4H), 3.34 (s, 3H), 3.33 (s, 3H), 1.33 (t, *J* = 7.1 Hz, 3H). ^{13}C NMR (101 MHz, $CDCl_3$) δ 178.4, 177.8, 174.6, 173.5, 164.9, 138.3, 134.1, 127.6, 127.0, 114.1, 113.4, 71.9, 71.9, 70.5, 70.4, 67.4, 66.9, 61.2, 59.0, 59.0, 46.0, 45.7, 14.3. R_f (5% methanol in ethyl acetate) = 0.41. HRMS (ESI) calculated for $C_{23}H_{30}N_2O_8$ (H+): 463.2075, found: 463.2092.

5-(trifluoromethyl)-3,8-dihydrocyclobuta[b]quinoxaline-1,2-dione (**SI-3**)

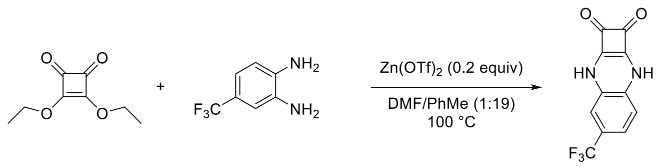

Synthesized according to general synthetic protocol A with diethyl squarate (0.60 mL, 4.0 mmol), 4-(trifluoromethyl)benzene-1,2-diamine (740 mg, 4.2 mmol), zinc trifluoromethanesulfonate (290 mg, 0.80 mmol), DMF (0.4 mL), and toluene (7.6 mL). The product was afforded as a black solid (0.34 g, 34% crude yield) and used without further purification. Note this product has some solubility in methanol and only small amounts should be used for washing purposes.

While the compound was not analytically pure and was carried forward crude, the mixture was characterized by ^{1}H NMR and this crude data is included below and the spectrum is also provided for reference in the appropriate section: ^{1}H NMR (400 MHz, DMSO) δ 10.26 (s, 2H), 7.00 (d, *J* = 8.2 Hz, 1H), 6.48 (d, *J* = 2.0 Hz, 1H), 6.43 (d, *J* = 8.1 Hz, 1H).

3,8-bis(2-(2-methoxyethoxy)ethyl)-5-(trifluoromethyl)-3,8-dihydrocyclobuta[b]quinoxaline-1,2-dione (**SQX-1**)

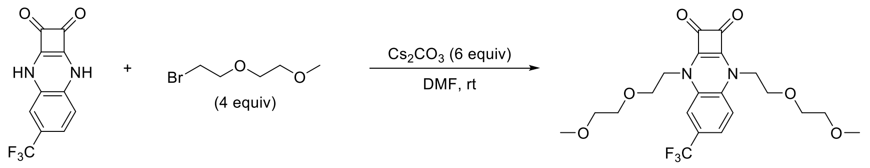

1 mmol scale: Synthesized according to general synthetic protocol B with squaramide (**SI-3**) (254 mg, 1.00 mmol), 1-bromo-2-(2-methoxyethoxy)ethane (0.54 mL, 4.0 mmol), Cs_2CO_3 (1.95 g, 6.00 mmol), and DMF (10 mL). The crude reaction mixture was purified via automated flash chromatography (24 g SiO_2, 100% ethyl acetate) followed by preparatory TLC (SiO_2, 100% ethyl acetate) to yield a dark orange solid (121 mg, 26% yield).

5 mmol scale: Synthesized according to general synthetic protocol B with squaramide (**SI-3**) (1.27 g, 5.00 mmol), 1-bromo-2-(2-methoxyethoxy)ethane (2.69 mL, 20 mmol), Cs_2CO_3 (9.77 g, 30.0 mmol), and DMF (50 mL). The crude reaction mixture was purified via automated flash chromatography (80 g SiO_2, 70 to 100% ethyl acetate in hexanes) to yield a dark orange solid (565 mg, 25% yield).

^{1}H NMR (400 MHz, $CDCl_3$) δ 6.97 (d, *J* = 8.3 Hz, 1H), 6.72 (d, *J* = 2.2 Hz, 1H), 6.55 (d, *J* = 8.4 Hz, 1H), 3.76 (t, *J* = 4.2 Hz, 4H), 3.73 – 3.68 (m, 4H), 3.68 – 3.61 (m, 4H), 3.54 – 3.46 (m, 4H), 3.34 (s, 3H), 3.33 (s, 3H). ^{13}C NMR (126 MHz, $CDCl_3$) δ 178.5, 178.1, 174.5, 174.3, 137.6, 135.5, 127.4 (q, $J_{C\text{-}F}$ = 33.3 Hz), 123.4 (q, $J_{C\text{-}F}$ = 271.8 Hz), 124.5 (d, $J_{C\text{-}F}$ = 271.8 Hz), 122.7 (q, $J_{C\text{-}F}$ = 4.2 Hz), 113.8, 111.1 (q, $J_{C\text{-}F}$ = 3.7 Hz), 72.1, 72.0, 70.8, 70.7, 59.2, 59.2, 46.4, 46.2.^{19}F NMR (376 MHz, $CDCl_3$) δ -62.7. R_f (100% ethyl acetate) = 0.28. HRMS (ESI) calculated for $C_{21}H_{25}F_3N_2O_6$ (H+): 459.1738, found: 459.1751.

diethyl 1,2-dioxo-1,2,3,8-tetrahydrocyclobuta[b]quinoxaline-5,6-dicarboxylate (**SI-4**)

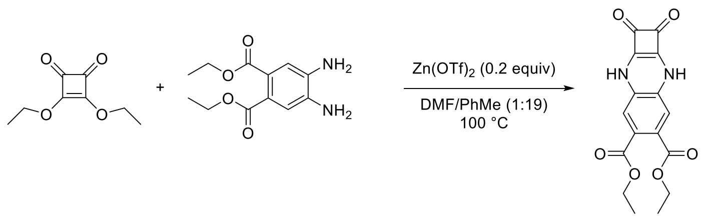

Synthesized according to general synthetic protocol A with diethyl squarate (0.19 mL, 1.3 mmol), diethyl 4,5-diaminophthalate (340 mg, 1.4 mmol), zinc trifluoromethanesulfonate (93 mg, 0.28 mmol), DMF (0.13 mL), and toluene (2.4 mL). The product was afforded as an orange solid (275 mg, 62% crude yield) and used without further purification.

While the compound was not analytically pure and was carried forward crude, the mixture was characterized by ^{1}H NMR and this crude data is included below and the spectrum is also provided for reference in the appropriate section: ^{1}H NMR (400 MHz, DMSO-d_6) δ 10.32 (s, 2H), 6.50 (s, 2H), 4.18 (q, *J* = 7.1 Hz, 4H), 1.21 (t, *J* = 7.1 Hz, 6H).

diethyl 3,8-bis(2-(2-methoxyethoxy)ethyl)-1,2-dioxo-1,2,3,8-tetrahydrocyclobuta[b]quinoxaline-5,6-dicarboxylate (**SQX-4**)

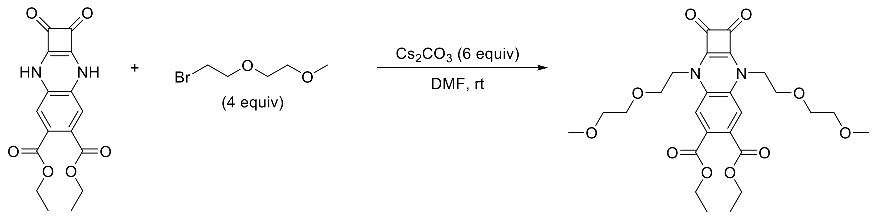

Synthesized according to general synthetic protocol B with squaramide (**SI-4**) (254 mg, 0.770 mmol), 1-bromo-2-(2-methoxyethoxy)ethane (0.41 mL, 3.1 mmol), Cs_2CO_3 (1.50 g, 4.61 mmol), and DMF (7.7 mL). The crude reaction mixture was purified via automated flash chromatography (16 g SiO_2, methanol in ethyl acetate 0 to 5%) followed by preparatory TLC (SiO_2, 5% methanol in ethyl acetate) to yield an orange solid (129 mg, 31% yield).

^{1}H NMR (500 MHz, $CDCl_3$) δ 6.71 (s, 2H), 4.27 (q, *J* = 7.1 Hz, 4H), 3.74 (t, *J* = 4.9 Hz, 4H), 3.70 – 3.66 (m, 4H), 3.65 – 3.59 (m, 4H), 3.51 – 3.46 (m, 4H), 3.32 (s, 6H), 1.30 (t, *J* = 7.2 Hz, 6H). ^{13}C NMR (126 MHz, $CDCl_3$) δ 178.3, 174.4, 166.1, 136.8, 129.4, 113.9, 72.0, 70.7, 67.4, 61.9, 59.1, 46.2, 14.1. R_f (5% methanol in ethyl acetate) = 0.35. HRMS (ESI) calculated for $C_{26}H_{34}N_2O_{10}$ (H+): 535.2286, found: 535.2301.

ethyl 3,8-bis(2-(dimethylamino)ethyl)-1,2-dioxo-1,2,3,8-tetrahydrocyclobuta[b]quinoxaline-5-carboxylate (**SI-5**)

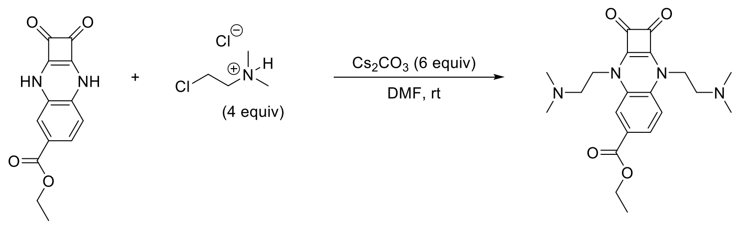

Synthesized according to a slight modification (number of equivalents of electrophile) of general synthetic protocol B with squaramide (**SI-2**) (258 mg, 1.00 mmol), 2-chloro-*N,N*-dimethylethylamine hydrochloride (317 mg, 2.2 mmol), Cs_2CO_3 (1.95 g, 6.00 mmol), and DMF (10 mL). In another deviation from the general protocol, the reaction mixture was allowed to stir for 40 h at room temperature. The crude reaction mixture was purified via automated flash chromatography (40 g SiO_2, methanol in ethyl acetate 0 to 50%) to yield a yellow/orange oil (116 mg, 29% yield) that was quickly carried forward to the alkylation step.

^{1}H NMR (400 MHz, $CDCl_3$) δ 7.41 (dd, *J* = 8.3, 1.7 Hz, 1H), 6.96 (d, *J* = 1.8 Hz, 1H), 6.39 (d, *J* = 8.3 Hz, 1H), 4.26 (q, *J* = 7.1 Hz, 2H), 3.57 (t, *J* = 6.3 Hz, 4H), 2.53 (q, *J* = 6.6 Hz, 4H), 2.25 (s, 6H), 2.24 (s, 7H), 1.30 (t, *J* = 7.1 Hz, 3H). ^{13}C NMR (101 MHz, $CDCl_3$) δ 178.5, 177.8, 174.5, 173.5, 164.9, 138.2, 134.2, 127.7, 127.0, 113.9, 113.0, 61.3, 55.5, 55.4, 45.7, 45.7, 44.5, 44.2, 14.3. R_f (50% methanol in ethyl acetate) = 0.28.

2,2'-(5-(ethoxycarbonyl)-1,2-dioxo-1,2-dihydrocyclobuta[b]quinoxaline-3,8-diyl)bis(N,N,N-trimethylethan-1-aminium) bis(hexafluorophosphate) (**SQX-5**)

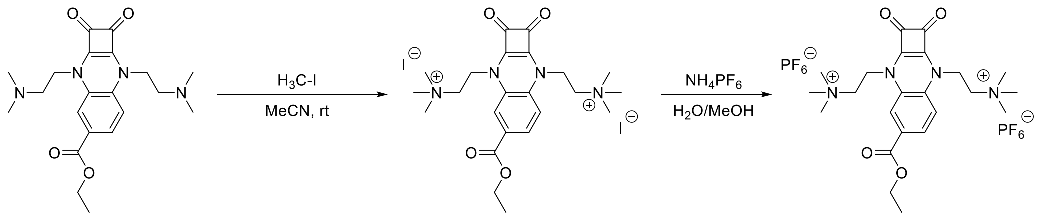

An oven-dried 25 mL round-bottom flask equipped with a magnetic stir bar was charged with squaramide **SI-5** (116 mg, 0.290 mmol) and placed under a nitrogen atmosphere. MeCN (3 mL) was added followed by methyl iodide (42 µL, 0.67 mmol) via syringe. The reaction mixture was allowed to stir at room temperature for 42 h at which point the volatiles were removed with a stream of nitrogen followed by high-vacuum. The resulting crude material was then dissolved in 4.5 mL of water with just enough methanol to obtain full solubility (approximately 3.5 mL). Ammonium hexafluorophosphate (540 mg, 3.3 mmol) was then added and the mixture was stirred vigorously for 20 minutes. The resulting precipitate was filtered and dried under high vacuum to yield a yellow solid (145 mg, 20% yield) that did not require further purification.

^{1}H NMR (400 MHz, CD_3CN) δ 7.54 (d, *J* = 8.3 Hz, 1H), 7.06 (s, 1H), 6.66 (d, *J* = 8.3 Hz, 1H), 4.30 (q, *J* = 7.1 Hz, 2H), 3.99 (dt, *J* = 11.1, 7.1 Hz, 4H), 3.61 (q, *J* = 7.7 Hz, 4H), 3.15 (s, 18H), 1.33 (t, *J* = 7.1 Hz, 3H). ^{13}C NMR (126 MHz, $CDCl_3$) δ 179.4, 179.0, 174.9, 174.2, 165.4, 138.1, 134.4, 129.0, 128.9, 115.0, 115.0, 62.8, 62.6, 62.3, 54.5, 54.5, 41.1, 40.9, 14.5. ^{19}F NMR (376 MHz, CD_3CN) δ -71.81 (d, $J_{P\text{-}F}$ = 707.3 Hz). HRMS (ESI) m/z calculated for $C_{23}H_{34}N_4O_4$ (2+): 215.1285, found: 215.1283.

3,4-bis(phenylamino)cyclobut-3-ene-1,2-dione (**SI-6**)

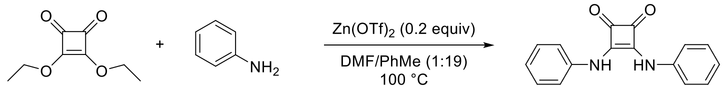

Synthesized according to general synthetic protocol C with diethyl squarate (0.30 mL, 2.0 mmol), aniline (0.38 mL, 4.2 mmol), zinc trifluoromethanesulfonate (145 mg, 0.400 mmol), DMF (0.2 mL), and toluene (3.8 mL). The product was afforded as a yellow solid (459 mg, 87% crude yield) and used without further purification.

While the compound was not analytically pure and was carried forward crude, the mixture was characterized by ^{1}H NMR and this crude data is included below and the spectrum is also provided for reference in the appropriate section: ^{1}H NMR (400 MHz, DMSO-d_6) δ 9.89 (s, 2H), 7.50 (d, *J* = 8.0 Hz, 4H), 7.39 (t, *J* = 7.9 Hz, 4H), 7.09 (t, *J* = 7.3 Hz, 2H). Data are consistent with those reported in the literature.[2]

3,4-bis(butyl(phenyl)amino)cyclobut-3-ene-1,2-dione (**SQA-3**)

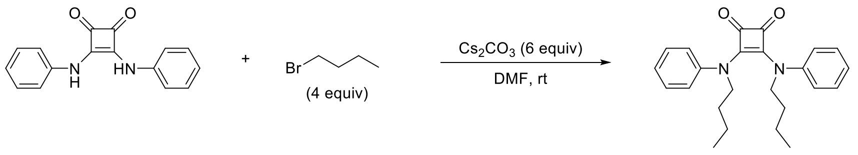

Synthesized according to general synthetic protocol D with squaramide (**SI-6**) (133 mg, 0.500 mmol), 1-bromobutane (0.22 mL, 2.0 mmol), Cs_2CO_3 (0.98 g, 3.0 mmol), and DMF (5 mL). The crude reaction mixture was purified via automated flash chromatography (24 g SiO_2, ethyl acetate in hexanes 0 to 20%) to yield a pale yellow solid (88 mg, 48% yield).

^{1}H NMR (400 MHz, $CDCl_3$) δ 6.96 – 6.89 (m, 4H), 6.89 – 6.81 (m, 2H), 6.45 (d, J = 8.1 Hz, 4H), 4.08 – 3.98 (m, 4H), 1.62 – 1.49 (m, 4H), 1.33 (dq, J = 14.7, 7.3 Hz, 4H), 0.86 (t, J = 7.4 Hz, 6H). ^{13}C NMR (101 MHz, $CDCl_3$) δ 186.4, 167.6, 141.4, 128.7, 125.1, 122.5, 52.5, 32.0, 19.7, 13.8. R_f (hexanes/ethyl acetate 5:1) = 0.46. HRMS (ESI) m/z calculated for $C_{24}H_{28}N_2O_2$ (H+): 377.2224, found: 377.2236.

3,4-bis((4-(trifluoromethyl)phenyl)amino)cyclobut-3-ene-1,2-dione (**SI-7**)

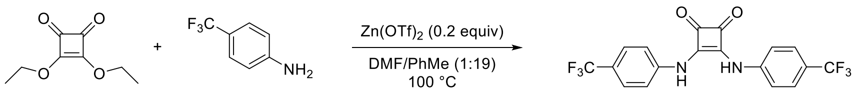

Synthesized according to general synthetic protocol C with diethyl squarate (0.60 mL, 4.0 mmol), 4-(trifluoromethyl)aniline (1.05 mL, 8.4 mmol), zinc trifluoromethanesulfonate (290 mg, 0.80 mmol), DMF (0.4 mL), and toluene (7.6 mL). The product was afforded as a pale yellow solid (1.09 g, 68% crude yield) and used without further purification.

While the compound was not analytically pure and was carried forward crude, the mixture was characterized by ^{1}H NMR and this crude data is included below and the spectrum is also provided for reference in the appropriate section: ^{1}H NMR (400 MHz, DMSO-d_6) δ 10.28 (s, 2H), 7.74 (d, J = 8.4 Hz, 4H), 7.63 (d, J = 8.5 Hz, 4H). Data are consistent with those reported in the literature.[3]

3,4-bis(butyl(4-(trifluoromethyl)phenyl)amino)cyclobut-3-ene-1,2-dione (**SQA-4**)

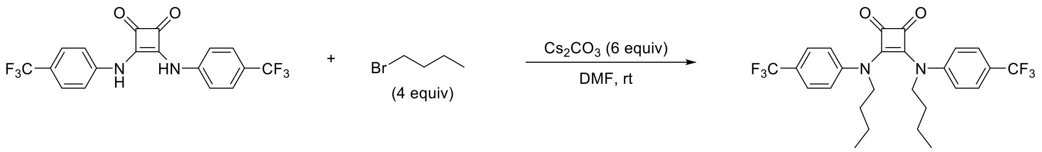

Synthesized according to general synthetic protocol D with squaramide (**SI-7**) (200. mg, 0.500 mmol), 1-bromobutane (0.22 mL, 2.0 mmol), Cs_2CO_3 (0.98 g, 3.0 mmol), and DMF (5 mL). The crude reaction mixture was purified via automated flash chromatography (24 g SiO_2, ethyl acetate in hexanes 0 to 20%) to yield a white solid (114 mg, 44% yield).

^{1}H NMR (400 MHz, $CDCl_3$) δ 7.20 (d, J = 8.2 Hz, 4H), 6.57 (d, J = 8.2 Hz, 4H), 4.13 (t, J = 7.5 Hz, 4H), 1.71 – 1.54 (m, 4H), 1.37 (h, J = 7.2 Hz, 4H), 0.91 (t, J = 7.3 Hz, 6H). ^{13}C NMR (126 MHz, $CDCl_3$) δ 186.6, 167.3, 144.0, 127.20 (q, $J_{C\text{-}F}$ = 33.1 Hz), 123.66 (q, $J_{C\text{-}F}$ = 271.7 Hz), 122.4, 52.3, 32.1, 19.8, 13.8. ^{19}F NMR (470 MHz, $CDCl_3$) δ -62.7. R_f (hexanes/ethyl acetate 5:1) = 0.27. HRMS (ESI) m/z calculated for $C_{26}H_{26}F_6N_2O_2$ (H+): 513.1971, found: 513.1980.

3,4-bis((3-(trifluoromethyl)phenyl)amino)cyclobut-3-ene-1,2-dione (**SI-8**)

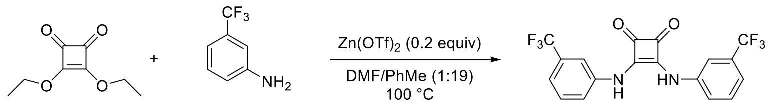

Synthesized according to general synthetic protocol C with diethyl squarate (0.60 mL, 4.0 mmol), 3-(trifluoromethyl)aniline (1.05 mL, 8.40 mmol), zinc trifluoromethanesulfonate (290 mg, 0.80 mmol), DMF (0.4 mL), and toluene (7.6 mL). The product was afforded as an off-white solid (1.29 g, 81% crude yield) and used without further purification.

While the compound was not analytically pure and was carried forward crude, the mixture was characterized by ^{1}H NMR and this crude data is included below and the spectrum is also provided for reference in the appropriate section: ^{1}H NMR (400 MHz, DMSO-D_6) δ 10.18 (s, 2H), 7.90 (s, 2H), 7.66 – 7.56 (m, 4H), 7.42 (d, J = 7.0 Hz, 2H).

3,4-bis(butyl(3-(trifluoromethyl)phenyl)amino)cyclobut-3-ene-1,2-dione (**SQA-5**)

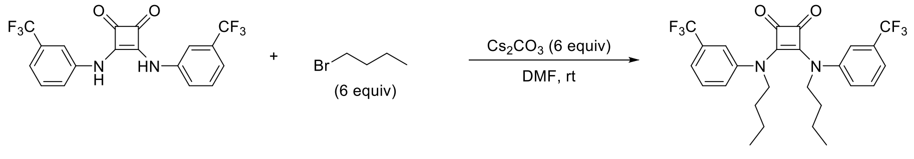

Synthesized according to general synthetic protocol D with squaramide (**SI-8**) (200. mg, 0.500 mmol), 1-bromobutane (0.32 mL, 3.0 mmol), Cs_2CO_3 (0.98 g, 3.0 mmol), and DMF (5 mL). The crude reaction mixture was purified via automated flash chromatography (24 g SiO_2, ethyl acetate in hexanes 0 to 20%) to yield a white solid (129 mg, 50% yield).

^{1}H NMR (400 MHz, $CDCl_3$) δ 7.16 – 7.04 (m, 4H), 6.75 – 6.65 (m, 4H), 4.11 (t, J = 7.5 Hz, 4H), 1.70 – 1.53 (m, 4H), 1.36 (h, J = 7.4 Hz, 4H), 0.89 (t, J = 7.3 Hz, 6H). ^{13}C NMR (126 MHz, $CDCl_3$) δ 186.50, 167.17, 141.68, 131.17 (q, $J_{C\text{-}F}$ = 32.8 Hz), 129.55, 124.91, 123.37 (q, $J_{C\text{-}F}$ = 272.6 Hz), 121.94 (q, $J_{C\text{-}F}$ = 3.6 Hz), 118.88 (q, $J_{C\text{-}F}$ = 3.7 Hz), 52.48, 32.09, 19.73, 13.74. ^{19}F NMR (470 MHz, $CDCl_3$) δ -63.0. HRMS (ESI) m/z calculated for $C_{26}H_{26}F_6N_2O_2$ (H+): 513.1971, found: 513.1986.

dimethyl 4,4'-((3,4-dioxocyclobut-1-ene-1,2-diyl)bis(azanediyl))dibenzoate (**SI-9**)

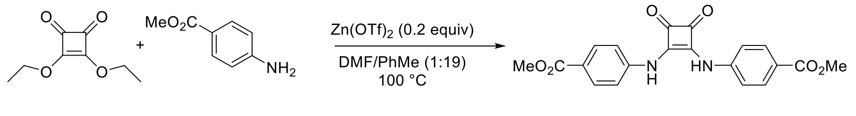

Synthesized according to general synthetic protocol C with diethyl squarate (0.60 mL, 4.0 mmol), methyl 4-aminobenzoate (1.27 g, 8.40 mmol), zinc trifluoromethanesulfonate (290 mg, 0.80 mmol), DMF (0.4 mL), and toluene (7.6 mL). The product was afforded as an off-white solid (1.47 g, 97% crude yield) and used without further purification.

While the compound was not analytically pure and was carried forward crude, the mixture was characterized by ^{1}H NMR and this crude data is included below and the spectrum is also provided for reference in the appropriate section: ^{1}H NMR (400 MHz, DMSO-d$_6$) δ 10.25 (s, 2H), 7.97 (d, *J* = 8.5 Hz, 4H), 7.59 (d, *J* = 8.4 Hz, 4H), 3.84 (s, 6H).

dimethyl 4,4'-((3,4-dioxocyclobut-1-ene-1,2-diyl)bis(butylazanediyl))dibenzoate (**SQA-6**)

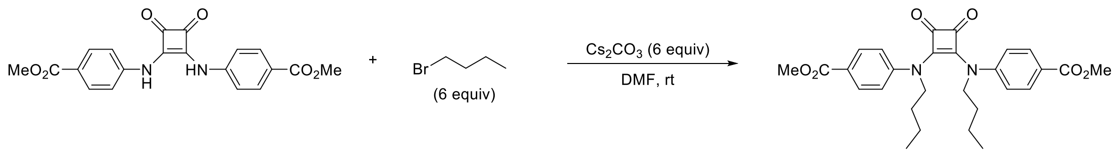

Synthesized according to general synthetic protocol D with squaramide (**SI-9**) (190 mg, 0.500 mmol), 1-bromobutane (0.32 mL, 3.0 mmol), Cs_2CO_3 (0.98 g, 3.0 mmol), and DMF (5 mL). The crude reaction mixture was purified via automated flash chromatography (40 g SiO_2, ethyl acetate in hexanes 0 to 50%) to yield a pale yellow solid (55 mg, 22% yield).

^{1}H NMR (400 MHz, $CDCl_3$) δ 7.58 (d, *J* = 8.7 Hz, 4H), 6.53 (d, *J* = 8.7 Hz, 4H), 4.12 (t, *J* = 7.6 Hz, 4H), 3.87 (s, 6H), 1.69 – 1.52 (m, 4H), 1.36 (dq, *J* = 14.6, 7.4 Hz, 4H), 0.90 (t, *J* = 7.3 Hz, 6H). ^{13}C NMR (101 MHz, $CDCl_3$) δ 186.8, 167.5, 166.0, 144.9, 130.2, 126.5, 121.5, 52.3, 52.2, 32.1, 19.8, 13.8. R_f (hexanes/ethyl acetate 2:1) = 0.30. HRMS (ESI) m/z calculated for $C_{28}H_{32}N_2O_6$ (H+): 493.2333, found: 493.2348.

diethyl 4,4'-((3,4-dioxocyclobut-1-ene-1,2-diyl)bis(azanediyl))dibenzoate (**SI-10**)

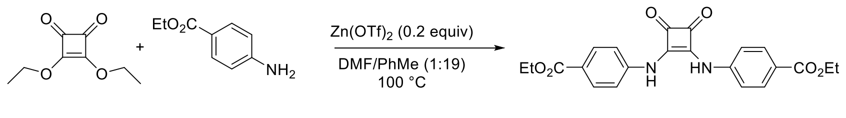

Synthesized according to general synthetic protocol C with diethyl squarate (0.60 mL, 4.0 mmol), ethyl 4-aminobenzoate (1.39 g, 8.40 mmol), zinc trifluoromethanesulfonate (290 mg, 0.80 mmol), DMF (0.4 mL), and toluene (7.6 mL). The product was afforded as an off-white solid (1.25 g, 77% crude yield) and used without further purification.

While the compound was not analytically pure and was carried forward crude, the mixture was characterized by ^{1}H NMR and this crude data is included below and the spectrum is also provided for reference in the appropriate section: ^{1}H NMR (400 MHz, DMSO-D_6) δ 10.24 (s, 2H), 7.96 (d, *J* = 8.8 Hz, 4H), 7.58 (d, *J* = 8.8 Hz, 4H), 4.30 (q, *J* = 7.1 Hz, 4H), 1.32 (t, *J* = 7.1 Hz, 6H).

diethyl 4,4'-((3,4-dioxocyclobut-1-ene-1,2-diyl)bis(butylazanediyl))dibenzoate (**SQA-7**)

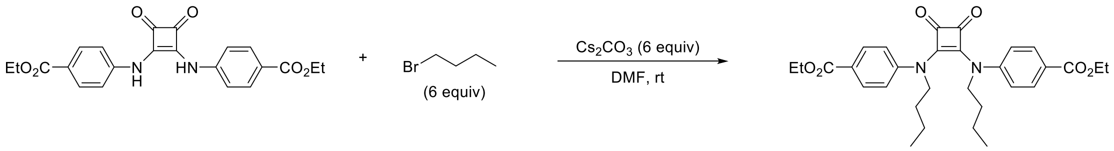

Synthesized according to general synthetic protocol D with squaramide (**SI-10**) (204 mg, 0.500 mmol), 1-bromobutane (0.32 mL, 3.0 mmol), Cs_2CO_3 (0.98 g, 3.0 mmol), and DMF (5 mL). The crude reaction mixture was purified via automated flash chromatography (24 g SiO_2, ethyl acetate in hexanes 0 to 20%) to yield a pale yellow solid (124 mg, 48% yield).

^{1}H NMR (500 MHz, $CDCl_3$) δ 7.58 (d, *J* = 8.4 Hz, 4H), 6.52 (d, *J* = 8.4 Hz, 4H), 4.32 (q, *J* = 7.1 Hz, 4H), 4.10 (t, *J* = 7.6 Hz, 4H), 1.60 (p, *J* = 7.7 Hz, 4H), 1.35 (t, *J* = 7.3 Hz, 6H), 1.34 (obscured m, 4 H) 0.88 (t, *J* = 7.4 Hz, 6H). ^{13}C NMR (126 MHz, $CDCl_3$) δ 186.7, 167.5, 165.5, 144.7, 130.1, 126.8, 121.6, 61.1, 52.1, 32.0, 19.8, 14.4, 13.8. HRMS (ESI) m/z calculated for $C_{30}H_{36}N_2O_6$ (H+): 521.2646, found: 521.2660.

di-tert-butyl 4,4'-((3,4-dioxocyclobut-1-ene-1,2-diyl)bis(azanediyl))dibenzoate (**SI-11**)

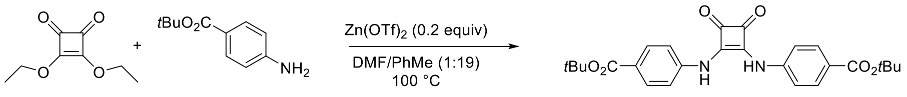

Synthesized according to general synthetic protocol C with diethyl squarate (0.15 mL, 0.99 mmol), *tert*-buytl 4-aminobenzoate (400. mg, 4.07 mmol), zinc trifluoromethanesulfonate (73 mg, 0.20 mmol), DMF (0.1 mL), and toluene (1.9 mL). The product was afforded as an off-white solid (417 mg, 91% crude yield) and used without further purification.

While the compound was not analytically pure and was carried forward crude, the mixture was characterized by ^{1}H NMR ^{13}C and this crude data is included below and the spectrum is also provided for reference in the appropriate section: ^{1}H NMR (500 MHz, DMSO-d_6) δ 10.19 (s, 2H), 7.91 (d, *J* = 8.7 Hz, 4H), 7.57 (d, *J* = 8.7 Hz, 4H), 1.54 (s, 18H). ^{13}C NMR (126 MHz, DMSO-d_6) δ 182.1, 165.9, 164.4, 142.3, 130.7, 125.7, 118.0, 80.4, 27.8.

di-tert-butyl 4,4'-((3,4-dioxocyclobut-1-ene-1,2-diyl)bis(butylazanediyl))dibenzoate (**SQA-8**)

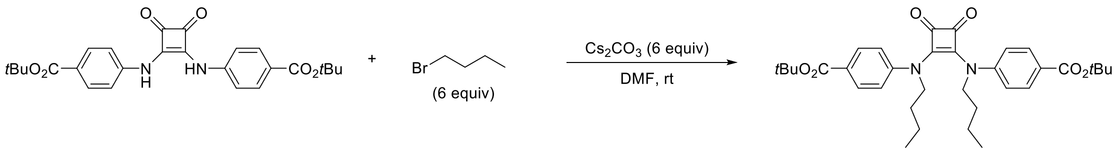

Synthesized according to general synthetic protocol D with squaramide (**SI-11**) (231 mg, 0.500 mmol), 1-bromobutane (0.32 mL, 3.0 mmol), Cs_2CO_3 (0.98 g, 3.0 mmol), and DMF (5 mL). The crude reaction mixture was purified via automated flash chromatography (40 g SiO_2, ethyl acetate in hexanes 0 to 35%) to yield a pale yellow solid (77 mg, 27% yield).

^{1}H NMR (400 MHz, $CDCl_3$) δ 7.56 (d, J = 8.5 Hz, 4H), 6.53 (d, J = 8.4 Hz, 4H), 4.13 (t, J = 7.6 Hz, 4H), 1.62-1.53 (m, 4H), 1.60 (s, 18H), 1.45 – 1.30 (m, 4H), 0.91 (t, J = 7.3 Hz, 6H). ^{13}C NMR (101 MHz, $CDCl_3$) δ 186.6, 167.4, 164.6, 144.4, 129.9, 128.4, 121.7, 81.2, 52.1, 32.0, 28.2, 19.7, 13.8. R_f (hexanes/ethyl acetate 3:1) = 0.34. HRMS (ESI) m/z calculated for $C_{34}H_{44}N_2O_6$ (H+): 577.3272, found: 577.3289.

3,4-bis((4-bromophenyl)amino)cyclobut-3-ene-1,2-dione (**SI-12**)

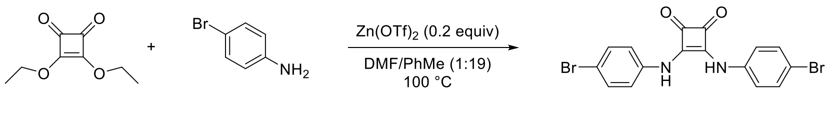

Synthesized according to general synthetic protocol C with diethyl squarate (0.60 mL, 4.0 mmol), 4-bromoaniline (1.43 g, 8.40 mmol), zinc trifluoromethanesulfonate (290 mg, 0.80 mmol), DMF (0.4 mL), and toluene (7.6 mL). The product was afforded as a solid (1.42 g, 85% crude yield) and used without further purification.

While the compound was not analytically pure and was carried forward crude, the mixture was characterized by ^{1}H NMR and this crude data is included below and the spectrum is also provided for reference in the appropriate section: ^{1}H NMR (400 MHz, DMSO-d_6) δ 9.96 (s, 2H), 7.56 (d, J = 8.9 Hz, 4H), 7.42 (d, J = 8.9 Hz, 4H). Data are consistent with those reported in the literature.[4]

3,4-bis((4-bromophenyl)(butyl)amino)cyclobut-3-ene-1,2-dione (**SQA-9**)

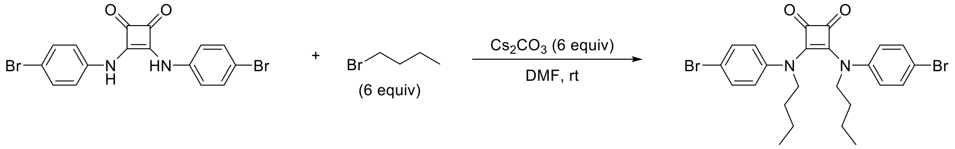

Synthesized according to general synthetic protocol D with squaramide (**SI-12**) (211 mg, 0.500 mmol), 1-bromobutane (0.32 mL, 3.0 mmol), Cs_2CO_3 (0.98 g, 3.0 mmol), and DMF (5 mL). The crude reaction mixture was purified via automated flash chromatography (24 g SiO_2, ethyl acetate in hexanes 0 to 35%) to yield a solid (136 mg, 51% yield).

^{1}H NMR (500 MHz, $CDCl_3$) δ 7.13 (d, *J* = 8.7 Hz, 2H), 6.37 (d, *J* = 8.7 Hz, 2H), 4.03 (t, *J* = 7.5 Hz, 2H), 1.61 – 1.51 (m, 2H), 1.33 (h, *J* = 7.4 Hz, 2H), 0.88 (t, *J* = 7.4 Hz, 3H). ^{13}C NMR (126 MHz, $CDCl_3$) δ 186.2, 167.3, 140.3, 131.7, 124.5, 118.9, 52.5, 32.0, 19.8, 13.9. R_f (hexanes/ethyl acetate 4:1) = 0.37. HRMS (ESI) m/z calculated for $C_{24}H_{26}Br_2N_2O_2$ (H+): 533.0434, found: 533.0433.

3,4-bis((4-chloro-2,6-difluorophenyl)amino)cyclobut-3-ene-1,2-dione (**SI-13**)

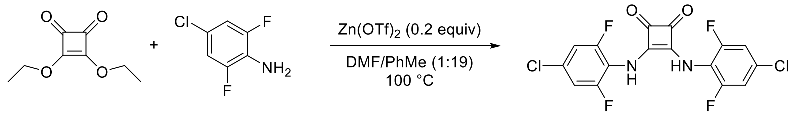

Synthesized according to general synthetic protocol C with diethyl squarate (0.60 mL, 4.0 mmol), 4-chloro-2,6-difluoroaniline (1.37 g, 8.40 mmol), zinc trifluoromethanesulfonate (290 mg, 0.80 mmol), DMF (0.4 mL), and toluene (7.6 mL). The product was afforded in approximately 70% purity as an off-white solid (510 mg, 32% crude yield) and used without further purification.

While the compound was not analytically pure and was carried forward crude, the mixture was characterized by ^{1}H NMR and this crude data is included below and the spectrum is also provided for reference in the appropriate section: ^{1}H NMR (400 MHz, DMSO-d_6) δ 9.86 (s, 2H), 7.40 (d, *J* = 7.7 Hz, 4H).

3,4-bis(butyl(4-chloro-2,6-difluorophenyl)amino)cyclobut-3-ene-1,2-dione (**SQA-10**)

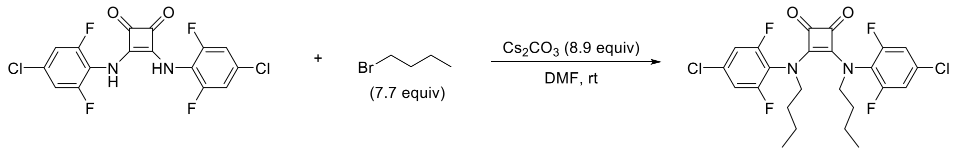

Synthesized according to general synthetic protocol D with squaramide (**SI-13**) (195 mg, 0.481 mmol), 1-bromobutane (0.4 mL, 3.7 mmol), Cs_2CO_3 (1.4 g, 4.3 mmol), and DMF (6 mL). The crude reaction mixture was purified via automated flash chromatography (24 g SiO_2, ethyl acetate in hexanes 0 to 40%) to yield an off-white solid (85 mg, 34% yield).

^{1}H NMR (500 MHz, $CDCl_3$) δ 6.78 (d, *J* = 7.7 Hz, 4H), 3.90 (t, *J* = 7.7 Hz, 4H), 1.46 (p, *J* = 7.6 Hz, 4H), 1.28 (h, *J* = 7.4 Hz, 4H), 0.85 (t, *J* = 7.3 Hz, 6H). ^{13}C NMR (126 MHz, $CDCl_3$) δ 185.0, 169.2, 157.1 (dd, *J* = 255.9, 5.8 Hz), 133.7 (t, *J* = 12.6 Hz), 118.3, 113.5 (dd, *J* = 21.7, 4.8 Hz), 53.0, 31.2, 19.5, 13.8. ^{19}F NMR (470 MHz, $CDCl_3$) δ -115.24 (d, $J_{F\text{-}H}$ = 8.4 Hz). R_f (hexanes/ethyl acetate 3:1) = 0.55. HRMS (ESI) m/z calculated for $C_{24}H_{22}Cl_2F_4N_2O_2$ (H+): 517.1067, found: 517.1077.

3,4-bis((4-nitrophenyl)amino)cyclobut-3-ene-1,2-dione (**SI-14**)

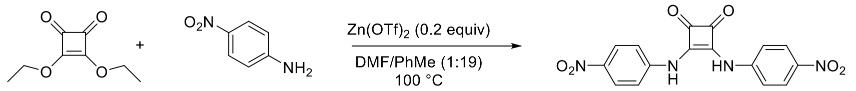

Synthesized according to general synthetic protocol C with diethyl squarate (0.60 mL, 4.0 mmol), 4-nitroaniline (1.16 g, 8.4 mmol), zinc trifluoromethanesulfonate (290 mg, 0.80 mmol), DMF (0.4 mL), and toluene (7.6 mL). The product was afforded an orange solid (1.27 g, 90% crude yield) and used without further purification.

While the compound was not analytically pure and was carried forward crude, the mixture was characterized by ^{1}H NMR and this crude data is included below and the spectrum is also provided for reference in the appropriate section: ^{1}H NMR (400 MHz, DMSO-d_6) δ 10.53 (s, 2H), 8.28 (d, J = 8.8 Hz, 4H), 7.65 (d, J = 8.8 Hz, 4H). Data are consistent with those reported in the literature.[5]

3,4-bis(butyl(4-nitrophenyl)amino)cyclobut-3-ene-1,2-dione (**SQA-11**)

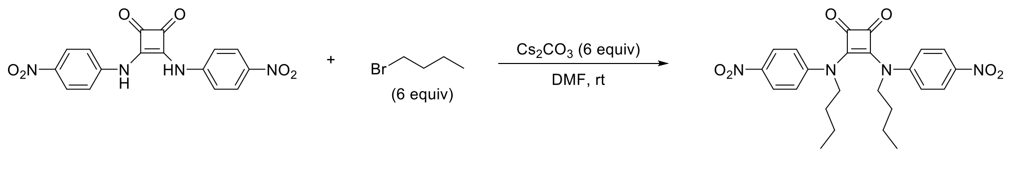

Synthesized according to general synthetic protocol D with squaramide (**SI-14**) (177 mg, 0.500 mmol), 1-bromobutane (0.32 mL, 3.0 mmol), Cs_2CO_3 (0.98 g, 3.0 mmol), and DMF (5 mL). The crude reaction mixture was purified via two sequential automated flash chromatography (40 g SiO_2, ethyl acetate in hexanes 0 to 35%) to yield an orange solid (18 mg, 8% yield).

^{1}H NMR (400 MHz, $CDCl_3$) δ 7.86 (d, J = 9.1 Hz, 4H), 6.66 (d, J = 9.1 Hz, 4H), 4.21 (t, J = 7.6 Hz, 4H), 1.77 – 1.63 (m, 4H), 1.42 (h, J = 7.4 Hz, 4H), 0.95 (t, J = 7.4 Hz, 6H). ^{13}C NMR (126 MHz, $CDCl_3$) δ 186.8, 167.5, 146.1, 144.1, 124.5, 121.6, 52.3, 32.2, 19.9, 13.8. R_f (hexanes/ethyl acetate 2:1) = 0.42. HRMS (ESI) m/z calculated for $C_{24}H_{26}N_4O_6$ (H+): 467.1925, found: 467.1922.

diethyl 4,4'-((3,4-dioxocyclobut-1-ene-1,2-diyl)bis(methylazanediyl))dibenzoate (**SQA-12**)

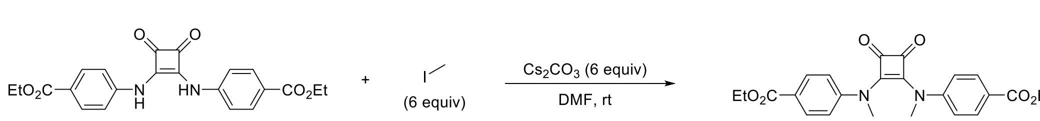

Synthesized according to general synthetic protocol D with squaramide (**SI-10**) (204 mg, 0.500 mmol), iodomethane (0.19 mL, 3.0 mmol), Cs_2CO_3 (0.98 g, 3.0 mmol), and DMF (5 mL). The crude reaction mixture was purified via automated flash chromatography (40 g SiO_2, ethyl acetate in 1:1 toluene:hexanes 0 to 40%) to yield a pale orange solid (65 mg, 30% yield).

^1H NMR (400 MHz, $CDCl_3$) δ 7.65 (d, *J* = 8.8 Hz, 4H), 6.65 (d, *J* = 8.7 Hz, 4H), 4.31 (q, *J* = 7.1 Hz, 4H), 3.71 (s, 6H), 1.35 (t, *J* = 7.1 Hz, 6H). ^{13}C NMR (101 MHz, $CDCl_3$) δ 187.1, 167.8, 165.5, 146.1, 130.4, 126.7, 120.0, 61.1, 38.5, 14.4. R_f (hexanes/ethyl acetate/toluene 1:1:1) = 0.28. HRMS (ESI) m/z calculated for $C_{24}H_{24}N_2O_6$ (H+): 437.1707, found: 437.1714.

<u>ethyl 4-(butyl(2-((4-(ethoxycarbonyl)phenyl)(isopropyl)amino)-3,4-dioxocyclobut-1-en-1-yl)amino)benzoate</u> (**SQA-13**)

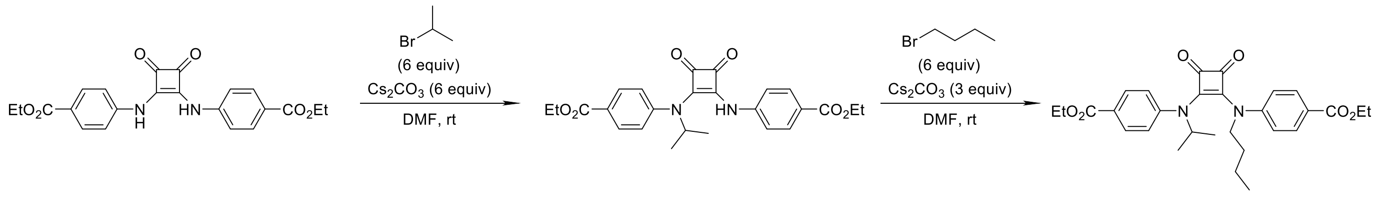

Monoalkylated product ethyl 4-((2-((4-(ethoxycarbonyl)phenyl)(isopropyl)amino)-3,4-dioxocyclobut-1-en-1-yl)amino)benzoate was synthesized according to general synthetic protocol D with squaramide (**SI-10**) (204 mg, 0.500 mmol), 2-bromopropane (0.28 mL, 3.0 mmol), Cs_2CO_3 (0.98 g, 3.0 mmol), and DMF (5 mL). The crude reaction mixture was purified via automated flash chromatography (24 g SiO_2, ethyl acetate in hexanes 0 to 50%) to yield the named product (90 mg, 40% yield) which was immediately carried forward to the second alkylation step.

R_f (hexanes/ethyl acetate 1:1) = 0.41.

The monoalkylated product was carried forward to a second alkylation that was run according to general synthetic protocol D with the monoalkyated squaramide (90 mg, 0.20 mmol), 1-bromobutane (0.13 mL, 1.2 mmol), Cs_2CO_3 (0.20 g, 0.60 mmol), and DMF (4 mL). The crude reaction mixture was purified via automated flash chromatography (24 g SiO_2, ethyl acetate in hexanes 0 to 35%) to yield the desired product as a white solid (58 mg, 57% yield).

^1H NMR (500 MHz, $CDCl_3$) δ 7.62 (d, *J* = 8.6 Hz, 2H), 7.53 (d, *J* = 8.6 Hz, 2H), 6.45 (d, *J* = 8.6 Hz, 2H), 6.42 (d, *J* = 8.7 Hz, 2H), 4.81 (p, *J* = 6.8 Hz, 1H), 4.41 – 4.29 (m, 4H), 4.00 (t, *J* = 7.6 Hz, 2H), 1.58 – 1.47 (m, 2H), 1.43 – 1.26 (m, 14H), 0.86 (t, *J* = 7.4 Hz, 3H). ^{13}C NMR (126 MHz, $CDCl_3$) δ 186.7, 186.5, 169.0, 168.1, 165.8, 165.6, 144.8, 142.8, 130.2, 129.6, 127.8, 126.9, 125.1, 121.8, 61.3, 61.3, 55.5, 52.4, 32.1, 22.2, 19.8, 14.5, 14.5, 13.9. R_f (hexanes/ethyl acetate 3:1) = 0.28. HRMS (ESI) m/z calculated for $C_{29}H_{34}N_2O_6$ (H+): 507.2490, found: 507.2497.

diethyl 4,4'-(7,8-dioxo-2,5-diazabicyclo[4.2.0]oct-1(6)-ene-2,5-diyl)dibenzoate (**SQA-14**)

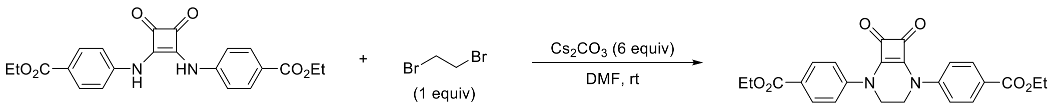

Synthesized according to a slight modification of general synthetic protocol D (equivalents of electrophile have been modified) with squaramide (**SI-10**) (204 mg, 0.500 mmol), 1,2-dibromoethane (0.13 mL, 0.50 mmol), Cs_2CO_3 (0.98 g, 3.0 mmol), and DMF (5 mL). The crude reaction mixture was purified via sequential automated flash chromatography (24 g SiO_2, loaded with dichloromethane, ethyl acetate in hexanes 30 to 90% then 24 g SiO_2, ethyl acetate in dichloromethane 0 to 20%) to yield an off-white solid (71 mg, 33% yield).

R_f (hexanes/ethyl acetate 1:3) = 0.73. ^{1}H NMR (400 MHz, $CDCl_3$) δ 8.11 (d, J = 8.8 Hz, 3H), 7.46 (d, J = 8.9 Hz, 3H), 4.36 (q, J = 7.1 Hz, 4H), 4.11 (s, 4H), 1.38 (t, J = 7.1 Hz, 6H). ^{13}C NMR (101 MHz, $CDCl_3$) δ 180.1, 165.9, 165.7, 143.5, 131.5, 126.8, 117.6, 61.2, 45.3, 14.5. HRMS (ESI) m/z calculated for $C_{24}H_{22}N_2O_6$ (H+): 435.1551, found: 467.1922.

3,4-bis((2-(trifluoromethyl)phenyl)amino)cyclobut-3-ene-1,2-dione (**SI-15**)

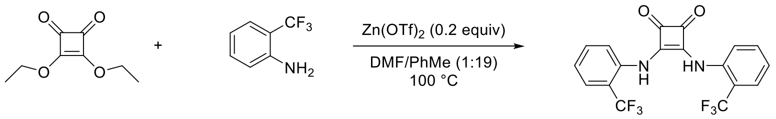

Synthesized according to general synthetic protocol C with diethyl squarate (0.60 mL, 4.0 mmol), 2-(trifluoromethyl)aniline (1.06 mL, 8.40 mmol), zinc trifluoromethanesulfonate (290 mg, 0.80 mmol), DMF (0.4 mL), and toluene (7.6 mL). The following change was made to the workup procedure for this compound due to its relatively high solubility in methanol and DMF: At the completion of the reaction, volatiles were removed under reduced pressure to yield an approximately 3:2 ratio of the desired product and the product that results from monoaddition. This crude reaction mixture was purified via careful automated flash chromatography (40 g SiO_2, ethyl acetate in hexanes 0 to 45%) to afford the product as a white solid (813 mg, 51% yield).

R_f (hexanes/ethyl acetate 1:1) = 0.6. ^{1}H NMR (400 MHz, DMSO-d_6) δ 11.01 (s, 2H), 7.74 (d, J = 7.8 Hz, 2H), 7.68 (t, J = 7.7 Hz, 2H), 7.53 (d, J = 8.0 Hz, 2H), 7.46 (t, J = 7.7 Hz, 2H).

2,5-bis(2-(trifluoromethyl)phenyl)-2,5-diazabicyclo[4.2.0]oct-1(6)-ene-7,8-dione (**SQA-15**)

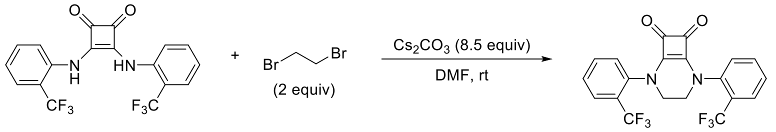

Synthesized according to a slight modification of general synthetic protocol D (equivalents have been modified) with squaramide (**SI-15**) (140 mg, 0.35 mmol), 1,2-dibromoethane (0.060 mL, 0.69 mmol), Cs_2CO_3 (0.98 g, 3.0 mmol), and DMF (5 mL). The crude reaction mixture was purified via sequential automated flash chromatography (24 g SiO_2, ethyl acetate in hexanes 0 to 70%) to yield an off-white solid (78 mg, 52% yield).

^{1}H NMR (500 MHz, CD_3CN) δ 7.86 (dd, J = 7.8, 1.5 Hz, 2H), 7.78 (td, J = 7.7, 1.5 Hz, 2H), 7.66 – 7.60 (m, 4H), 3.90 (s, 4H). ^{13}C NMR (126 MHz, CD_3CN) δ 182.42, 169.54, 140.30 (q, $J_{C\text{-}F}$ = 1.8 Hz), 134.95, 130.66, 130.22, 128.51 (q, $J_{C\text{-}F}$ = 5.1 Hz), 127.64 (q, $J_{C\text{-}F}$ = 30.4 Hz), 124.62 (q, $J_{C\text{-}F}$ = 272.6 Hz), 51.76 (q, $J_{C\text{-}F}$ = 1.8 Hz). ^{19}F NMR (470 MHz, CD_3CN) δ -60.79. R_f (hexanes/ethyl acetate 1:2) = 0.32. HRMS (ESI) m/z calculated for $C_{20}H_{12}F_6N_2O_2$ (H+): 427.0876, found: 427.0890.

3-ethoxy-4-((4-methoxyphenyl)amino)cyclobut-3-ene-1,2-dione (**SI-16**)

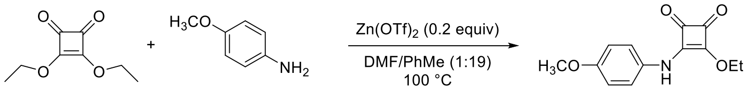

An oven- or flamed-dried round-bottom flask equipped with a magnetic stir bar was charged with the 4-methoxy aniline (493 mg, 0.400 mmol) and zinc trifluoromethanesulfonate (290 mg, 0.800 mmol) and then placed under a nitrogen atmosphere. DMF (0.4 mL) and toluene (7.6 mL) was then added followed by a rapid addition of diethyl squarate via syringe (0.60 mL, 0.40 mmol) with strong stirring. The resulting reaction mixture was heated to 100 °C (oil bath temperature) for 16 h overnight. At this point, the rection mixture was cooled to room temperature and the resulting solid material was filtered and washed with a small amount of toluene then water before being dried under high vacuum. The crude reaction mixture was purified via automated flash chromatography (40 g SiO_2, dry loaded in celite, ethyl acetate in dichloromethane 0 to 30%) to yield a yellow solid (508 mg, 53% yield).

^{1}H NMR (600 MHz, $CDCl_3$) δ 8.26 (s, 1H), 7.24 (obscured d, 2H), 6.92 – 6.87 (m, 2H), 4.87 (q, J = 7.1 Hz, 2H), 3.81 (s, 3H), 1.50 (t, J = 7.1 Hz, 3H). ^{13}C NMR (151 MHz, $CDCl_3$) δ 189.8, 183.8, 177.1, 167.9, 157.4, 129.1, 122.8, 115.3, 71.4, 54.1, 13.9. Data are consistent with those reported in the literature.[6]

3-(butyl(4-methoxyphenyl)amino)-4-ethoxycyclobut-3-ene-1,2-dione (**SQA-16**)

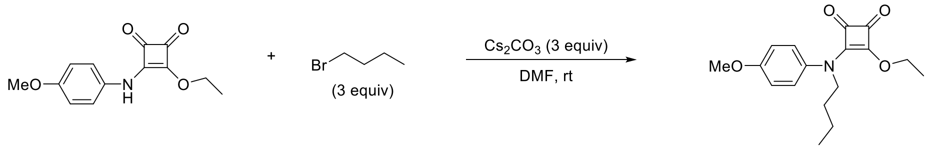

Synthesized according to a slightly modified general synthetic protocol D with squaramide (**SI-16**) (124 mg, 0.500 mmol), 1-bromobutane (0.16 mL, 1.5 mmol), Cs_2CO_3 (0.49 g, 1.5 mmol), and DMF (5 mL). The crude reaction mixture was purified via automated flash chromatography (40 g SiO_2, ethyl acetate in hexanes 0 to 45%) to yield a solid (109 mg, 24% yield).

Characterization of the product is consistent with a mixture of conformational isomers being present that are slow to interconvert within the NMR time-scale at rt. ^{1}H NMR (500 MHz, $CDCl_3$) δ 7.07 (d, *J* = 8.3 Hz, 2H), 6.95 – 6.80 (m, 2H), 4.87 – 4.79 (m) and 4.60 (q, *J* = 7.1 Hz) (2H combined total), 4.09 (t, *J* = 7.3 Hz) and obscured peak at 3.80 (m) (2H combined total), 3.82 (s, 3H), 1.51 (dt, *J* = 16.3, 7.7 Hz, 3H), 1.38 – 1.22 (m, 4H), 0.95 – 0.84 (m, 3H). ^{13}C NMR (126 MHz, $CDCl_3$) δ 188.7, 186.9, 183.9, 177.2, 176.8, 171.8, 171.2, 158.7, 132.7, 126.3, 126.0, 114.2, 114.0, 69.8, 69.4, 55.5, 53.1, 52.1, 30.7, 19.4, 15.9, 15.7, 13.7. R_f (hexanes/ethyl acetate 1.5:1) = 0.44. HRMS (ESI) m/z calculated for $C_{17}H_{21}NO_4$ (H+): 304.1543, found: 304.1553.

3,4-bis(dibutylamino)cyclobut-3-ene-1,2-dione (**SQA-17**)

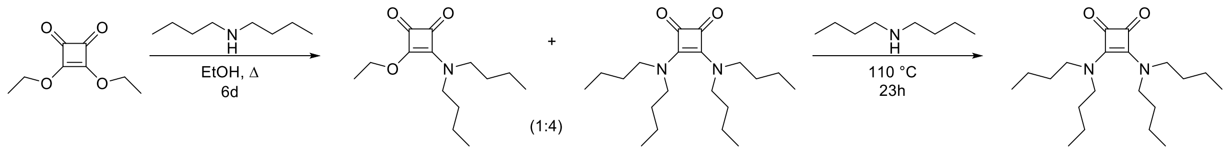

An oven-dried 100 mL three-neck round-bottom flask equipped with a magnetic stir bar and reflux condenser was placed under a nitrogen atmosphere and charged with ethanol (20 mL), diethyl squarate (0.30 mL, 2.0 mmol), and *N,N*-di-*n*-butylamine (2.0 mL, 12 mmol). The reaction mixture was heated to reflux (oil bath temperature 90 °C) and stirred for 6 days. At this point, the volatiles were removed under reduced pressure to reveal a 1:4 ratio of the mono- and di-addition products. This crude reaction mixture was transferred into a 50 mL round-bottom flask equipped with a magnetic stir bar, placed under a nitrogen atmosphere, and charged with *N,N*-di-*n*-butylamine (10 mL, 59 mmol). The reaction mixture was then stirred at 110 °C (oil bath temperature) for 23 h. Voltaile materials were removed under reduced pressure and the crude reaction mixture was purified via automated flash chromatography (24 g SiO_2, 1:1 hexane:chloroform in ethyl acetate 0 to 15%) to yield a clear colorless oil (248 mg, 37% yield).

R_f (hexanes/chloroform/ethyl acetate 1:1:0.2) = 0.31. ^{1}H NMR (400 MHz, $CDCl_3$) δ 3.47 (t, *J* = 7.4 Hz, 4H), 1.56 (p, *J* = 7.5 Hz, 4H), 1.30 (h, *J* = 7.3 Hz, 4H), 0.91 (t, *J* = 7.3 Hz, 6H). ^{13}C NMR (101 MHz, $CDCl_3$) δ 185.1, 170.4, 50.0, 30.8, 19.9, 13.8. HRMS (ESI) calculated for $C_{20}H_{36}N_2O_2$ (H+): 337.2850, found: 337.2853.

bis(2-(2-methoxyethoxy)ethyl) 4,4'-((3,4-dioxocyclobut-1-ene-1,2-diyl)bis(azanediyl))dibenzoate (**SI-17**)

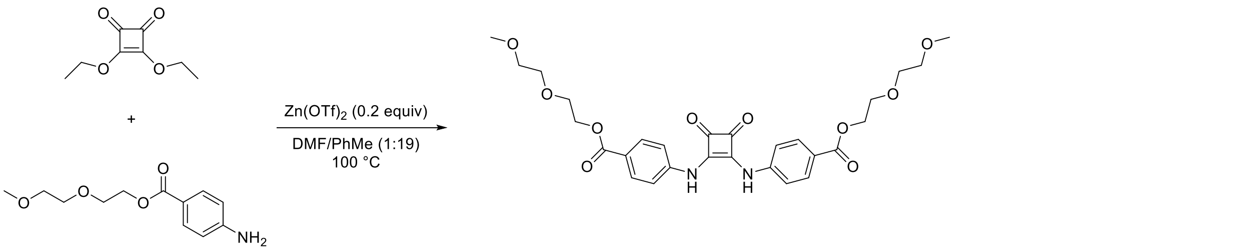

Synthesized according to a modified version of general synthetic protocol C with diethyl squarate (1.42 mL, 8.57 mmol), 2-(2-methoxyethoxy)ethyl 4-aminobenzoate (4.31 g, 18.0 mmol), zinc trifluoromethanesulfonate (620. mg, 1.71 mmol), DMF (0.9 mL), and toluene (16 mL). In a deviation from the standard protocol, after cooling to room temperature, the reaction mixture was diluted with DCM (500 mL) and extracted with water (100 mL) and then washed with brine (100 mL). The organic layer was then dried ($MgSO_4$), filtered, and concentrated under reduced pressure to afford a waxy yellow solid (4.89 g, approximately quantitative crude yield) and used without further purification.

While the compound was not analytically pure and was carried forward crude, the mixture was characterized by ^{1}H NMR and this crude data is included below and the spectrum is also provided for reference in the appropriate section: ^{1}H NMR (400 MHz, DMSO-d_6) δ 10.50 (s, 2H), 7.91 (d, *J* = 8.8 Hz, 4H), 7.60 (d, *J* = 8.6 Hz, 4H), 4.37 – 4.31 (m, 4H), 3.75 – 3.70 (m, 4H), 3.59 (dd, *J* = 5.8, 3.6 Hz, 4H), 3.45 (dd, *J* = 5.8, 3.7 Hz, 4H), 3.23 (s, 6H).

bis(2-(2-methoxyethoxy)ethyl) 4,4'-((3,4-dioxocyclobut-1-ene-1,2-diyl)bis(methylazanediyl))dibenzoate (**SQA-1**)

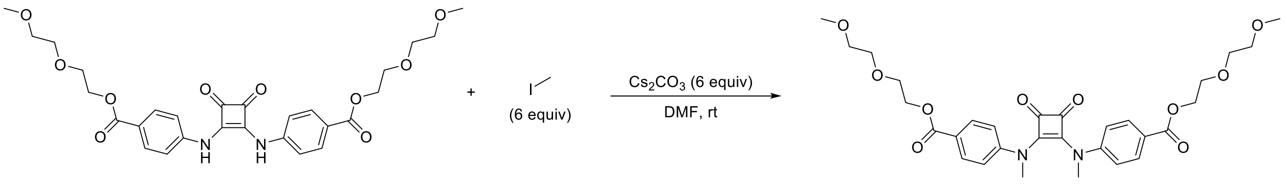

Synthesized according to general synthetic protocol D with squaramide (**SI-17**) (4.34 g, 7.80 mmol), iodomethane (2.91 mL, 46.8 mmol), Cs_2CO_3 (15.2 g, 46.8 mmol), and DMF (78 mL). The crude reaction mixture was purified via automated flash chromatography (two identical columns were required: 280 g SiO_2, methanol in ethyl acetate 0 to 3%) to yield an orange solid (702 mg, 30% yield).

^{1}H NMR (400 MHz, $CDCl_3$) δ 7.68 (d, *J* = 8.7 Hz, 3H), 6.66 (d, *J* = 8.7 Hz, 4H), 4.45 – 4.35 (m, 4H), 3.82 – 3.75 (m, 4H), 3.68 (s, 6H), 3.67 – 3.63 (m, 4H), 3.57 – 3.51 (m, 4H), 3.35 (s, 6H). ^{13}C NMR (126 MHz, $CDCl_3$) δ 187.1, 167.9, 165.4, 146.1, 130.6, 126.3, 119.9, 72.0, 70.6, 69.3, 64.2, 59.1, 38.5. R_f (100% ethyl acetate) = 0.31. HRMS (ESI) m/z calculated for $C_{30}H_{36}N_2O_{10}$ (H+): 585.2443, found: 585.2452.

Bis(2-(2-methoxyethoxy)ethyl) 1,1'-(3,4-dioxocyclobut-1-ene-1,2-diyl)bis(indoline-5-carboxylate) (**SQA-18**)

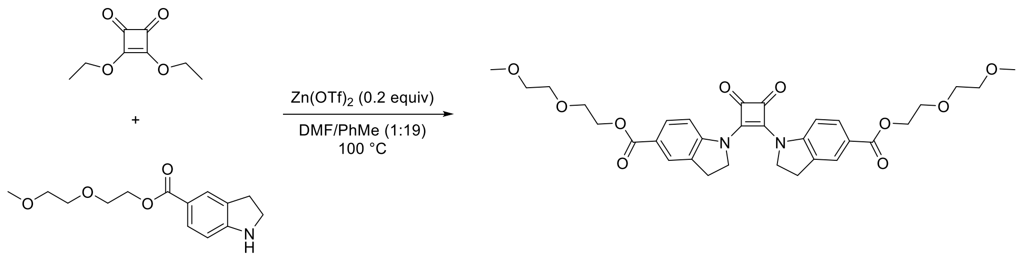

Synthesized according to general synthetic protocol C with diethyl squarate (0.059 mL, 0.40 mmol), 2-(2-methoxyethoxy)ethyl indoline-5-carboxylate (222 mg, 0.837 mmol), zinc trifluoromethanesulfonate (29 mg, 0.080 mmol), DMF (0.05 mL), and toluene (0.95 mL). In a deviation from the standard protocol, after cooling to room temperature, volatiles were removed under reduced pressure and the crude reaction mixture was purified via automated flash chromatography (24 g SiO_2, methanol in ethyl acetate 0 to 10%) to yield an orange solid (92 g, 38% crude yield).

^{1}H NMR (500 MHz, $CDCl_3$) δ 7.91 (d, J = 1.6 Hz, 2H), 7.81 (dd, J = 8.5, 1.7 Hz, 2H), 7.09 (d, J = 8.4 Hz, 2H), 4.43 (dd, J = 5.5, 4.1 Hz, 4H), 4.35 (t, J = 8.3 Hz, 4H), 3.83 – 3.78 (m, 4H), 3.69 – 3.65 (m, 4H), 3.59 – 3.53 (m, 4H), 3.37 (s, 6H), 3.28 (t, J = 8.2 Hz, 4H). ^{13}C NMR (126 MHz, $CDCl_3$) δ 186.0, 166.4, 166.0, 147.7, 131.2, 130.6, 127.2, 125.5, 113.0, 72.0, 70.7, 69.4, 64.2, 59.2, 52.8, 28.6. R_f (10% methanol in ethyl acetate) = 0.50. HRMS (ESI) m/z calculated for $C_{32}H_{36}N_2O_{10}$ (H+): 609.2443, found: 609.2439.

3,4-bis((4-(methylthio)phenyl)amino)cyclobut-3-ene-1,2-dione (**SI-18**)

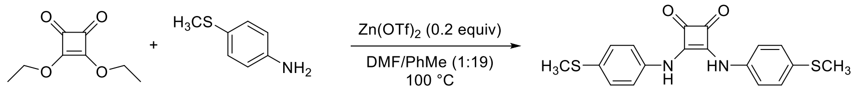

Synthesized according to general synthetic protocol C with diethyl squarate (0.60 mL, 4.0 mmol), 4-(methylthio)aniline (1.05 mL, 8.40 mmol), zinc trifluoromethanesulfonate (290 mg, 0.80 mmol), DMF (0.4 mL), and toluene (7.6 mL). The product was afforded as a yellow/orange solid (1.49 g, 84% crude yield) and used without further purification.

While the compound was not analytically pure and was carried forward crude, the mixture was characterized by ^{1}H NMR and this crude data is included below and the spectrum is also provided for reference in the appropriate section: ^{1}H NMR (500 MHz, DMSO-d_6) δ 9.85 (s, 2H), 7.44 (d, J = 8.3 Hz, 4H), 7.30 (d, J = 8.3 Hz, 4H), 2.47 (s, 6H).

3,4-bis(butyl(4-(methylthio)phenyl)amino)cyclobut-3-ene-1,2-dione (**SQA-19**)

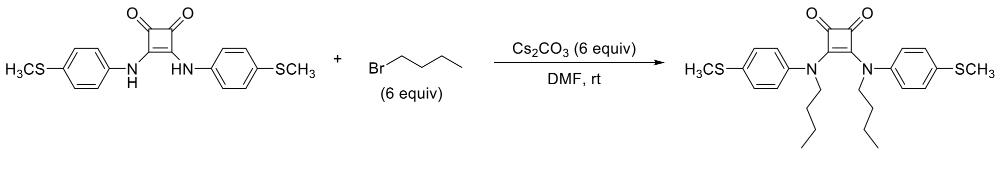

Synthesized according to general synthetic protocol D with squaramide (**SI-18**) (178 mg, 0.500 mmol), 1-bromobutane (0.32 mL, 3.0 mmol), Cs_2CO_3 (0.98 g, 3.0 mmol), and DMF (5 mL). In a deviation from the general protocol, the reaction mixture was allowed to stir at rt for 38 h. The crude reaction mixture was purified via automated flash chromatography (40 g SiO_2, ethyl acetate in hexanes 0 to 40%) to yield a pale-yellow solid (95 mg, 41% yield).

^{1}H NMR (400 MHz, $CDCl_3$) δ 6.83 (d, *J* = 8.3 Hz, 4H), 6.40 (dd, *J* = 8.1, 1.3 Hz, 4H), 4.01 (t, *J* = 7.5 Hz, 4H), 2.43 (d, *J* = 1.0 Hz, 6H), 1.60 – 1.46 (m, 4H), 1.32 (h, *J* = 7.4 Hz, 4H), 0.86 (t, *J* = 7.3 Hz, 6H). ^{13}C NMR (101 MHz, $CDCl_3$) δ 186.1, 167.2, 138.7, 135.6, 126.7, 123.4, 52.5, 31.9, 19.7, 16.1, 13.9. R_f (hexanes/ethyl acetate 5:1) = 0.12. HRMS (ESI) m/z calculated for $C_{26}H_{32}N_2O_2S_2$ (H+): 469.1978, found: 469.1994.

3,4-bis((4-methoxyphenyl)amino)cyclobut-3-ene-1,2-dione (**SI-19**)

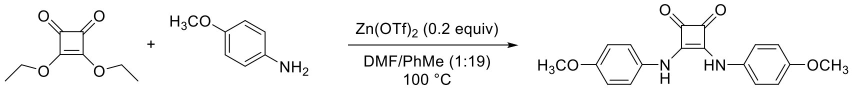

Synthesized according to general synthetic protocol C with diethyl squarate (0.60 mL, 4.0 mmol), 4-(methoxy)aniline (1.0 g, 8.4 mmol), zinc trifluoromethanesulfonate (290 mg, 0.80 mmol), DMF (0.4 mL), and toluene (7.6 mL). The product was afforded as a solid (977 mg, 75% crude yield) and used without further purification.

While the compound was not analytically pure and was carried forward crude, the mixture was characterized by ^{1}H NMR and this crude data is included below and the spectrum is also provided for reference in the appropriate section: ^{1}H NMR (400 MHz, DMSO-d_6) δ 9.68 (s, 2H), 7.40 (d, *J* = 9.0 Hz, 4H), 6.95 (d, *J* = 9.0 Hz, 4H), 3.74 (s, 6H). Data are consistent with those reported in the literature.[4]

3,4-bis(butyl(4-methoxyphenyl)amino)cyclobut-3-ene-1,2-dione (**SQA-2**)

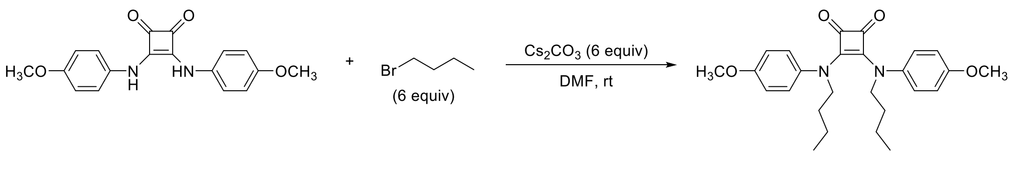

Synthesized according to general synthetic protocol D with squaramide (**SI-19**) (162 mg, 0.500 mmol), 1-bromobutane (0.32 mL, 3.0 mmol), Cs_2CO_3 (0.98 g, 3.0 mmol), and DMF (5 mL). The crude reaction

mixture was purified via automated flash chromatography (40 g SiO_2, ethyl acetate in hexanes 0 to 45%) to yield a pale-orange solid (109 mg, 50% yield).

^{1}H NMR (500 MHz, $CDCl_3$) δ 6.49 (d, *J* = 9.0 Hz, 4H), 6.43 (d, *J* = 8.9 Hz, 4H), 3.97 (t, *J* = 7.4 Hz, 4H), 3.73 (s, 6H), 1.54 – 1.46 (m, 4H), 1.31 (h, *J* = 7.4 Hz, 4H), 0.86 (t, *J* = 7.4 Hz, 6H). ^{13}C NMR (126 MHz, $CDCl_3$) δ 185.8, 167.5, 157.2, 135.0, 124.3, 113.9, 55.6, 53.0, 31.8, 19.7, 13.9. R_f (hexanes/ethyl acetate 2:1) = 0.22. HRMS (ESI) m/z calculated for $C_{26}H_{32}N_2O_4$ (H+): 437.2435, found: 437.2447.

<u>3,4-bis((4-isopropoxyphenyl)amino)cyclobut-3-ene-1,2-dione</u> (**SI-20**)

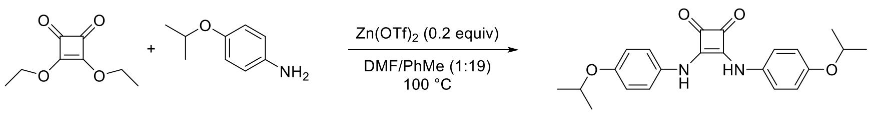

Synthesized according to general synthetic protocol C with diethyl squarate (0.60 mL, 4.0 mmol), 4-(isopropoxy)aniline (1.23 g, 8.40 mmol), zinc trifluoromethanesulfonate (290 mg, 0.80 mmol), DMF (0.4 mL), and toluene (7.6 mL). The product was afforded as an off-white solid (1.29 g, 85% crude yield) and used without further purification.

While the compound was not analytically pure and was carried forward crude, the mixture was characterized by ^{1}H NMR and this crude data is included below and the spectrum is also provided for reference in the appropriate section: ^{1}H NMR (400 MHz, DMSO-d_6) δ 9.68 (s, 2H), 7.37 (d, *J* = 8.9 Hz, 4H), 6.92 (d, *J* = 9.0 Hz, 4H), 4.56 (hept, *J* = 6.0 Hz, 2H), 1.25 (d, *J* = 6.0 Hz, 12H).

<u>3,4-bis(butyl(4-isopropoxyphenyl)amino)cyclobut-3-ene-1,2-dione</u> (**SQA-20**)

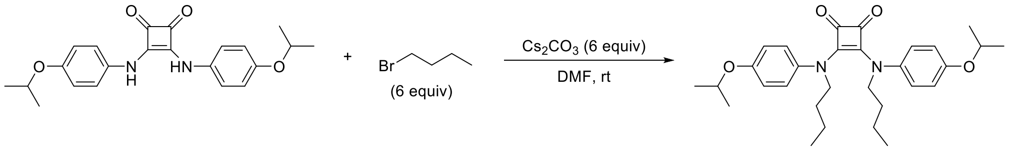

Synthesized according to general synthetic protocol D with squaramide (**SI-20**) (190 mg, 0.500 mmol), 1-bromobutane (0.32 mL, 3.0 mmol), Cs_2CO_3 (0.98 g, 3.0 mmol), and DMF (5 mL). The crude reaction mixture was purified via automated flash chromatography (55 g SiO_2, ethyl acetate in hexanes 0 to 35%) to yield a white solid (122 mg, 50% yield).

^{1}H NMR (500 MHz, $CDCl_3$) δ 6.48 – 6.44 (m, 4H), 6.44 – 6.39 (m, 4H), 4.40 (hept, *J* = 6.0 Hz, 2H), 3.97 (t, *J* = 7.4 Hz, 4H), 1.58 – 1.46 (m, 4H), 1.36-1.27 (m, 4H) 1.32 (d, *J* = 6.0 Hz, 12H), 0.87 (t, *J* = 7.3 Hz, 6H). ^{13}C NMR (126 MHz, $CDCl_3$) δ 185.8, 167.4, 155.4, 134.7, 124.1, 115.5, 70.1, 53.0, 31.9, 22.1, 19.8, 13.9. R_f (hexanes/ethyl acetate 2:1) = 0.50. HRMS (ESI) m/z calculated for $C_{30}H_{40}N_2O_4$ (H+): 493.3061, found: 493.3077.

Solubility measurements:

1) 3,8-bis(2-(2-methoxyethoxy)ethyl)-5-(trifluoromethyl)-3,8-dihydrocyclobuta[b]quinoxaline-1,2-dione (**SQX-1**)

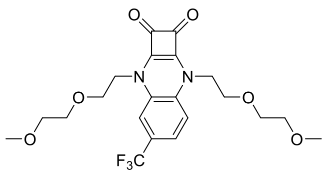

Solubility measurements in neat acetonitrile were conducted based upon a modified version of the published method of Sanford and coworkers.[7] The title compound (540 mg) was added to a 2-dram vial with a magnetic stir and dry MeCN was added in 10 µL aliquots until the material in the bottom of the vial is flowable and homogenous. This solution was sealed and stirred overnight with a magnetic stir bar. At that point, stirring was stopped, and two 20 µL aliquots were removed using a Hamilton 800 Series Microliter Syringe and dispensed into pre-tared 1-dram vials and dried under high vacuum overnight. The resulting mass increase in the vials was converted into moles, divided by the volume dispensed, and averaged over two experiments.

1st aliquot – 20.8 mg in 20.0 µL equals 2.27 M

2nd aliquot – 20.1 mg in 20 µL equals 2.19 M

Average solubility – 2.23 ± 0.06 M

Solubility measurements in 500 mM $TBAPF_6$ in acetonitrile were conducted by generating a UV-vis calibration curve of the **SQX-1** compound, as shown below.

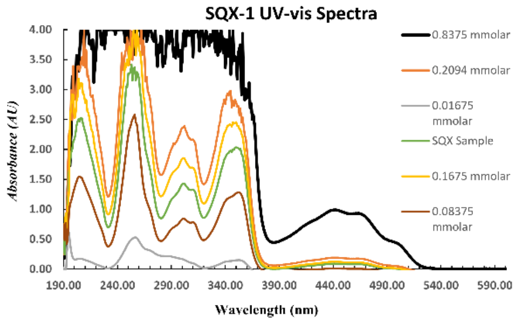

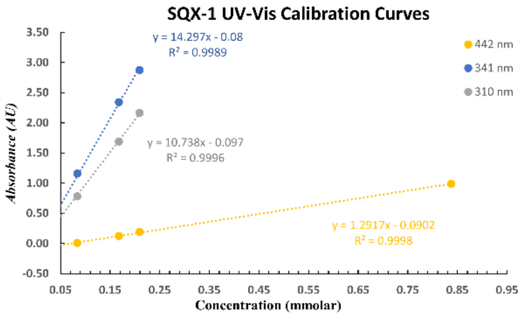

A saturated solution of **SQX-1** was prepared by adding 62uL of 500 mM $TBAPF_6$ in MeCN solution ("electrolyte solution") to 85.3 mg of pure **SQX-1** in a nitrogen-filled glovebox until a free-flowing liquid was observed. This solution was allowed to equilibrate for three days, and no solid remained in solution after equilibration. A 10uL aliquot of this saturated solution was then diluted with 900uL of electrolyte solution, and this 910mL solution ("solution 1") was removed from the glovebox. 100uL of solution 1 was diluted with 900uL of electrolyte solution to form the sample solution. This sample displayed a 442 nm absorbance of 0.0907, a 341 nm absorbance of 1.904, and a 310 nm absorbance of 1.3304. Solving for x in the calibration curve equations, the 442 absorbance yielded 0.140 mM, the 271 gave 0.139 mM, and 310 nm gave 0.133 mM. This averaged to 0.137 mM. With a 910x dilution yields a saturated concentration of **1.249 M**.

2) bis(2-(2-methoxyethoxy)ethyl) 4,4'-((3,4-dioxocyclobut-1-ene-1,2 diyl)bis(methylazanediyl))dibenzoate (**SQA-1**)

Solubility measurements in 500 mM $TBAPF_6$ in acetonitrile were conducted by generating a UV-vis calibration curve of the **SQA-1** compound, as shown below.

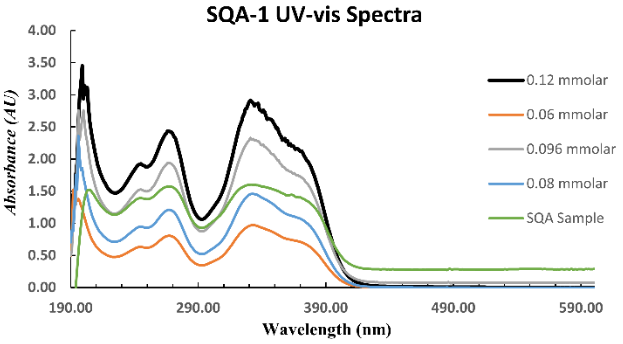

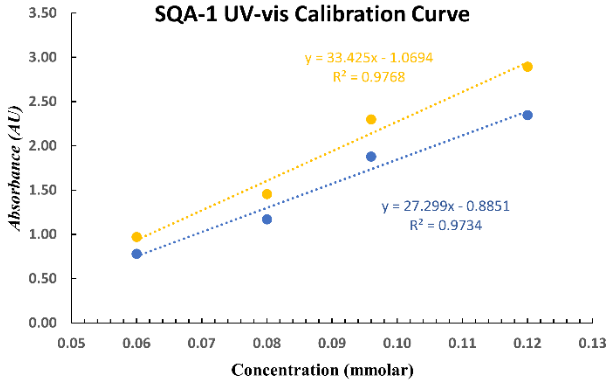

A saturated solution of SQA-1 was prepared by adding 200uL of 500 mM $TBAPF_6$ in MeCN solution ("electrolyte solution") to 244.4 mg of pure **SQA-1** in a nitrogen-filled glovebox. This solution was allowed to equilibrate overnight. It was then heated to 47° C for 25 minutes to increase dissolution, and left to cool again overnight. Substantial remaining solid SQA-1 was observed in the solution. A 10uL aliquot of this saturated solution was then diluted with 900uL of electrolyte solution, and this

910mL solution ("solution 1") was removed from the glovebox. 100uL of solution 1 was diluted with 900uL of electrolyte solution to form solution 2. 100uL of solution 2 was then diluted with 900uL of electrolyte solution to yield the sample solution. This sample displayed an absorbance at 332 nm of 1.602, and at 271 nm of 1.549. Solving for x in the calibration curve equations, the 332 absorbance yielded 0.0979 mM and the 271 0.0892 mM, averaging 0.0935 mM. This 9100x dilution yields a saturated concentration of **0.85095 M**. It is possible that the kinetic barrier to solvation was not fully overcome by mild heating, so this should be regarded as a saturation minimum value.

Electrochemical Investigations:

General methods and materials: Acetonitrile (MeCN) (99.9%, extra dry over molecular sieves) was obtained from Thermo Scientific. Tetrabutylammonium hexafluorophosphate (TBAPF6; >99%, for electrochemical analysis) was obtained from MilliporeSigma™, dried under high vacuum for 48 h at 75 °C and transferred to a N_2-filled glovebox for storage and use. All electrochemical experiments were performed in a N_2 filled glove box with an atmosphere <0.1 ppm oxygen and <0.1 ppm water. Electrolyte solutions were prepared in the glovebox by first drying the acetonitrile over freshly activated 3 Å molecular sieves for at least 24 h. Supporting electrolyte was then added and the solvent/electrolyte mixture was further dried for another 24 h before use. The resulting solvent/electrolyte mixtures were stored over the 3 Å molecular sieves in the glovebox. All potentials are reported relative to the ferrocene/ferrocenium couple (Fc/Fc^+) and this adjustment is made for each sample through the addition of a ferrocene reference at the end of each set of CV experiments.

Cyclic voltammetry: Cyclic voltammetry (CV) experiments were performed with a CH Instruments 760 Bipotentiostat with a three-electrode electrochemical cell. A glassy carbon disk electrode (BASi, 3.0 mm diameter) was used as a working electrode, a Ag/Ag^+ electrode (5 mM $AgBF_4$ in 0.5 M $TBAPF_6$ in MeCN) sealed with a Coralpor frit was used as a non-aqueous quasi-reference electrode (BASi), and platinum mesh was used as a counter electrode. The glassy carbon electrode was polished outside the glovebox using alumina (MicroPolish II, Buehler) in Milli-Q® water before being dried with acetone and brought into the glovebox. Unless otherwise indicated, all CV measurements were performed by dissolving the compound in stock 0.5 M $TBAPF_6$ in acetonitrile to give a concentration of 5 mM.

H-cell cycling: Bulk charge/discharge measurements were carried out in a nitrogen-filled glovebox with a CH Instruments 760 Bipotentiostat in a custom H-cell (pictured below) with a fritted glass separator (P5). The working and counter electrodes were carbon (Duocel® RVC Foam, 100 PPI, 3% relative density). An Ag/Ag^+ quasi-reference electrode (described above) was used on the working side of the H-cell. The active compound was dissolved in 0.5 M $TBAPF_6$ in acetonitrile to give a redox active material concentration of 5 mM. The working chamber of the H-cell was first loaded with 5 mL of the electrolyte/ROM solution while the counter chamber was loaded with 5 mL of only 0.5 M $TBAPF_6$ in acetonitrile. One charging event of the working chamber was completed at which point the solution was removed from the counter chamber and replaced with 5 mL of the electrolyte/ROM solution. A discharge event was then conducted followed by 99 more charge-discharge cycles. Charging and discharging were all conducted with a current of 5 mA and both chambers of the H-cell were continuously stirred with magnetic stir bars. The upper and lower voltage cutoffs, unless otherwise indicated, were 0.4 V plus and minus the $E_{1/2}$ (determined with CV at a scan rate of 100 mV/s or 500 mV/s) for one-electron cycling and 0.4 V minus $E_{1/2}^1$ and 0.4 V plus $E_{1/2}^2$ for

two-electron cycling. Results of H-cell bulk electrolysis are displayed here in graphs of normalized discharge capacity (normalized relative to theoretical capacity) vs time for 100 (unless otherwise noted) charge-discharge cycles. Each data point in these figures represents one cycle. Use of time instead of cycle number in the figures is done in order to provide a more accurate representation of stability.[8]

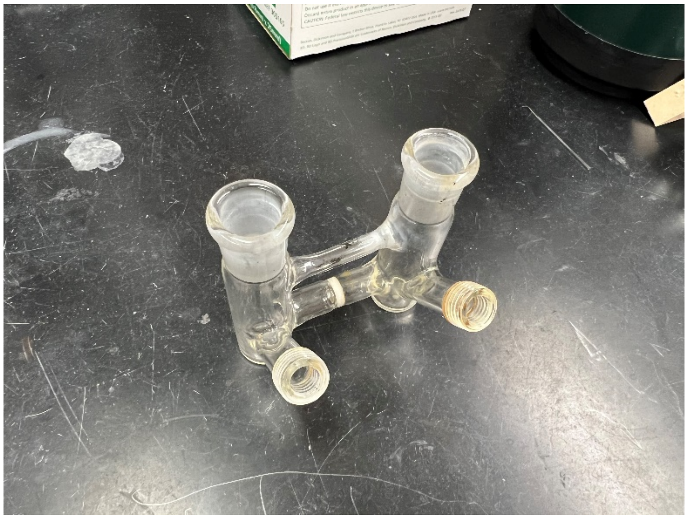

Figure SI-1: Picture of custom H-cell.

Flow Cell Cycling: Data from flow cell cycling was collected on a BioLogic VSP potentiostat (operated in a galvanostatic mode) in a nitrogen filled glovebox. The flow cell design used in these experiments was previously described in the literature by Brushett and coworkers (pictured below).[9] Briefly, the cell utilizes a zero-gap design that features graphite charge collectors built with interdigitated flow fields. Two layers of carbon felt (Sigracet® 29AA) with approximately 20% compression and with an active cross-sectional area of 2.55 cm^2 were utilized as electrodes on each side of the flow cell and held in place with ePTFA gaskets. The two sides of the flow cell were separated by a Daramic® AA-175 porous separator. All components of the flow cell were dried in an oven overnight, assembled outside of the glovebox, and immediately brought into the glovebox through an antechamber via a 1.5-hour evacuation/nitrogen backfill process. The assembled flow cell was allowed to equilibrate in the glovebox for 24 hours prior to use. Continuous flow was provided by a Cole-Parmer Masterflex® pump featuring a two-channel rotor through a combination of Masterflex compressible pump tubing and PFA flexible tubing. Flow rates were 20 mL/min with charge and discharge rates of 10 mA/cm^2. The upper and lower voltage cutoffs were either 0.35 V plus and minus the $\Delta E_{1/2}$ (determined with CV at a scan rate of 100 mV/s) or 0.35 V plus and 0.50 V minus the $\Delta E_{1/2}$ as indicated in the main manuscript text. An equilibration period of 45-60 mins was utilized before active charging and discharging, during which the working solutions were flowed through the cell. Details on the loading of each chamber of the flow system as well as data and results are detailed in the main text.

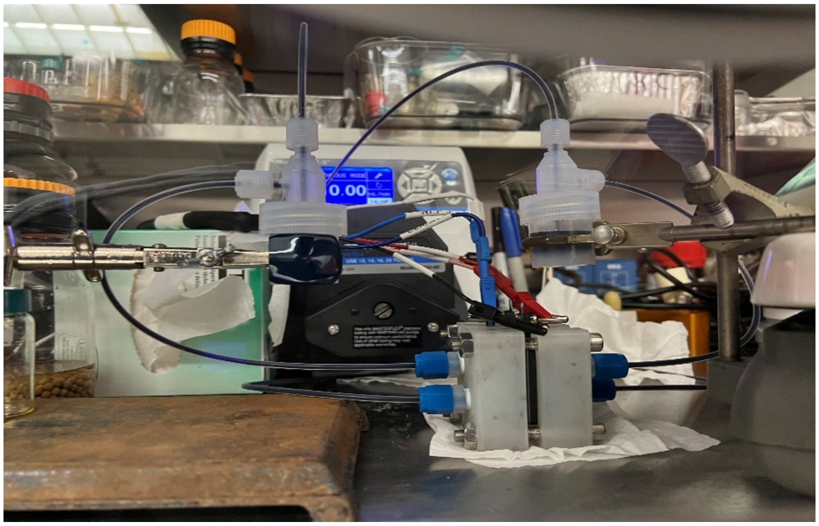

Figure SI-2: Picture of flow cell during a representative run.

H-Cell Studies:

3,8-bis(2-(2-methoxyethoxy)ethyl)-3,8-dihydrocyclobuta[b]quinoxaline-1,2-dione (**SQX-2**)

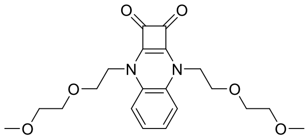

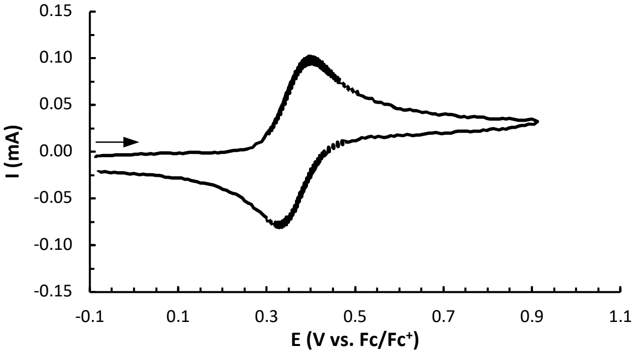

Figure SI-3: Cyclic voltammetry of **SQX-2** (5 mM) in 0.5 M TBAFPF$_6$ in MeCN at a scan rate of 100 mV/s.

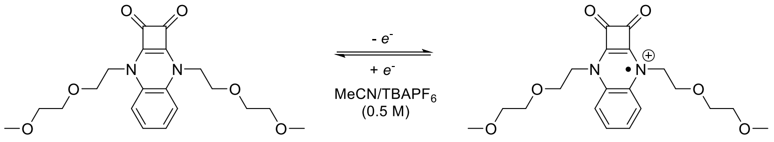

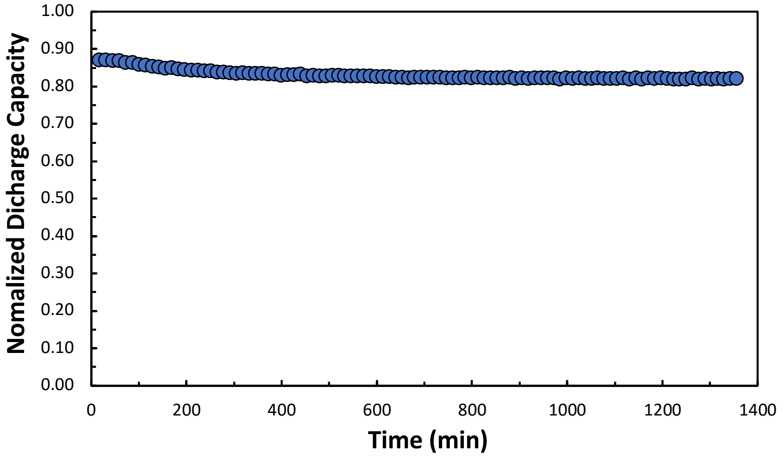

Figure SI-4: H-cell oxidative cycling (100 cycles) of **SQX-2** (5 mM) in 0.5 M TBAFPF$_6$ in MeCN at a current of 5 mA. Upper voltage cutoff 300 mV positive of $E_{1/2}$.

ethyl 3,8-bis(2-(2-methoxyethoxy)ethyl)-1,2-dioxo-1,2,3,8-tetrahydrocyclobuta[b]quinoxaline-5-carboxylate (**SQX-3**)

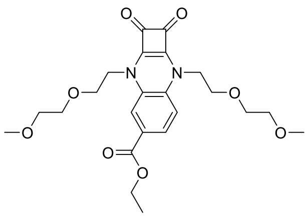

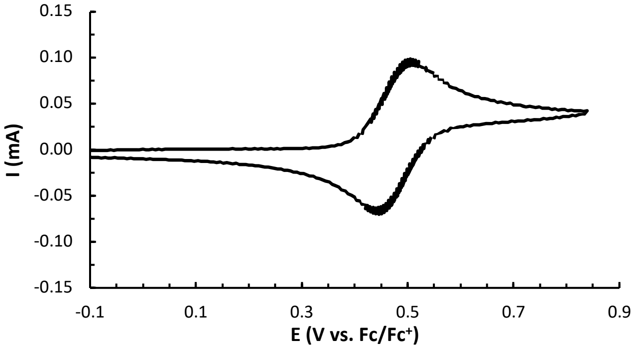

Figure SI-5: Cyclic voltammetry of **SQX-3** (5 mM) in 0.5 M $TBAFPF_6$ in MeCN at a scan rate of 100 mV/s.

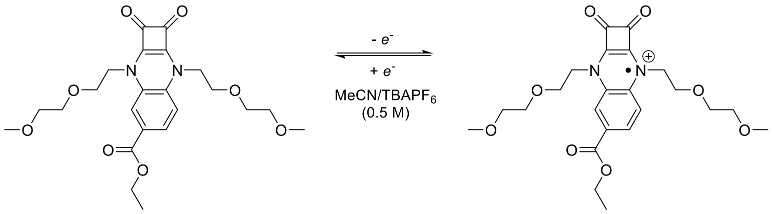

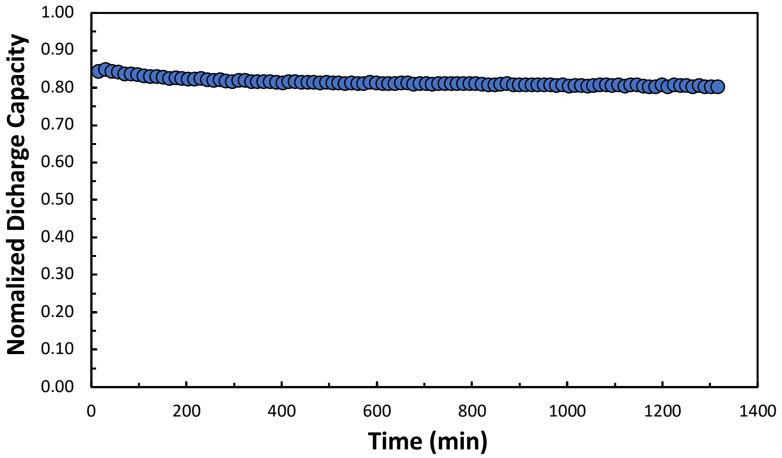

Figure SI-6: H-cell oxidative cycling (100 cycles) of **SQX-3** (5 mM) in 0.5 M $TBAFPF_6$ in MeCN at a current of 5 mA.

3,8-bis(2-(2-methoxyethoxy)ethyl)-5-(trifluoromethyl)-3,8-dihydrocyclobuta[b]quinoxaline-1,2-dione (**SQX-1**)

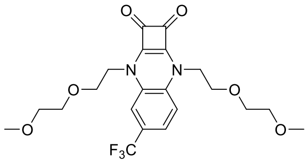

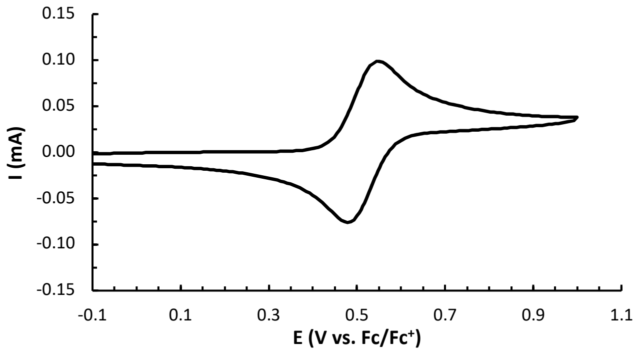

Figure SI-7: Cyclic voltammetry of **SQX-1** (5 mM) in 0.5 M TBAFPF$_6$ in MeCN at a scan rate of 100 mV/s.

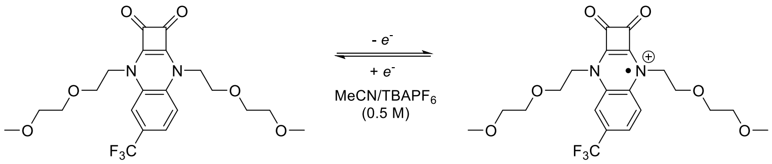

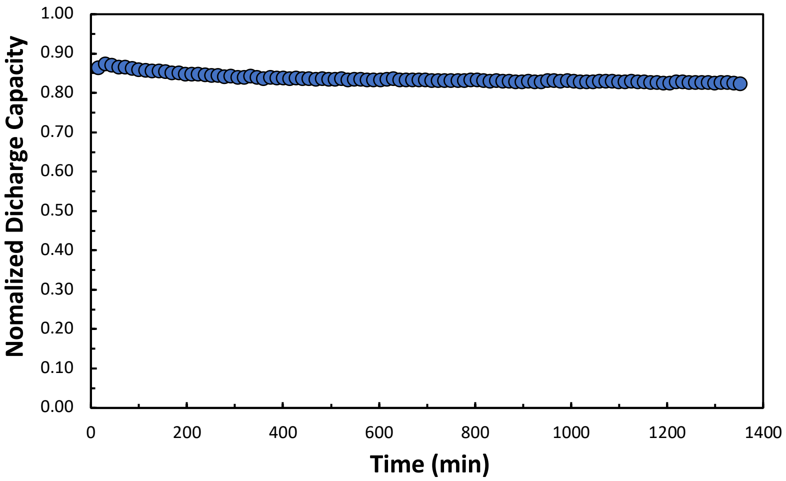

Figure SI-8: H-cell oxidative cycling (100 cycles) of **SQX-1** (5 mM) in 0.5 M TBAFPF$_6$ in MeCN at a current of 5 mA.

diethyl 3,8-bis(2-(2-methoxyethoxy)ethyl)-1,2-dioxo-1,2,3,8-tetrahydrocyclobuta[b]quinoxaline-5,6-dicarboxylate (**SQX-4**)

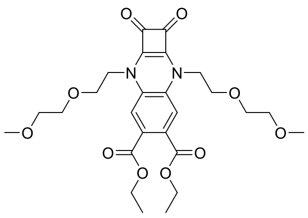

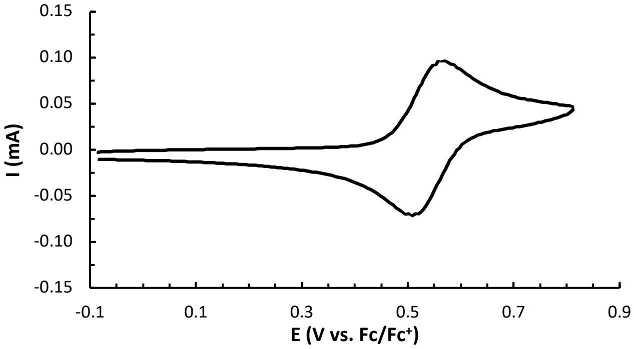

Figure SI-9: Cyclic voltammetry of **SQX-4** (5 mM) in 0.5 M $TBAFPF_6$ in MeCN at a scan rate of 100 mV/s.

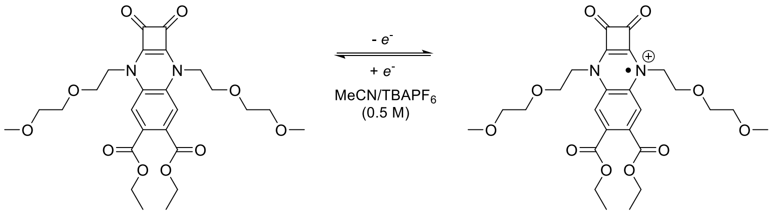

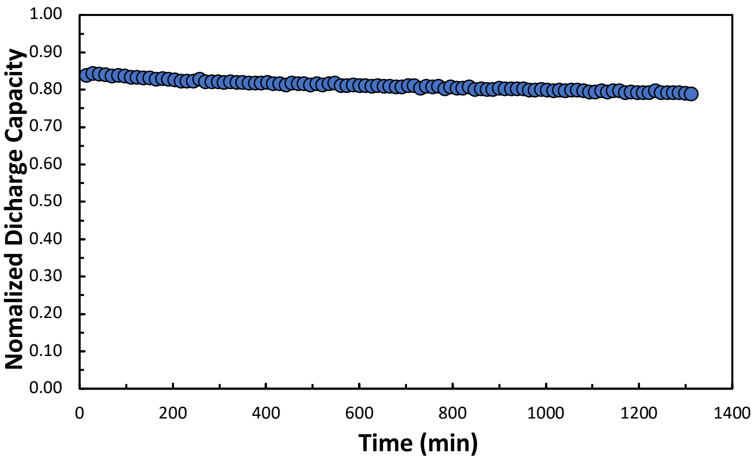

Figure SI-10: H-cell oxidative cycling (100 cycles) of **SQX-4** (5 mM) in 0.5 M $TBAFPF_6$ in MeCN at a current of 5 mA.

2,2'-(5-(ethoxycarbonyl)-1,2-dioxo-1,2-dihydrocyclobuta[b]quinoxaline-3,8-diyl)bis(N,N,N-trimethylethan-1-aminium) bis(hexafluorophosphate) (**SQX-5**)

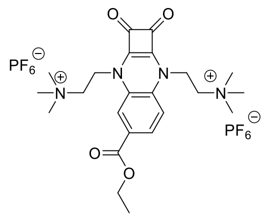

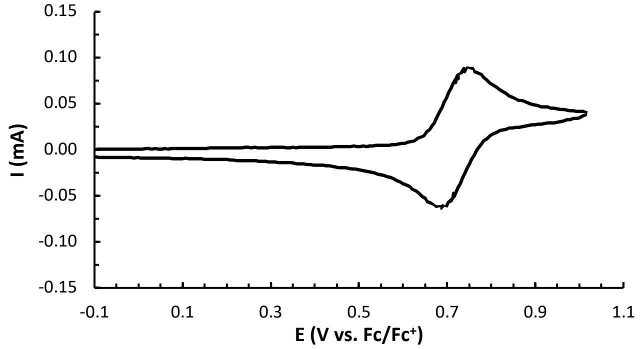

Figure SI-11: Cyclic voltammetry of **SQX-5** (5 mM) in 0.5 M $TBAFPF_6$ in MeCN at a scan rate of 100 mV/s.

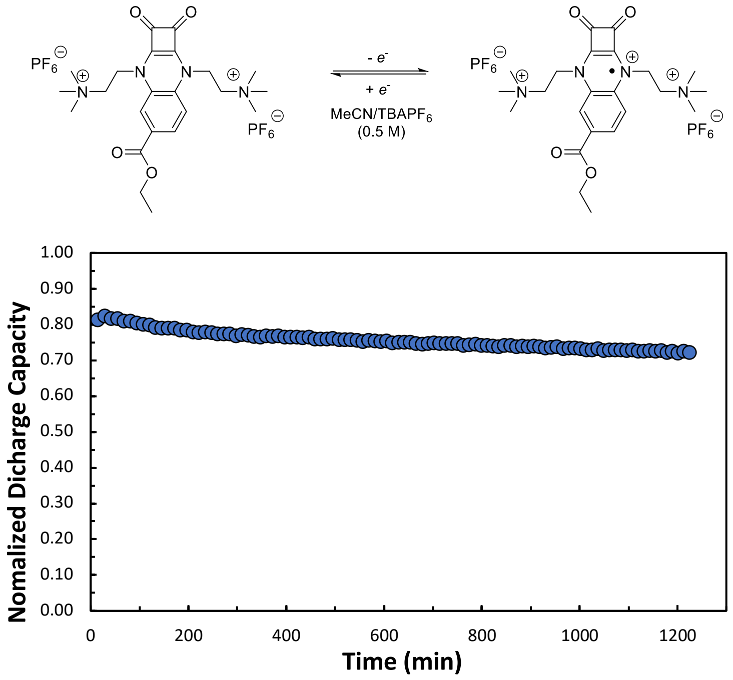

Figure SI-12: H-cell oxidative cycling (100 cycles) of **SQX-5** (5 mM) in 0.5 M $TBAFPF_6$ in MeCN at a current of 5 mA.

<u>3,4-bis(butyl(phenyl)amino)cyclobut-3-ene-1,2-dione</u> (**SQA-3**)

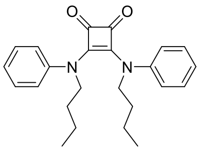

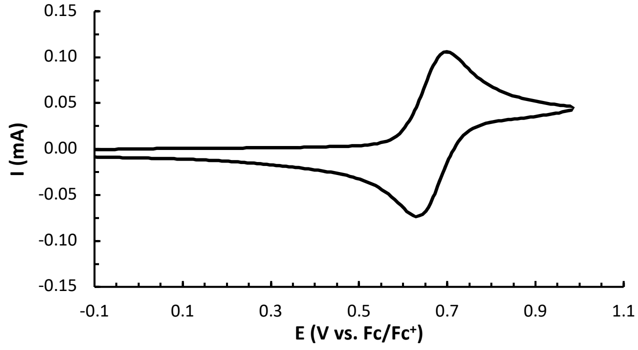

Figure SI-13: Cyclic voltammetry of **SQA-3** (5 mM) in 0.5 M $TBAFPF_6$ in MeCN at a scan rate of 100 mV/s.

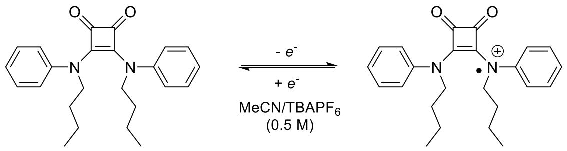

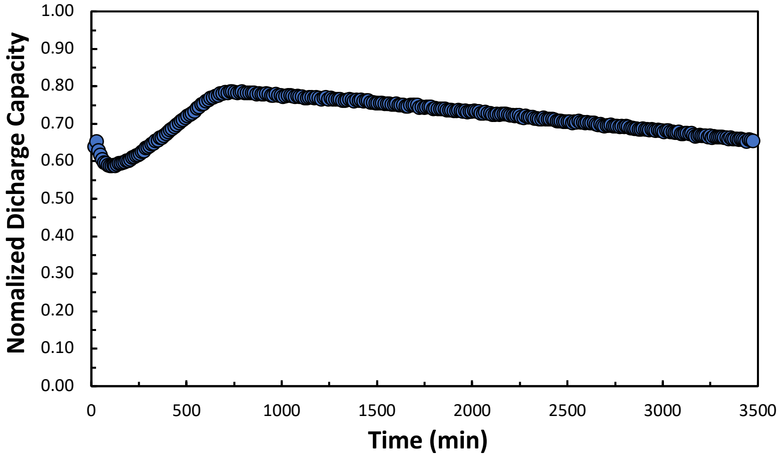

Figure SI-14: H-cell oxidative cycling (300 cycles) of **SQA-3** (5 mM) in 0.5 M $TBAFPF_6$ in MeCN at a current of 5 mA. Upper voltage cutoff 250 mV positive of $E_{1/2}$.

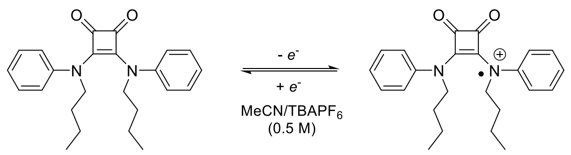

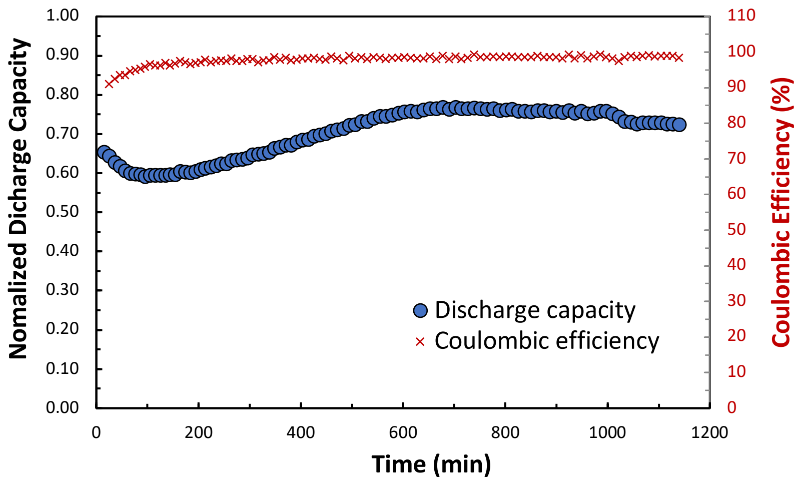

Figure SI-15: H-cell oxidative cycling (100 cycles) of **SQA-3** (5 mM) in 0.5 M $TBAFPF_6$ in MeCN at a current of 5 mA aong with coulombic efficiency. Upper voltage cutoff 300 mV positive of $E_{1/2}$.

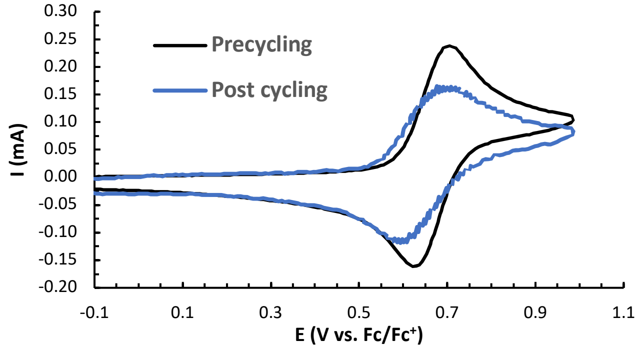

Figure SI-16: Cyclic voltammetry of **SQA-3** (5 mM) in 0.5 M $TBAFPF_6$ in MeCN at a scan rate of 500 mV/s before and after 100 H-cell charge-discharge cycles.

Post cycling analysis and characterization of biaryl dimer <u>(4,4'-([1,1'-biphenyl]-4,4'-diylbis(butylazanediyl))bis(3-(butyl(phenyl)amino)cyclobut-3-ene-1,2-dione))</u> (2): After 100 H-cell charge-discharge cycles ending on a complete discharge, the material on the working side of the H-cell was removed from the glovebox and diluted with 100 mL of diethyl ether. The resulting precipitated $TBAPF_6$ was removed via vacuum filtration and the crude filtrate was concentrated under reduced pressure. A crude ^1H NMR was then obtained in $CDCl_3$ after which the major product was isolated via preparatory TLC (3:1

hexane/ethyl acetate then 3:2 hexane/ethyl acetate). Analysis of the resulting ^{1}H NMR, ^{13}C NMR, and HRMS data allowed us to assign the major component of the crude reaction mixture to be the biaryl dimer of the original squaramide catholyte (see below). This presumably forms during the initial charging cycles and its formation is likely responsible for the low initial coulombic efficiency that eventually approaches 99% as well the apparent drop and subsequent increase in battery capacity.

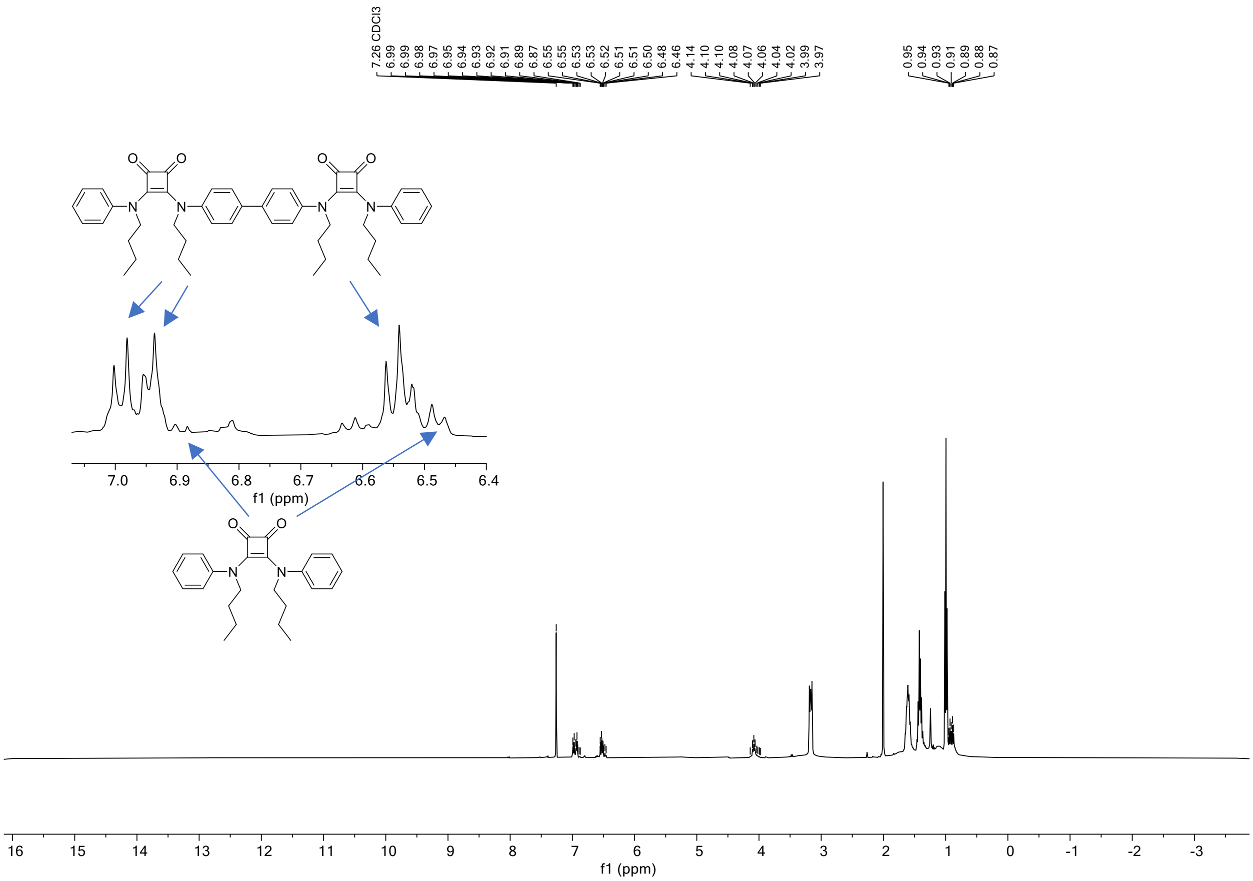

Figure SI-17: Crude NMR of the working side of the H-cell following H-cell oxidative cycling of **SQA-3** (5 mM) in 0.5 M $TBAFPF_6$ in MeCN at a current of 5 mA.

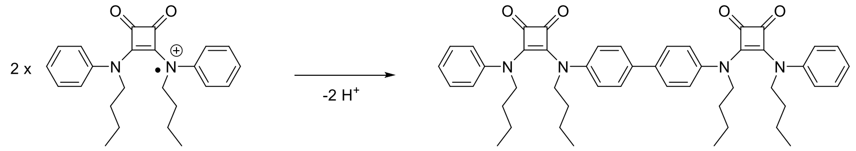

^{1}H NMR (500 MHz, $CDCl_3$) δ 7.00 – 6.97 (m, 4H), 6.97 – 6.90 (m, 6H), 6.58 – 6.48 (m, 8H), 4.09 (t, J = 7.6 Hz, 4H), 4.08 (t, J = 7.6 Hz, 4H), 1.70 – 1.62 (m, 4H), 1.62 – 1.57 (m, 4H), 1.39 (m, 8H), 0.94 (t, J = 7.4 Hz, 6H), 0.90 (t, J = 7.4 Hz, 6H). ^{13}C NMR (126 MHz, $CDCl_3$) δ 186.5, 167.8, 167.4, 141.6, 140.9, 137.1, 128.6, 127.0, 125.2,

122.9, 122.8, 52.7, 52.6, 32.3, 32.2, 19.9, 19.8, 14.0, 13.9. HRMS (ESI) calculated for $C_{48}H_{54}N_4O_4$ (H+): 751.4218, found: 751.4223.

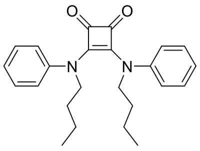

(Two-electron)

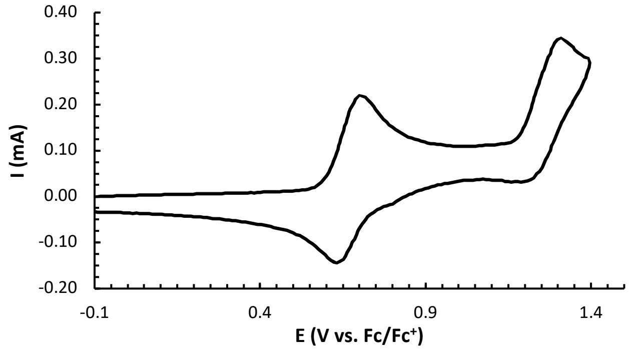

Figure SI-18: Cyclic voltammetry of **SQA-3** (5 mM) in 0.5 M $TBAFPF_6$ in MeCN at a scan rate of 500 mV/s showing two oxidations.

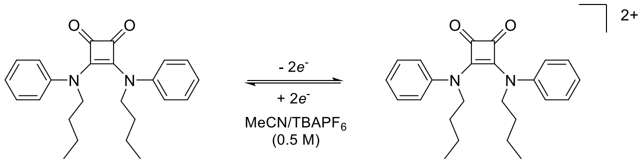

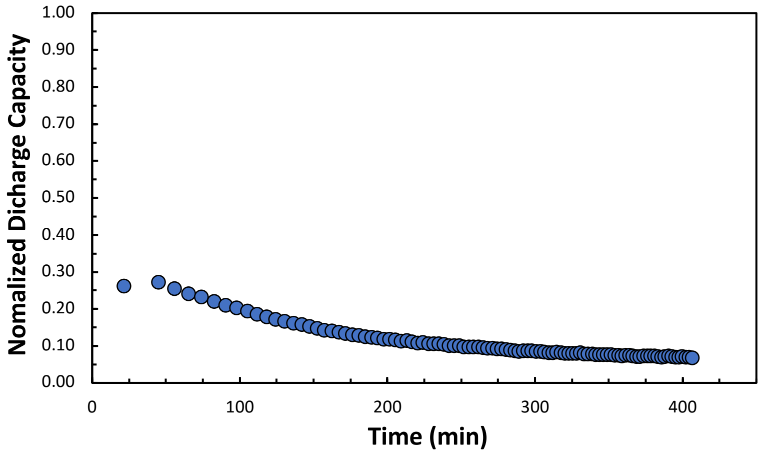

Figure SI-19: H-cell two-electron oxidative cycling (100 cycles) of **SQA-3** (5 mM) in 0.5 M $TBAFPF_6$ in MeCN at a current of 5 mA. Upper voltage cutoff 130 mV positive of $E_{1/2}^2$.

3,4-bis(butyl(4-(trifluoromethyl)phenyl)amino)cyclobut-3-ene-1,2-dione (**SQA-4**)

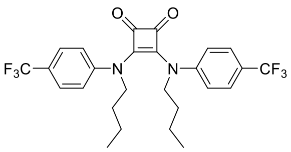

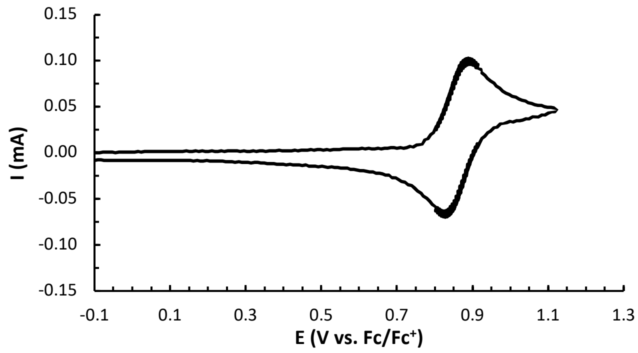

Figure SI-20: Cyclic voltammetry of **SQA-4** (5 mM) in 0.5 M $TBAFPF_6$ in MeCN at a scan rate of 100 mV/s.

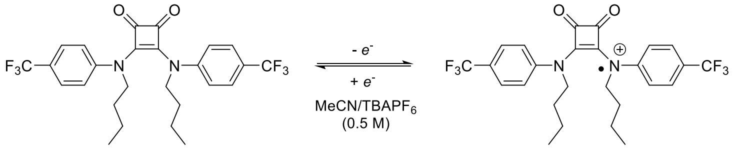

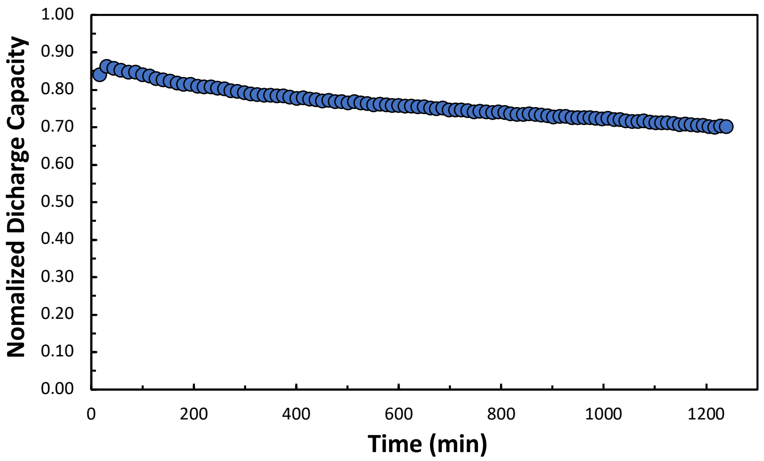

Figure SI-21: H-cell oxidative cycling (100 cycles) of **SQA-4** (5 mM) in 0.5 M $TBAFPF_6$ in MeCN at a current of 5 mA. Upper voltage cutoff 250 mV positive of $E_{1/2}$.

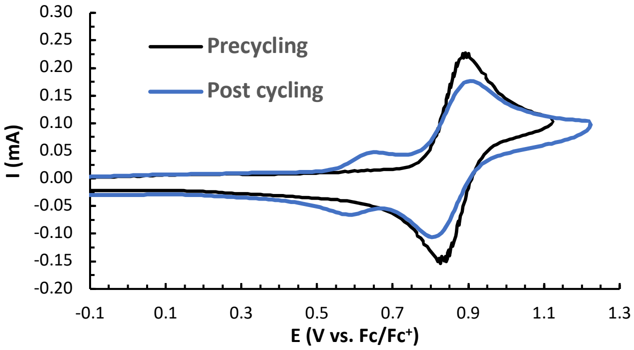

Figure SI-22: Cyclic voltammetry of **SQA-4** (5 mM) in 0.5 M TBAFPF$_6$ in MeCN at a scan rate of 500 mV/s before and after 100 H-cell charge-discharge cycles. Ratio of $I_{pa,\mathbf{3}}/I_{pa,\mathbf{SQA}}$ = 0.27 where $I_{pa,\mathbf{3}}$ is the peak anodic current corresponding to the generic byproduct **3** and $I_{pa,\mathbf{SQA}}$ is the peak anodic current corresponding to the remaining SQA.

3,4-bis(butyl(3-(trifluoromethyl)phenyl)amino)cyclobut-3-ene-1,2-dione (**SQA-5**)

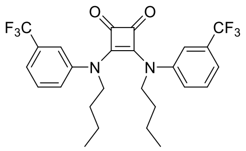

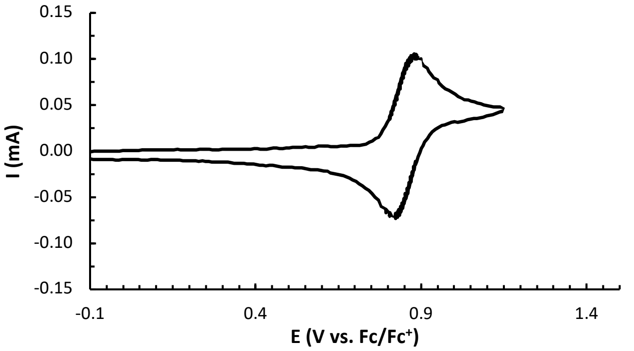

Figure SI-23: Cyclic voltammetry of **SQA-5** (5 mM) in 0.5 M TBAFPF$_6$ in MeCN at a scan rate of 100 mV/s.

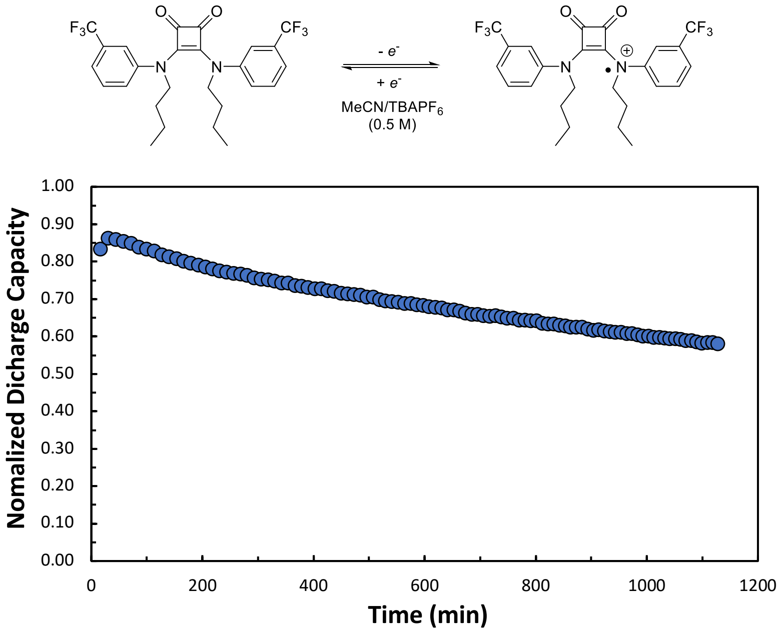

Figure SI-24: H-cell oxidative cycling (100 cycles) of **SQA-5** (5 mM) in 0.5 M $TBAFPF_6$ in MeCN at a current of 5 mA.

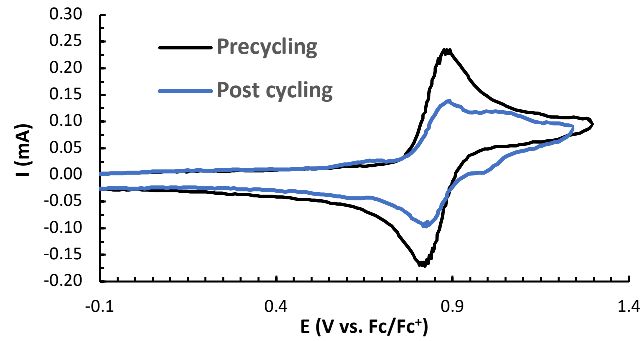

Figure SI-25: Cyclic voltammetry of **SQA-5** (5 mM) in 0.5 M $TBAFPF_6$ in MeCN at a scan rate of 500 mV/s before and after 100 H-cell charge-discharge cycles. Ratio of $I_{pa,\mathbf{3}}/I_{pa,\mathbf{SQA}}$ = 0.13.

dimethyl 4,4'-((3,4-dioxocyclobut-1-ene-1,2-diyl)bis(butylazanediyl))dibenzoate (**SQA-6**)

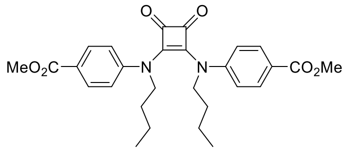

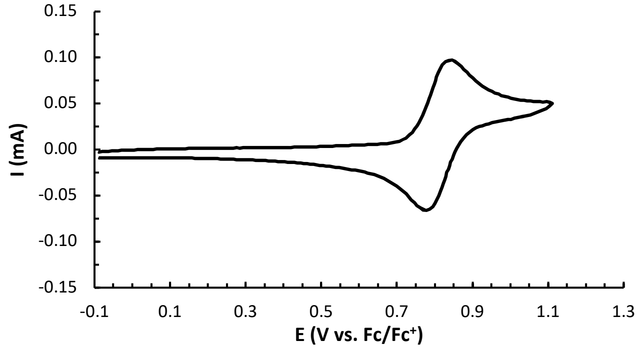

Figure SI-26: Cyclic voltammetry of **SQA-6** (5 mM) in 0.5 M $TBAFPF_6$ in MeCN at a scan rate of 100 mV/s.

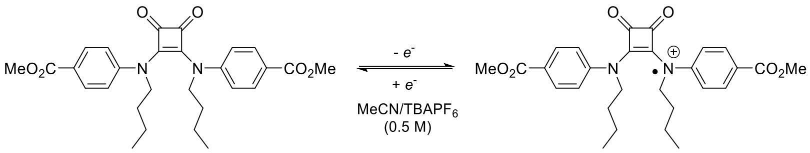

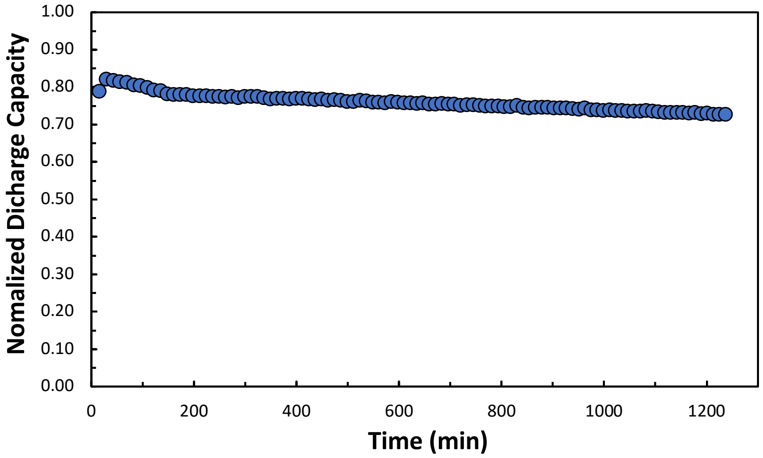

Figure SI-27: H-cell oxidative cycling (100 cycles) of **SQA-6** (5 mM) in 0.5 M $TBAFPF_6$ in MeCN at a current of 5 mA. Upper voltage cutoff 270 mV positive of $E_{1/2}$.

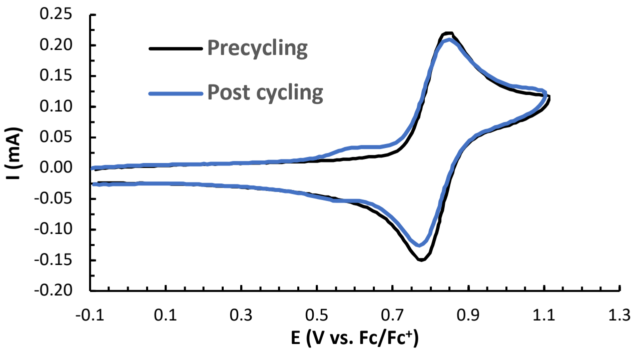

Figure SI-28: Cyclic voltammetry of **SQA-6** (5 mM) in 0.5 M $TBAFPF_6$ in MeCN at a scan rate of 500 mV/s before and after 100 H-cell charge-discharge cycles. Ratio of $I_{pa,\mathbf{3}}/I_{pa,\mathbf{SQA}}$ = 0.11.

In order to definitively identify the decomposition product with a more negative oxidation potential the following procedure was performed:

Post cycling analysis and characterization of <u>methyl 8-butyl-3-(4-(methoxycarbonyl)phenyl)-1,2-dioxo-1,2,3,8-tetrahydrocyclobuta[b]quinoxaline-5-carboxylate</u> (**3, R =** $\mathbf{CO_2Me}$) (JST-III-6)**:** After 100 H-cell charge-discharge cycles ending on a complete discharge, the material on the working side of the H-cell was removed from the glovebox and diluted with 100 mL of diethyl ether. The resulting precipitated $TBAPF_6$ was removed via vacuum filtration and the crude filtrate was concentrated under reduced pressure. The major decomposition product was isolated via preparatory TLC (2:1 hexanes/ethyl acetate). Analysis of the resulting ^{1}H NMR, HRMS, and CV data allowed us to assign the decomposition product to be the intramolecular diaminoaryl product **3 R=** $\mathbf{CO_2Me}$ (see below). The other similarly observed decomposition products for other squaramide catholytes are assigned by analogy. This product can be thought to formally arise from intramolecular addition of a nitrogen nucleophile to an electron deficient arene (due to the radical cation behavior) followed by the formal loss of butane and the addition of an electron.

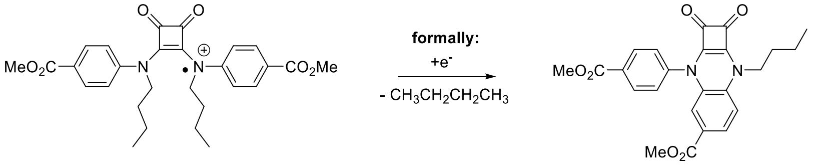

^{1}H NMR (400 MHz, $CDCl_3$) δ 8.24 – 8.17 (m, 2H), 7.54 (d, J = 8.2 Hz, 1H), 7.47 (d, J = 8.4 Hz, 2H), 7.10 (d, J = 1.7 Hz, 1H), 6.55 (d, J = 8.3 Hz, 1H), 3.96 (s, 3H), 3.80 (s, 3H), 3.67 (t, J = 7.1 Hz, 2H), 1.74 (q, J = 7.4 Hz, 2H), 1.53 – 1.46 (m, 2H), 1.03 (t, J = 7.4 Hz, 3H). HRMS (ESI) m/z calculated for $C_{24}H_{22}N_2O_6$ (Na+): 457.1370, found: 457.1371.

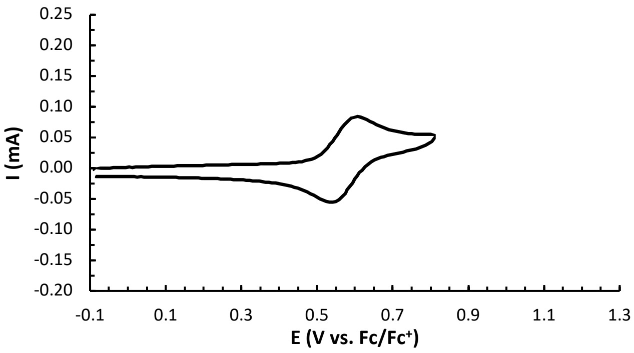

Figure SI-29: Cyclic voltammetry of isolated **3 R=CO_2Me** (3.5 mM) in 0.5 M $TBAFPF_6$ in MeCN at a scan rate of 500 mV/s.

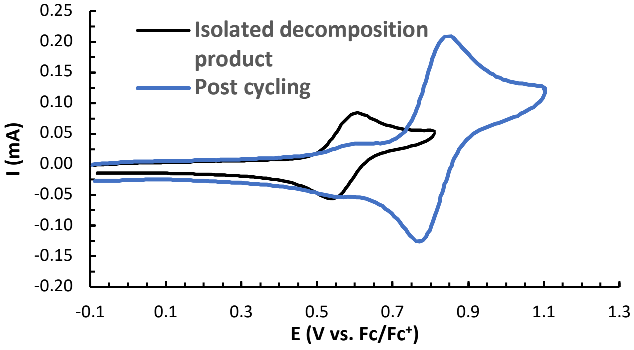

Figure SI-30: Cyclic voltammetry of **3 R=CO_2Me** (3.5 mM) in 0.5 M $TBAFPF_6$ in MeCN at a scan rate of 500 mV/s along with the cyclic voltammetry of **SQA-6** (5 mM) in 0.5 M $TBAFPF_6$ in MeCN at a scan rate of 500 mV/s after 100 H-cell charge-discharge cycles.

diethyl 4,4'-((3,4-dioxocyclobut-1-ene-1,2-diyl)bis(butylazanediyl))dibenzoate (**SQA-7**)

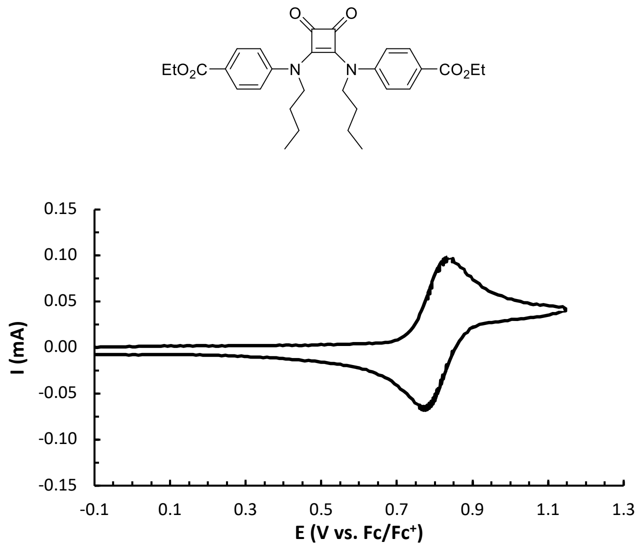

Figure SI-31: Cyclic voltammetry of **SQA-7** (5 mM) in 0.5 M $TBAFPF_6$ in MeCN at a scan rate of 100 mV/s.

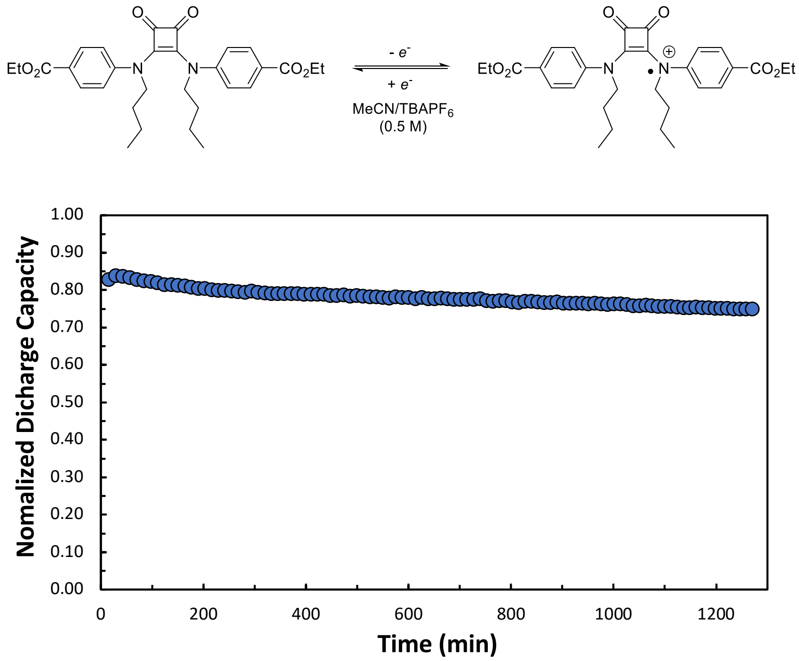

Figure SI-32: H-cell oxidative cycling (100 cycles) of **SQA-7** (5 mM) in 0.5 M $TBAFPF_6$ in MeCN at a current of 5 mA.

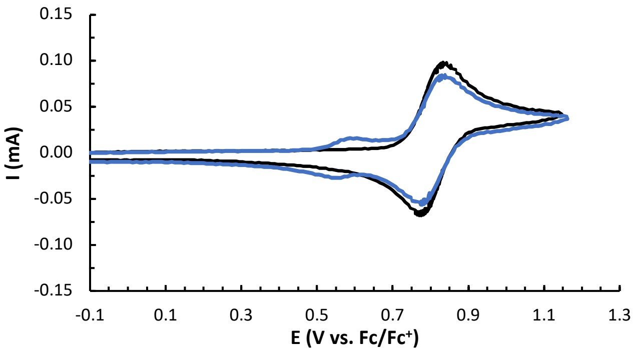

Figure SI-33: Cyclic voltammetry of **SQA-7** (5 mM) in 0.5 M TBAFPF$_6$ in MeCN at a scan rate of 100 mV/s before and after 100 H-cell charge-discharge cycles. Ratio of $I_{pa,\mathbf{3}}/I_{pa,\mathbf{SQA}}$ = 0.20.

di-tert-butyl 4,4'-((3,4-dioxocyclobut-1-ene-1,2-diyl)bis(butylazanediyl))dibenzoate (**SQA-8**)

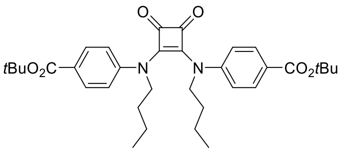

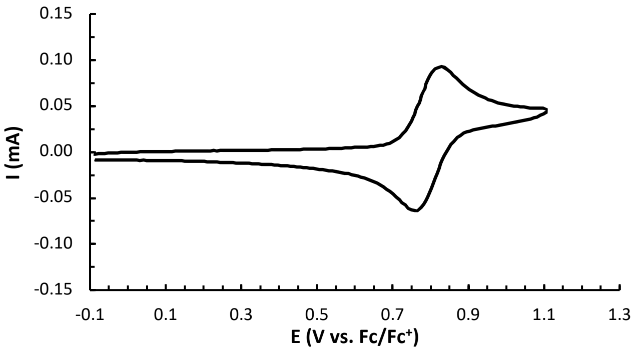

Figure SI-34: Cyclic voltammetry of **SQA-8** (5 mM) in 0.5 M TBAFPF$_6$ in MeCN at a scan rate of 100 mV/s.

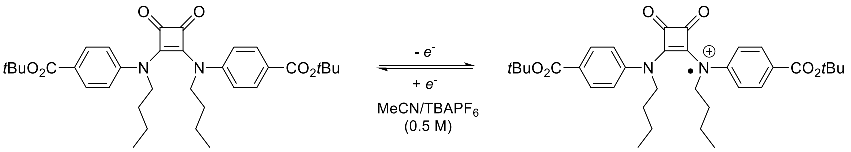

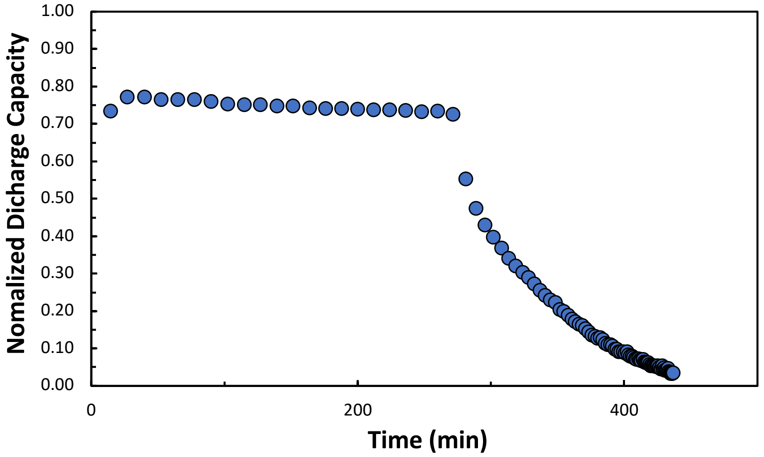

Figure SI-35: H-cell oxidative cycling (100 cycles) of **SQA-8** (5 mM) in 0.5 M $TBAFPF_6$ in MeCN at a current of 5 mA. Upper voltage cutoff 260 mV positive of $E_{1/2}$.

The results of this H-cell cycling data were replicated and we believe that the rapid decomposition is a real phenomenon potentially caused by a self-catalytic process involving decomposition of the *t*-butyl ester.

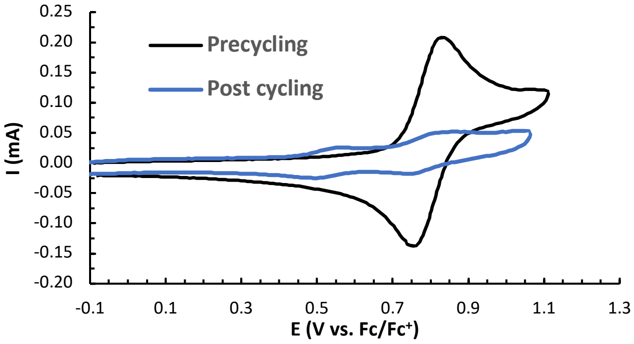

Figure SI-36: Cyclic voltammetry of **SQA-8** (5 mM) in 0.5 M $TBAFPF_6$ in MeCN at a scan rate of 500 mV/s before and after 100 H-cell charge-discharge cycles. Ratio of $I_{pa,\mathbf{3}}/I_{pa,\mathbf{SQA}}$ = 0.57.

3,4-bis((4-bromophenyl)(butyl)amino)cyclobut-3-ene-1,2-dione (**SQA-9**)

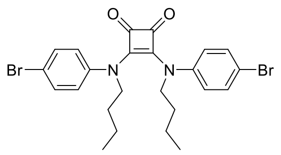

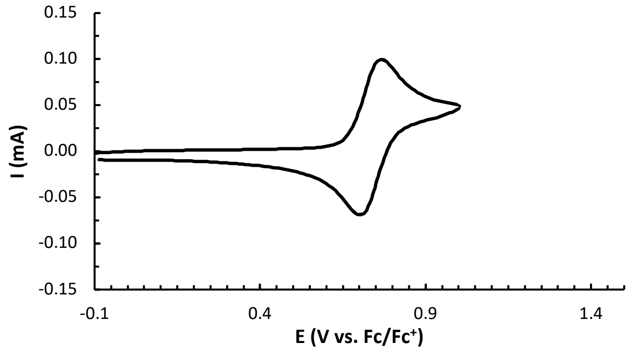

Figure SI-37: Cyclic voltammetry of **SQA-9** (5 mM) in 0.5 M $TBAFPF_6$ in MeCN at a scan rate of 100 mV/s.

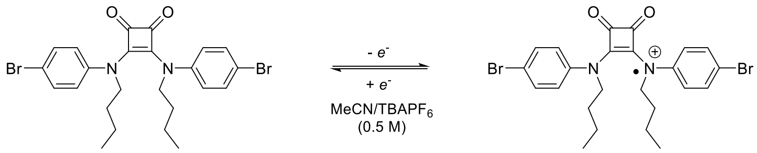

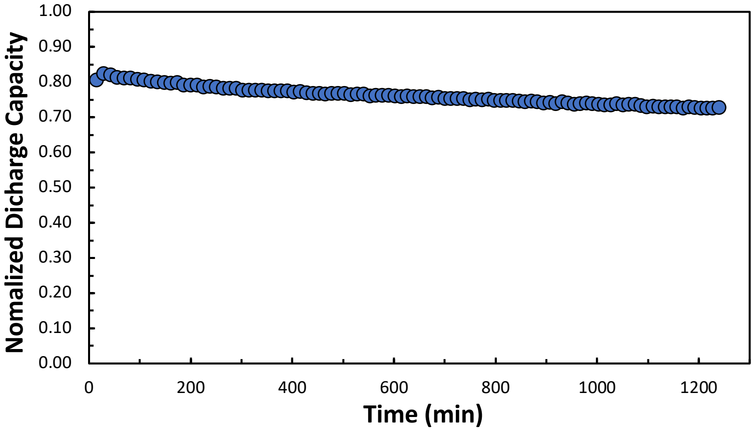

Figure SI-38: H-cell oxidative cycling (100 cycles) of **SQA-9** (5 mM) in 0.5 M $TBAFPF_6$ in MeCN at a current of 5 mA.

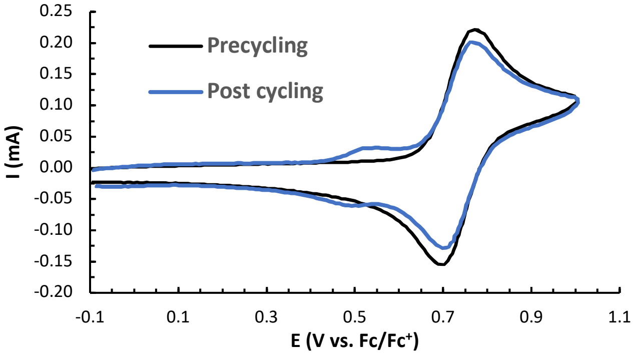

Figure SI-39: Cyclic voltammetry of **SQA-9** (5 mM) in 0.5 M $TBAFPF_6$ in MeCN at a scan rate of 500 mV/s before and after 100 H-cell charge-discharge cycles. Ratio of $I_{pa,\mathbf{3}}/I_{pa,\mathbf{SQA}}$ = 0.16.

3,4-bis(butyl(4-chloro-2,6-difluorophenyl)amino)cyclobut-3-ene-1,2-dione (**SQA-10**)

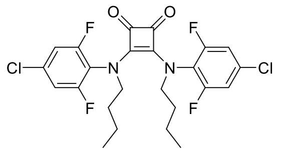

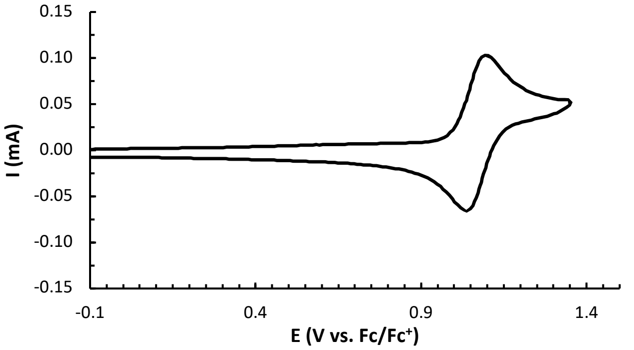

Figure SI-40: Cyclic voltammetry of **SQA-10** (5 mM) in 0.5 M $TBAFPF_6$ in MeCN at a scan rate of 100 mV/s.

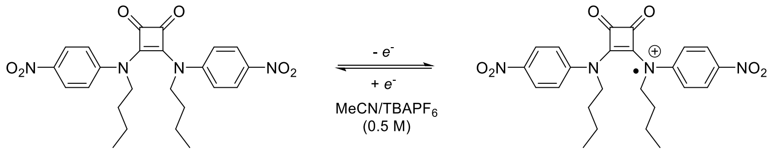

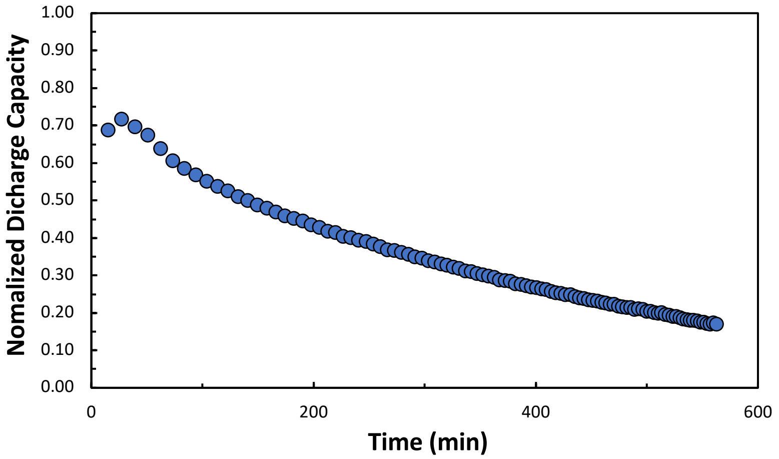

Figure SI-41: H-cell oxidative cycling (100 cycles) of **SQA-10** (5 mM) in 0.5 M $TBAFPF_6$ in MeCN at a current of 5 mA.

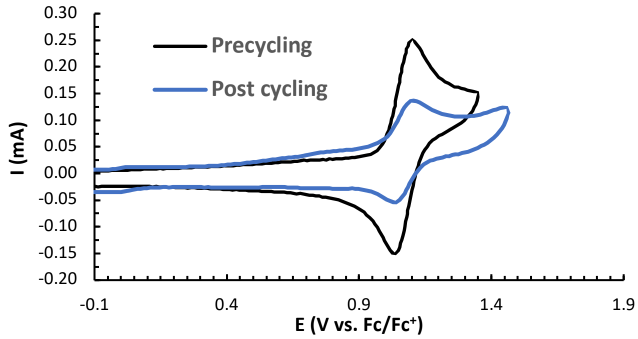

Figure SI-42: Cyclic voltammetry of **SQA-10** (5 mM) in 0.5 M $TBAFPF_6$ in MeCN at a scan rate of 500 mV/s before and after 100 H-cell charge-discharge cycles. Ratio of $I_{pa,\mathbf{3}}/I_{pa,\mathbf{SQA}}$ = 0.06.

3,4-bis(butyl(4-nitrophenyl)amino)cyclobut-3-ene-1,2-dione (**SQA-11**)

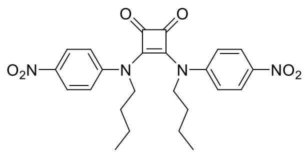

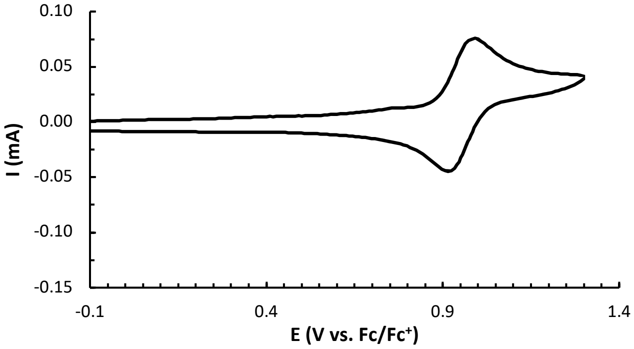

Figure SI-43: Cyclic voltammetry of **SQA-11** (4.15 mM) in 0.5 M $TBAFPF_6$ in MeCN at a scan rate of 100 mV/s.

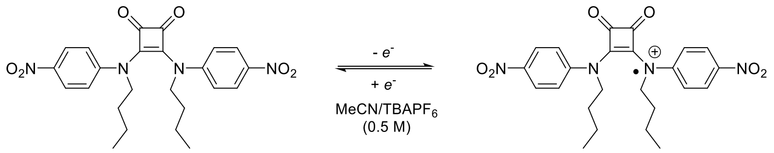

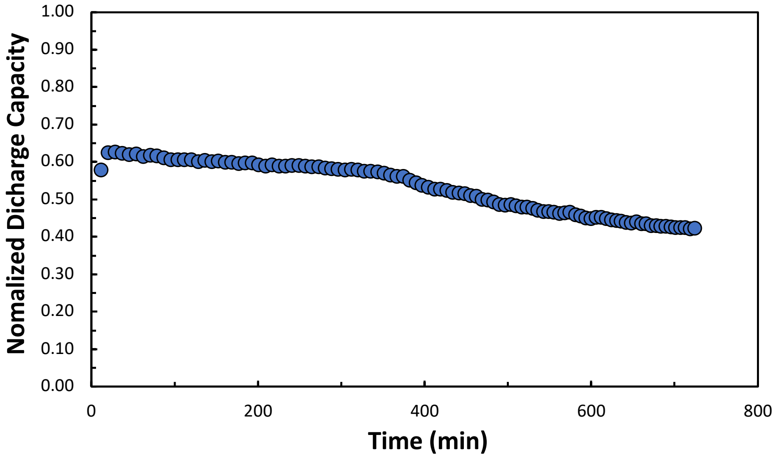

Figure SI-44: H-cell oxidative cycling (100 cycles) of **SQA-11** (4.15 mM) in 0.5 M $TBAFPF_6$ in MeCN at a current of 5 mA. Upper voltage cutoff 250 mV positive of $E_{1/2}$.

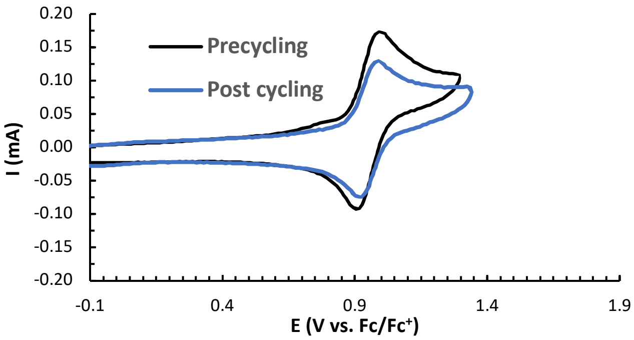

Figure SI-45: Cyclic voltammetry of **SQA-11** (4.15 mM) in 0.5 M TBAFPF$_6$ in MeCN at a scan rate of 500 mV/s before and after 100 H-cell charge-discharge cycles. No cyclized byproduct observed.

diethyl 4,4'-((3,4-dioxocyclobut-1-ene-1,2-diyl)bis(methylazanediyl))dibenzoate (**SQA-12**)

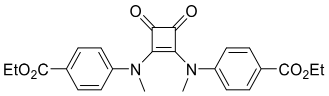

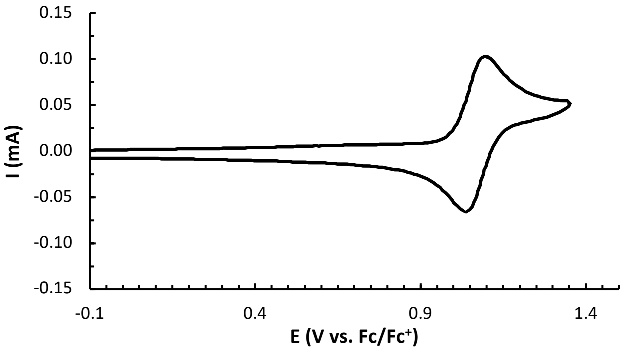

Figure SI-46: Cyclic voltammetry of **SQA-12** (5 mM) in 0.5 M TBAFPF$_6$ in MeCN at a scan rate of 100 mV/s.

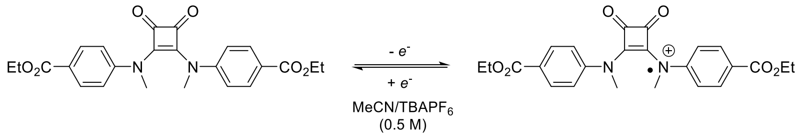

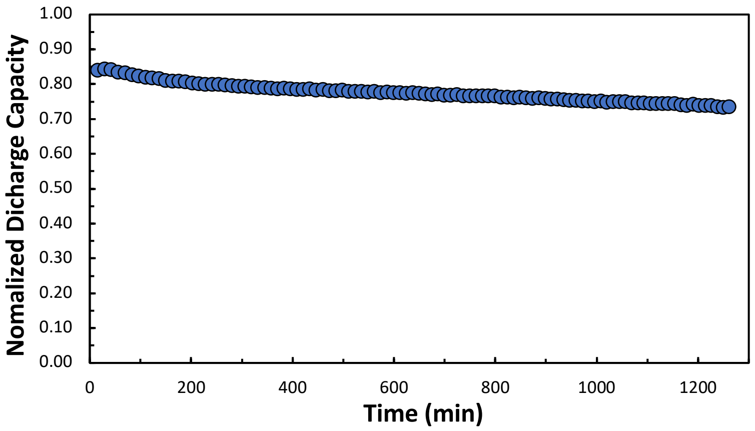

Figure SI-47: H-cell oxidative cycling (100 cycles) of **SQA-12** (5 mM) in 0.5 M $TBAFPF_6$ in MeCN at a current of 5 mA.

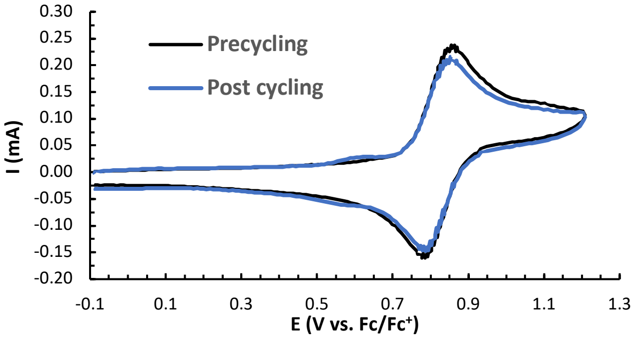

Figure SI-48: Cyclic voltammetry of **SQA-12** (5 mM) in 0.5 M $TBAFPF_6$ in MeCN at a scan rate of 500 mV/s before and after 100 H-cell charge-discharge cycles. Ratio of $I_{pa,\mathbf{3}}/I_{pa,\mathbf{SQA}}$ = 0.08.

ethyl 4-(butyl(2-((4-(ethoxycarbonyl)phenyl)(isopropyl)amino)-3,4-dioxocyclobut-1-en-1-yl)amino)benzoate (**SQA-13**)

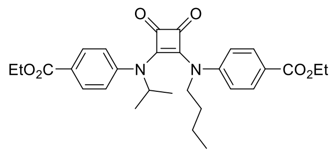

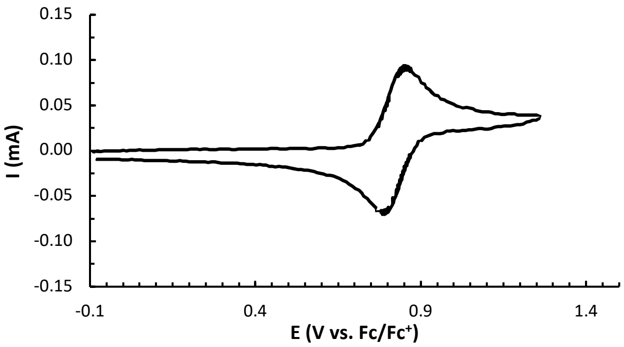

Figure SI-49: Cyclic voltammetry of **SQA-13** (5 mM) in 0.5 M $TBAFPF_6$ in MeCN at a scan rate of 100 mV/s.

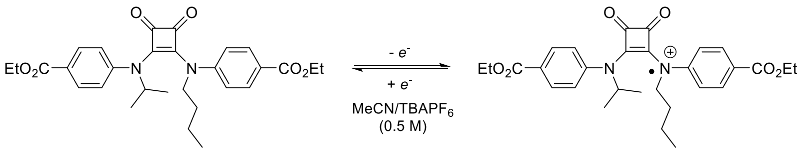

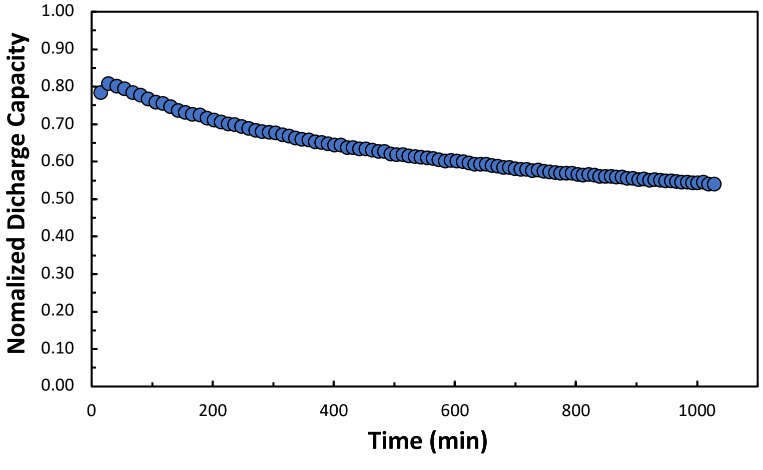

Figure SI-50: H-cell oxidative cycling (100 cycles) of **SQA-13** (5 mM) in 0.5 M $TBAFPF_6$ in MeCN at a current of 5 mA.

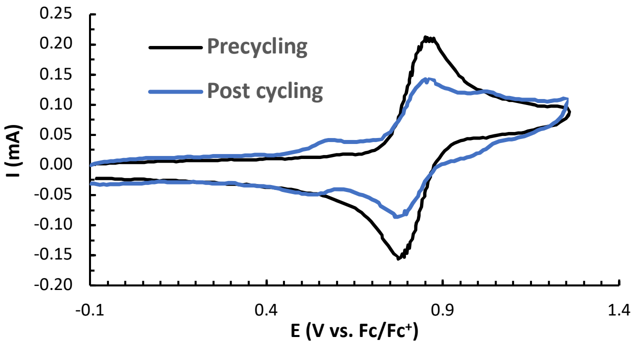

Figure SI-51: Cyclic voltammetry of **SQA-13** (5 mM) in 0.5 M $TBAFPF_6$ in MeCN at a scan rate of 500 mV/s before and after 100 H-cell charge-discharge cycles. Ratio of $I_{pa,\mathbf{3}}/I_{pa,\mathbf{SQA}}$ = 0.26.

<u>diethyl 4,4'-(7,8-dioxo-2,5-diazabicyclo[4.2.0]oct-1(6)-ene-2,5-diyl)dibenzoate</u> (**SQA-14**)

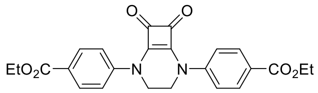

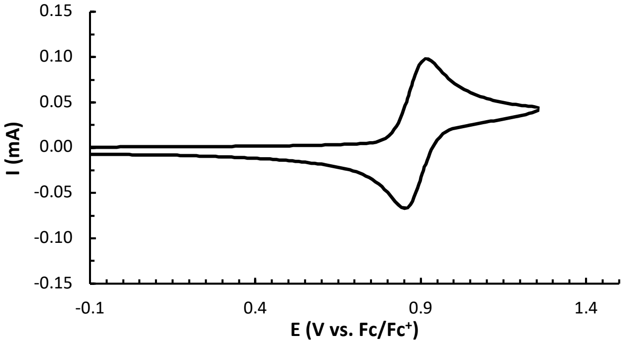

Figure SI-52: Cyclic voltammetry of **SQA-14** (5 mM) in 0.5 M $TBAFPF_6$ in MeCN at a scan rate of 100 mV/s.

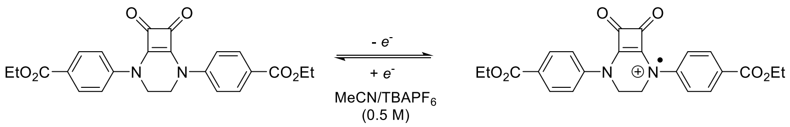

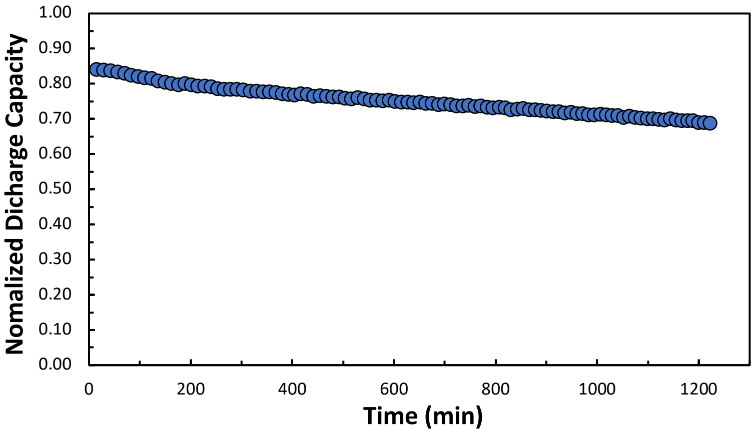

Figure SI-53: H-cell oxidative cycling (100 cycles) of **SQA-14** (5 mM) in 0.5 M $TBAFPF_6$ in MeCN at a current of 5 mA.

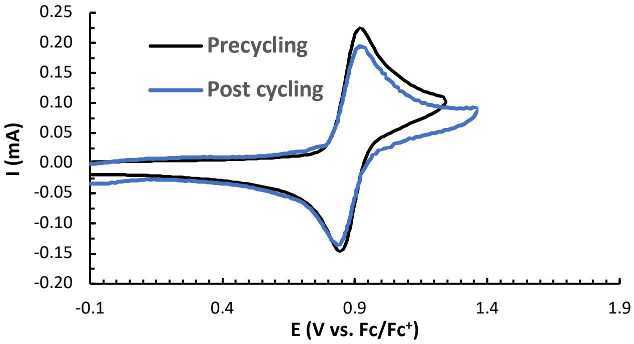

Figure SI-54: Cyclic voltammetry of **SQA-14** (5 mM) in 0.5 M $TBAFPF_6$ in MeCN at a scan rate of 500 mV/s before and after 100 H-cell charge-discharge cycles. Ratio of $I_{pa,\mathbf{3}}/I_{pa,\mathbf{SQA}}$ < 0.05 (unclear if any is present).

2,5-bis(2-(trifluoromethyl)phenyl)-2,5-diazabicyclo[4.2.0]oct-1(6)-ene-7,8-dione (**SQA-15**)

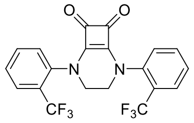

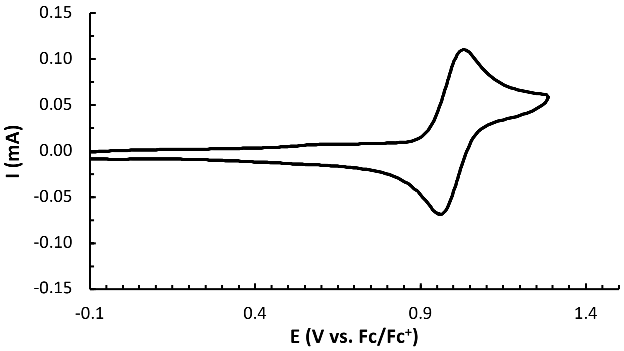

Figure SI-55: Cyclic voltammetry of **SQA-15** (5 mM) in 0.5 M $TBAFPF_6$ in MeCN at a scan rate of 100 mV/s.

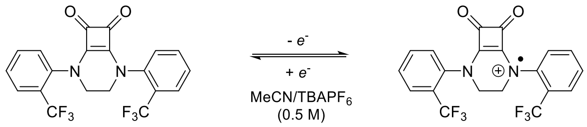

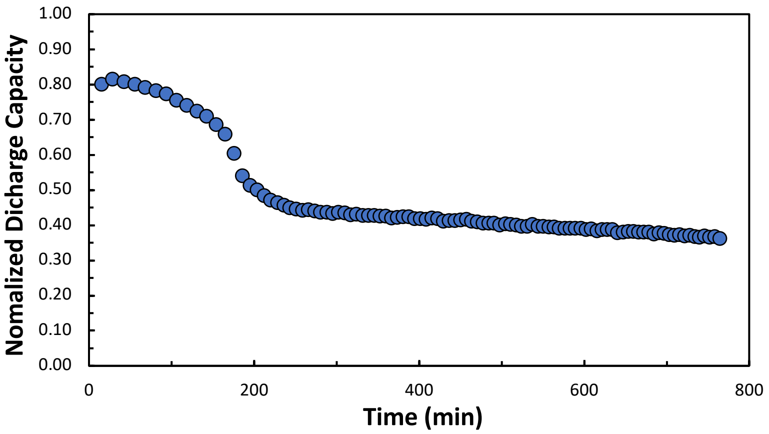

Figure SI-56: H-cell oxidative cycling (100 cycles) of **SQA-15** (5 mM) in 0.5 M $TBAFPF_6$ in MeCN at a current of 5 mA.

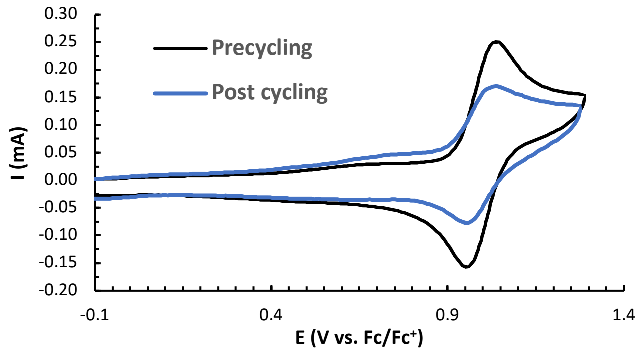

Figure SI-57: Cyclic voltammetry of **SQA-15** (5 mM) in 0.5 M $TBAFPF_6$ in MeCN at a scan rate of 500 mV/s before and after 100 H-cell charge-discharge cycles. Ratio of $I_{pa,3}/I_{pa,SQA}$ < 0.05 (unclear if any is present due to significant decomposition).

<u>3-(butyl(4-methoxyphenyl)amino)-4-ethoxycyclobut-3-ene-1,2-dione</u> (**SQA-16**)

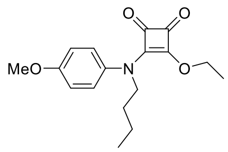

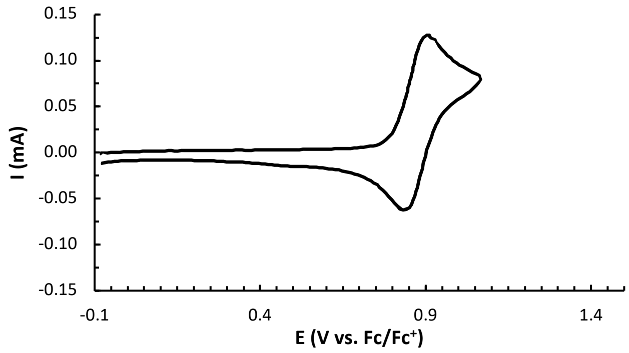

Figure SI-58: Cyclic voltammetry of **SQA-16** (5 mM) in 0.5 M $TBAFPF_6$ in MeCN at a scan rate of 100 mV/s.

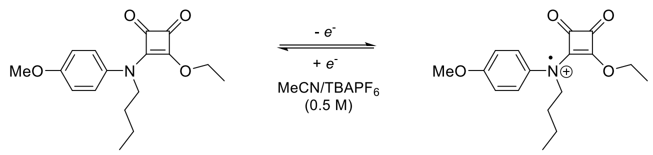

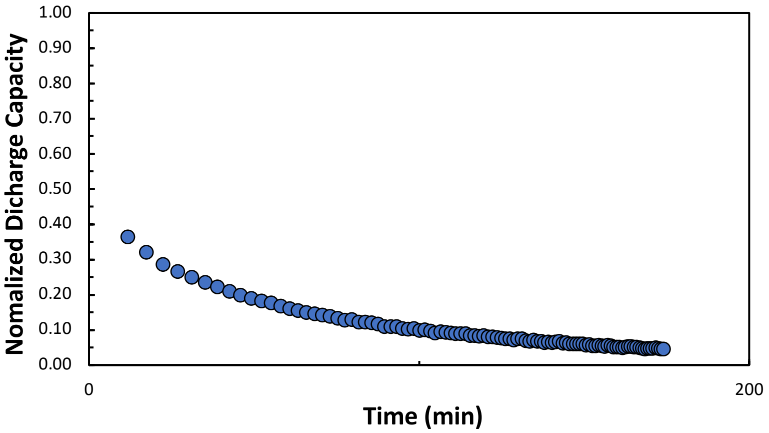

Figure SI-59: H-cell oxidative cycling (100 cycles) of **SQA-16** (5 mM) in 0.5 M TBAFPF$_6$ in MeCN at a current of 5 mA. Upper voltage cutoff 150 mV positive of $E_{1/2}$.

<u>3,4-bis(dibutylamino)cyclobut-3-ene-1,2-dione</u> (**SQA-17**)

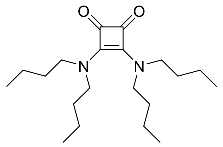

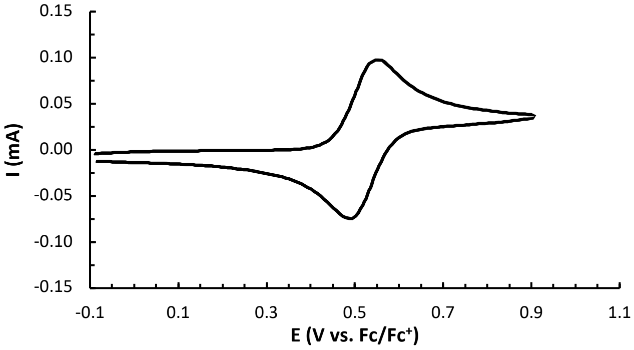

Figure SI-60: Cyclic voltammetry of **SQA-17** (5 mM) in 0.5 M TBAFPF$_6$ in MeCN at a scan rate of 100 mV/s.

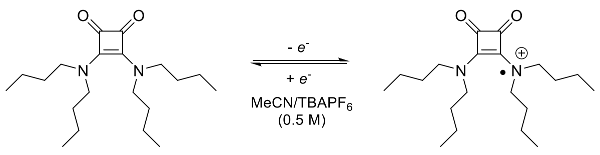

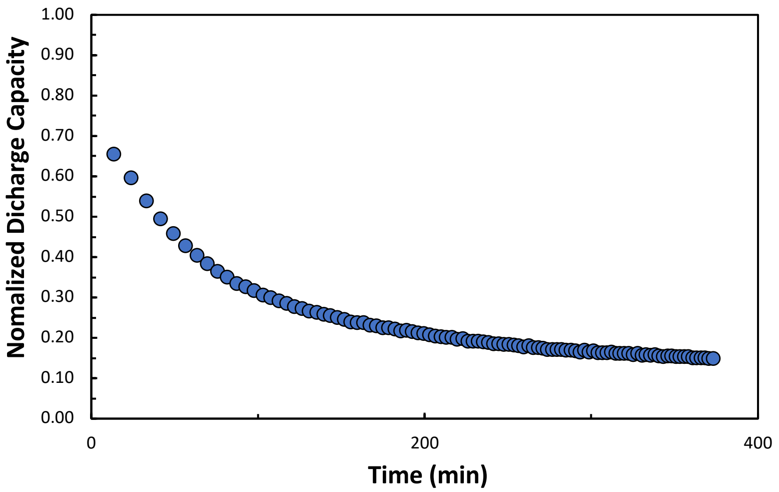

Figure SI-61: H-cell oxidative cycling (100 cycles) of **SQA-17** (5 mM) in 0.5 M $TBAFPF_6$ in MeCN at a current of 5 mA.

<u>bis(2-(2-methoxyethoxy)ethyl) 4,4'-((3,4-dioxocyclobut-1-ene-1,2-diyl)bis(methylazanediyl))dibenzoate</u> (**SQA-1**)

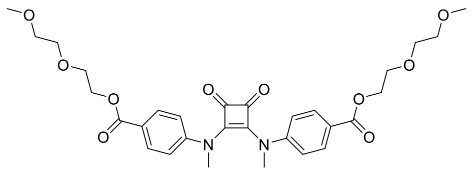

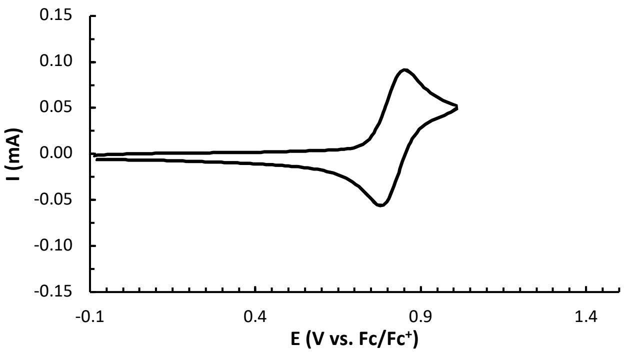

Figure SI-62: Cyclic voltammetry of **SQA-1** (5 mM) in 0.5 M $TBAFPF_6$ in MeCN at a scan rate of 100 mV/s.

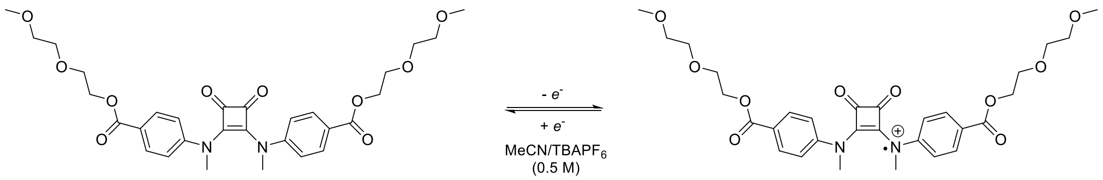

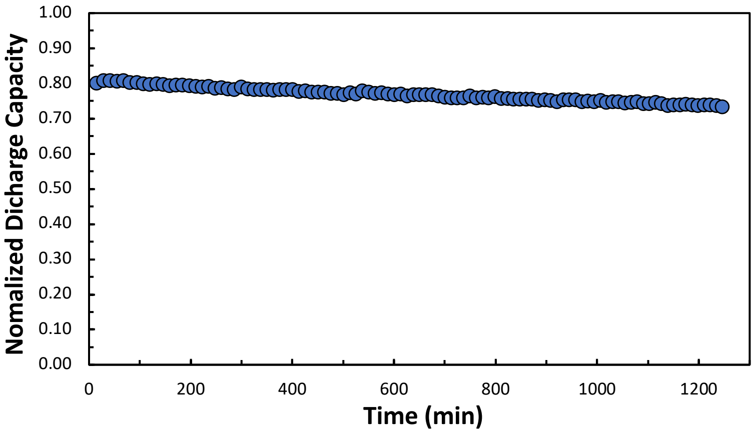

Figure SI-63: H-cell oxidative cycling (100 cycles) of **SQA-1** (5 mM) in 0.5 M $TBAFPF_6$ in MeCN at a current of 5 mA. Upper voltage cutoff 250 mV positive of $E_{1/2}$.

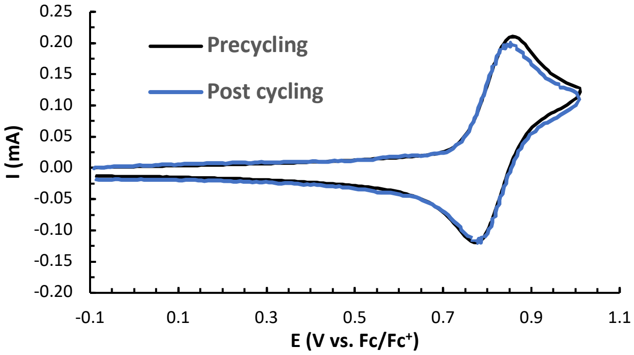

Figure SI-64: Cyclic voltammetry of **SQA-1** (5 mM) in 0.5 M $TBAFPF_6$ in MeCN at a scan rate of 500 mV/s before and after 100 H-cell charge-discharge cycles. $I_{pa,\mathbf{3}}/I_{pa,\mathbf{SQA}}$ = 0.04.

bis(2-(2-methoxyethoxy)ethyl) 1,1'-(3,4-dioxocyclobut-1-ene-1,2-diyl)bis(indoline-5-carboxylate) (**SQA-18**)

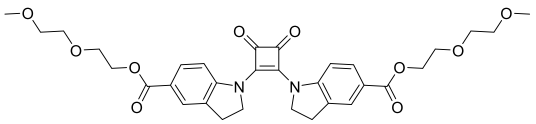

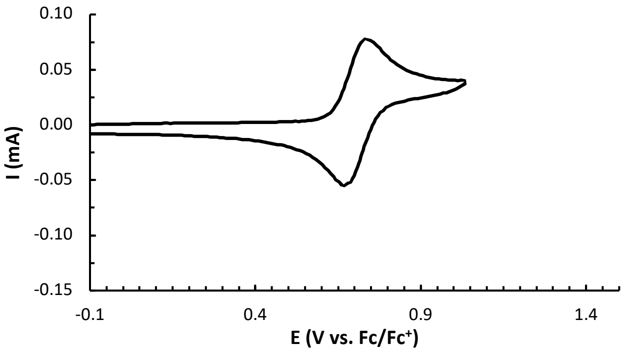

Figure SI-65: Cyclic voltammetry of **SQA-18** (5 mM) in 0.5 M TBAFPF$_6$ in MeCN at a scan rate of 100 mV/s.

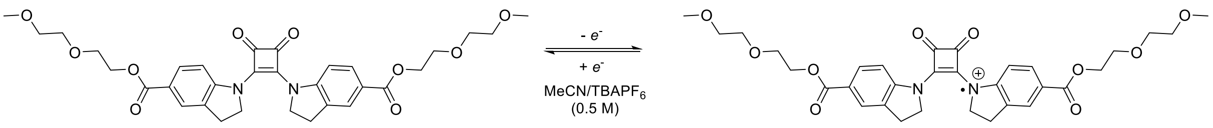

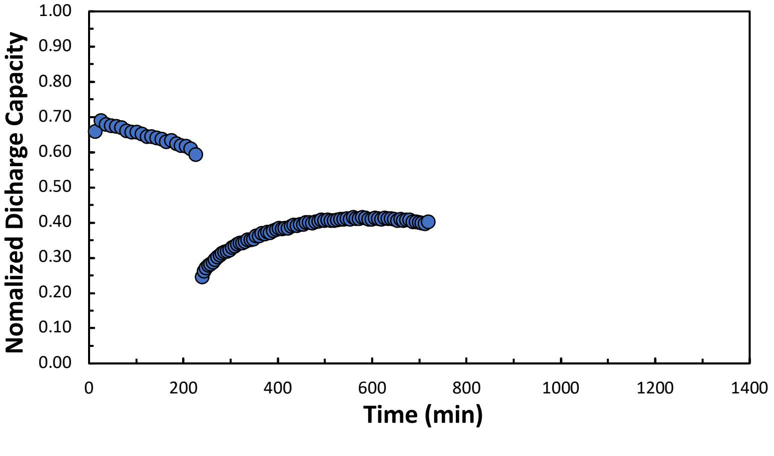

Figure SI-66: H-cell oxidative cycling (100 cycles) of **SQA-18** (5 mM) in 0.5 M TBAFPF$_6$ in MeCN at a current of 5 mA. Upper voltage cutoff 250 mV positive of $E_{1/2}$.

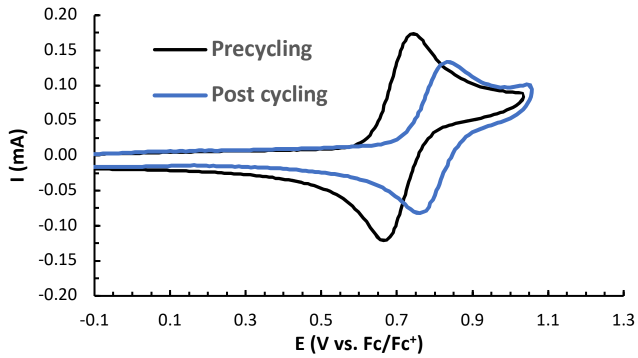

Figure SI-67: Cyclic voltammetry of **SQA-18** (5 mM) in 0.5 M TBAFPF$_6$ in MeCN at a scan rate of 500 mV/s before and after 100 H-cell charge-discharge cycles.

Based upon the pre- and post-cycling CVs, it was apparent that a new species with a more positive oxidation potential formed during the H-cell charge-discharge cycling. To identify this new species the following procedure was performed:

Post cycling analysis and characterization of biaryl dimer bis(2-(2-methoxyethoxy)ethyl) 1,2-dioxo-1,2,4,5,12,13-hexahydrocyclobuta[2,3][1,4]diazocino[6,5,4-hi:7,8,1-h'i']diindole-7,10-dicarboxylate (4): After 100 H-cell charge-discharge cycles ending on a complete discharge, the material on the working side of the H-cell was removed from the glovebox and diluted with 100 mL of diethyl ether. The resulting precipitated TBAPF$_6$ was removed via vacuum filtration and the crude filtrate was concentrated under reduced pressure. A crude ^{1}H NMR was then obtained in CDCl$_3$ after which the major product was isolated via preparatory TLC (100% ethyl acetate then 5% methanol in ethyl acetate). Analysis of the resulting ^{1}H NMR, ^{13}C NMR, and HRMS data allowed us to assign the major component of the crude reaction mixture to likely be the intramolecular biaryl coupling product of the original squaramide catholyte (see below). The formation of this species is likely responsible for the sudden decline in battery capacity that is observed after around 225 minutes and can be thought to formally arise from intramolecular biaryl coupling of the radical cation followed by lose of H$_2$ and addition of an electron.

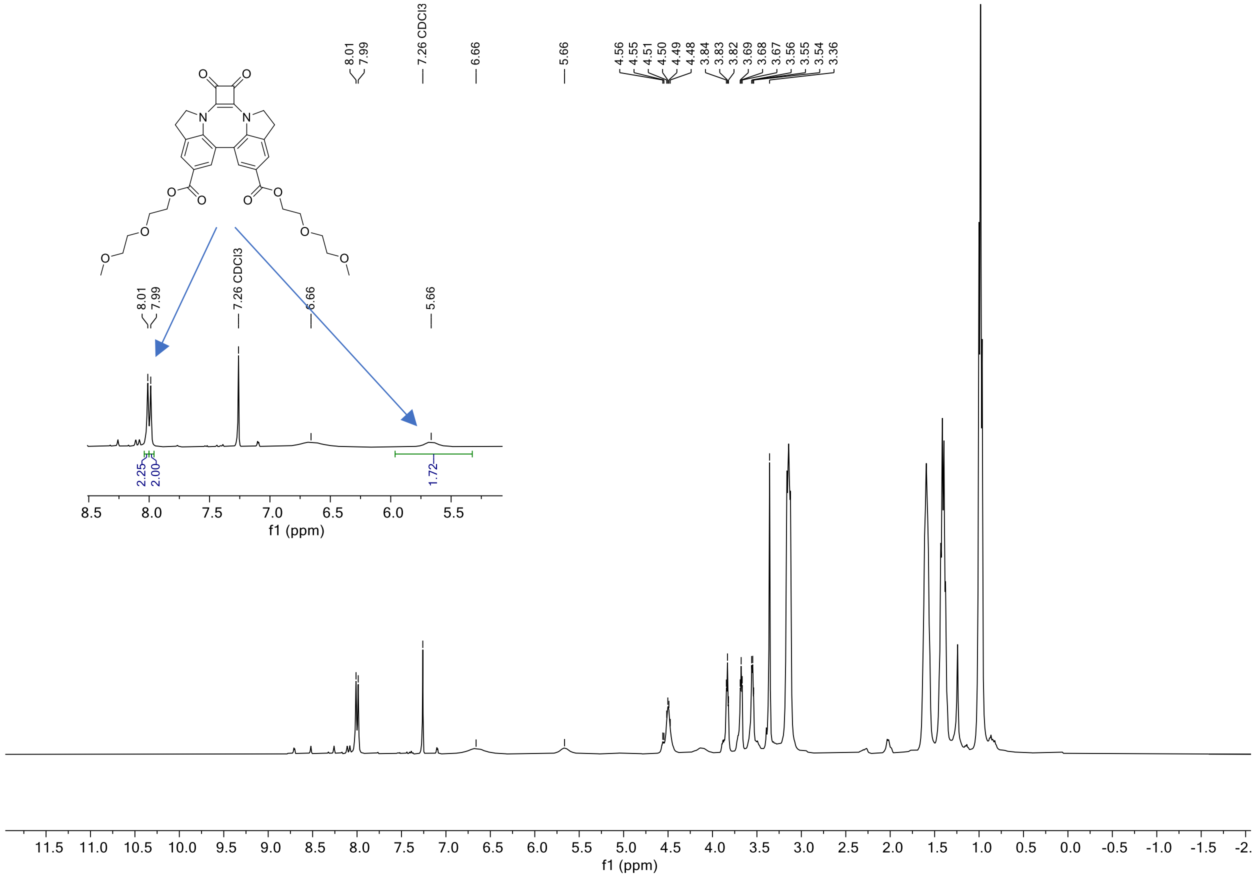

Figure SI-68: Crude NMR of the working side of the H-cell following H-cell oxidative cycling of **SQA-18** (5 mM) in 0.5 M TBAFPF$_6$ in MeCN at a current of 5 mA.

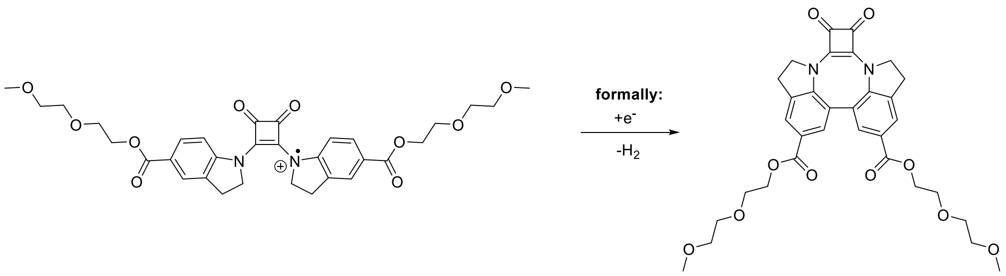

^{1}H NMR (600 MHz, CDCl$_3$) δ 8.02 (d, J = 1.6 Hz, 2H), 8.00 (d, J = 1.7 Hz, 2H), 5.68 (s, 2H), 4.50 (s, 4H), 4.12 (s, 2H), 3.84 (t, J = 4.9 Hz, 4H), 3.70 – 3.66 (m, 4H), 3.58 – 3.53 (m, 4H), 3.36 (s, 6H). ^{13}C NMR (151 MHz, CDCl$_3$) δ 184.7, 165.6, 163.9, 140.6, 135.8, 133.9, 126.6, 126.0, 122.5, 72.1, 70.7, 69.4, 64.5, 59.2, 50.7, 29.3. R$_f$ (100% ethyl acetate) = 0.31. HRMS (ESI) m/z calculated for $C_{32}H_{34}N_2O_{10}$ (H+): 607.2286, found: 607.2291.

<u>3,4-bis(butyl(4-(methylthio)phenyl)amino)cyclobut-3-ene-1,2-dione</u> (**SQA-19**)

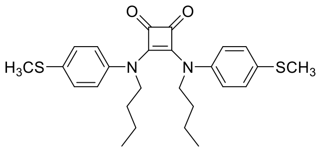

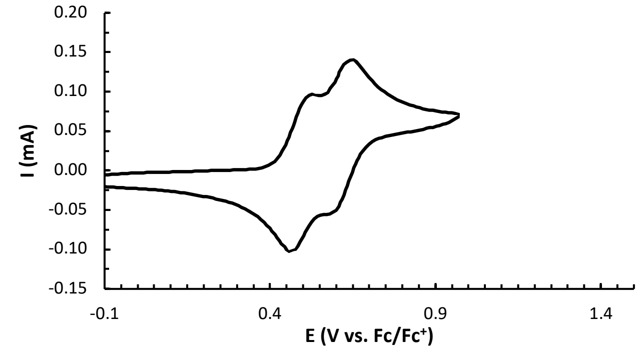

Figure SI-69: Cyclic voltammetry of **SQA-19** (5 mM) in 0.5 M $TBAFPF_6$ in MeCN at a scan rate of 100 mV/s.

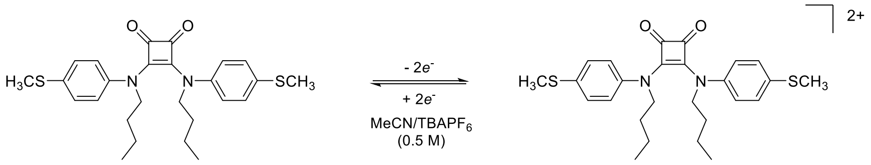

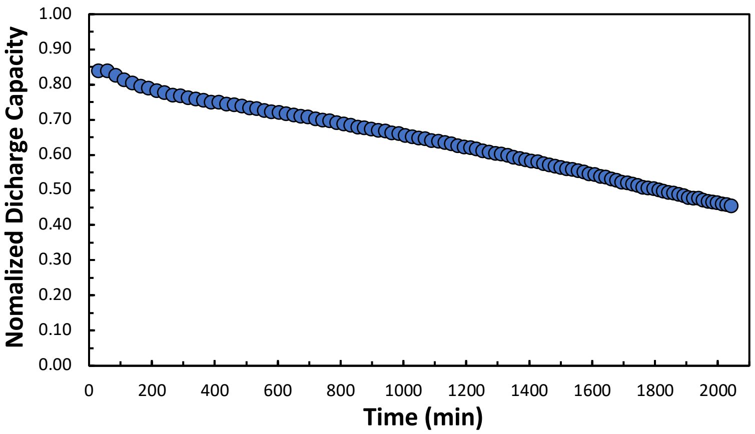

Figure SI-70: H-cell two-electron oxidative cycling (100 cycles) of **SQA-19** (5 mM) in 0.5 M $TBAFPF_6$ in MeCN at a current of 5 mA.

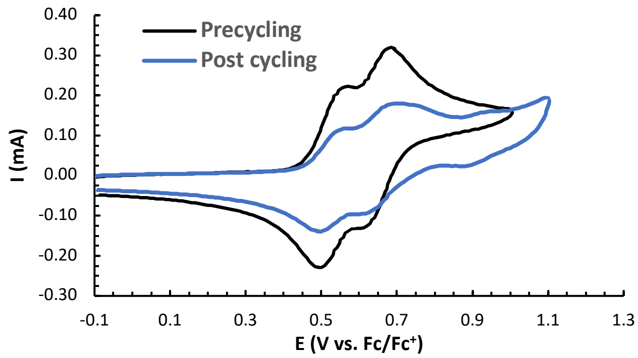

Figure SI-71: Cyclic voltammetry of **SQA-19** (5 mM) in 0.5 M TBAFPF$_6$ in MeCN at a scan rate of 500 mV/s before and after 100 H-cell charge-discharge cycles.

<u>3,4-bis(butyl(4-methoxyphenyl)amino)cyclobut-3-ene-1,2-dione</u> (**SQA-2**)

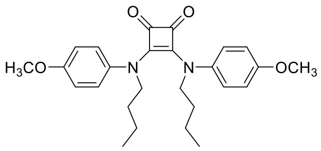

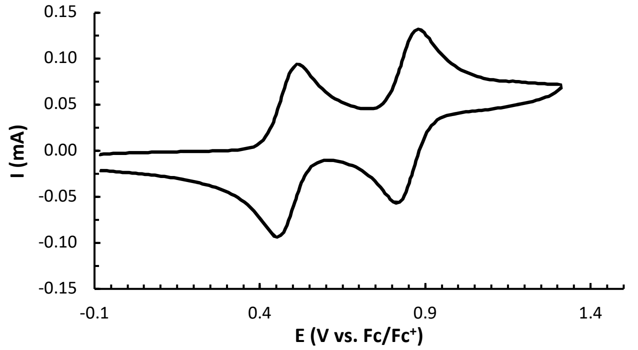

Figure SI-72: Cyclic voltammetry of **SQA-2** (5 mM) in 0.5 M TBAFPF$_6$ in MeCN at a scan rate of 100 mV/s.

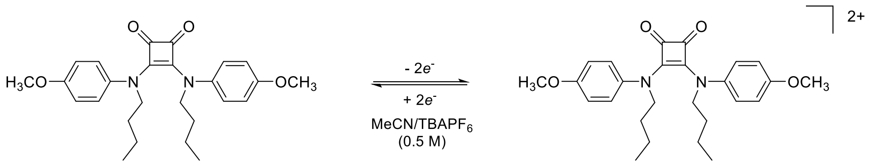

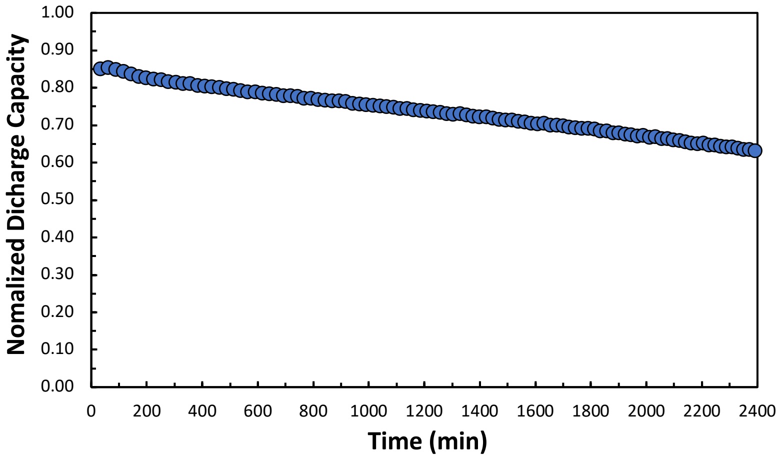

Figure SI-73: H-cell two-electron oxidative cycling (100 cycles) of **SQA-2** (5 mM) in 0.5 M $TBAFPF_6$ in MeCN at a current of 5 mA. Upper voltage cutoff 320 mV positive of $E_{1/2}^{2}$.

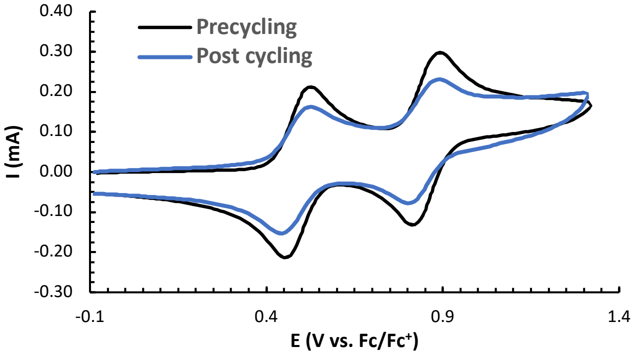

Figure SI-74: Cyclic voltammetry of **SQA-2** (5 mM) in 0.5 M $TBAFPF_6$ in MeCN at a scan rate of 500 mV/s before and after 100 H-cell charge-discharge cycles.

3,4-bis(butyl(4-isopropoxyphenyl)amino)cyclobut-3-ene-1,2-dione (**SQA-20**)

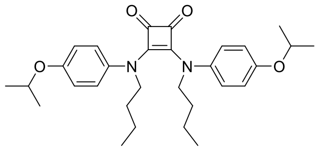

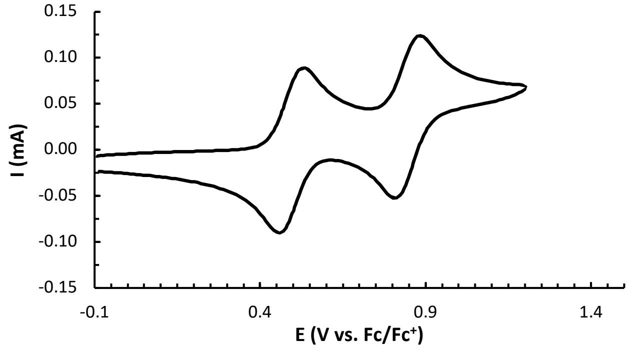

Figure SI-75: Cyclic voltammetry of **SQA-20** (5 mM) in 0.5 M $TBAFPF_6$ in MeCN at a scan rate of 100 mV/s.

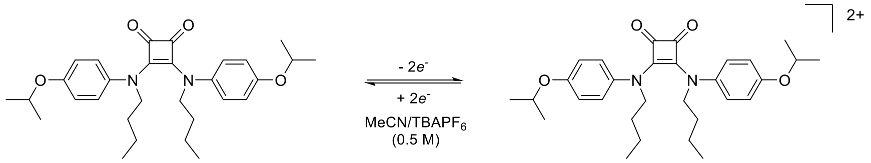

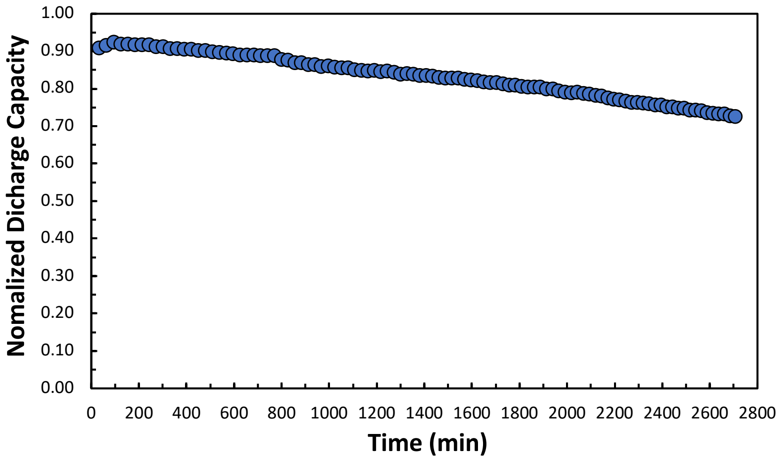

Figure SI-76: H-cell two-electron oxidative cycling (100 cycles) of **SQA-20** (5 mM) in 0.5 M $TBAFPF_6$ in MeCN at a current of 5 mA. Upper voltage cutoff 300 mV positive of $E_{1/2}^2$.

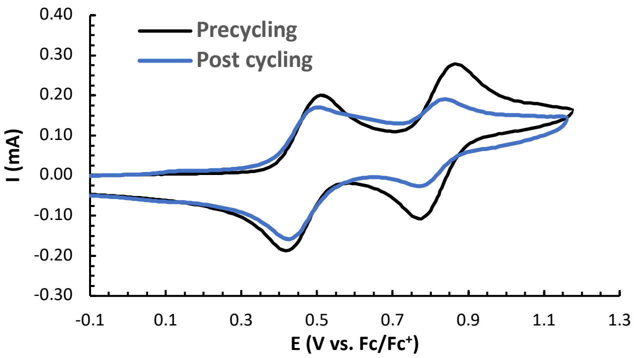

Figure SI-77: Cyclic voltammetry of **SQA-20** (5 mM) in 0.5 M TBAFPF$_6$ in MeCN at a scan rate of 500 mV/s before and after 100 H-cell charge-discharge cycles.

H-Cell Anolyte/Catholyte Compatibility Studies: As part of our efforts to test the new classes of squaramide catholytes in bench-scale flow cells, we decided to pursue a 50:50 mixed catholyte:anolyte design that helps to minimize the effects of crossover by eliminating any concentration gradients when the battery is in the fully discharged state. However, this type of flow cell requires that the catholyte and anolyte be stable in the presence of each other across all possible states of charge. In order to find a suitable anolyte that meets such conditions with our catholytes, we employed H-cell charge-discharge cycling experiments as follows:

Bulk charge/discharge measurements were carried out in a nitrogen-filled glovebox with a CH Instruments 760 Bipotentiostat in a custom H-cell (pictured below) with a fritted glass separator (P5). The working and counter electrodes were carbon (Duocel® RVC Foam, 100 PPI, 3% relative density). An Ag/Ag^{+} quasi-reference electrode (described above) was used on the working side of the H-cell. A 50:50 mixture of the corresponding catholyte and anolyte was dissolved in 0.5 M TBAPF$_6$ in acetonitrile to give a concentration of 5 mM for both species. The working chamber of the H-cell was first loaded with 5 mL of the electrolyte/ROM solution while the counter chamber was loaded with 5 mL of only 0.5 M TBAPF$_6$ in acetonitrile. The working chamber then underwent oxidative charging to form the radical cation of the catholyte while the anolyte remained a neutral observer. At this point, the solution was removed from the counter chamber and replaced with 5 mL of the electrolyte/50:50 ROM solution. A discharge event was then conducted followed by 99 more charge-discharge cycles. Charging and discharging were all conducted with a current of 5 mA and both chambers of the H-cell were continuously stirred with magnetic stir bars. The upper and lower voltage cutoffs, unless otherwise indicated, were 0.4 V plus and minus the $E_{1/2}$ (determined with CV at a scan rate of 100 mV/s or 500 mV/s). Following the final discharge, CV analysis was performed to ensure compatibility of both the anolyte and catholyte. The above procedure was then repeated, but with a reductive first charging event to form the reduced anolyte while the catholyte served as a neutral observer. A total of 100 charge-discharge cycles were then performed by following an analogous procedure, before the same post cycling analysis was performed. Results of H-cell bulk electrolysis are displayed here in graphs of normalized discharge capacity (normalized relative to theoretical capacity) vs time or cycle number for 100 (unless otherwise noted) charge-discharge cycles. Each data point in these figures represents one cycle.

Squaramide **SQX-1** and Pyridinium **1**$^{+}$ oxidative charge-discharge cycling (JST-III-58 and CHB-BK-46).

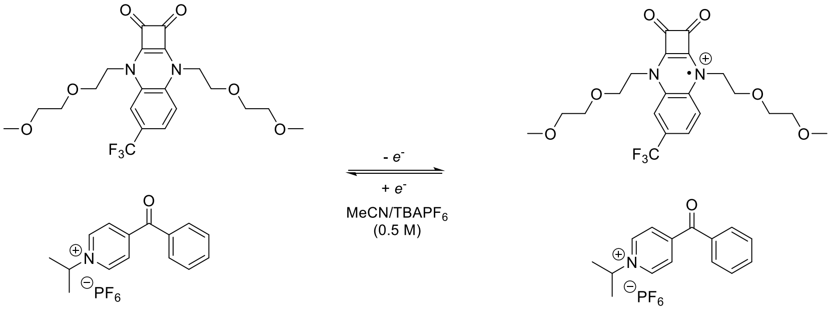

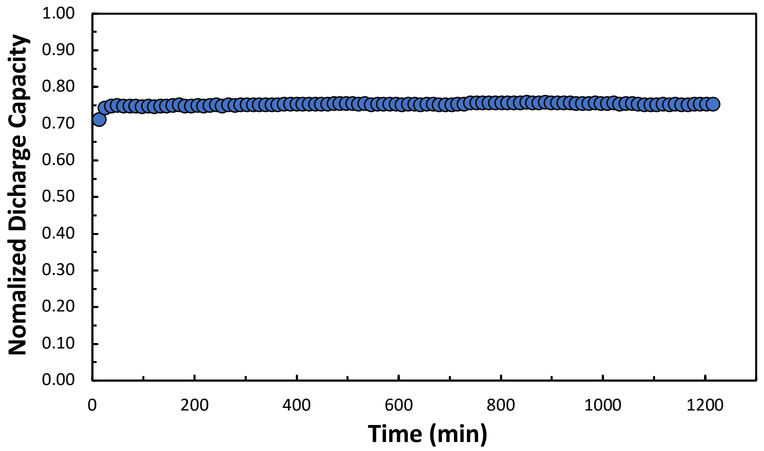

Figure SI-78: H-cell oxidative cycling (100 cycles) of **SQX-1** (5 mM) and **1**$^{+}$ (5 mM) in 0.5 M TBAFPF$_6$ in MeCN at a current of 5 mA.

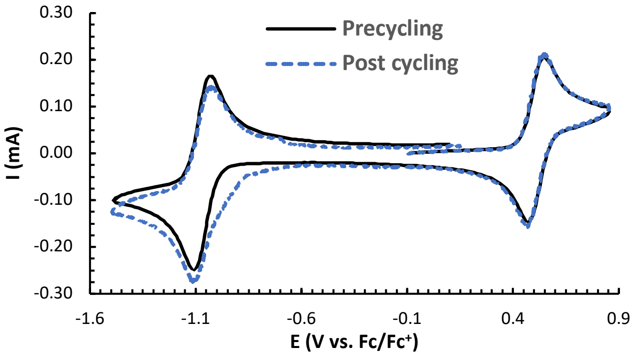

Figure SI-79: Cyclic voltammetry **SQX-1** (5 mM) and **1**$^{+}$ (5 mM) in 0.5 M TBAFPF$_6$ in MeCN at a scan rate of 500 mV/s before and after 100 H-cell oxidative charge-discharge cycles.

Squaramide **SQX-1** and Pyridinium **1**$^+$ reductive charge-discharge cycling

Please note that the pyridinium was used as a one-electron anolyte and therefore only its first reduction was considered in this analysis.

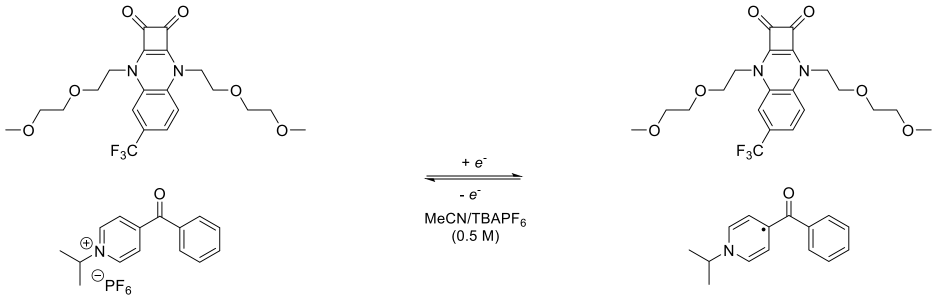

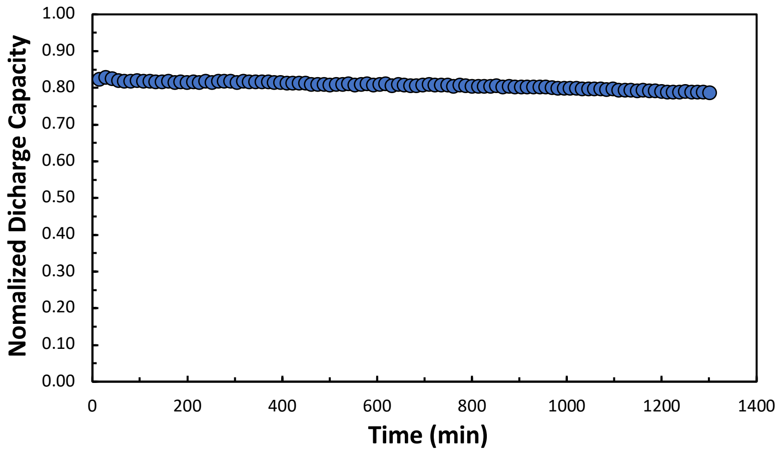

Figure SI-80: H-cell reductive cycling (100 cycles) of **SQX-1** (5 mM) and **1**$^+$ (5 mM) in 0.5 M $TBAFPF_6$ in MeCN at a current of 5 mA.

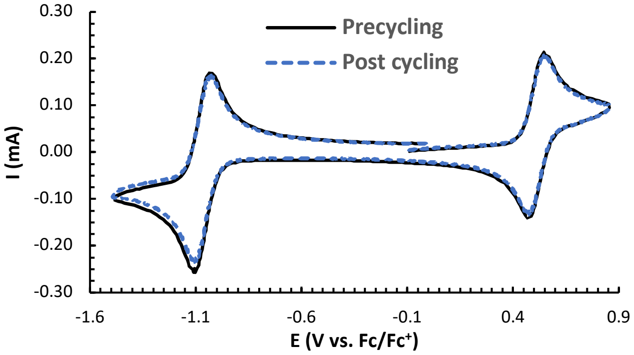

Figure SI-81: Cyclic voltammetry of **SQX-1** (5 mM) and **1**$^+$ (5 mM) in 0.5 M $TBAFPF_6$ in MeCN at a scan rate of 500 mV/s before and after 100 H-cell reductive charge-discharge cycles.

Squaramide **SQA-1** and viologen $\mathbf{5^{2+}}$ oxidative charge-discharge cycling

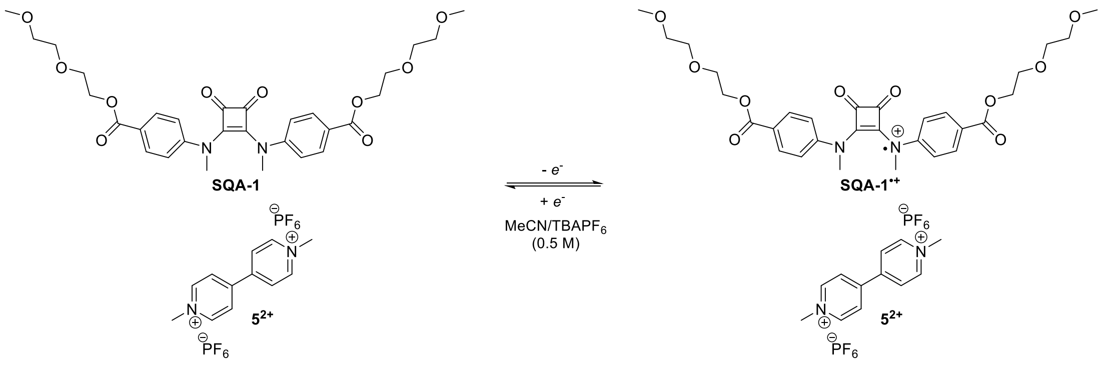

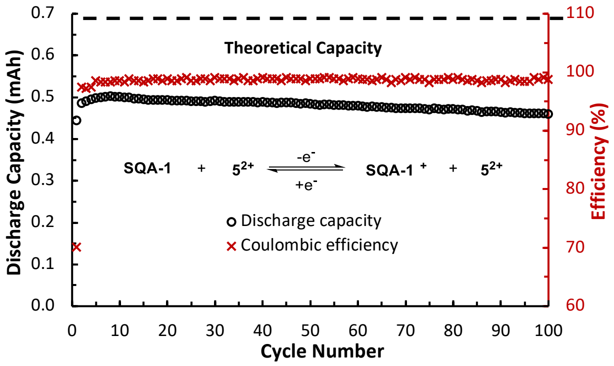

Figure SI-83: H-cell oxidative cycling (100 cycles) of **SQA-1** (5 mM) and $\mathbf{5^{2+}}$ (5 mM) in 0.5 M $TBAFPF_6$ in MeCN at a current of 5 mA.

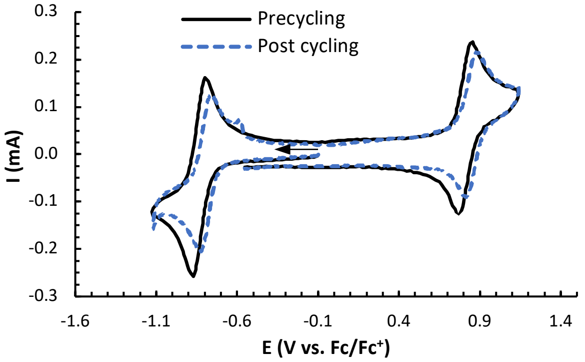

Figure SI-84: Cyclic voltammetry of **SQA-1** (5 mM) and $\mathbf{5^{2+}}$ (5 mM) in 0.5 M $TBAFPF_6$ in MeCN at a scan rate of 500 mV/s before and after 100 H-cell oxidative charge-discharge cycles.

Squaramide **SQA-1** and viologen **5**$^{2+}$ reductive charge-discharge cycling

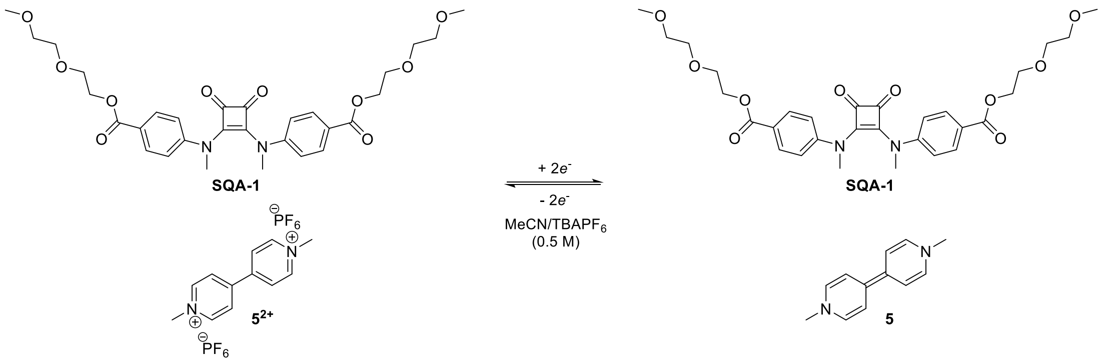

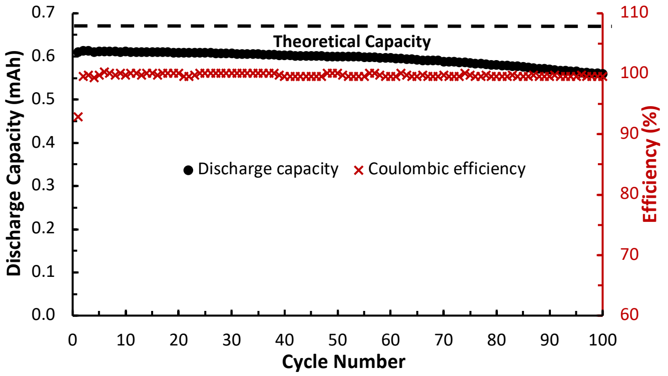

Figure SI-85: Two-electron H-cell reductive cycling (100 cycles) of **SQA-1** (5 mM) and **5**$^{2+}$ (2.5 mM) in 0.5 M $TBAFPF_6$ in MeCN at a current of 5 mA.

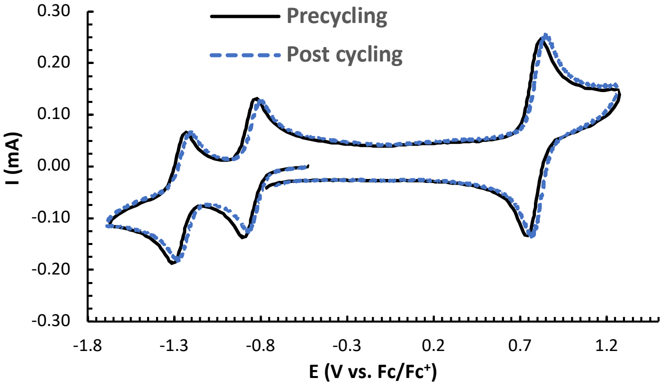

Figure SI-86: Cyclic voltammetry of **SQA-1** (5 mM) and **5**$^{2+}$ (2.5 mM) in 0.5 M $TBAFPF_6$ in MeCN at a scan rate of 500 mV/s before and after 100 H-cell two-electron reductive charge-discharge cycles.

Diffusion Coefficients and Heterogeneous Electron-Transfer Rates:

Diffusion coefficients were calculated from the slopes of peak height vs square root of scan rate and the Randles-Ševčík equation (*equation 1*) before being averaged to a single value.

$$i_p = 0.4463nFAC\left(\frac{nF\nu D}{RT}\right)^{1/2} \qquad (equation\ 1)$$

Where i_p is the peak current (A), n is the number of electrons transferred, A is the active area of the electrode (cm^2), C is the concenrtation in mol/cm^3, F is Faraday's constant (C/mol), ν is scan rate (V/s), D is the diffusion coefficient we are interested in calculating (cm^2/s), R is the ideal gas constant ($JK^{-1}mol^{-1}$), and T is temperature (K).

Heterogeneous electron-transfer rates were then determined based upon the diffusion coefficient as determined by the Nicholson method.[10,11]

V. References

[1] Niu, H.; Shu, Q.; Jin, S.; Li, B.; Zhu J.; Li, L.; Chen, S "A simple ratiometric and colorimetric chemosensor for the selective detection of fluoride in DMSO buffered solution" *Spectrochim. Acta A Mol. Biomol. Spectrosc.* **2016**, *153*, 194-198.

[2] Schaufelberger, F.; Seigel, K.; Ramström, O. "Hydrogen-Bond Catalysis of Imine Exchange in Dynamic Covalent Systems" *Chem. Eur. J.* **2020**, *26*, 15581-15588.

[3] Busschaert, N.; Kirby, I. L.; Young, S.; Coles, S. J.; Horton, P. N.; Light, M. E.; Gale, P. A. "Squaramides as Potent Transmembrane Anion Transporters" *Angew. Chem. Int. Ed.* **2012**, *51*, 4426-4430.

[4] Rostami, A.; Colin, A.; Li, X. Y.; Chudzinksi, M. G.; Lough, A. J.; Taylor, M. S. "*N,N*'-Diarylsquaramides: General, High-Yielding Synthesis and Applications in Colorimetric Anion Sensing" *J. Org. Chem.* **2010**, *75*, 3983-3992.

[5] Amendola, V.; Bergamaschi, G.; Boiocchi, M.; Fabbrizzi, L.; Milani, M. "The Squaramide versus Urea Contest for Anion Recognition" *Chem. Eur. J.* **2010**, *16*, 4368-4380.

[6] Long, N.; Le Gresley, A.; Solomonsz, A.; Wozniak, A.; Brough, S.; Wren, S. P. "Synthesis of Squaric Acid Monoamides as Building Blocks for Drug Discovery" *SynOpen* **2023**, *7*, 401-407.

[7] Sevov, C. S.; Samaroo, S. K.; Sanford, M. S. "Cyclopropenium Salts as Cyclable, High-Potential Catholytes in Nonaqueous Media" *Adv. Energy Mat.,* **2017**, *7*, 1602027.

[8] Kowalki, J. A.; Neyhouse B. J.; and Brushett, F. R. "The impact of bulk electrolysis cycling conditions on the perceived stability of redox active materials" *Electrochem. Commun.,* **2020**, *111*, 106625.

[9] Kowalki, J. A; Casselman, M. D.; Kaur, A. P.; Milshtein, J. D.; Elliott, C. F.; Modekrutti, S.; Attanayake, N. H.; Zhang, N.; Parkin, S. R.; Risko, C.; Brushett, F. R.; Odom, S. A. "A stable two-electron-donating phenothiazine for application in nonaqueous redox flow batteries" *J. Mater.*

Chem. A, **2017**, *5*, 24371-24379. (*b*) Milshtein, J. D.; Barton, J. L.; Darling R. M.; Brushett, F. R. “4-acetamido-2,2,6,6-tetramethylpiperidine-1-oxyl as a model organic redox active compound for nonaqueous flow batteries” *J. Power Sources,* **2016**, *327*, 151-159. (*c*) Milshtein, J. D.; Tenny, K. M.; Barton, J. L.; Drake, J.; Darling, R. M.; Brushett, F. R. “Quantifying Mass Transfer Rates in Redox Flow Batteries” *J. Electrochem. Soc.,* **2017**, *164*, E3265-E3275. (*d*) Milshtein, J. D.; Fisher, E. L.; Breault, T. M.; Thompson, L. T.; Brushett, F. R. “Feasibility of a Supporting-Salt-Free Nonaqueous Redox Flow Battery Utilizing Ionic Active Materials” *ChemSusChem*, **2017**, *10*, 2080-2088. (*e*) Milshtein, J. D.; Barton, J. L.; Carney, T. J.; Kowalski, J. A.; Darling, R. M.; Brushett, F. R. “Towards Low Resistance Nonaqueous Redox Flow Batteries” *J. Electrochem. Soc.,* **2017**, *164*, A2487-A2499.

[10] Nicholson, R. S. "Theory and Application of Cyclic Voltammetry for Measurement of Electrode Reaction Kinetics" *Anal. Chem.* **1965**, *37*, 1351–1355.

[11] Lavagnini, I.; Antiochia, R.; Magno, F. "An Extended Method for the Practical Evaluation of the Standard Rate Constant from Cyclic Voltammetric Data" *Electroanalysis* **2004**, *16*, 505–506.

VI. NMR Spectra

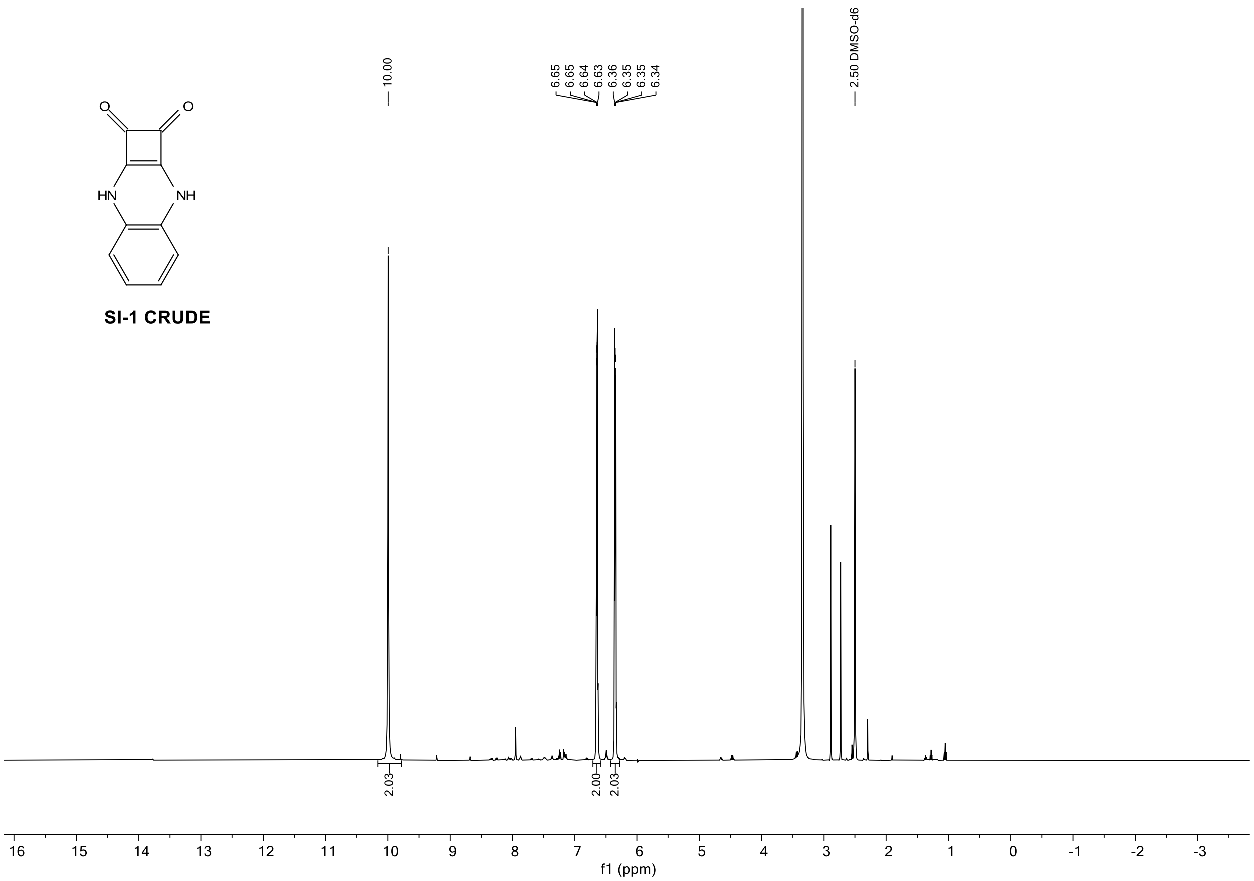

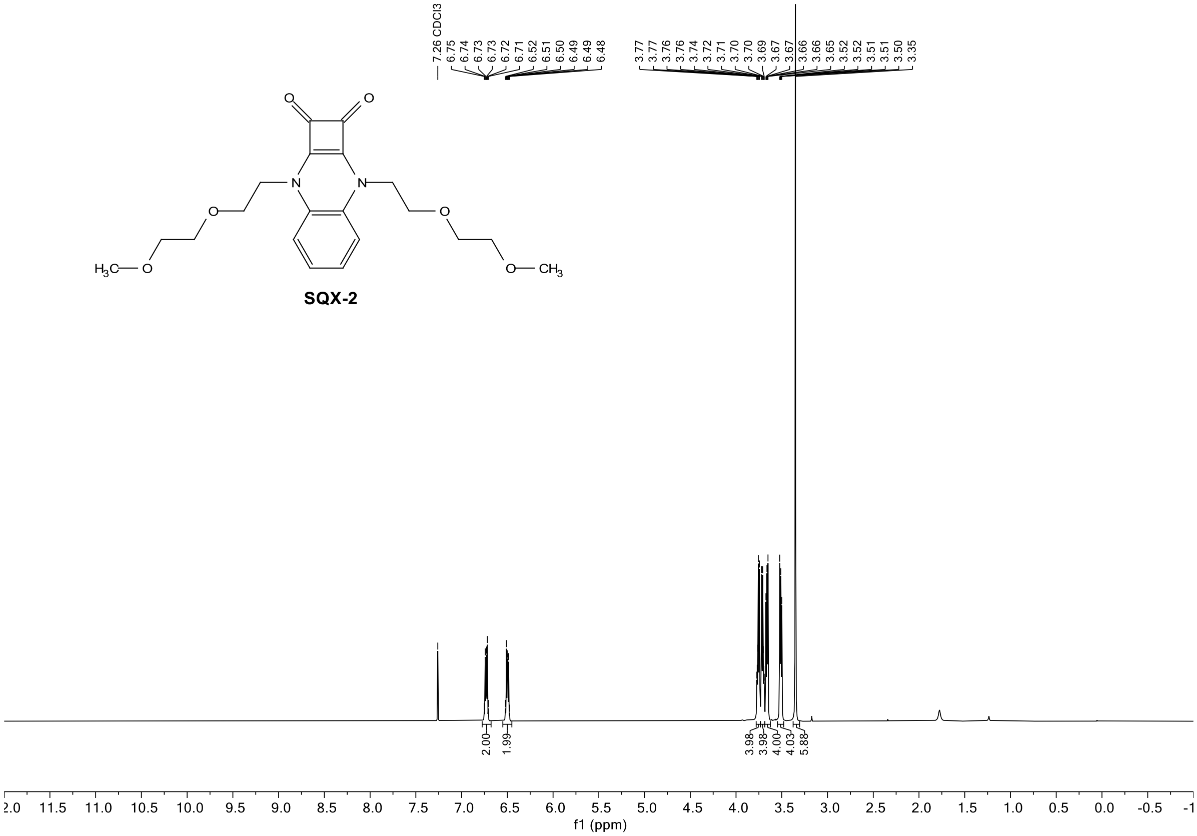
SQX-2
7.26 CDCl3
6.75
6.74
6.73
6.73
6.72
6.71
6.52
6.51
6.50
6.49
6.49
6.48
3.77
3.77
3.76
3.76
3.74
3.72
3.71
3.70
3.70
3.69
3.67
3.67
3.66
3.66
3.65
3.52
3.52
3.51
3.51
3.50
3.35
2.00
1.99
3.98
3.98
4.00
4.03
5.88
f1 (ppm)

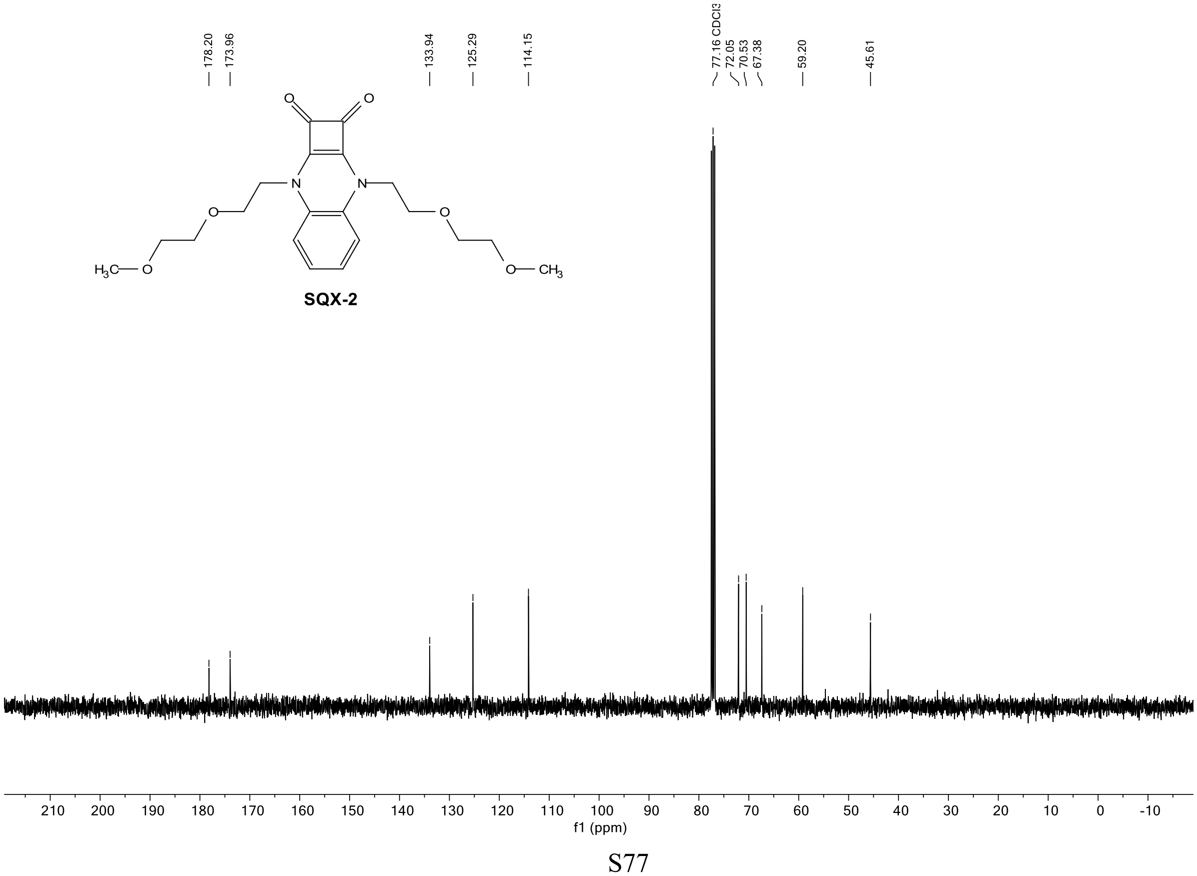
SQX-2
178.20
173.96
133.94
125.29
114.15
77.16 CDCl3
72.05
70.53
67.38
59.20
45.61
f1 (ppm)

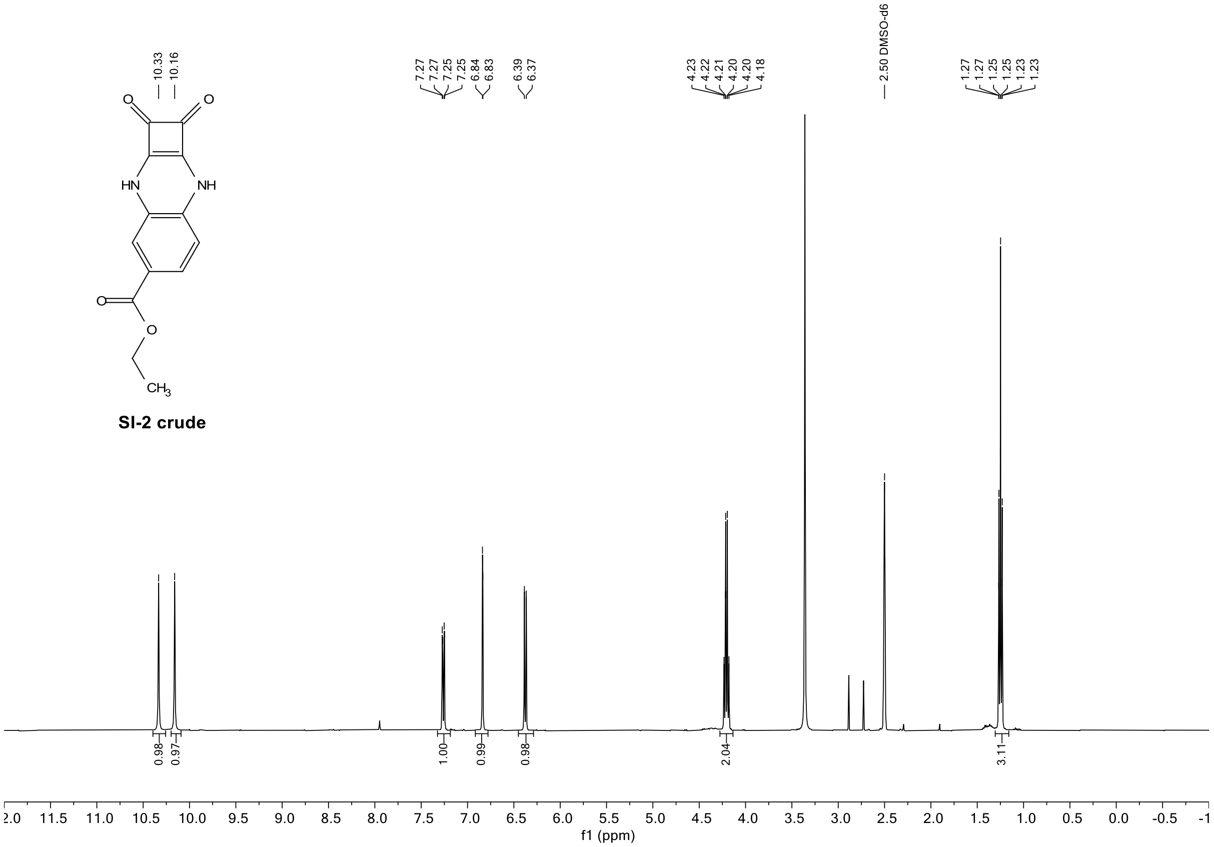
10.33
10.16
7.27
7.27
7.25
7.25
6.84
6.83
6.39
6.37
4.23
4.22
4.21
4.20
4.20
4.18
2.50 DMSO-d6
1.27
1.27
1.25
1.25
1.23
1.23
0.98
0.97
1.00
0.99
0.98
2.04
3.11
f1 (ppm)

SI-2 crude

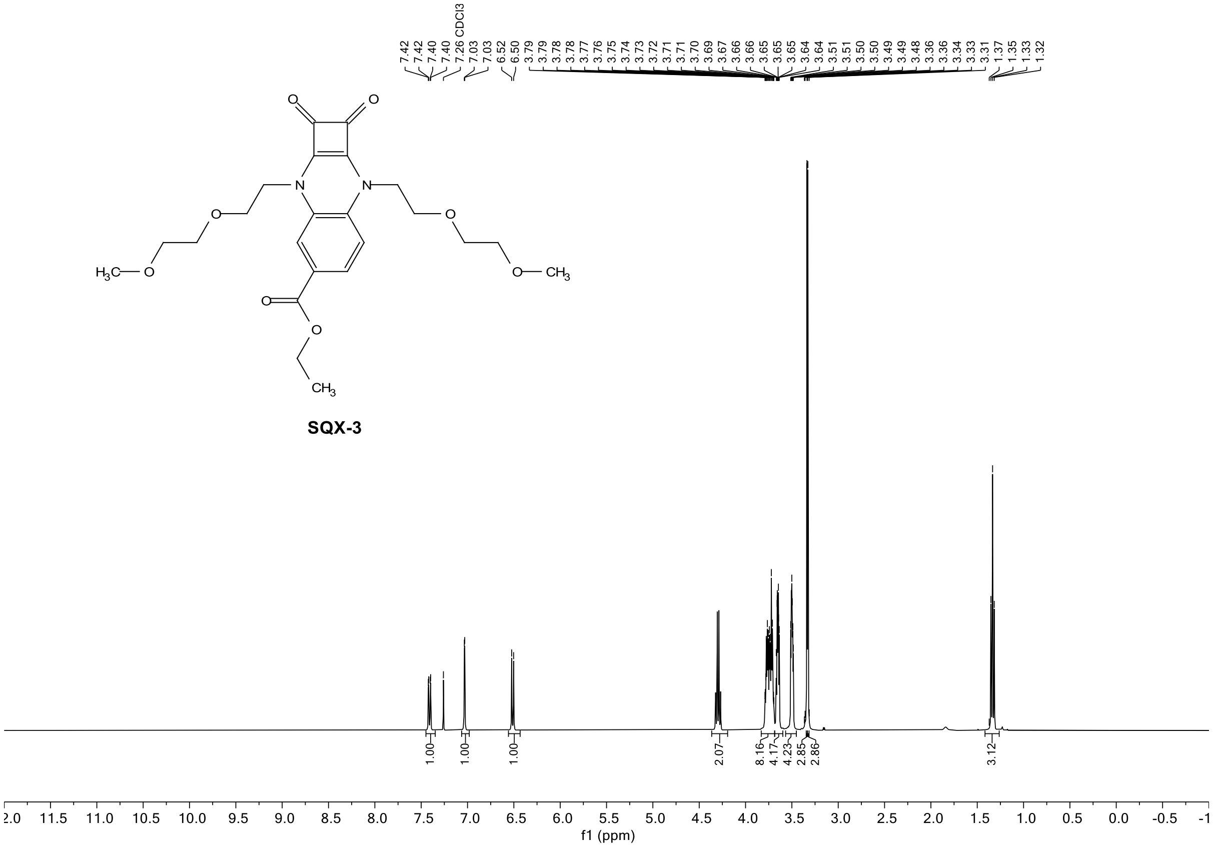
SQX-3
f1 (ppm)

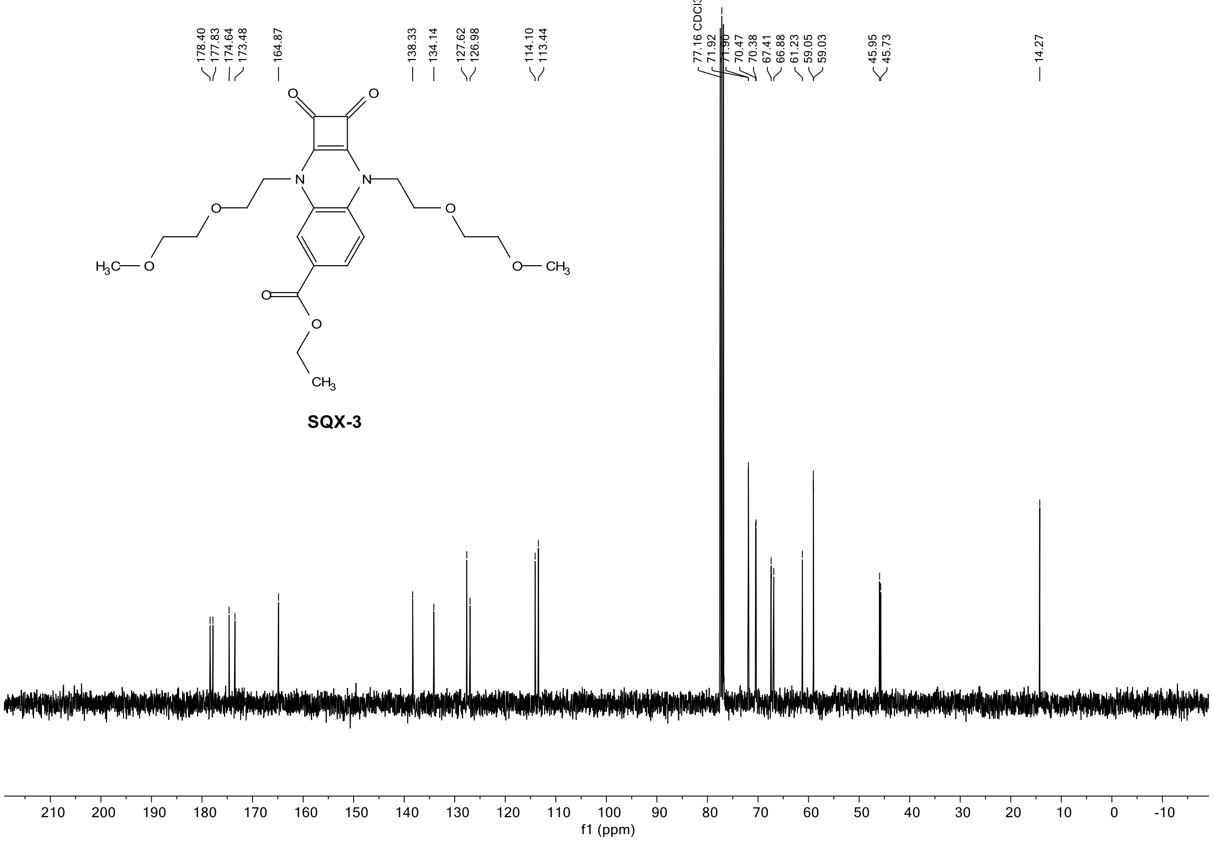
SQX-3
f1 (ppm)

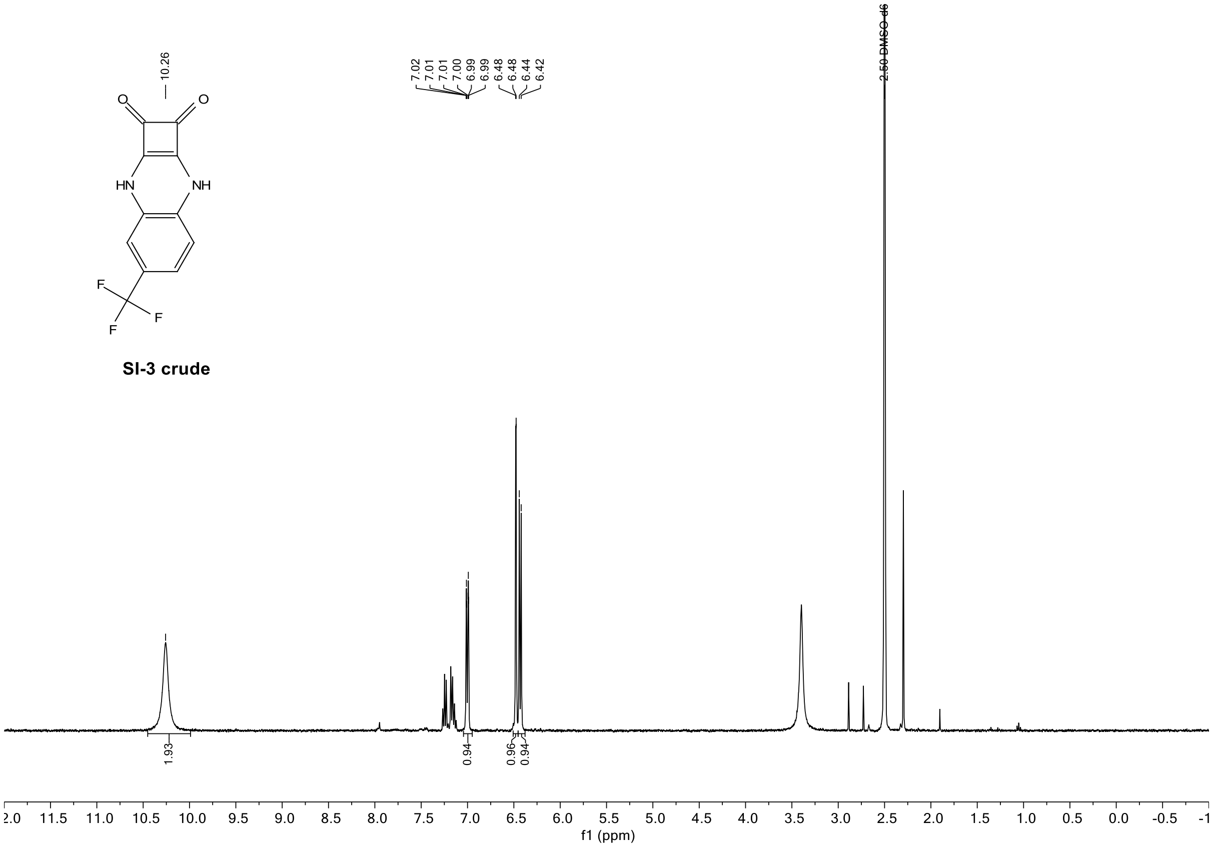
10.26
7.02
7.01
7.01
7.00
6.99
6.99
6.48
6.48
6.44
6.42
2.50 DMSO-d6
1.93
0.94
0.96
0.94
f1 (ppm)

SI-3 crude

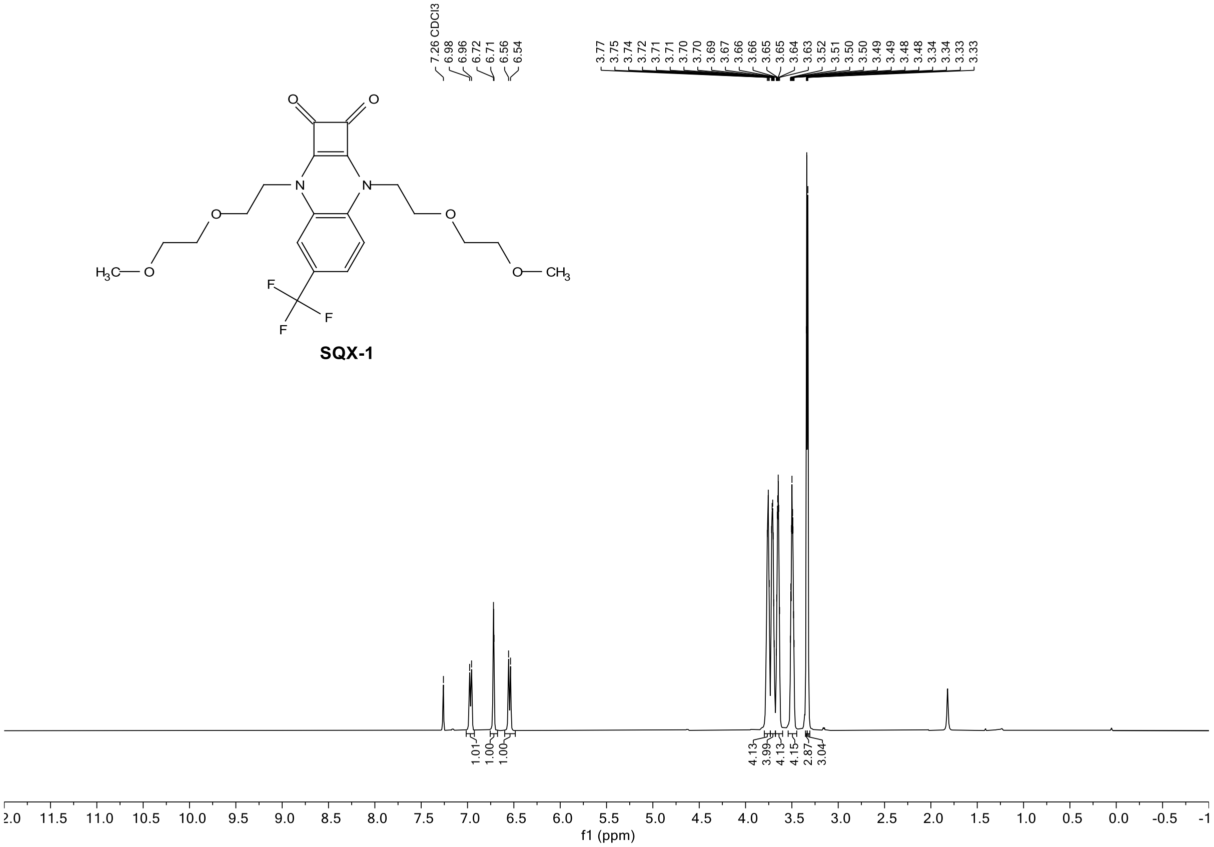
7.26 CDCl3
6.98
6.96
6.72
6.71
6.56
6.54
3.77
3.75
3.74
3.72
3.71
3.71
3.70
3.69
3.67
3.66
3.65
3.65
3.64
3.63
3.52
3.51
3.50
3.50
3.49
3.49
3.48
3.34
3.34
3.33
3.33
H3C—O
O—CH3
F
F
F
N
N
O
O
O
O
SQX-1
1.01
1.00
1.00
4.13
3.99
4.13
4.15
2.87
3.04
f1 (ppm)

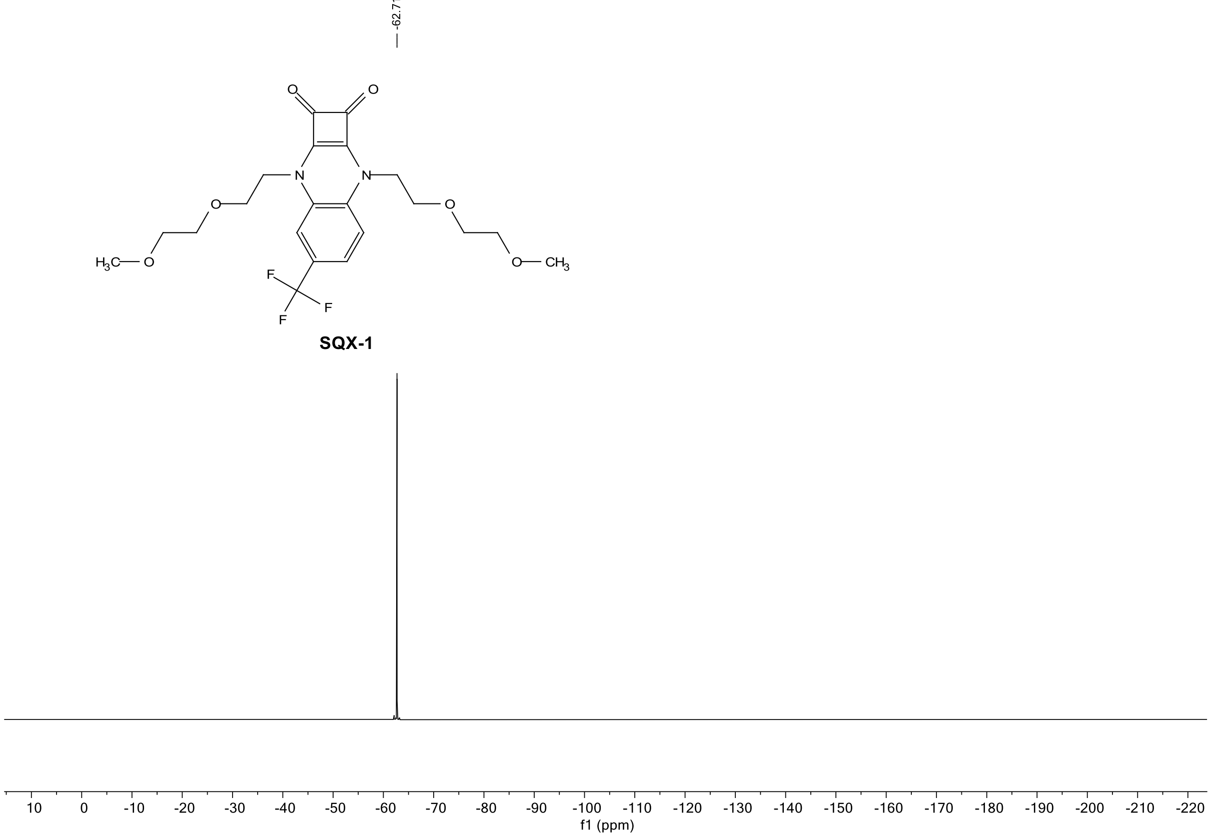
-62.71
H3C—O
O—CH3
F
F
F
N
N
O
O
O
O
SQX-1
f1 (ppm)

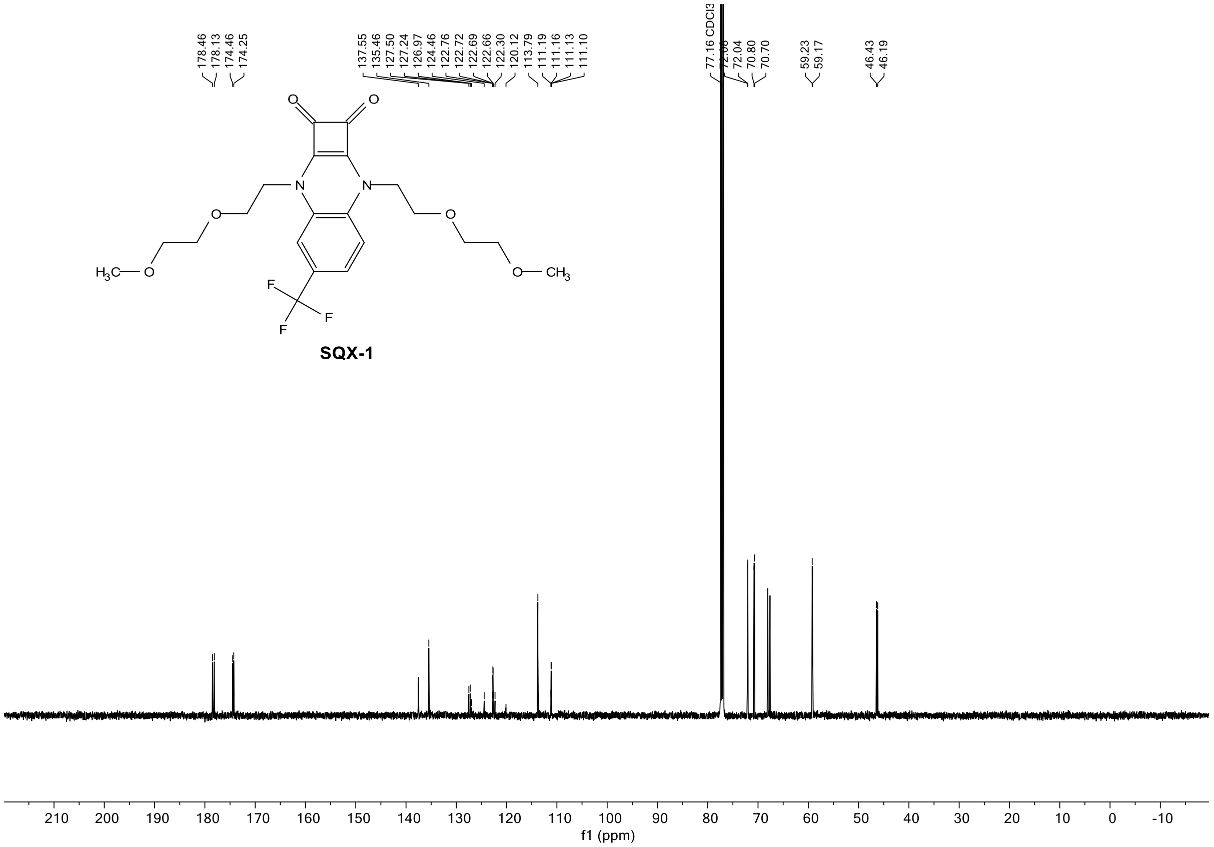
178.46
178.13
174.46
174.25
137.55
135.46
127.50
127.24
126.97
124.46
122.76
122.72
122.69
122.66
122.30
120.12
113.79
111.19
111.16
111.13
111.10
77.16 CDCl3
72.04
70.80
70.70
59.23
59.17
46.43
46.19
SQX-1
f1 (ppm)

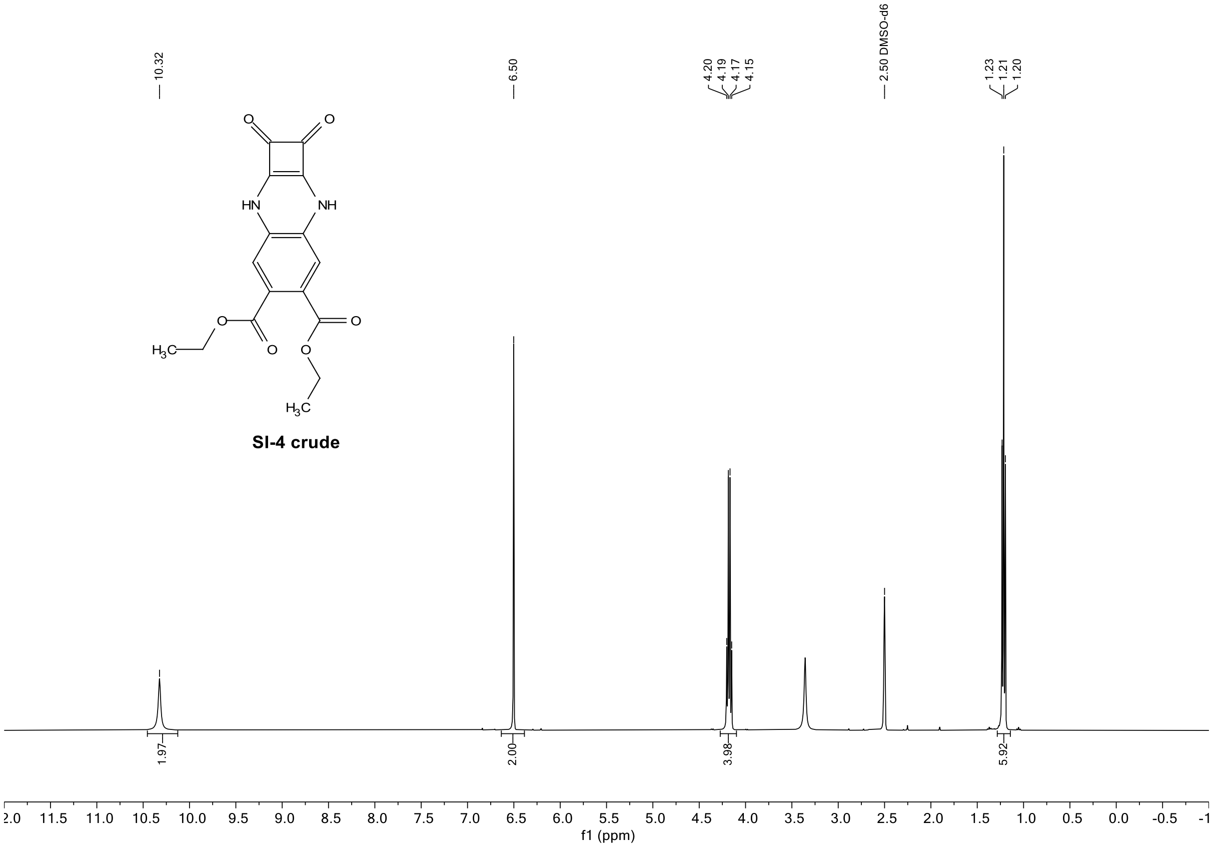
10.32
6.50
4.20
4.19
4.17
4.15
2.50 DMSO-d6
1.23
1.21
1.20
HN
NH
O
H3C
SI-4 crude
1.97
2.00
3.98
5.92
2.0 11.5 11.0 10.5 10.0 9.5 9.0 8.5 8.0 7.5 7.0 6.5 6.0 5.5 5.0 4.5 4.0 3.5 3.0 2.5 2.0 1.5 1.0 0.5 0.0 -0.5 -1
f1 (ppm)

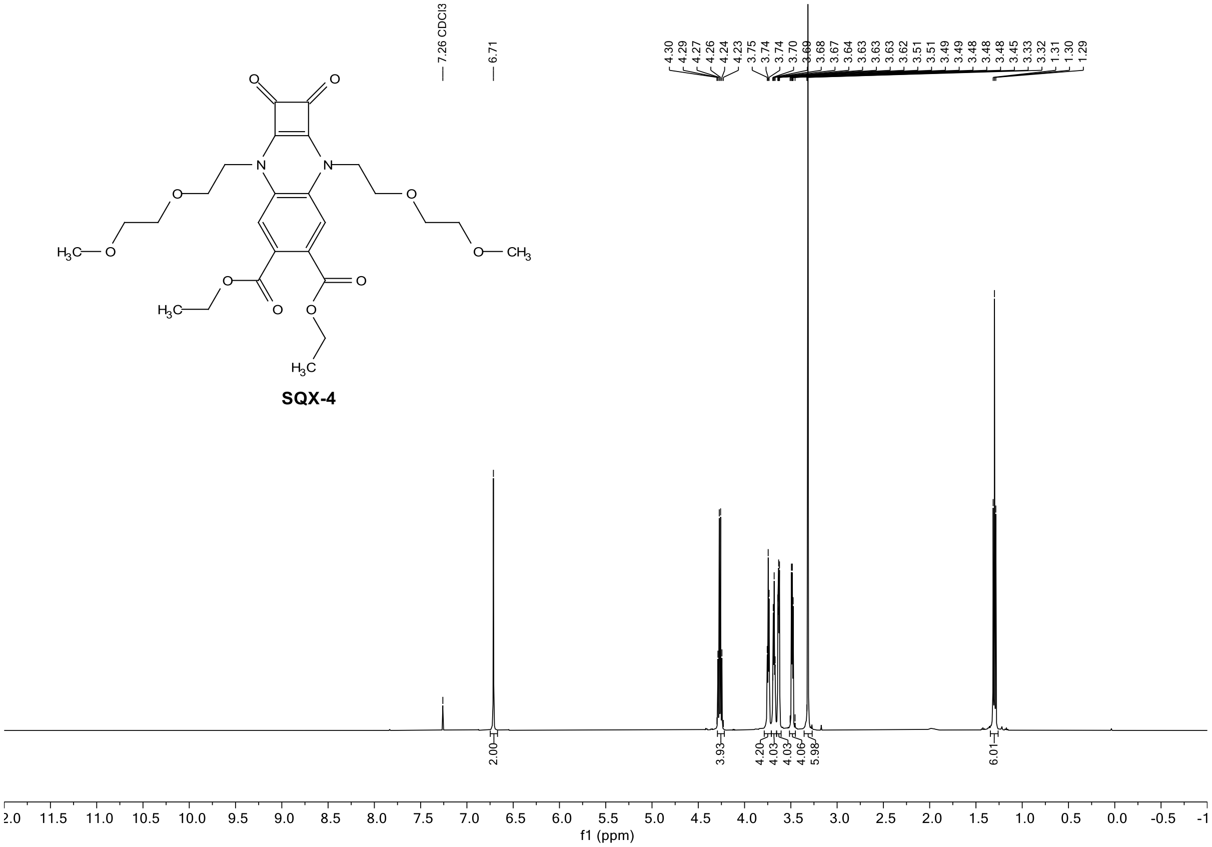
7.26 CDCl3
6.71
4.30
4.29
4.27
4.26
4.24
4.23
3.75
3.74
3.74
3.70
3.69
3.68
3.67
3.64
3.63
3.63
3.63
3.62
3.51
3.51
3.49
3.49
3.48
3.48
3.48
3.45
3.33
3.32
1.31
1.30
1.29
2.00
3.93
4.20
4.03
4.03
4.06
5.98
6.01
f1 (ppm)

SQX-4

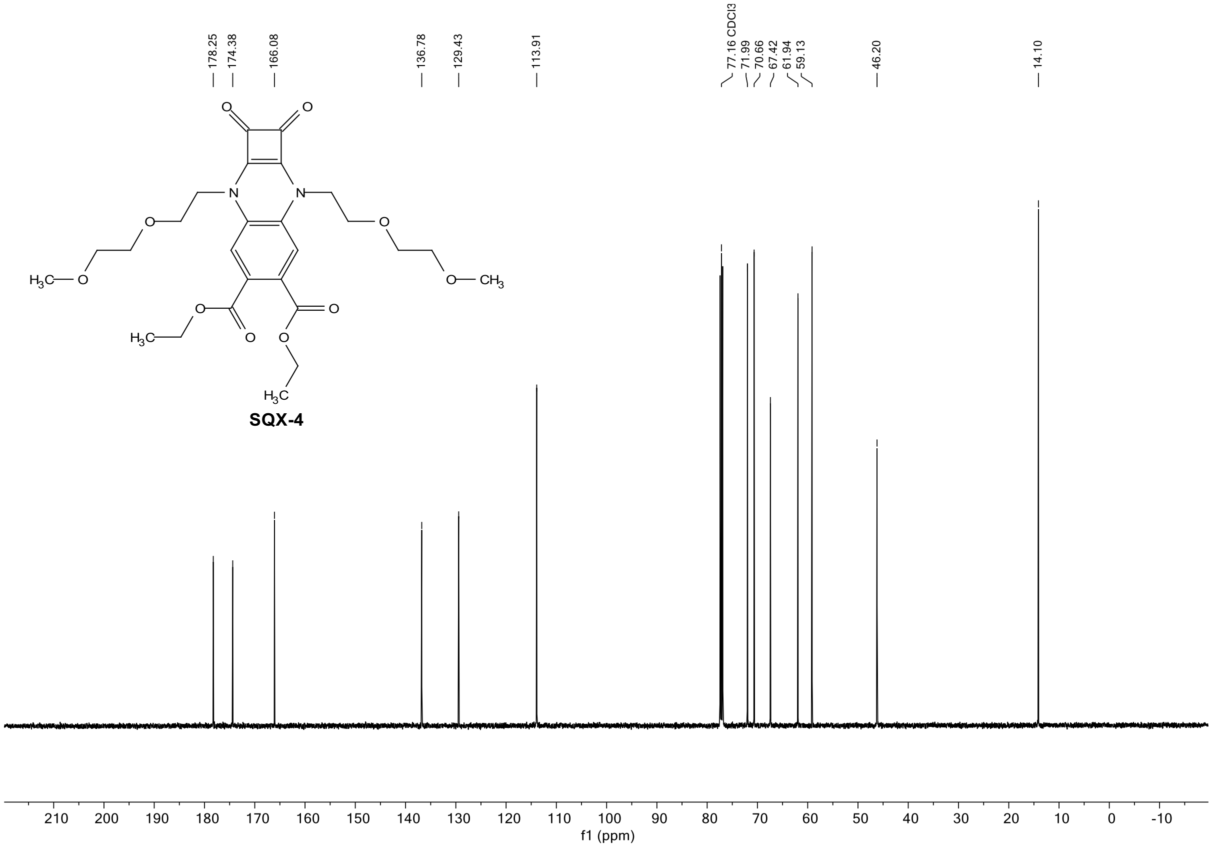
178.25
174.38
166.08
136.78
129.43
113.91
77.16 CDCl3
71.99
70.66
67.42
61.94
59.13
46.20
14.10
f1 (ppm)

SQX-4

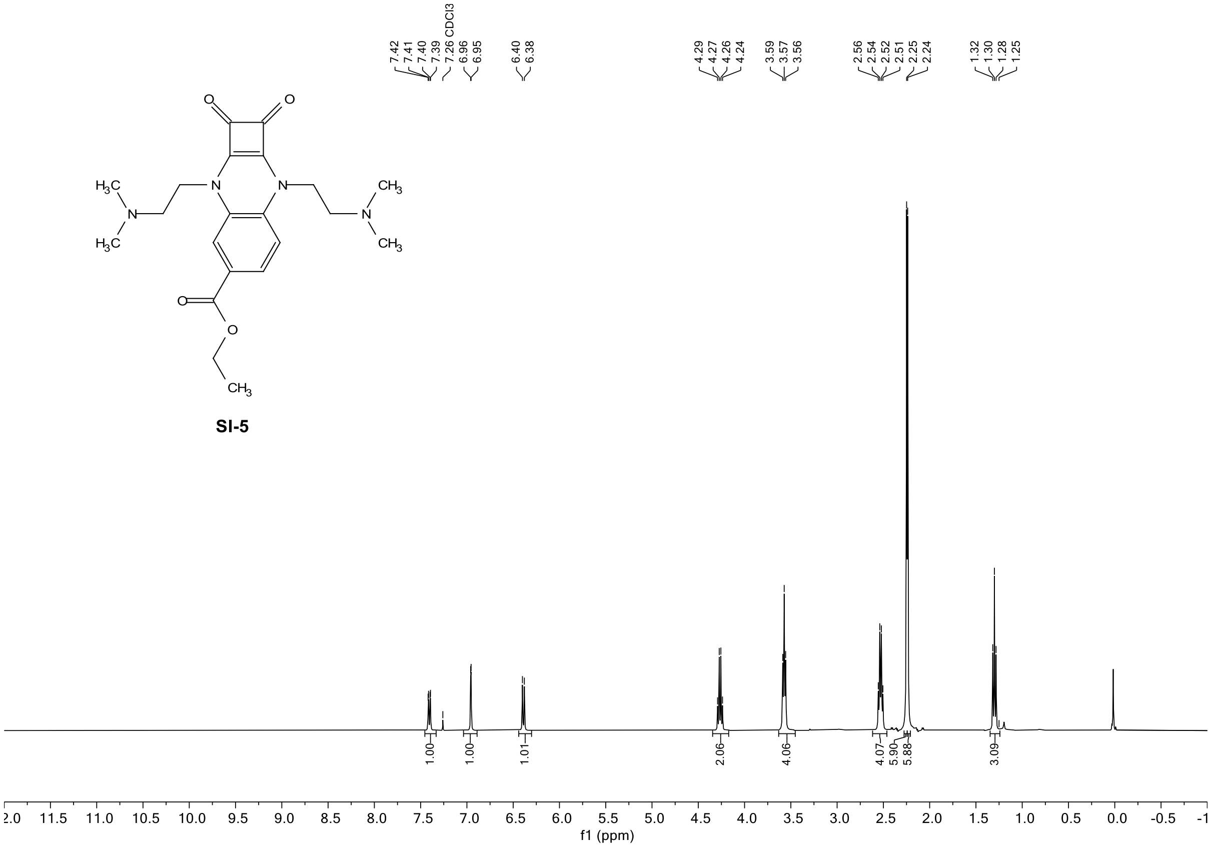
7.42
7.41
7.40
7.39
7.26 CDCl3
6.96
6.95
6.40
6.38
4.29
4.27
4.26
4.24
3.59
3.57
3.56
2.56
2.54
2.52
2.51
2.25
2.24
1.32
1.30
1.28
1.25
SI-5
1.00
1.00
1.01
2.06
4.06
4.07
5.90
5.88
3.09
f1 (ppm)

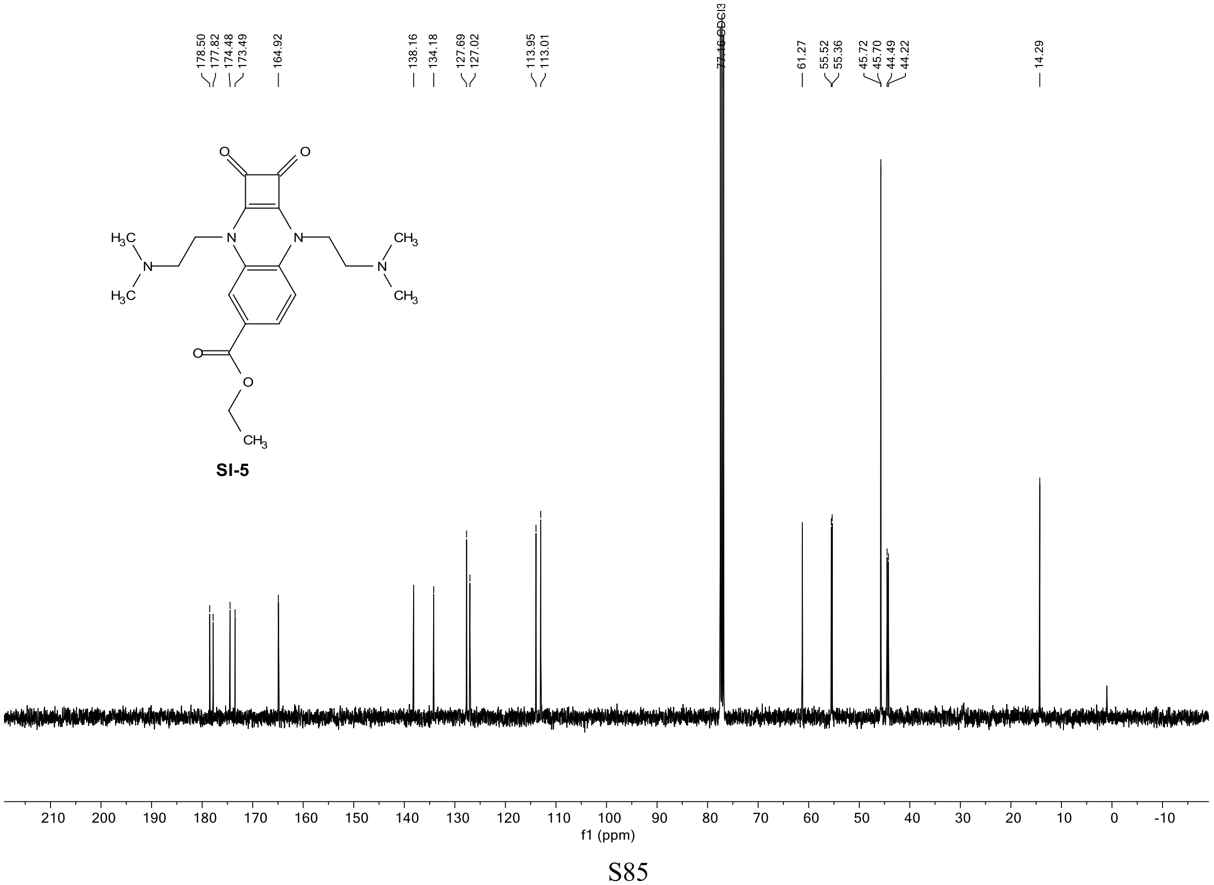
178.50
177.82
174.48
173.49
164.92
138.16
134.18
127.69
127.02
113.95
113.01
77.16 CDCl3
61.27
55.52
55.36
45.72
45.70
44.49
44.22
14.29
SI-5
f1 (ppm)

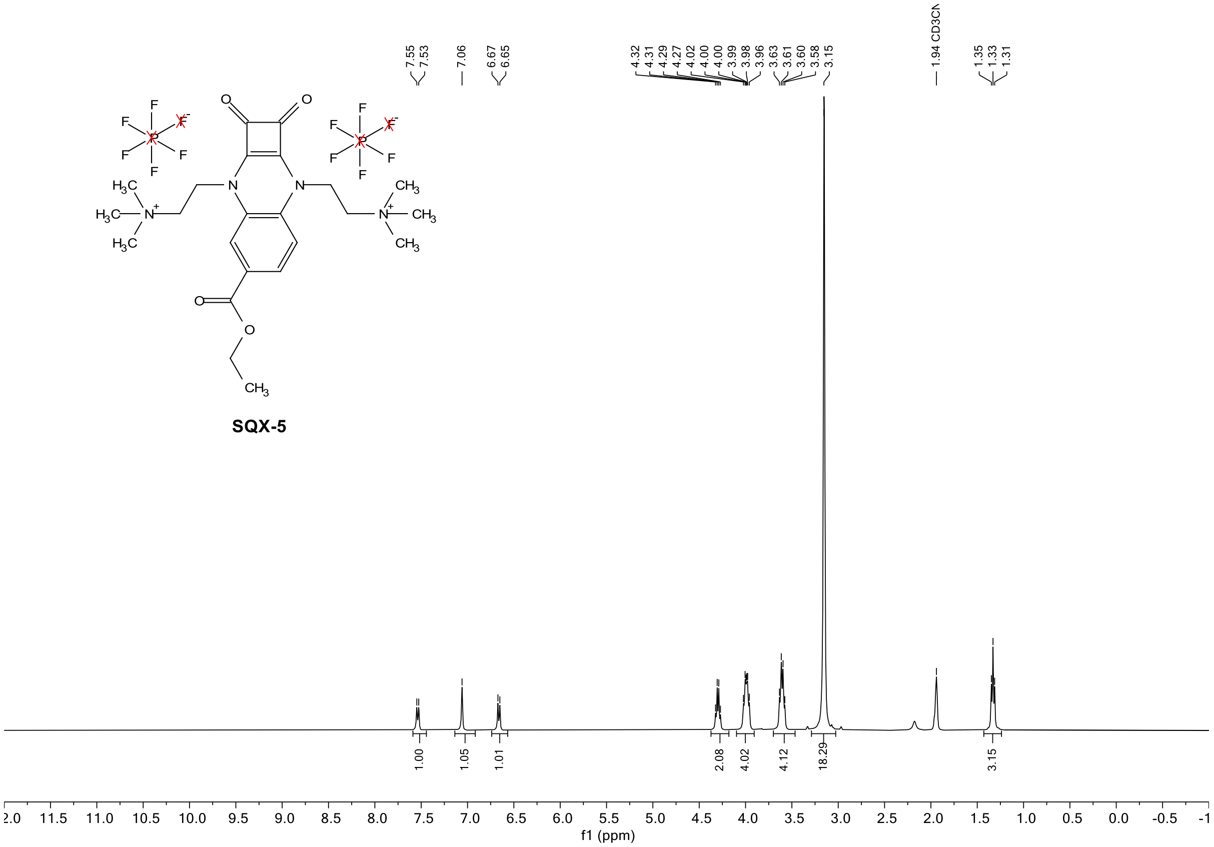

7.55
7.53
7.06
6.67
6.65
4.32
4.31
4.29
4.27
4.02
4.00
4.00
3.99
3.98
3.96
3.63
3.61
3.60
3.58
3.15
1.94 CD3CN
1.35
1.33
1.31
SQX-5
1.00
1.05
1.01
2.08
4.02
4.12
18.29
3.15
f1 (ppm)

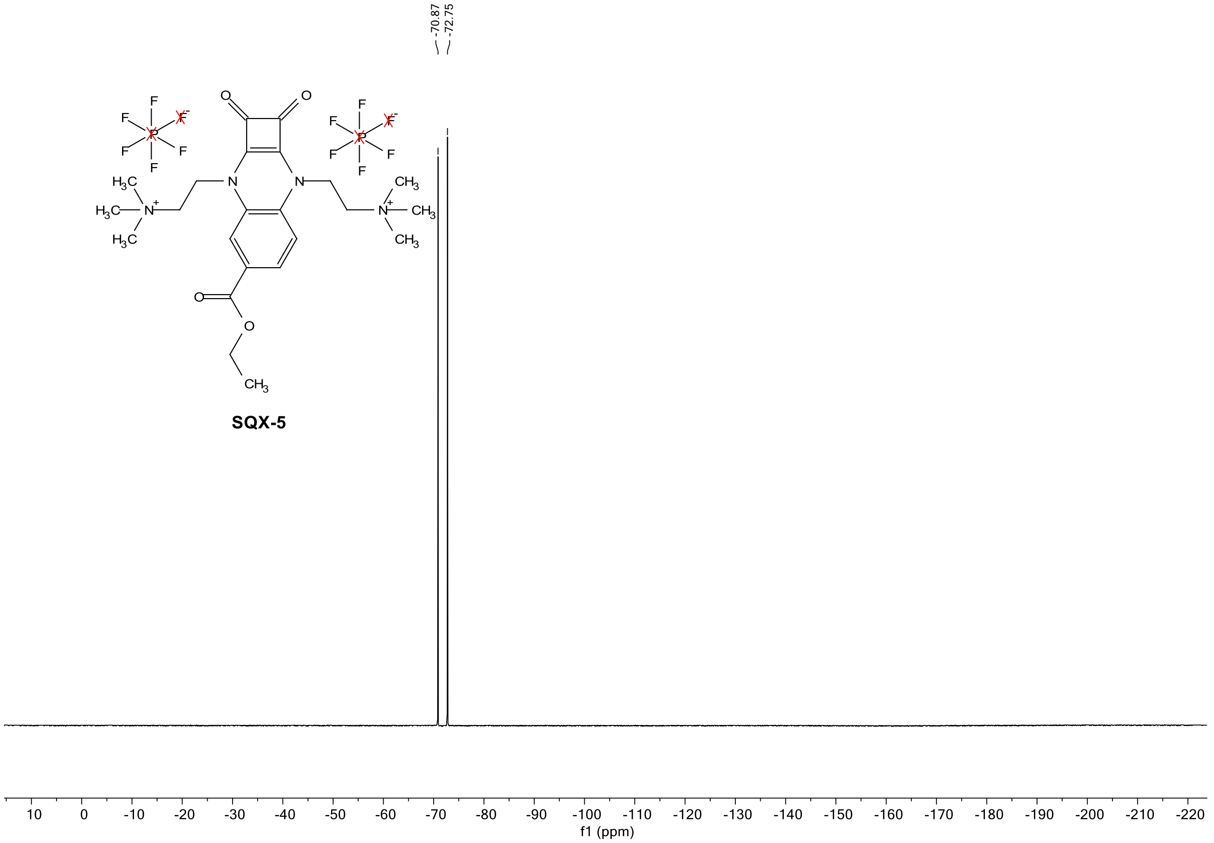

-70.87
-72.75
SQX-5
f1 (ppm)

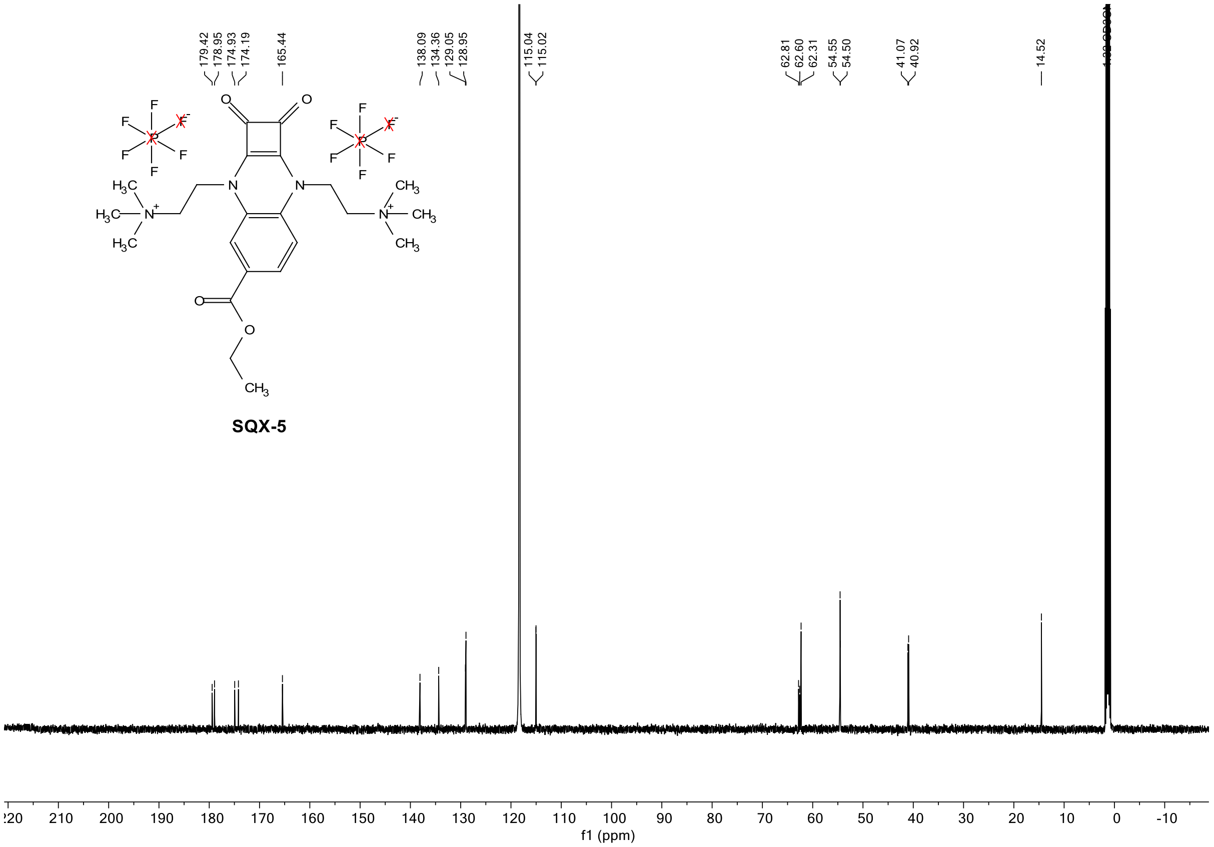

179.42
178.95
174.93
174.19
165.44
138.09
134.36
129.05
128.95
115.04
115.02
62.81
62.60
62.31
54.55
54.50
41.07
40.92
14.52
SQX-5
f1 (ppm)

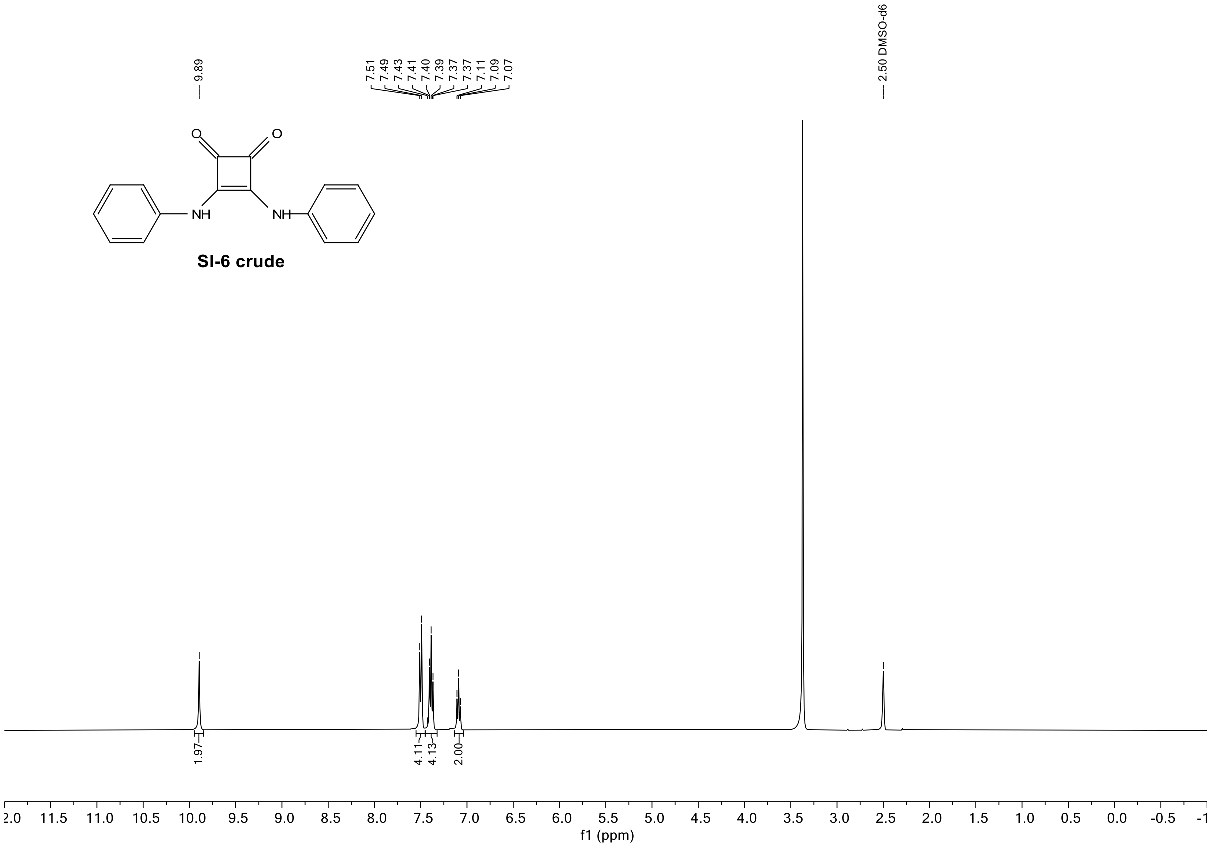
9.89
7.51
7.49
7.43
7.41
7.40
7.39
7.37
7.37
7.11
7.09
7.07
2.50 DMSO-d6
O
O
NH
NH
SI-6 crude
1.97
4.11
4.13
2.00
f1 (ppm)

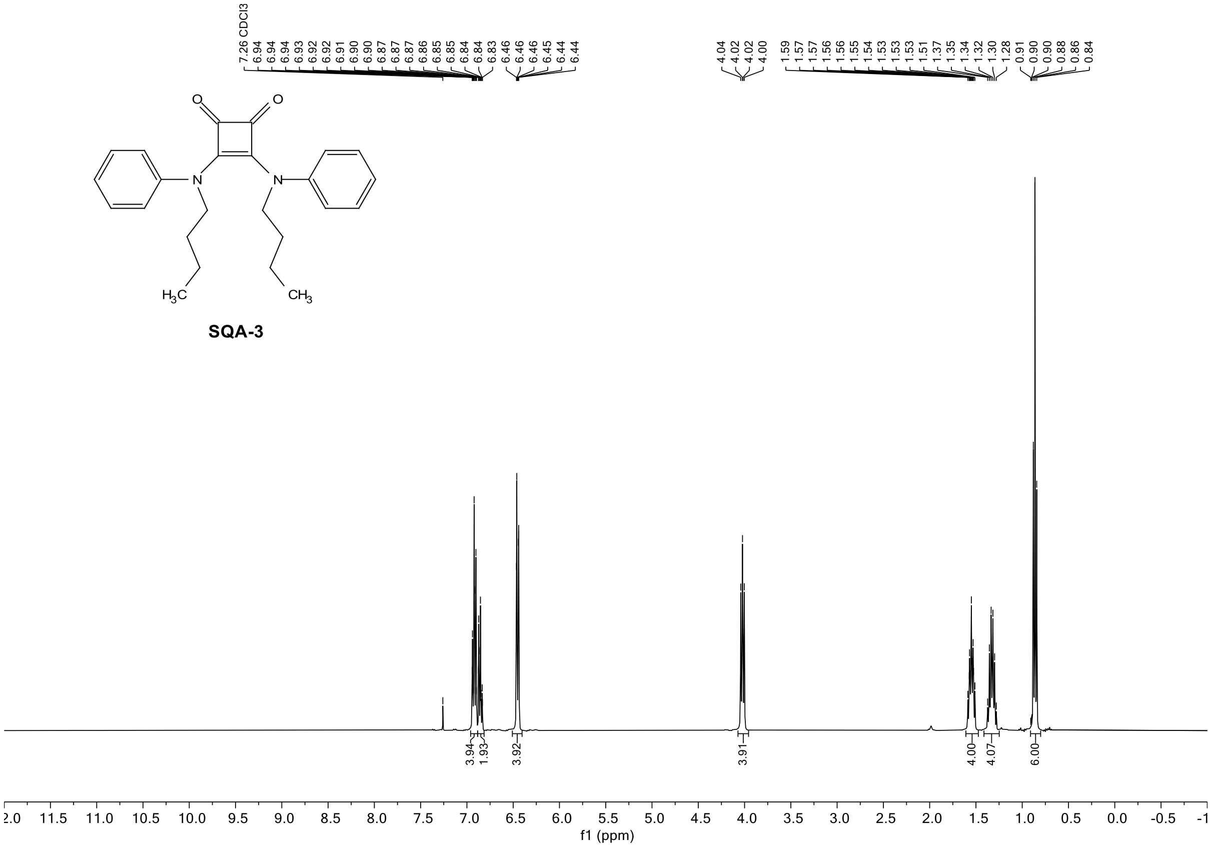
7.26 CDCl3
6.94
6.94
6.94
6.93
6.92
6.92
6.91
6.90
6.90
6.87
6.87
6.87
6.86
6.85
6.85
6.84
6.84
6.83
6.46
6.46
6.46
6.45
6.44
6.44
4.04
4.02
4.02
4.00
1.59
1.57
1.57
1.56
1.56
1.55
1.54
1.53
1.53
1.53
1.51
1.37
1.35
1.34
1.32
1.30
1.28
0.91
0.90
0.90
0.88
0.86
0.84
SQA-3
3.94
1.93
3.92
3.91
4.00
4.07
6.00
f1 (ppm)

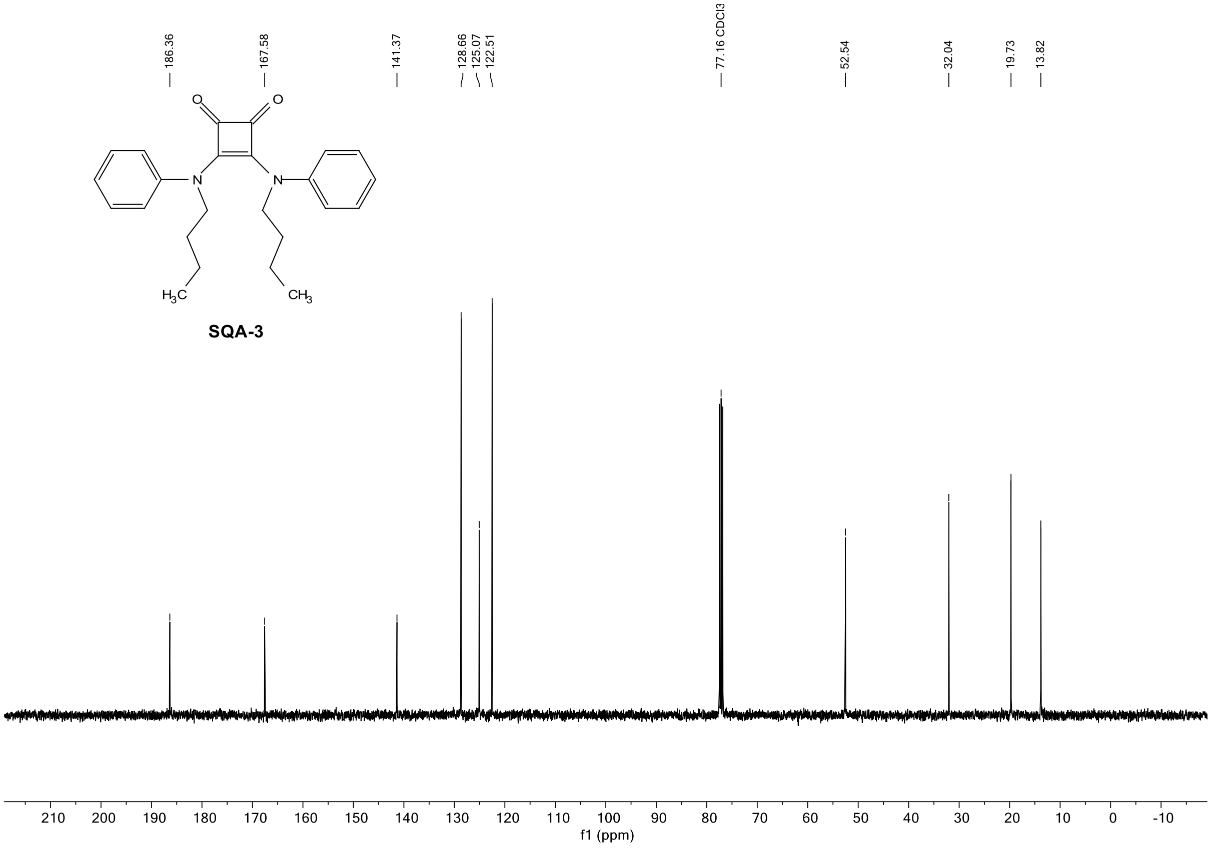
186.36
167.58
141.37
128.66
125.07
122.51
77.16 CDCl3
52.54
32.04
19.73
13.82
SQA-3
f1 (ppm)

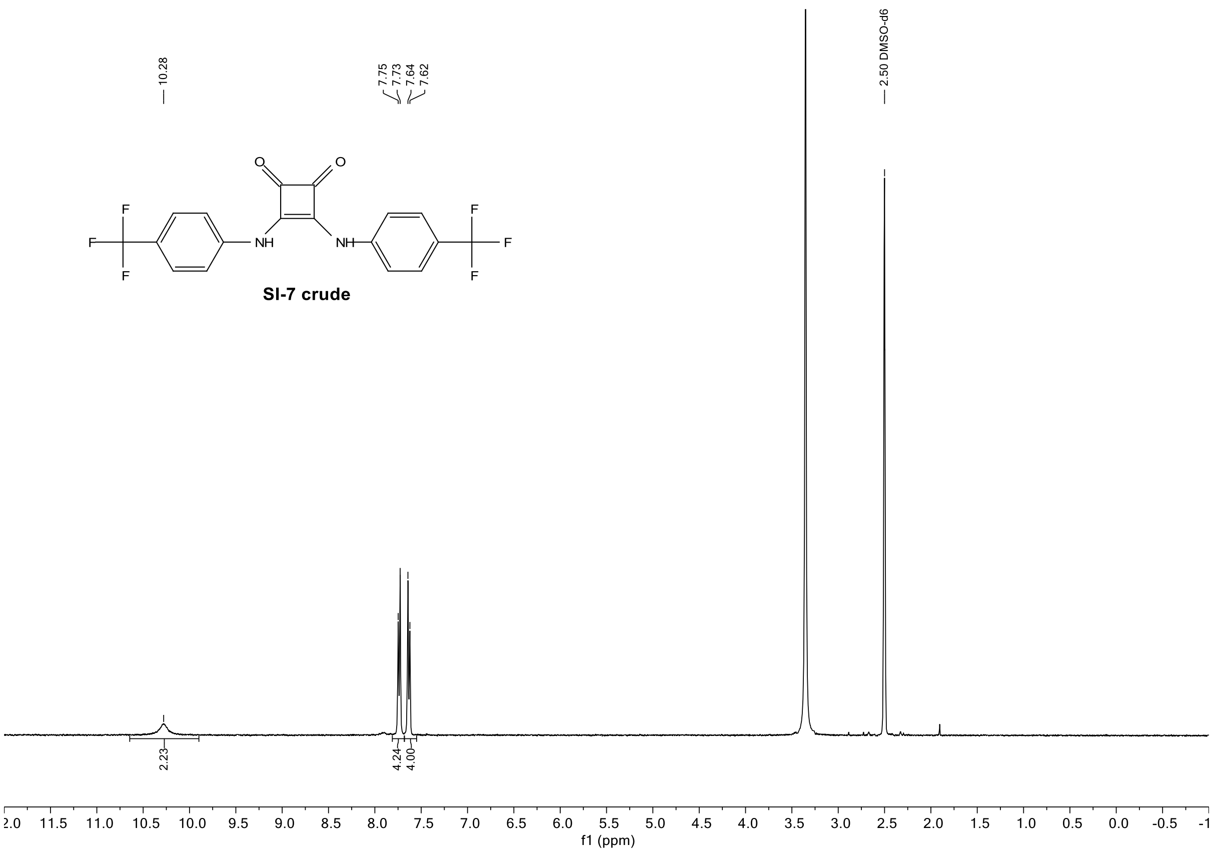

10.28
7.75
7.73
7.64
7.62
2.50 DMSO-d6
SI-7 crude
2.23
4.24
4.00
f1 (ppm)

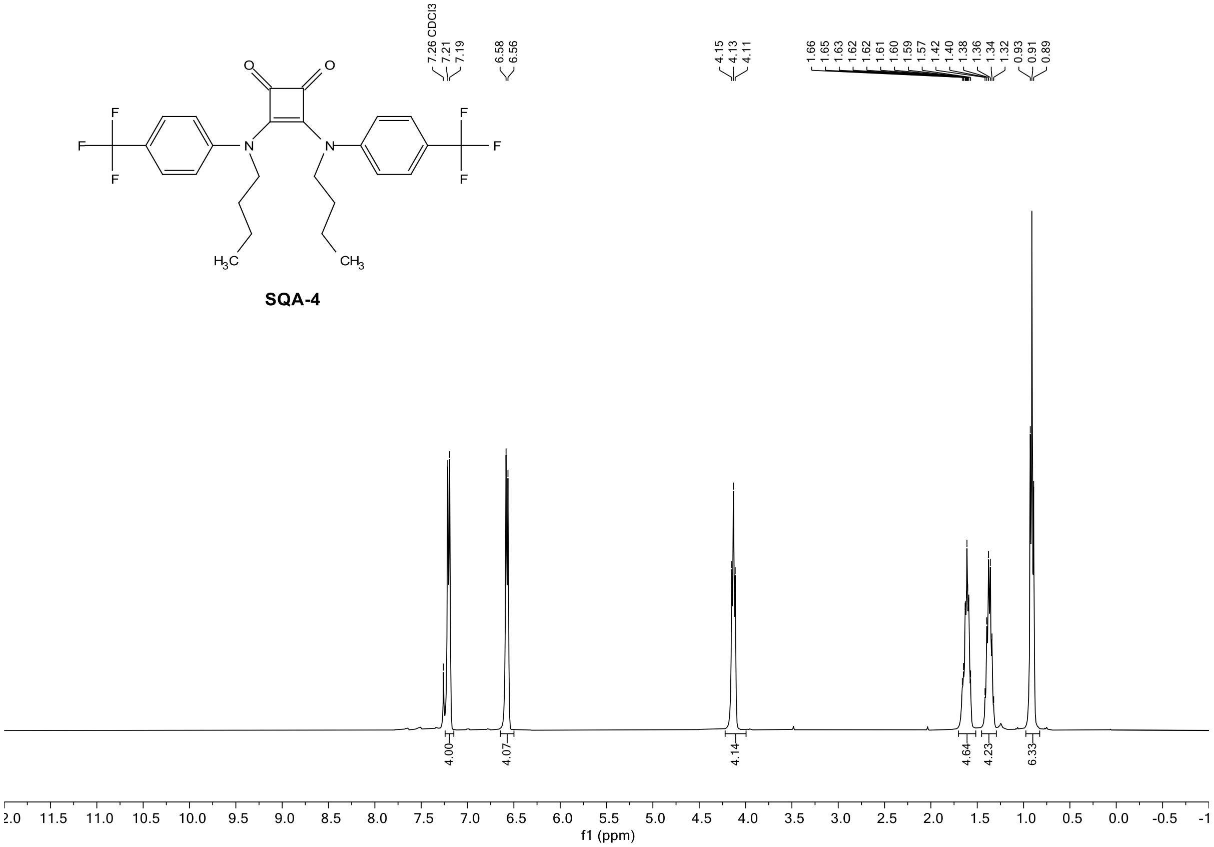
SQA-4
7.26 CDCl3
7.21
7.19
6.58
6.56
4.15
4.13
4.11
1.66
1.65
1.63
1.62
1.62
1.61
1.60
1.59
1.57
1.42
1.40
1.38
1.36
1.34
1.32
0.93
0.91
0.89
4.00
4.07
4.14
4.64
4.23
6.33
f1 (ppm)

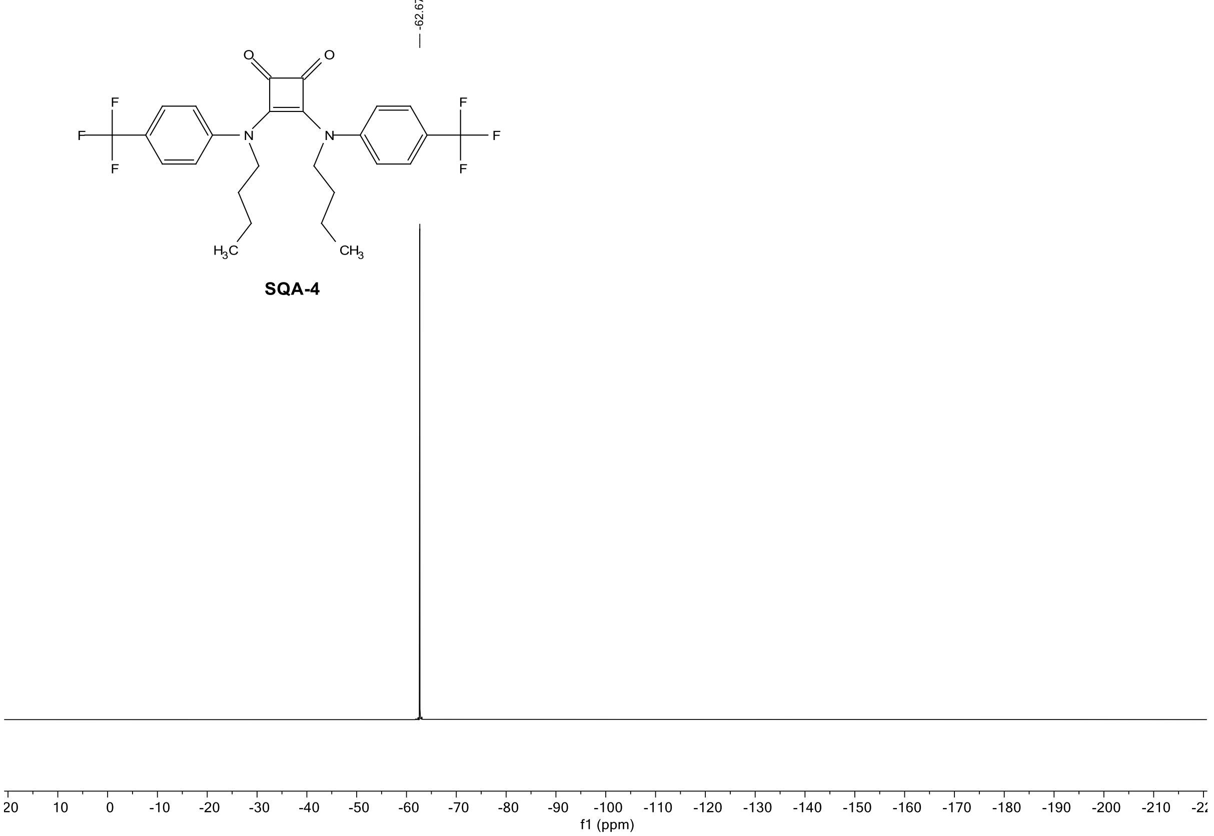
SQA-4
-62.67
f1 (ppm)

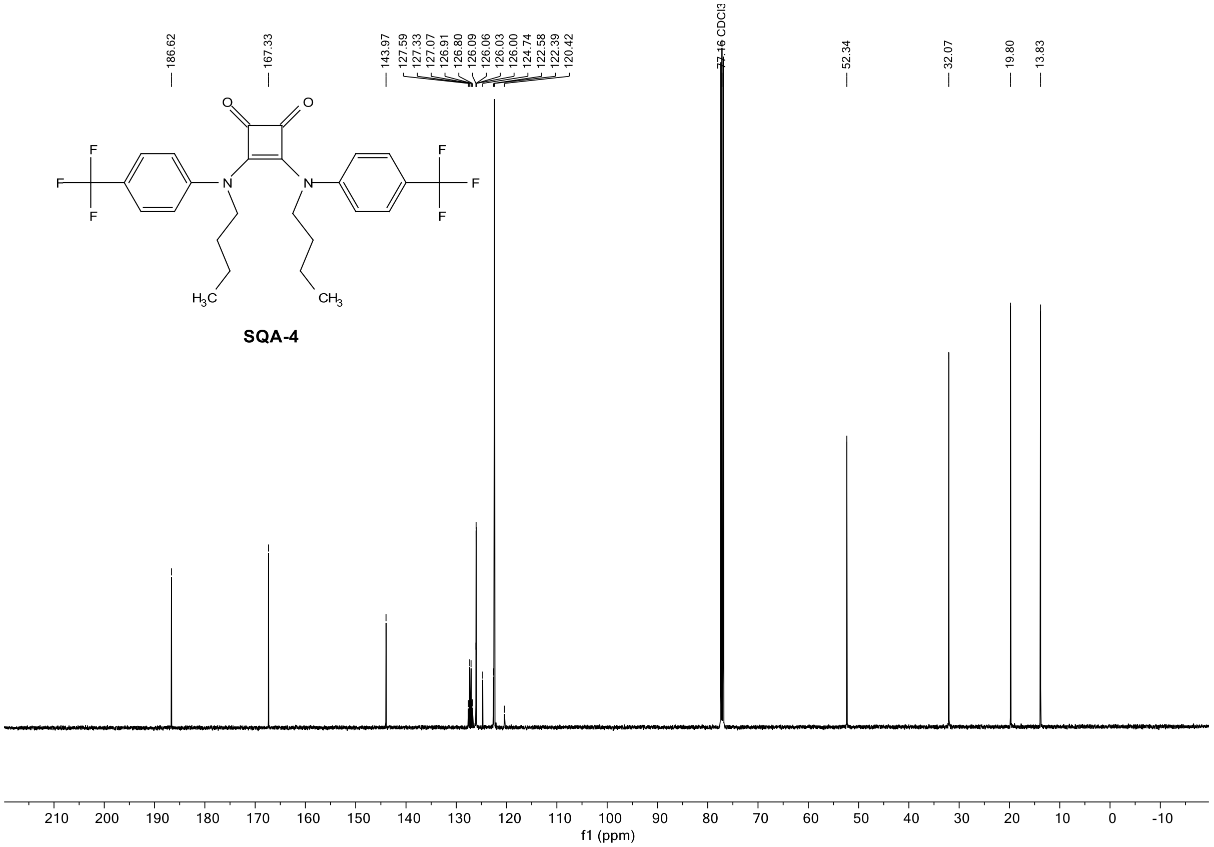
186.62
167.33
143.97
127.59
127.33
127.07
126.91
126.80
126.09
126.06
126.03
126.00
124.74
122.58
122.39
120.42
77.16 CDCl3
52.34
32.07
19.80
13.83
SQA-4
210 200 190 180 170 160 150 140 130 120 110 100 90 80 70 60 50 40 30 20 10 0 -10
f1 (ppm)

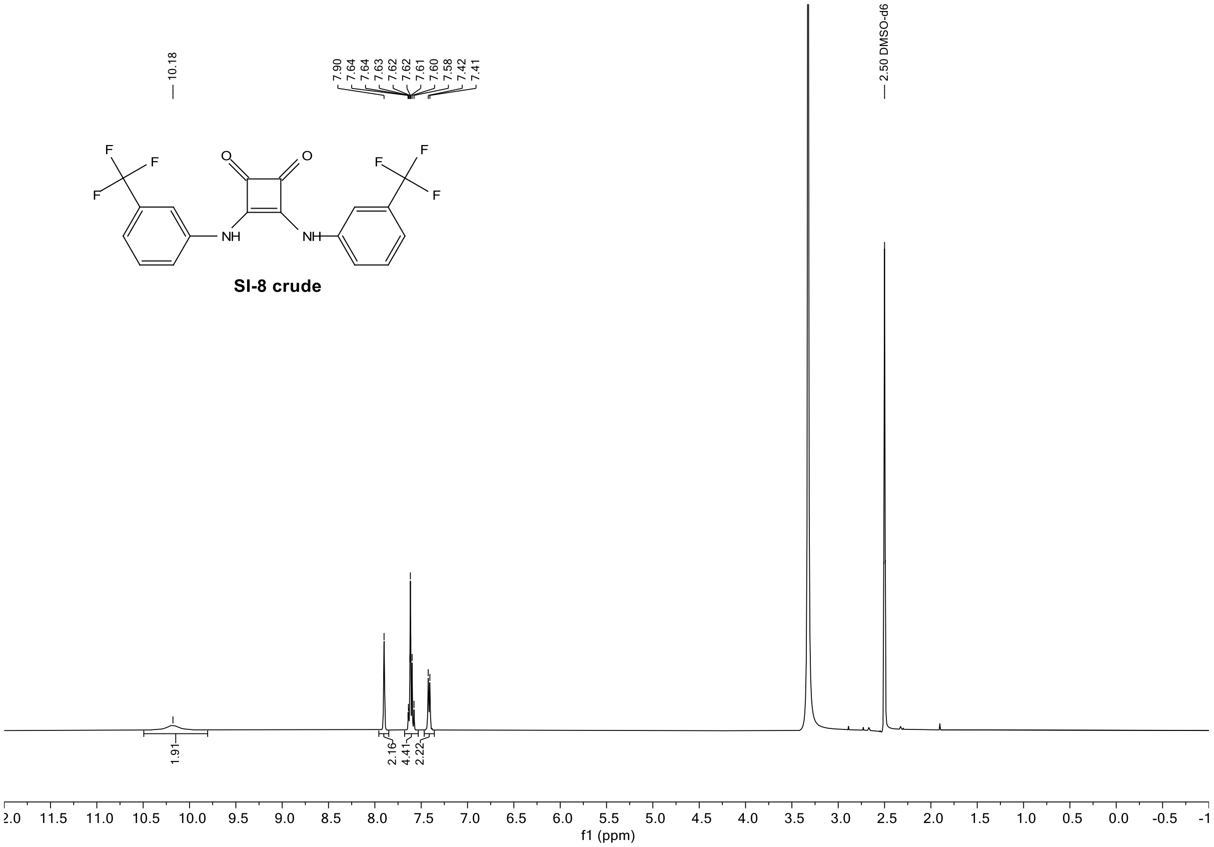
10.18
7.90
7.64
7.64
7.63
7.62
7.62
7.61
7.60
7.58
7.42
7.41
2.50 DMSO-d6
SI-8 crude
1.91
2.16
4.41
2.22
f1 (ppm)

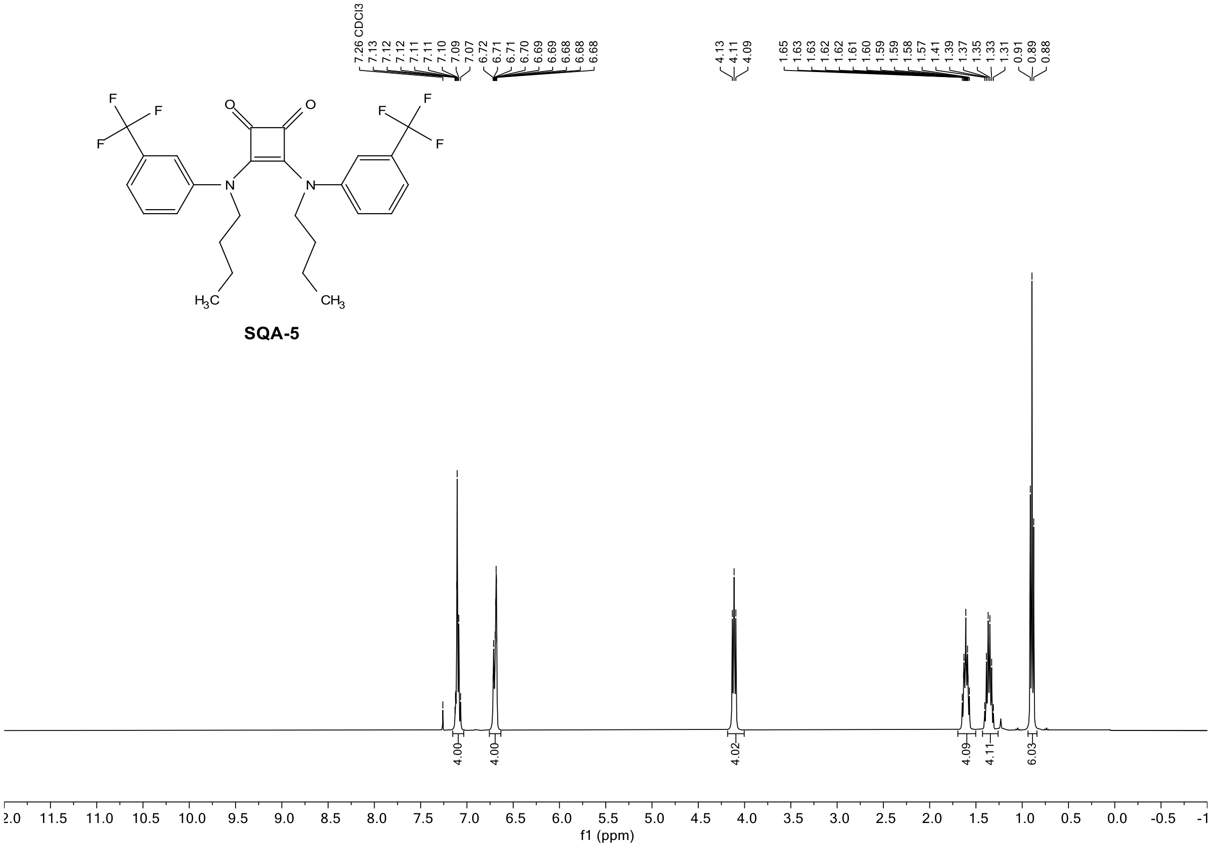
7.26 CDCl3
7.13
7.12
7.12
7.11
7.11
7.10
7.09
7.07
6.72
6.71
6.71
6.70
6.69
6.69
6.68
6.68
6.68
4.13
4.11
4.09
1.65
1.63
1.63
1.62
1.62
1.61
1.60
1.59
1.59
1.58
1.57
1.41
1.39
1.37
1.35
1.33
1.31
0.91
0.89
0.88
SQA-5
4.00
4.00
4.02
4.09
4.11
6.03
f1 (ppm)

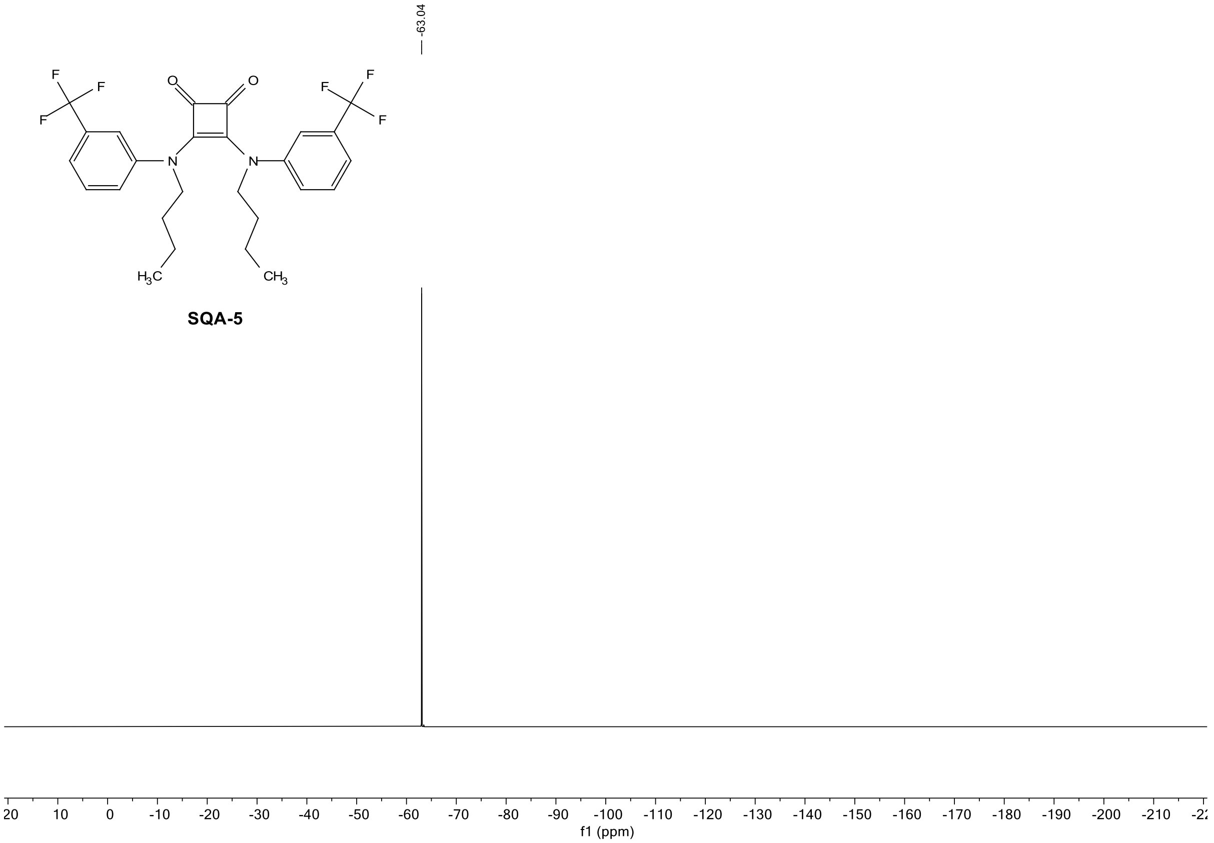
-63.04
SQA-5
f1 (ppm)

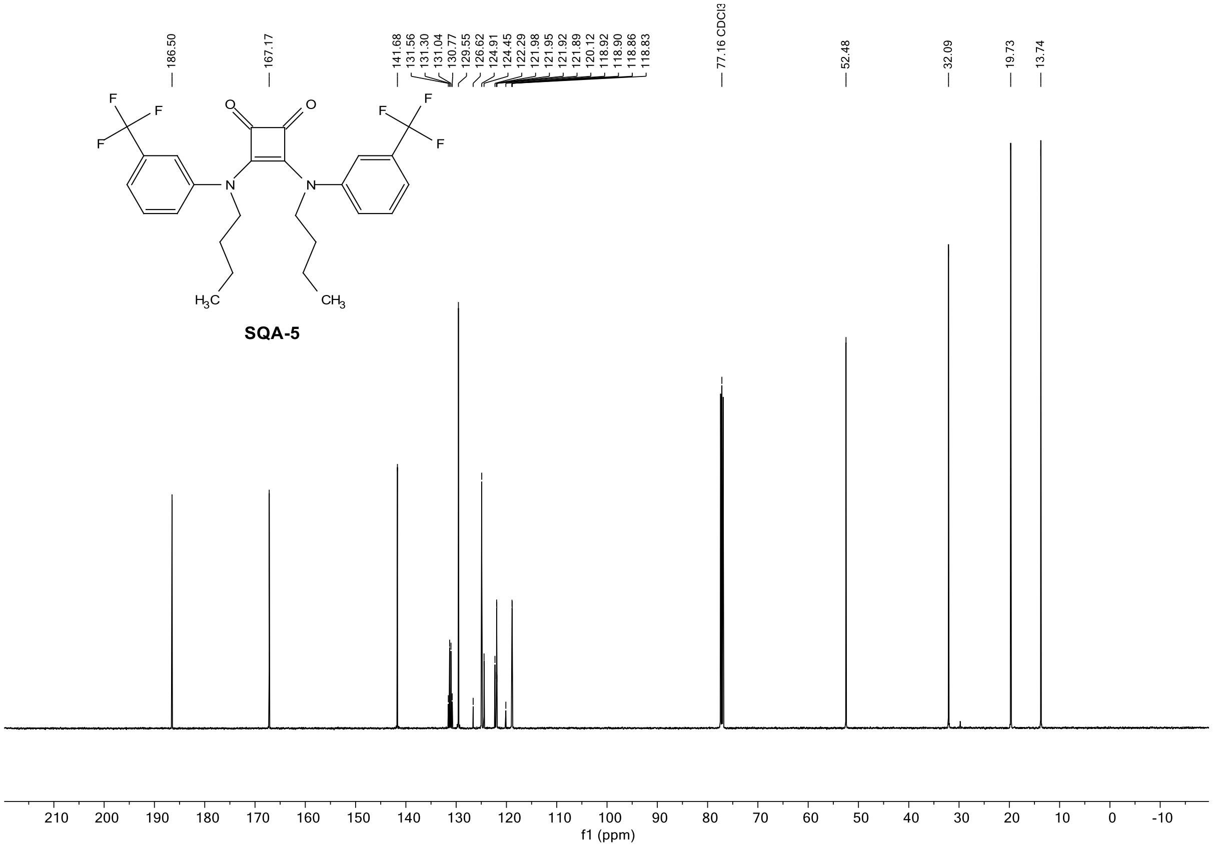

186.50
167.17
141.68
131.56
131.30
131.04
130.77
129.55
126.62
124.91
124.45
122.29
121.98
121.95
121.92
121.89
120.12
118.92
118.90
118.86
118.83
77.16 CDCl3
52.48
32.09
19.73
13.74
F
F
F
O
O
N
N
H3C
CH3
SQA-5
210 200 190 180 170 160 150 140 130 120 110 100 90 80 70 60 50 40 30 20 10 0 -10
f1 (ppm)

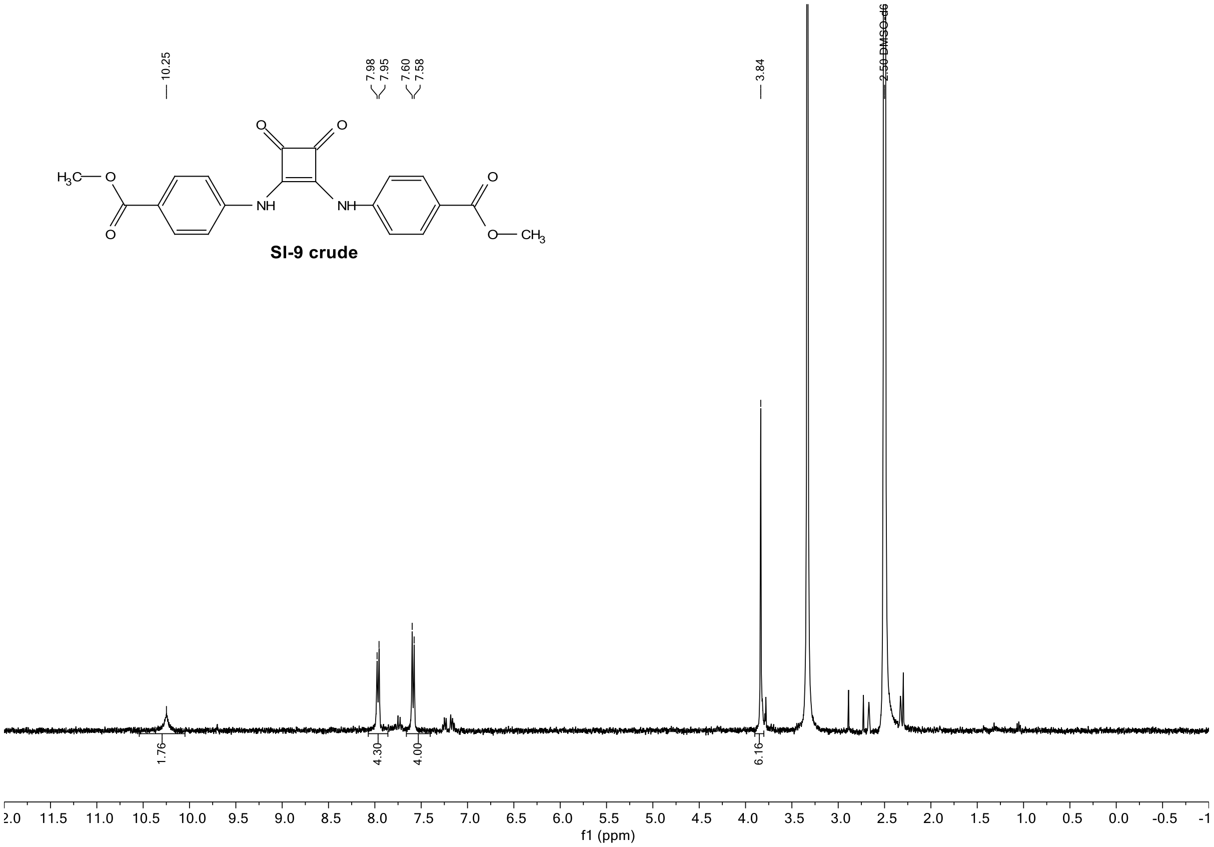

10.25
7.98
7.95
7.60
7.58
3.84
2.50 DMSO-d6
H_3C—O
NH
NH
O—CH_3
SI-9 crude
1.76
4.30
4.00
6.16
f1 (ppm)

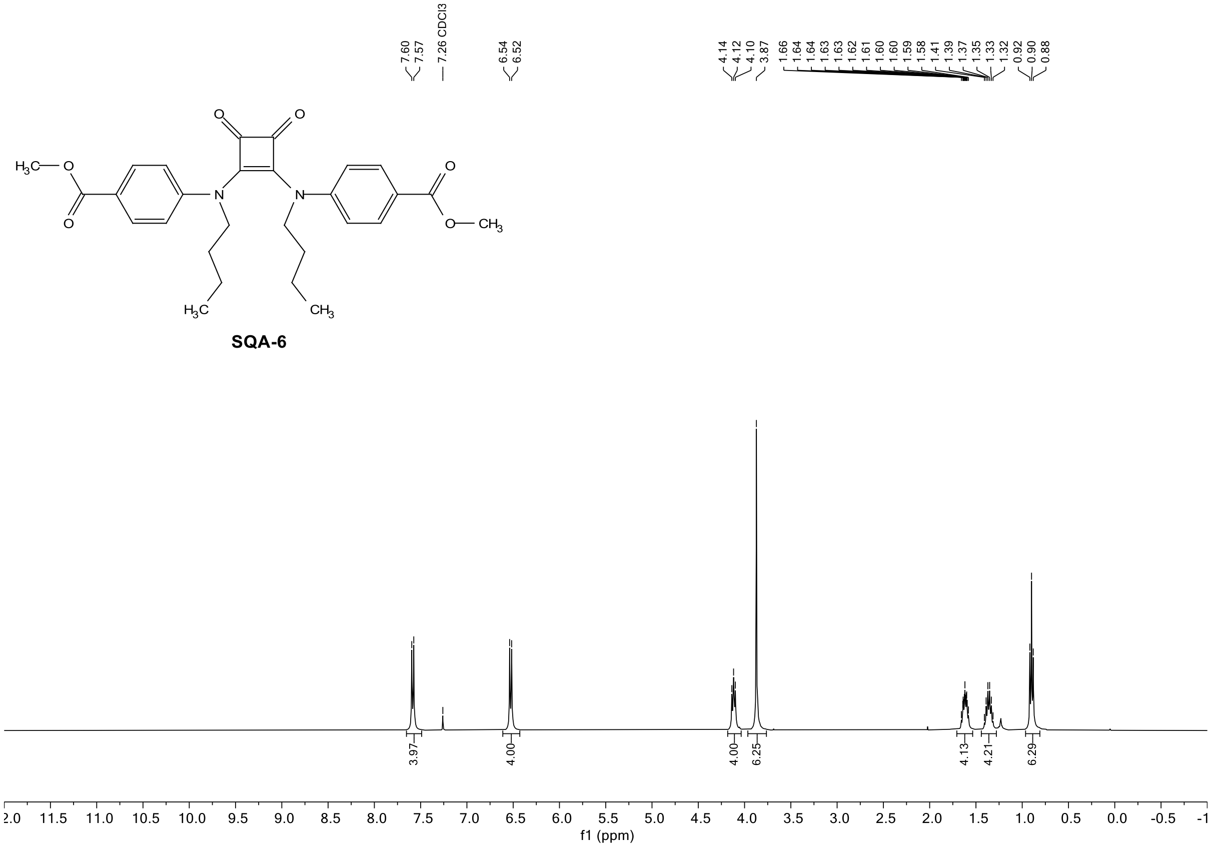
7.60
7.57
7.26 CDCl3
6.54
6.52
4.14
4.12
4.10
3.87
1.66
1.64
1.63
1.63
1.62
1.61
1.60
1.60
1.59
1.58
1.41
1.39
1.37
1.35
1.33
1.32
0.92
0.90
0.88
SQA-6
3.97
4.00
4.00
6.25
4.13
4.21
6.29
f1 (ppm)

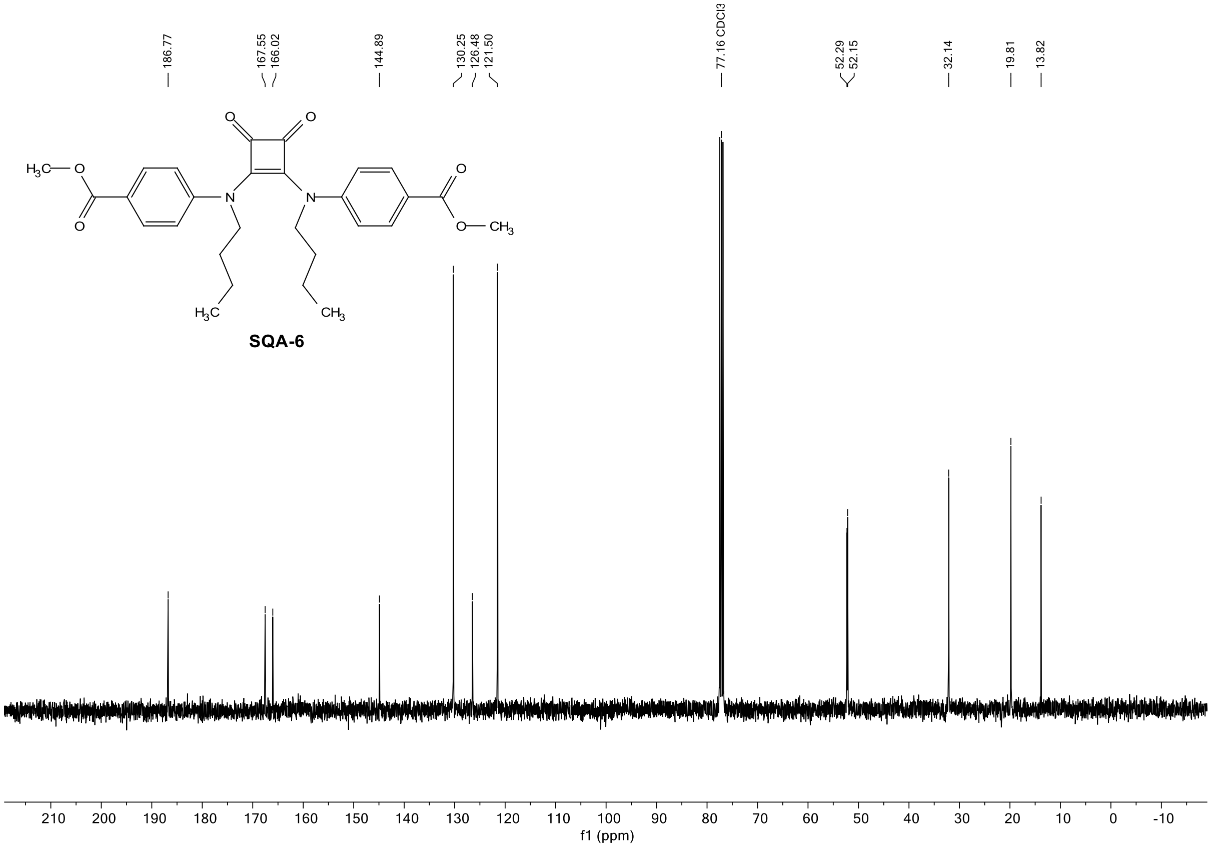
186.77
167.55
166.02
144.89
130.25
126.48
121.50
77.16 CDCl3
52.29
52.15
32.14
19.81
13.82
SQA-6
f1 (ppm)

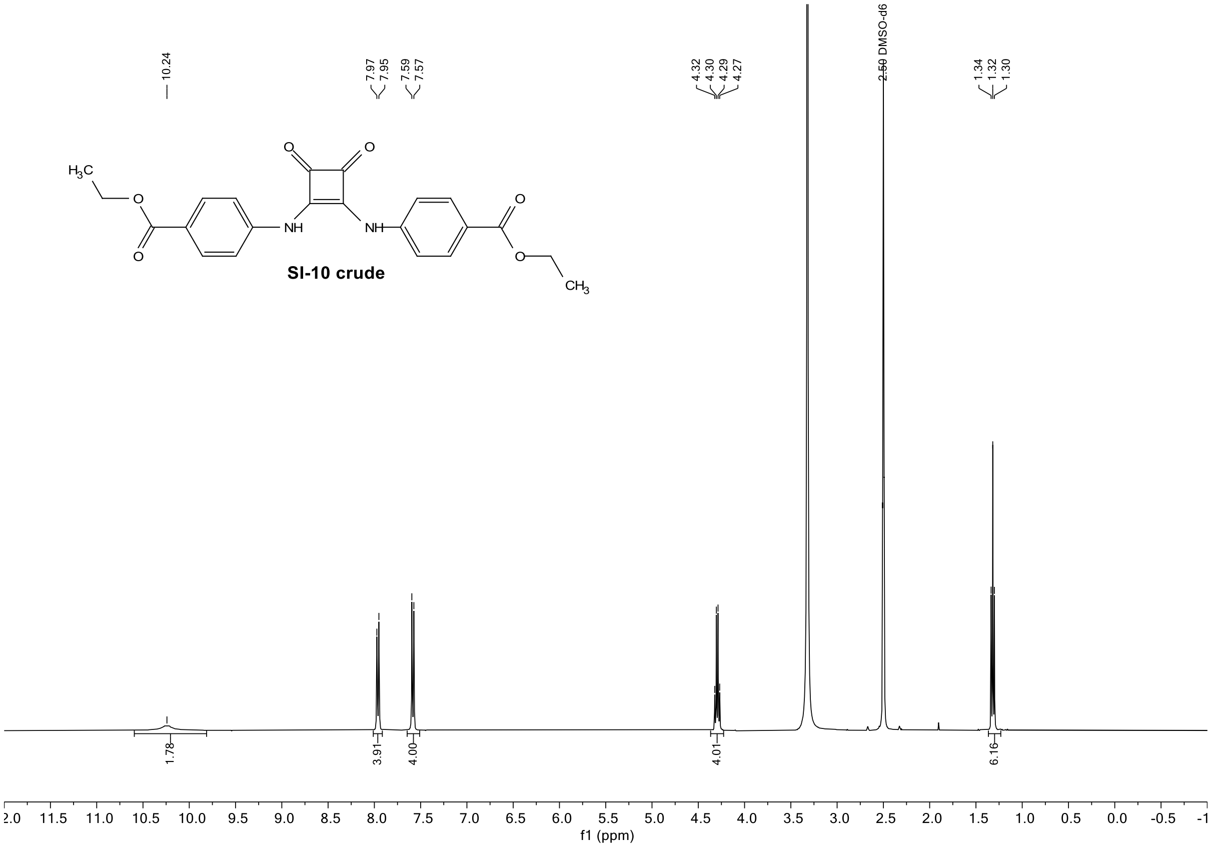

SI-10 crude
10.24
7.97
7.95
7.59
7.57
4.32
4.30
4.29
4.27
2.50 DMSO-d6
1.34
1.32
1.30
1.78
3.91
4.00
4.01
6.16
f1 (ppm)

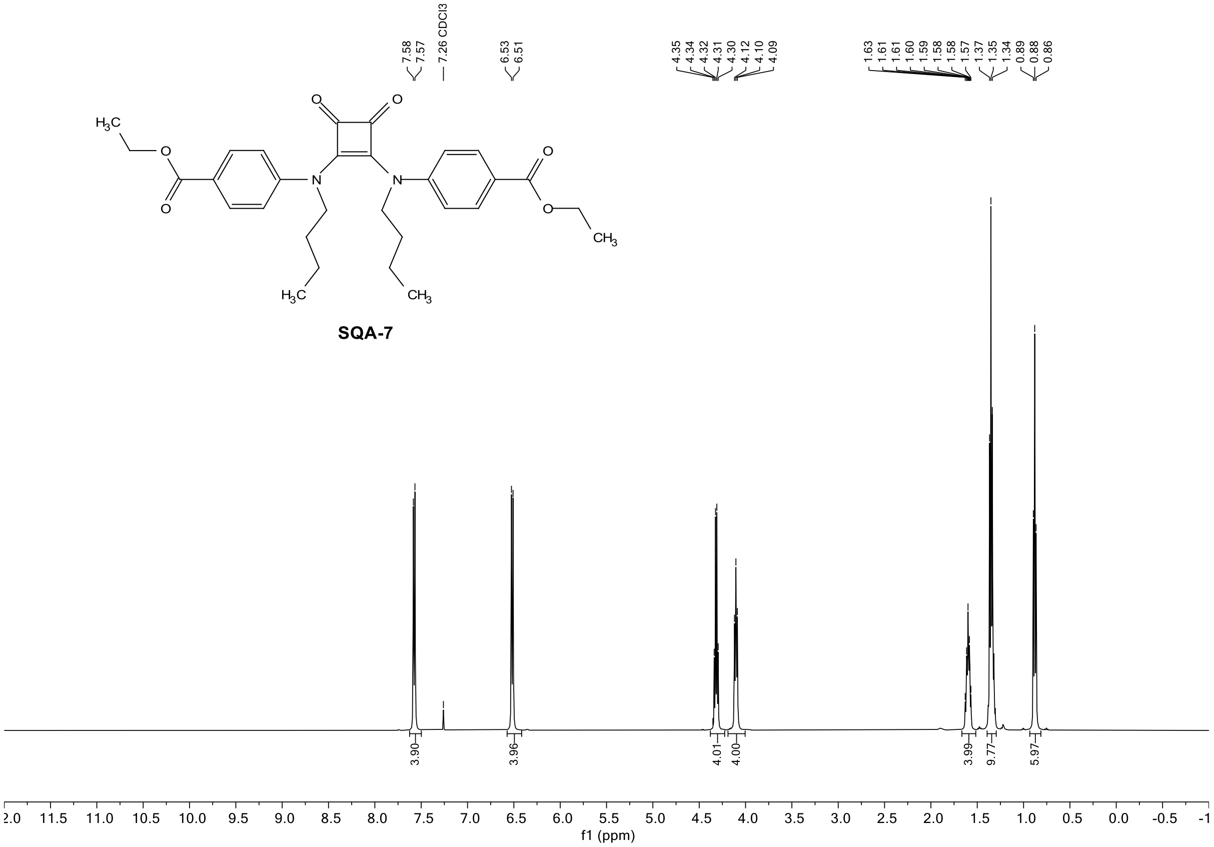
7.58
7.57
7.26 CDCl3
6.53
6.51
4.35
4.34
4.32
4.31
4.30
4.12
4.10
4.09
1.63
1.61
1.61
1.60
1.59
1.58
1.58
1.57
1.37
1.35
1.34
0.89
0.88
0.86
SQA-7
3.90
3.96
4.01
4.00
3.99
9.77
5.97
f1 (ppm)

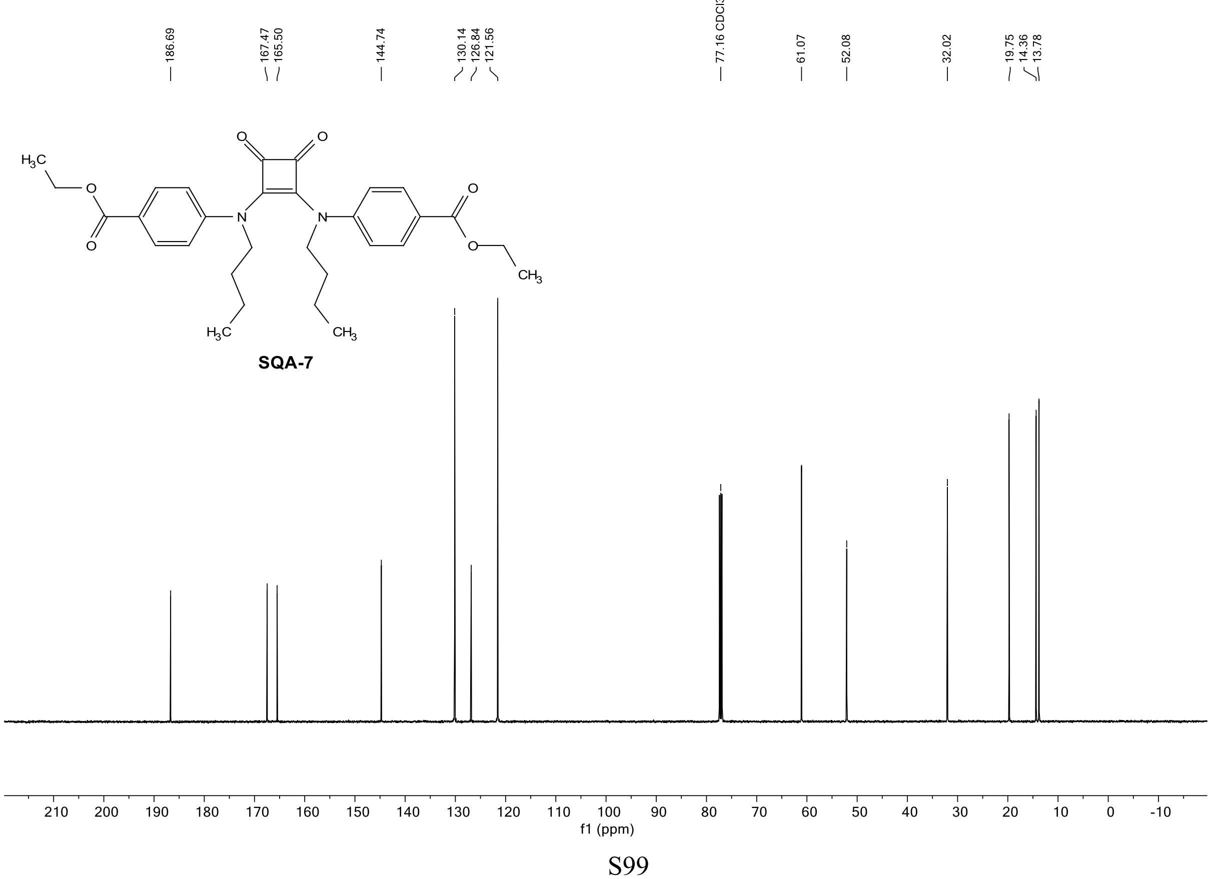
186.69
167.47
165.50
144.74
130.14
126.84
121.56
77.16 CDCl3
61.07
52.08
32.02
19.75
14.36
13.78
SQA-7
f1 (ppm)

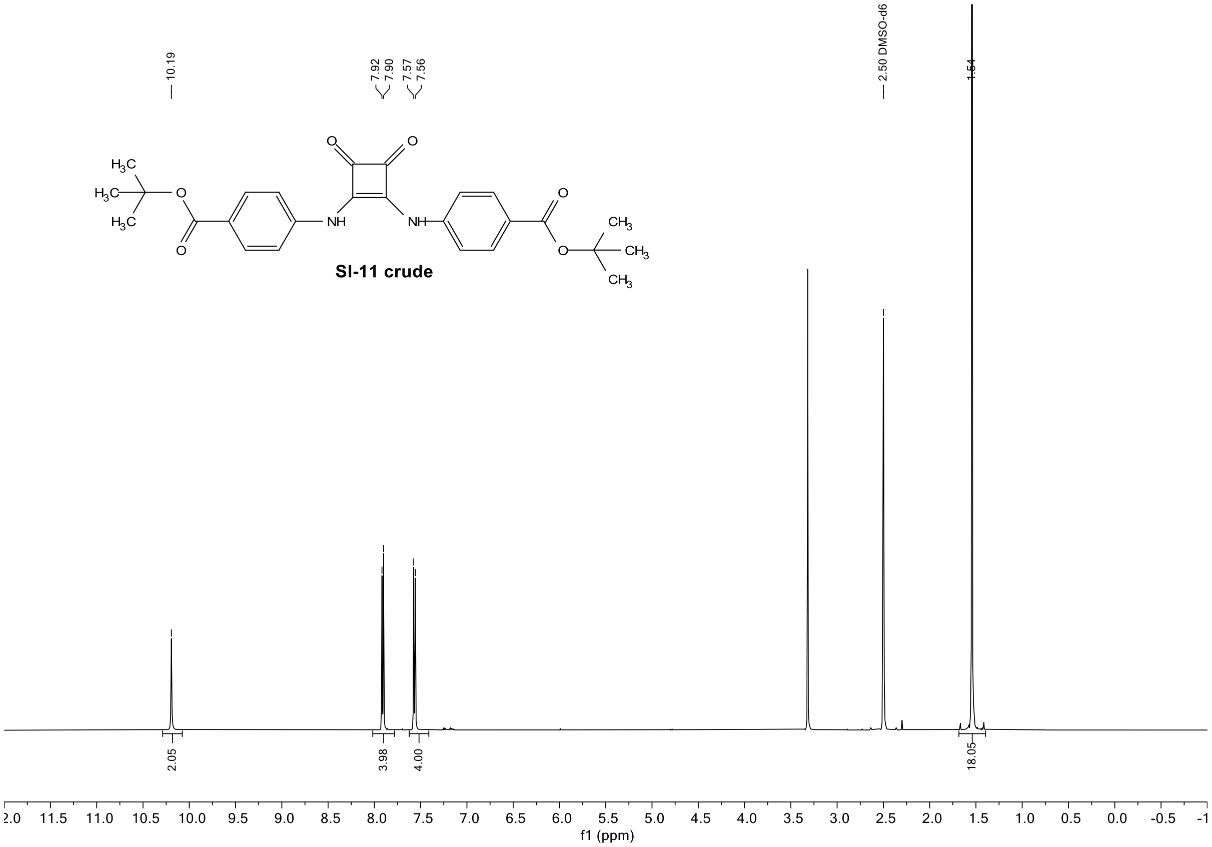

SI-11 crude
10.19
7.92
7.90
7.57
7.56
2.50 DMSO-d6
1.54
2.05
3.98
4.00
18.05
f1 (ppm)

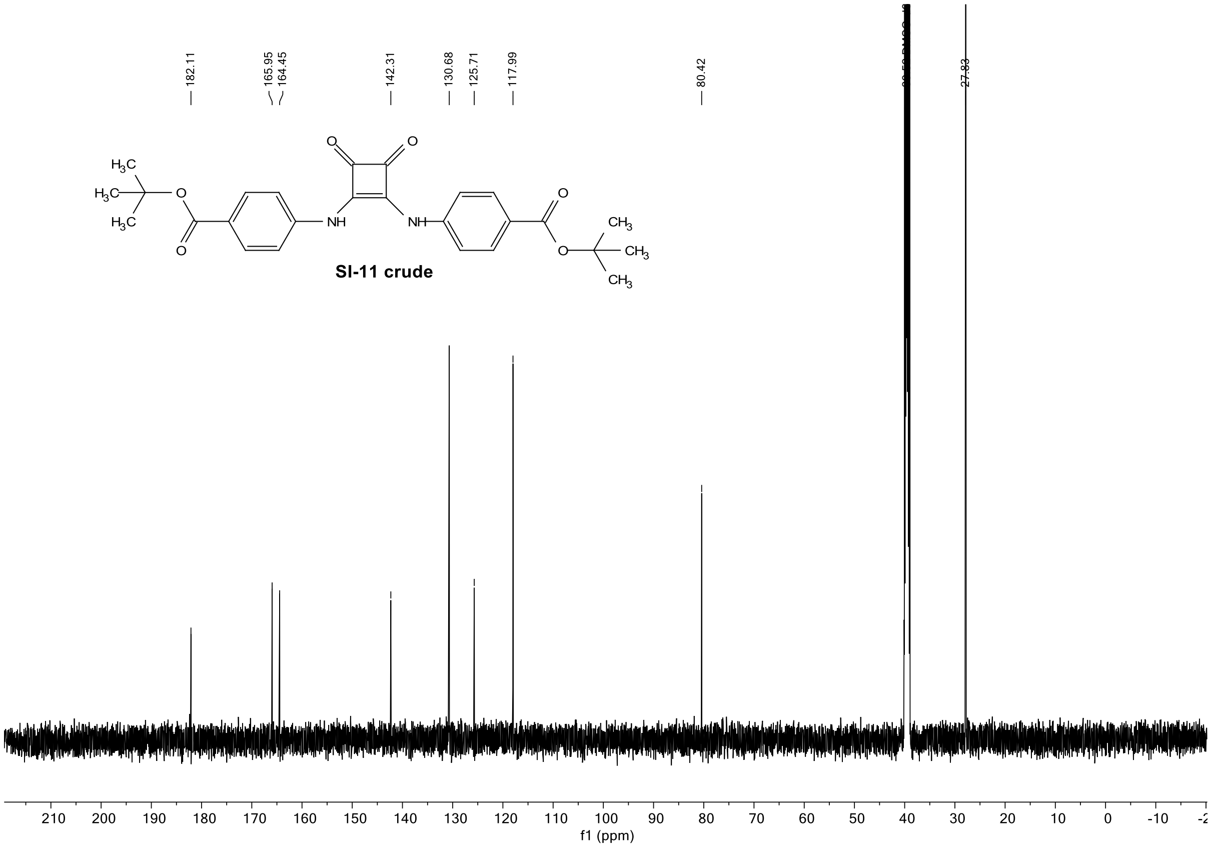

SI-11 crude
182.11
165.95
164.45
142.31
130.68
125.71
117.99
80.42
27.83
f1 (ppm)

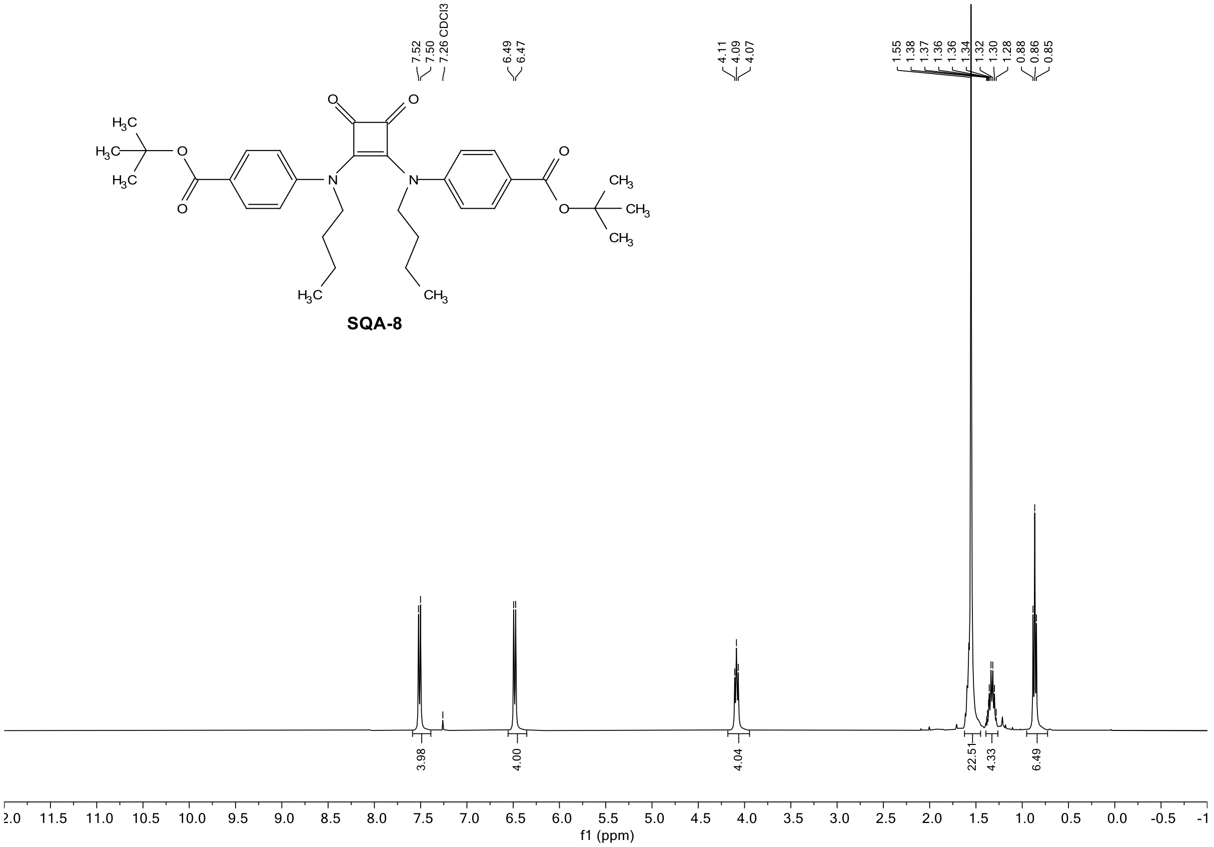
7.52
7.50
7.26 CDCl3
6.49
6.47
4.11
4.09
4.07
1.55
1.38
1.37
1.36
1.36
1.34
1.32
1.30
1.28
0.88
0.86
0.85
3.98
4.00
4.04
22.51
4.33
6.49
f1 (ppm)

SQA-8

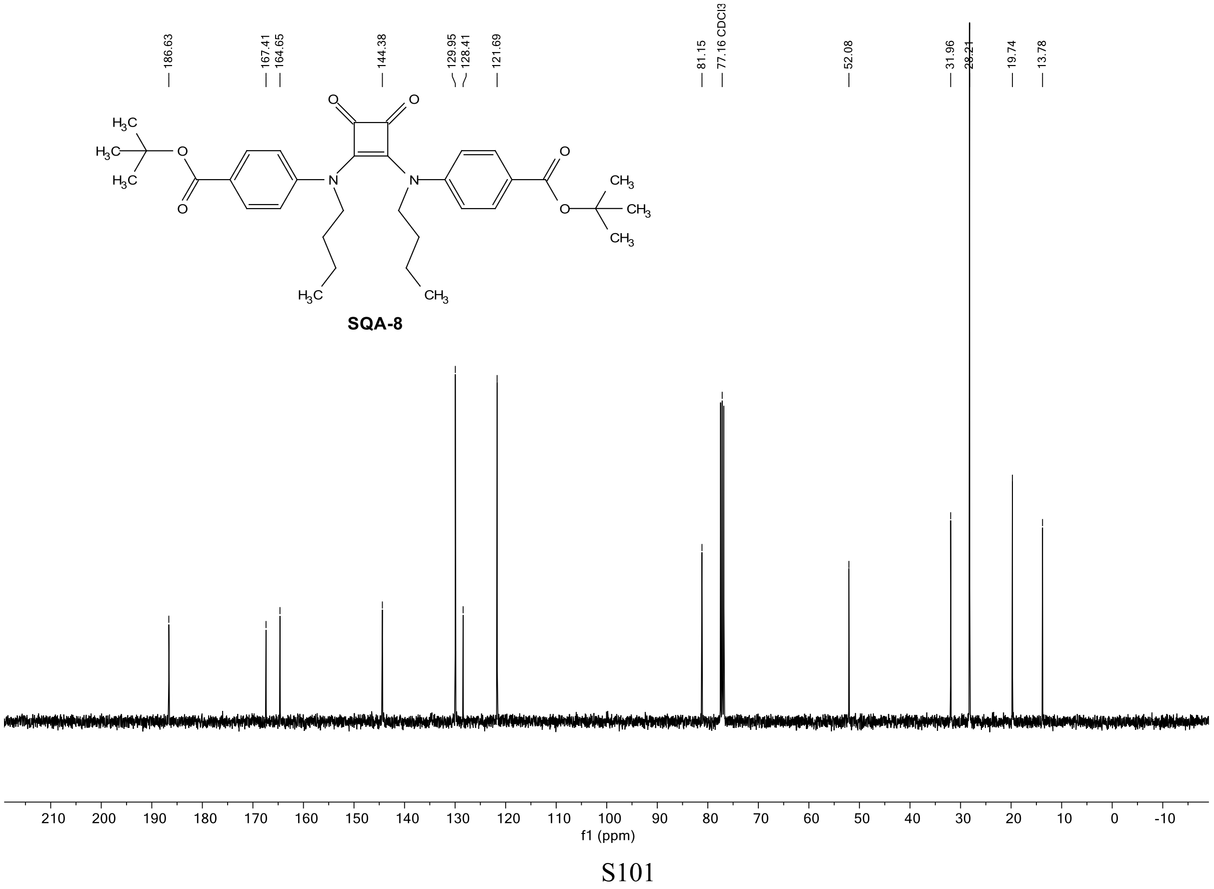
186.63
167.41
164.65
144.38
129.95
128.41
121.69
81.15
77.16 CDCl3
52.08
31.96
28.21
19.74
13.78
f1 (ppm)

SQA-8

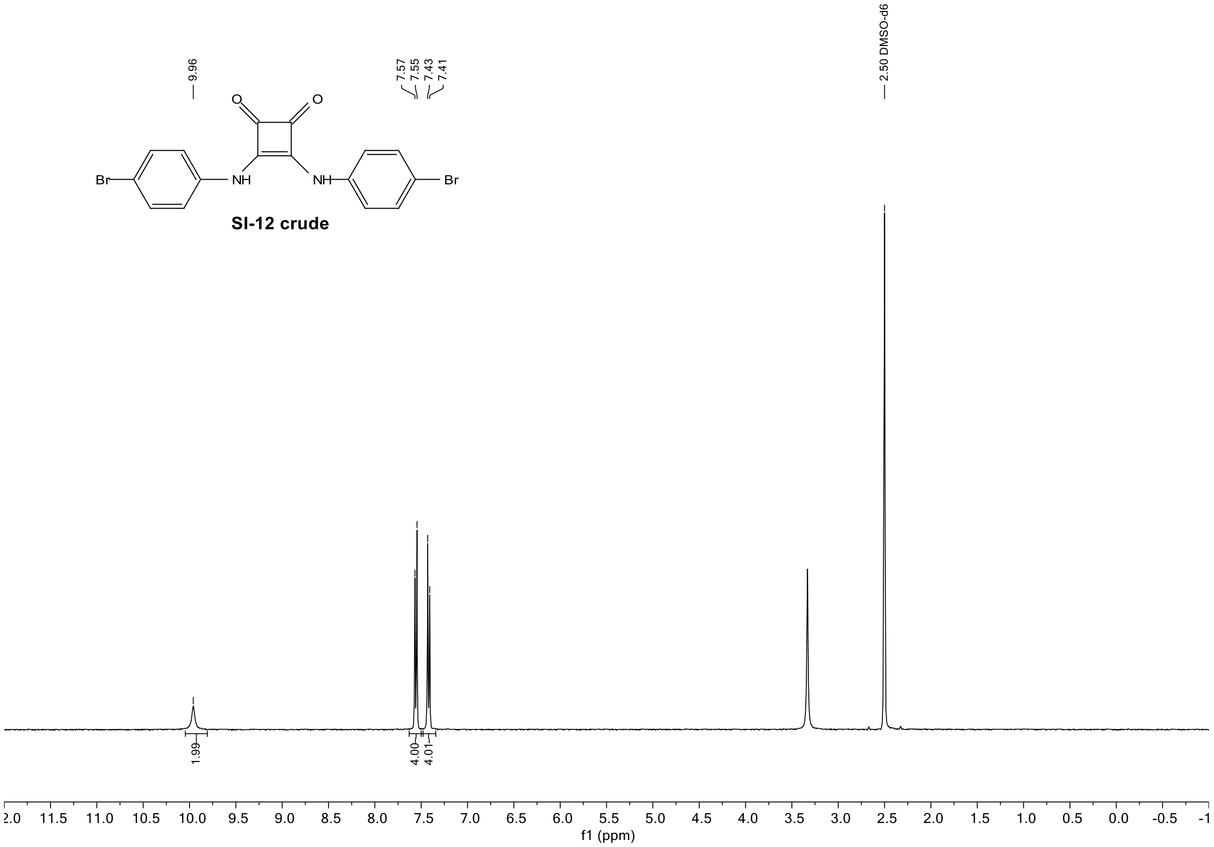

9.96
7.57
7.55
7.43
7.41
2.50 DMSO-d6
Br
NH
NH
Br
O
O
SI-12 crude
1.99
4.00
4.01
f1 (ppm)

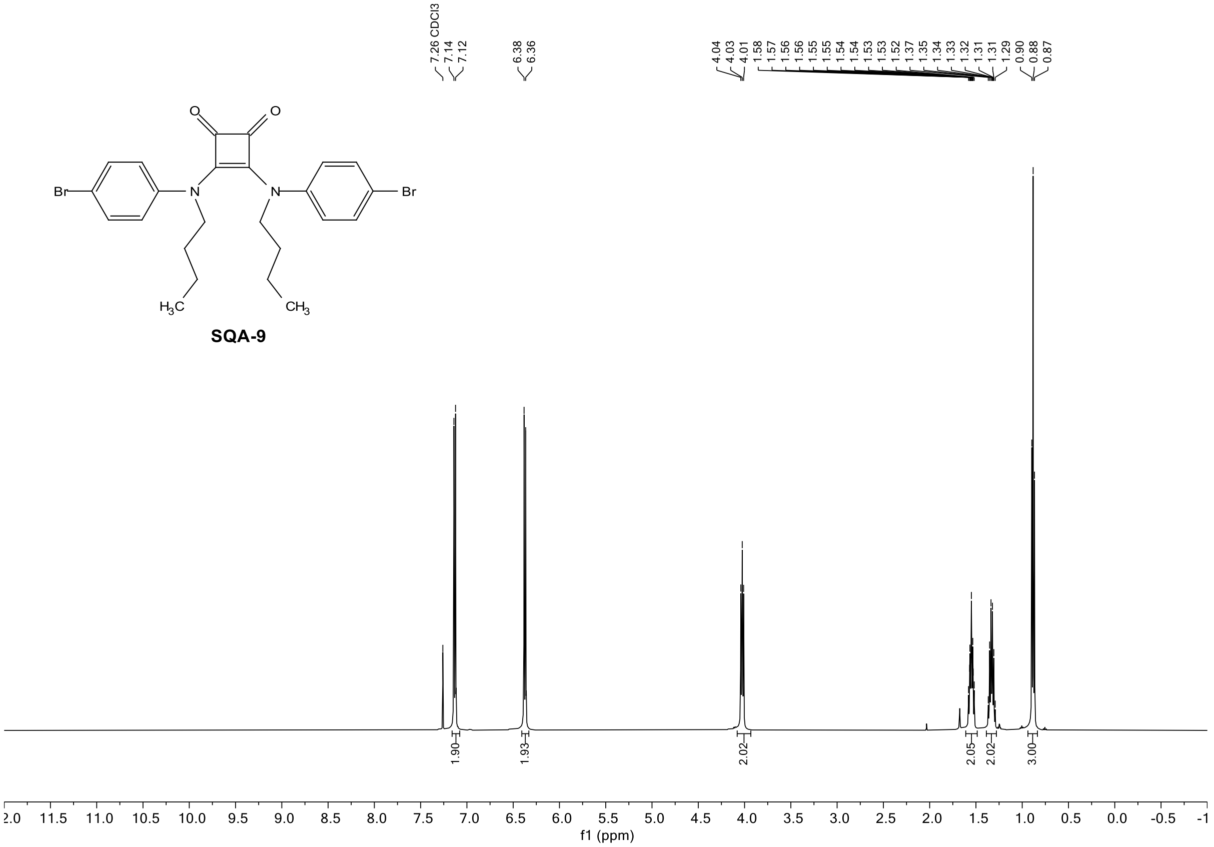

SQA-9

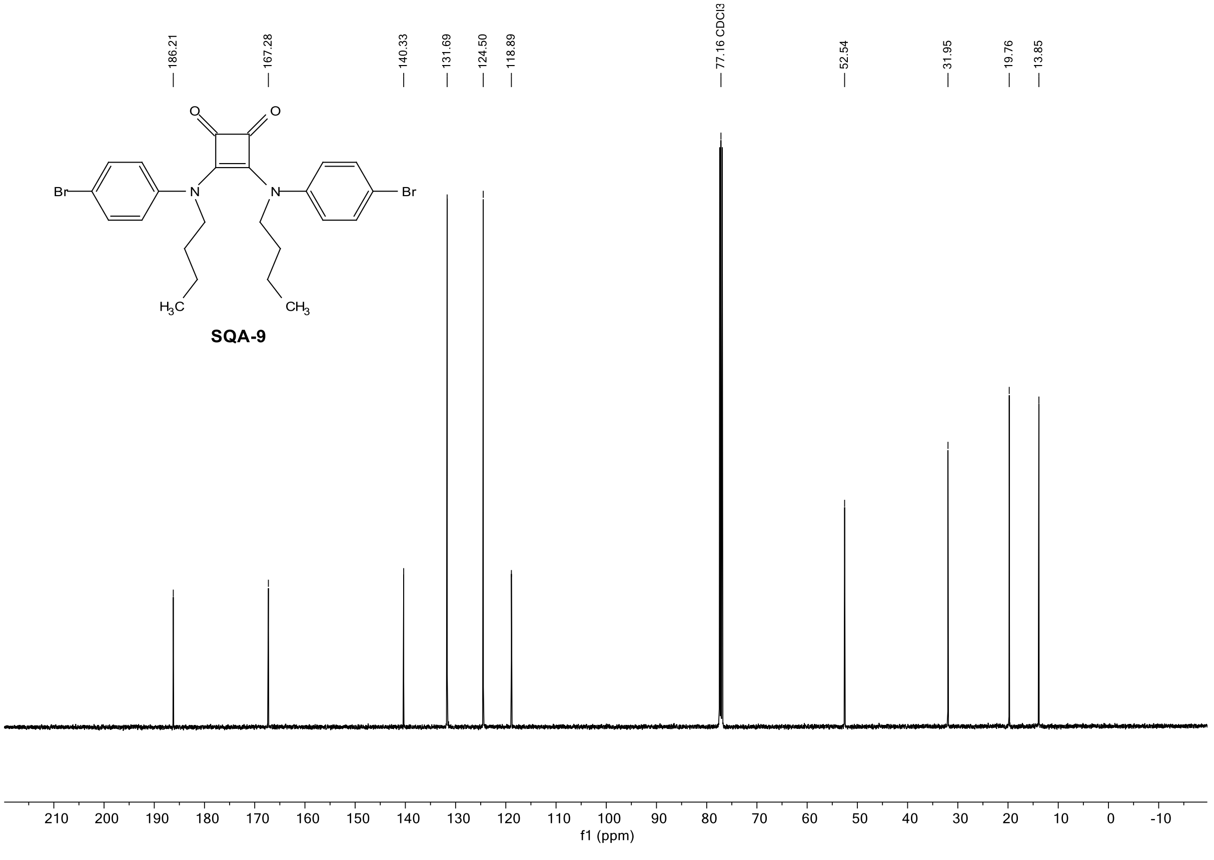

SQA-9

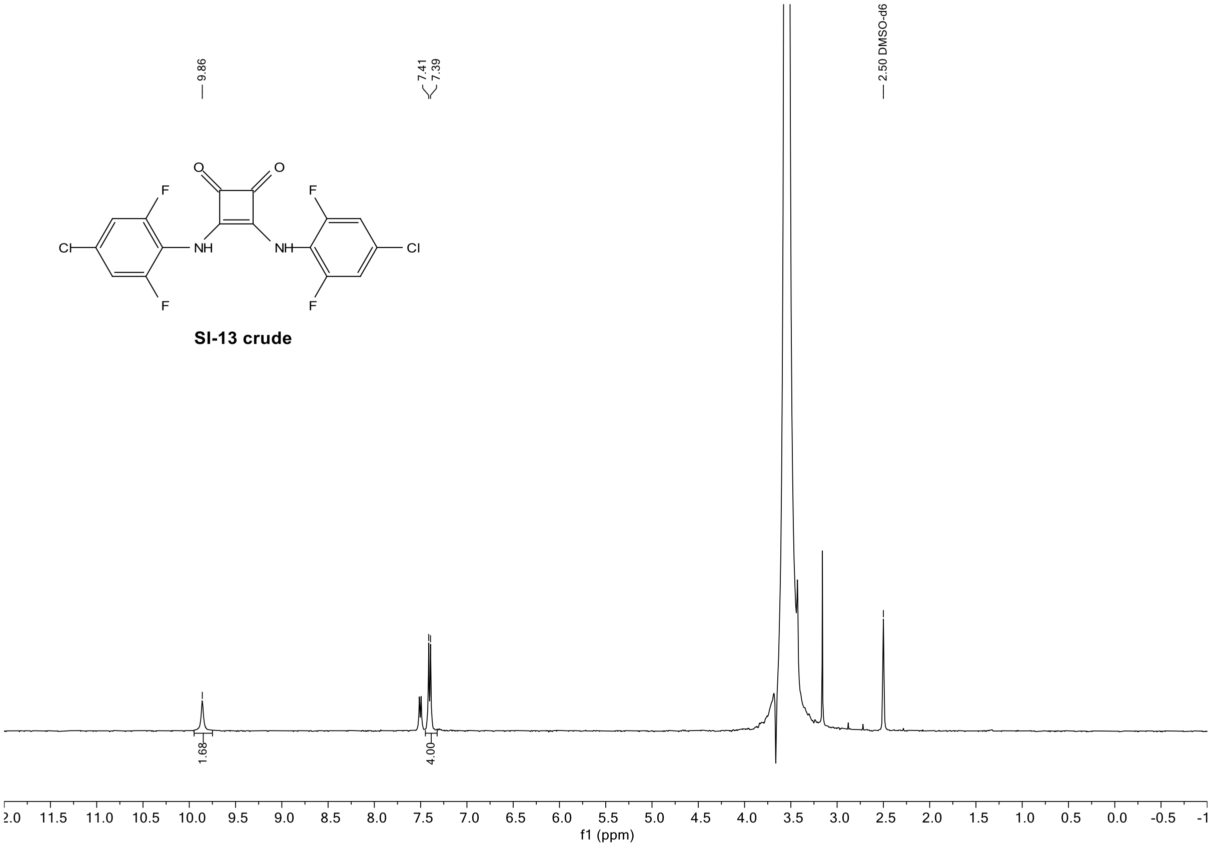
9.86
7.41
7.39
2.50 DMSO-d6
O
O
F
F
Cl
NH
NH
Cl
F
F
SI-13 crude
1.68
4.00
2.0 11.5 11.0 10.5 10.0 9.5 9.0 8.5 8.0 7.5 7.0 6.5 6.0 5.5 5.0 4.5 4.0 3.5 3.0 2.5 2.0 1.5 1.0 0.5 0.0 -0.5 -1
f1 (ppm)

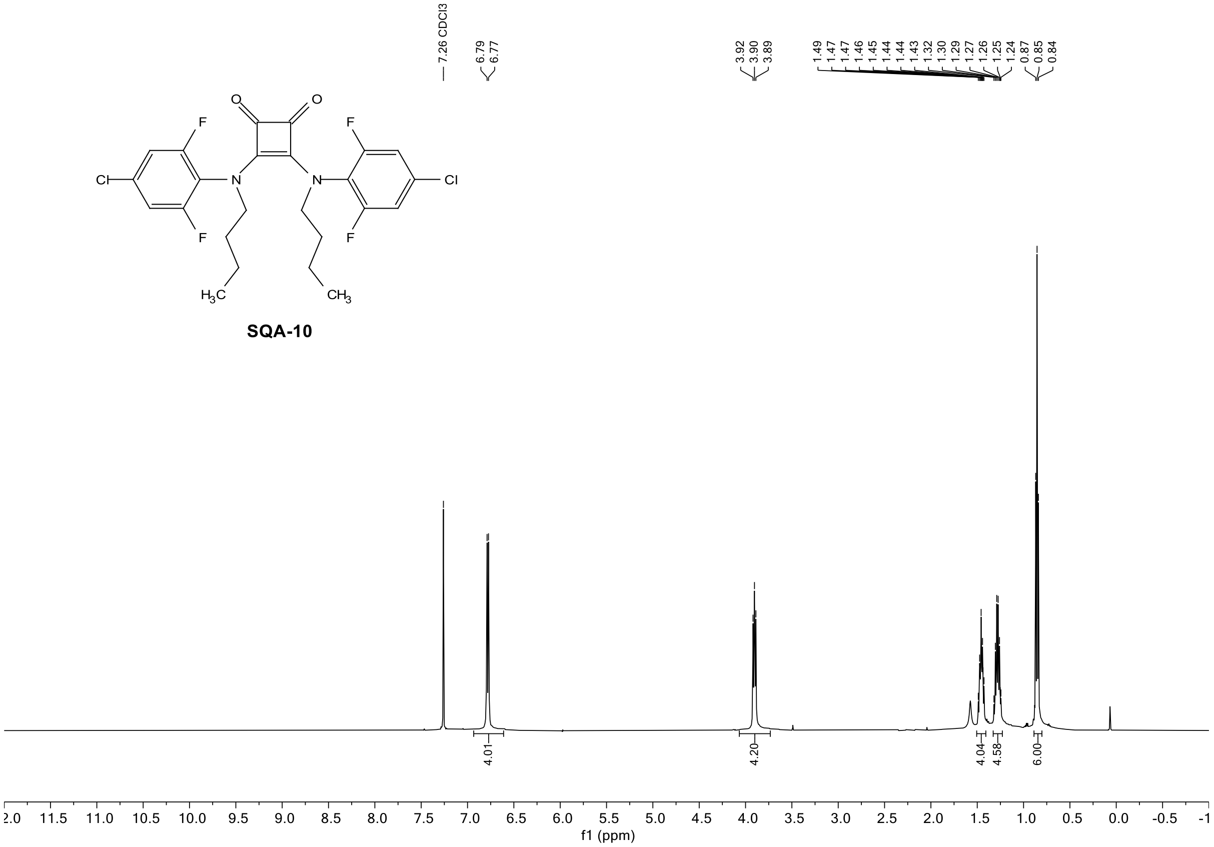

SQA-10

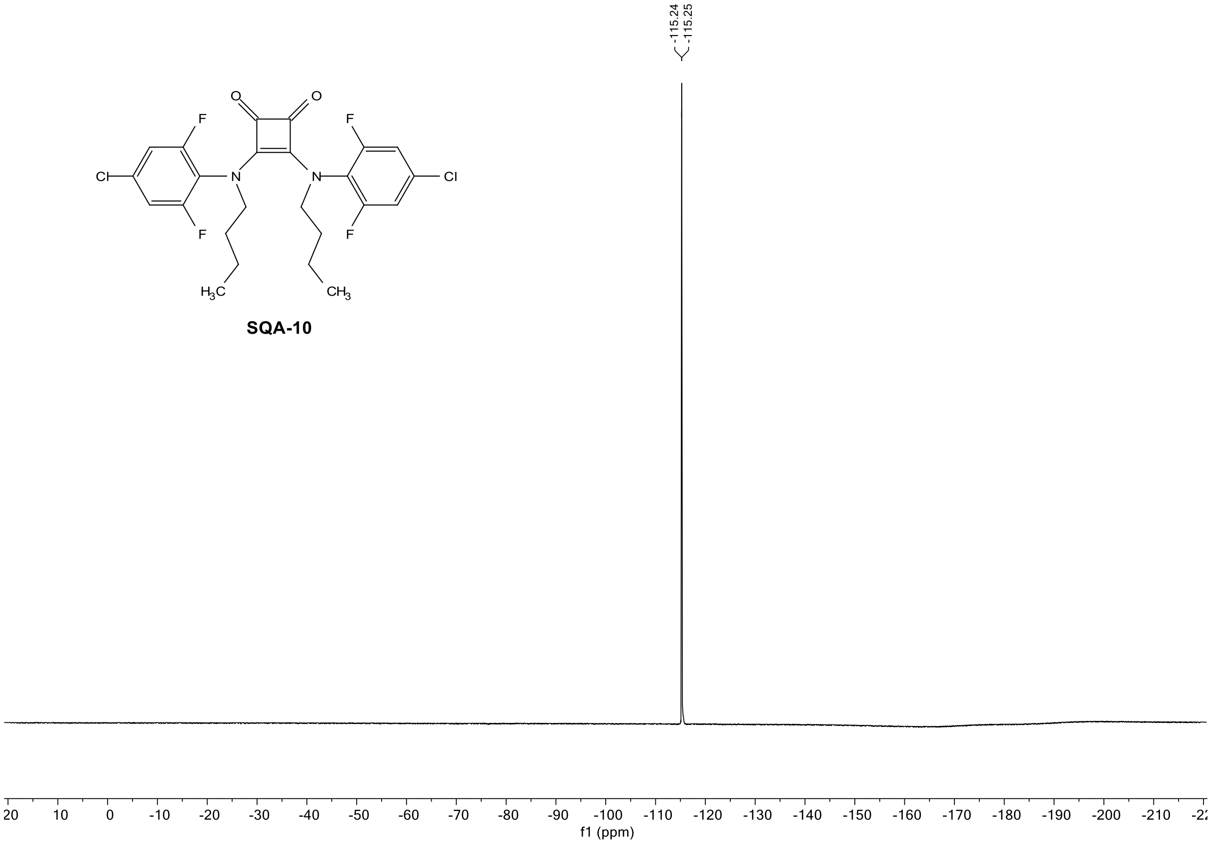

SQA-10

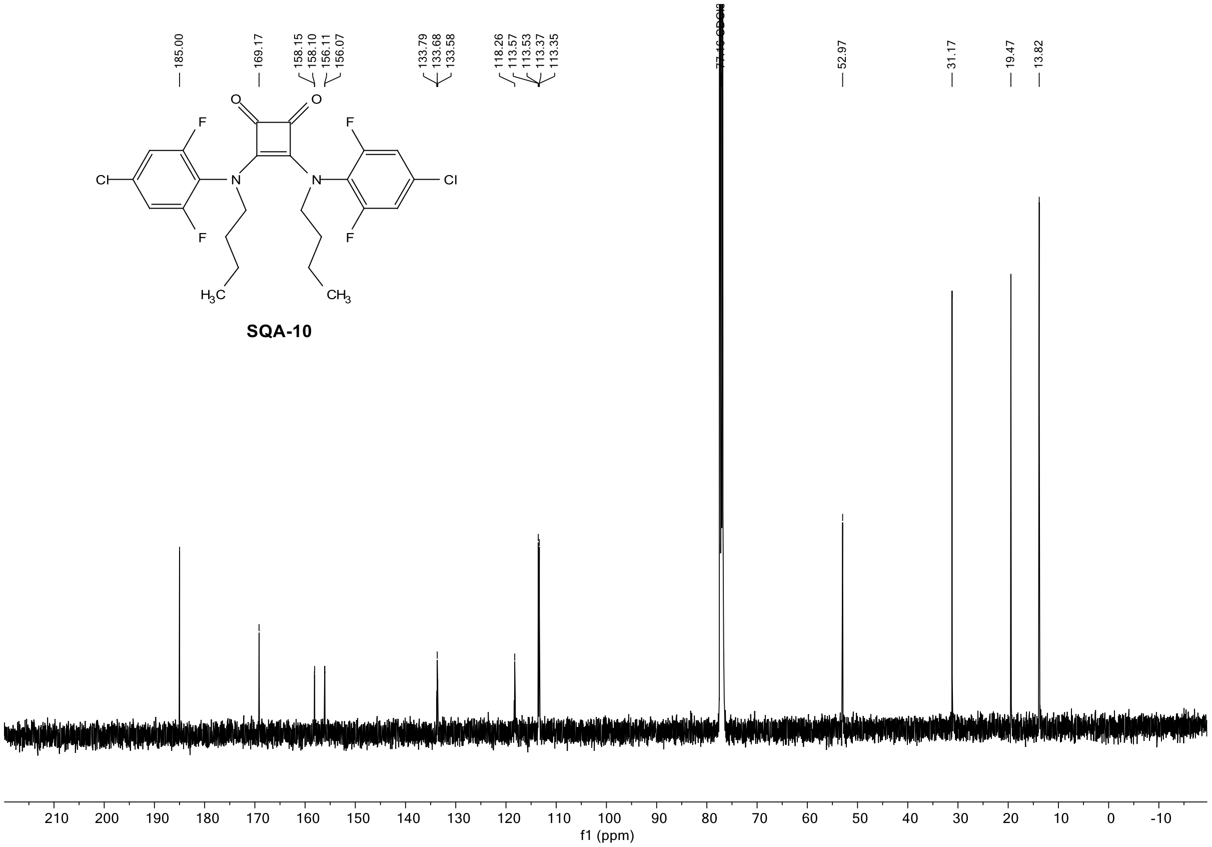
185.00
169.17
158.15
158.10
156.11
156.07
133.79
133.68
133.58
118.26
113.57
113.53
113.37
113.35
52.97
31.17
19.47
13.82
SQA-10
f1 (ppm)

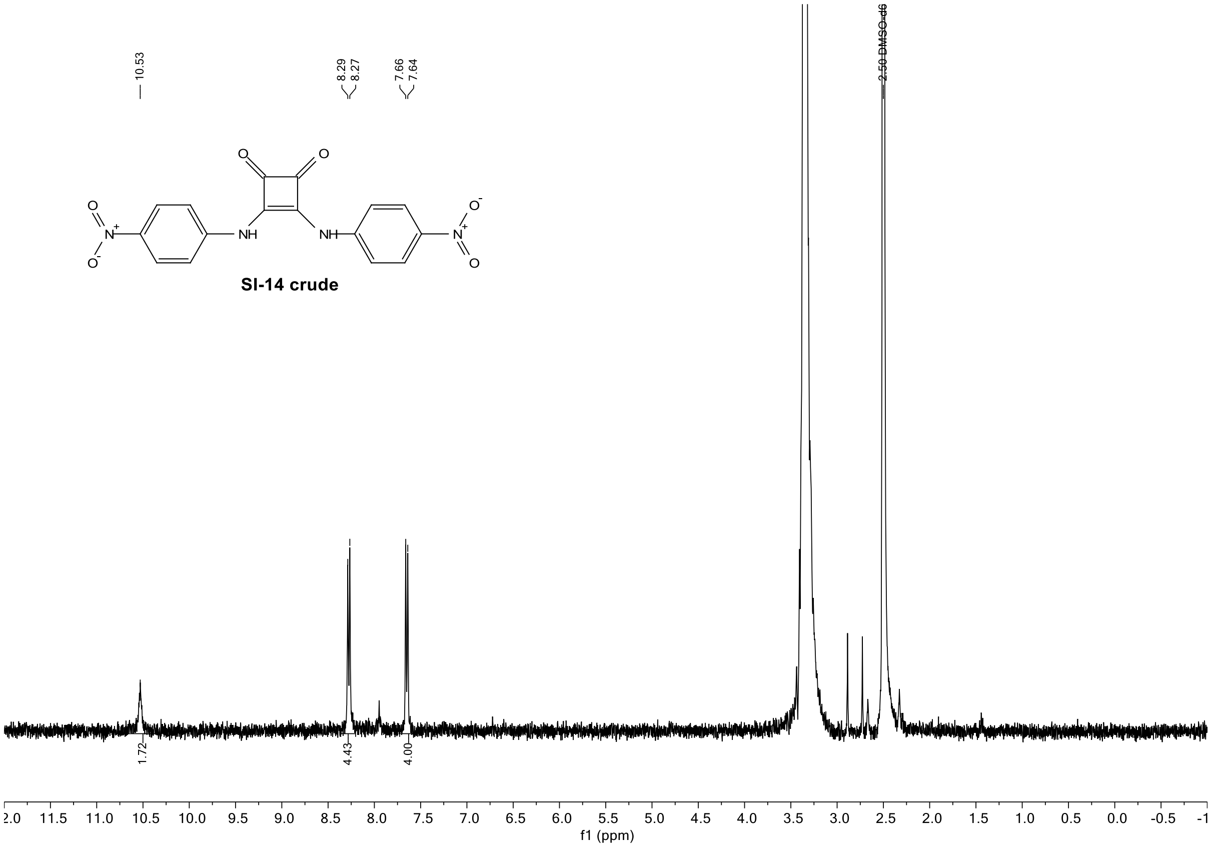
10.53
8.29
8.27
7.66
7.64
2.50 DMSO-d6
SI-14 crude
1.72
4.43
4.00
f1 (ppm)

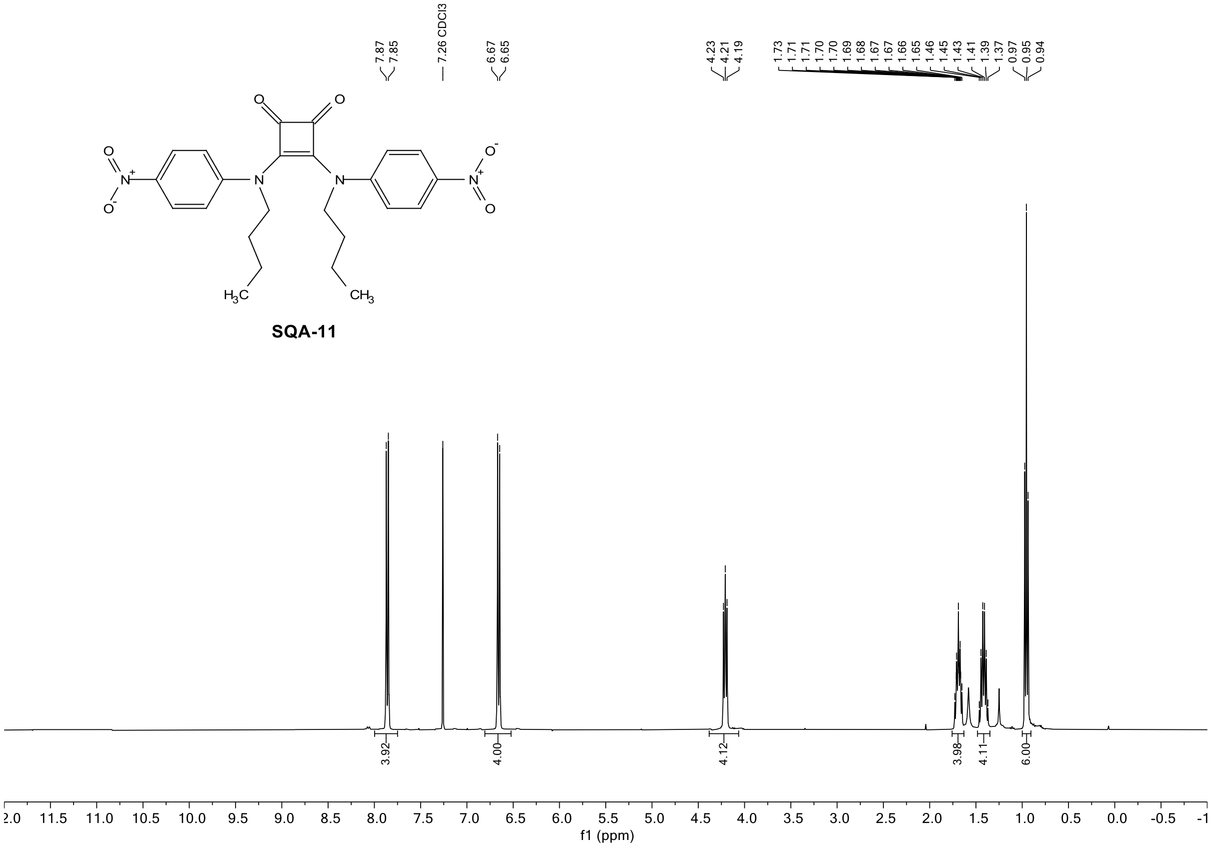
7.87
7.85
7.26 CDCl3
6.67
6.65
4.23
4.21
4.19
1.73
1.71
1.71
1.70
1.70
1.69
1.68
1.67
1.67
1.66
1.65
1.46
1.45
1.43
1.41
1.39
1.37
0.97
0.95
0.94
SQA-11
3.92
4.00
4.12
3.98
4.11
6.00
f1 (ppm)

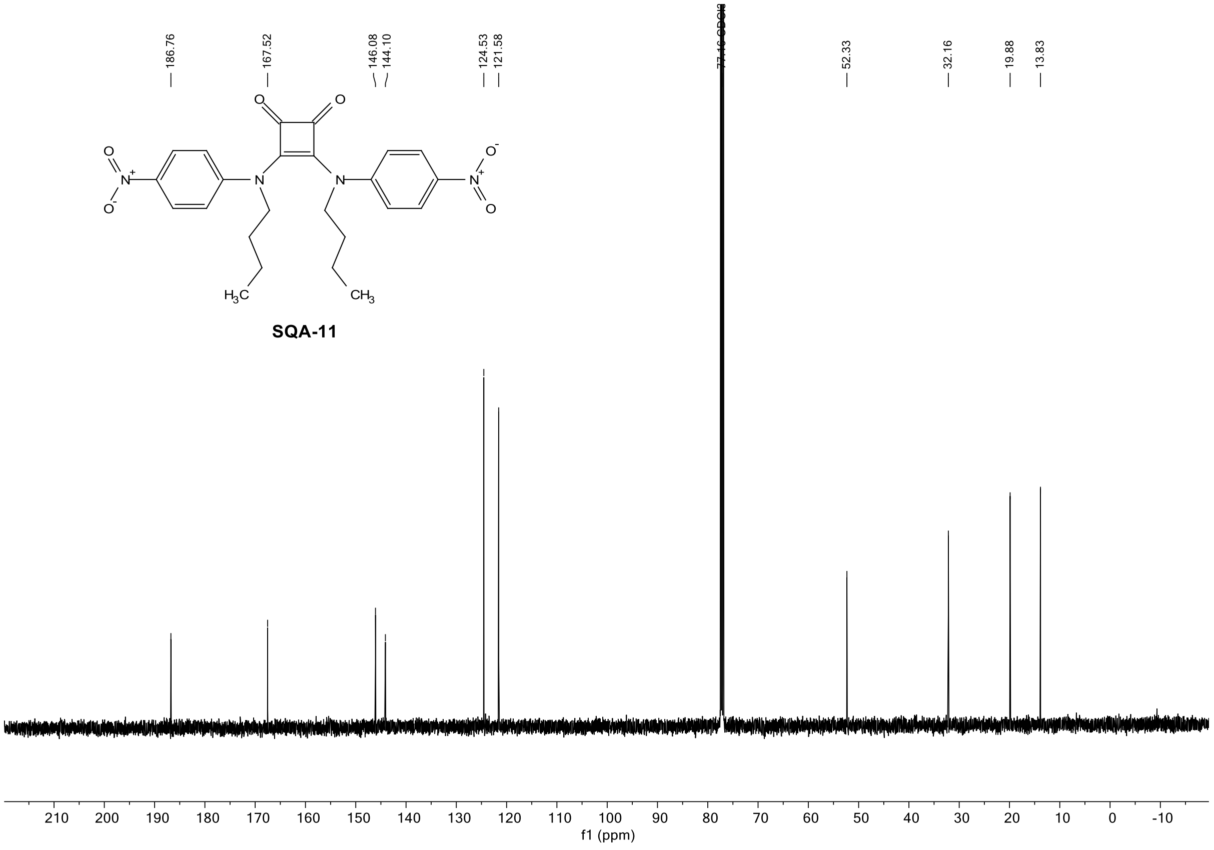
186.76
167.52
146.08
144.10
124.53
121.58
52.33
32.16
19.88
13.83
SQA-11
f1 (ppm)

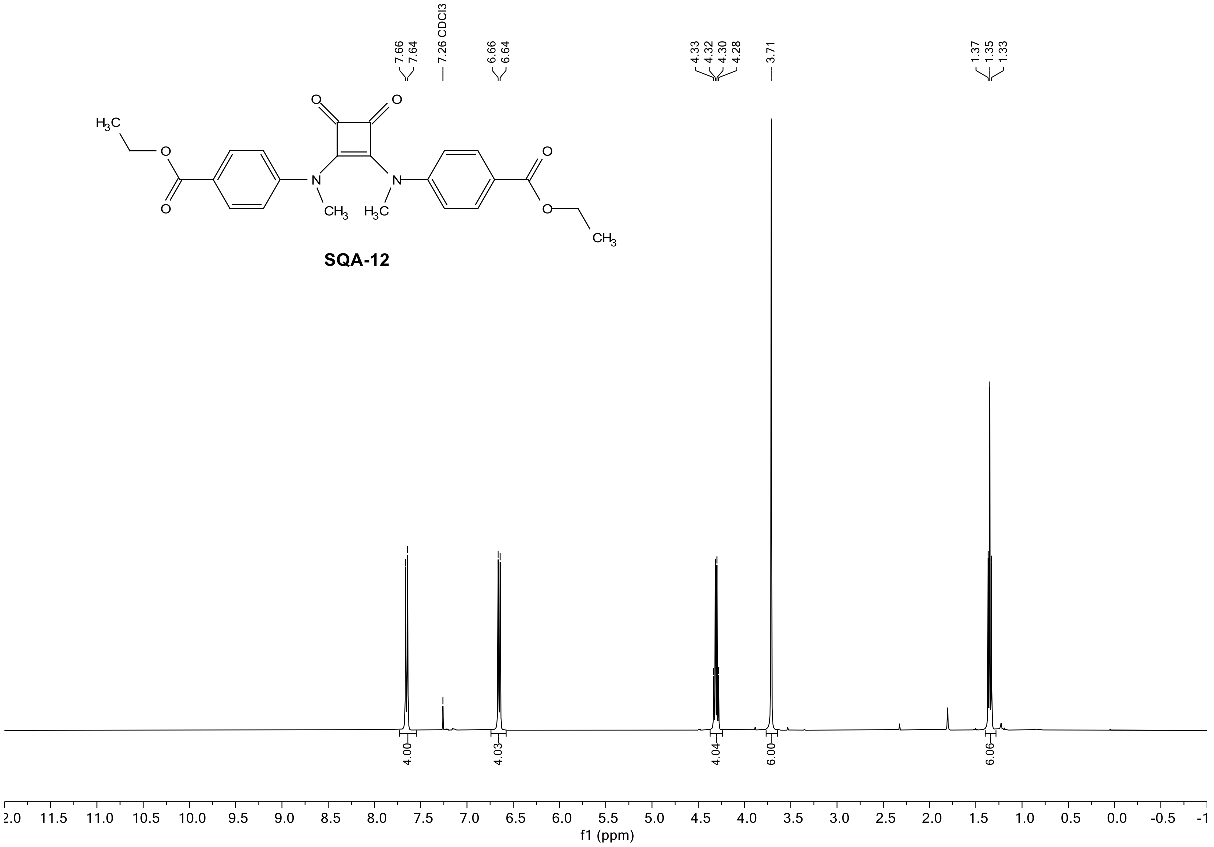
7.66
7.64
7.26 CDCl3
6.66
6.64
4.33
4.32
4.30
4.28
3.71
1.37
1.35
1.33
SQA-12
4.00
4.03
4.04
6.00
6.06
f1 (ppm)

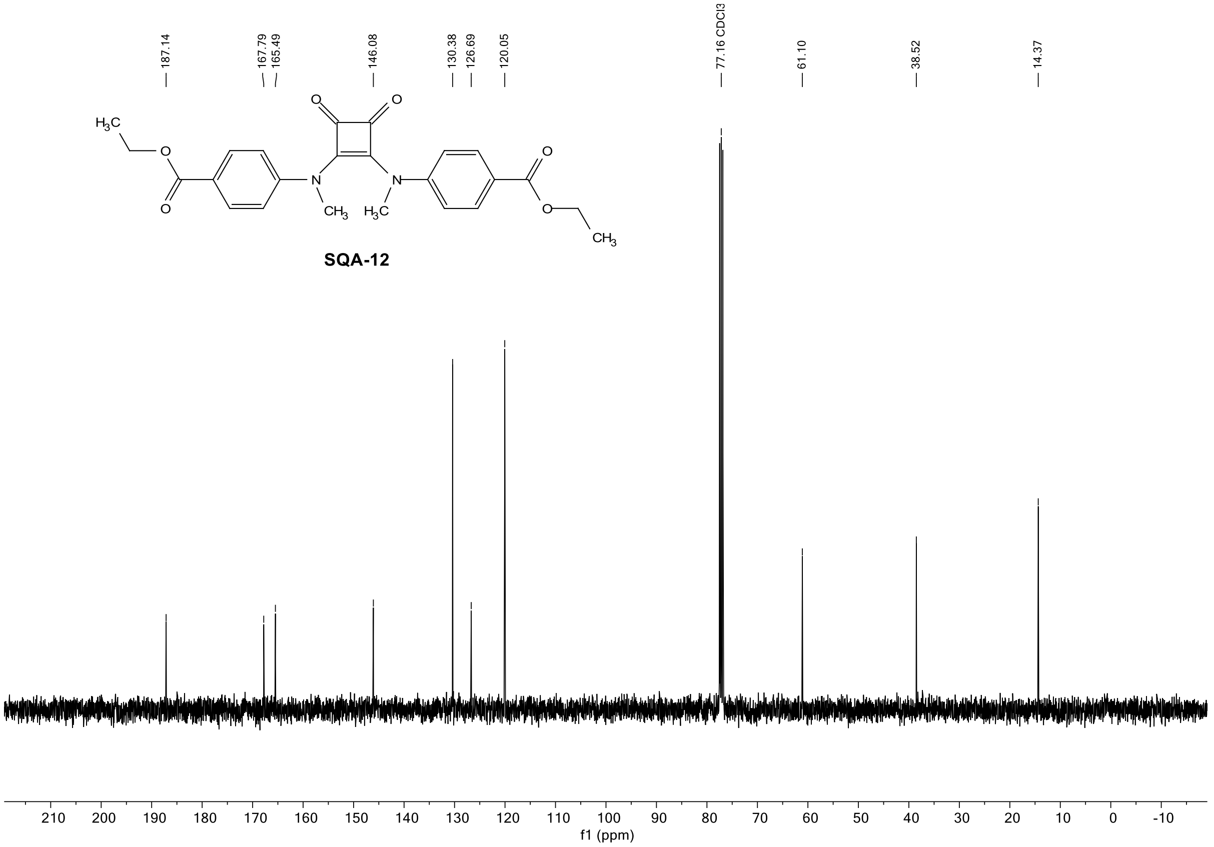
187.14
167.79
165.49
146.08
130.38
126.69
120.05
77.16 CDCl3
61.10
38.52
14.37
SQA-12
f1 (ppm)

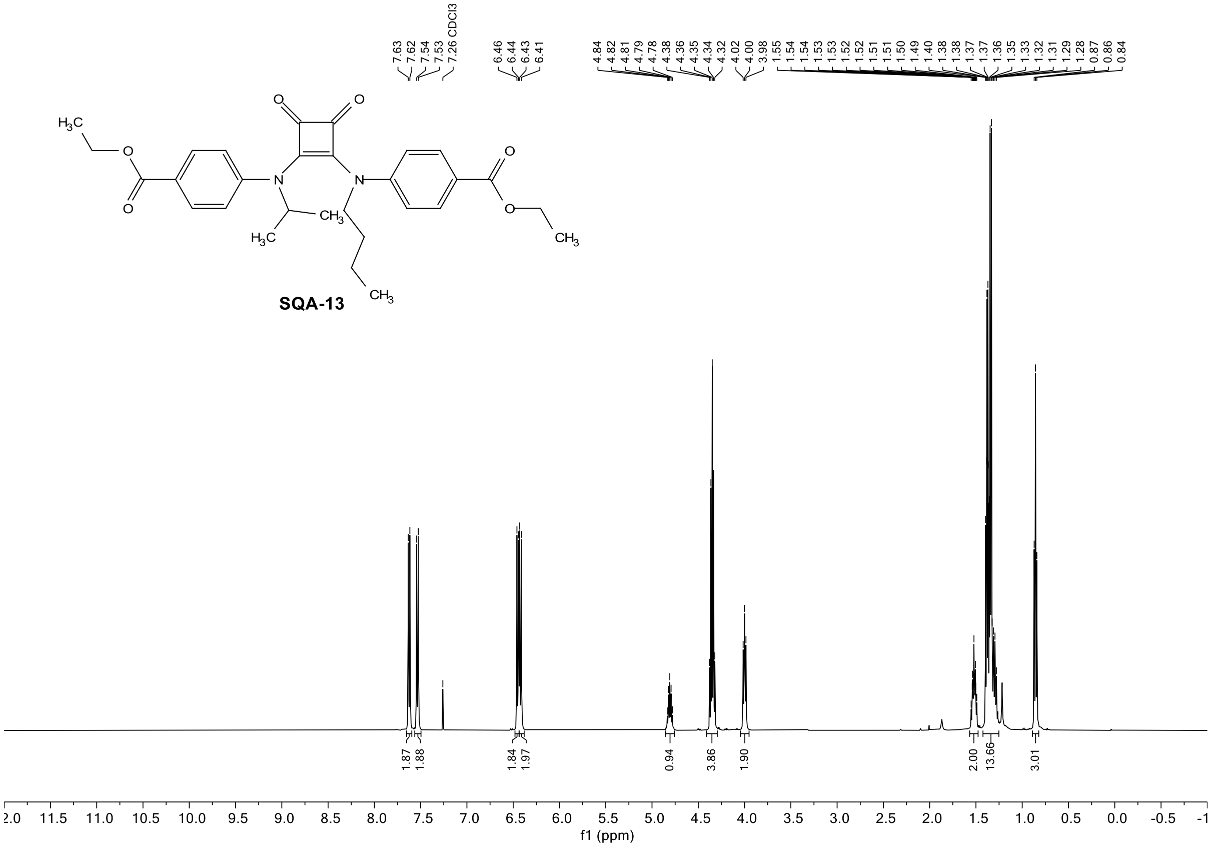
7.63
7.62
7.54
7.53
7.26 CDCl3
6.46
6.44
6.43
6.41
4.84
4.82
4.81
4.79
4.78
4.38
4.36
4.35
4.34
4.32
4.02
4.00
3.98
1.55
1.54
1.54
1.53
1.53
1.52
1.52
1.51
1.51
1.50
1.49
1.40
1.38
1.38
1.38
1.37
1.37
1.36
1.35
1.33
1.32
1.31
1.29
0.87
0.86
0.84
SQA-13
1.87
1.88
1.84
1.97
0.94
3.86
1.90
2.00
13.66
3.01
f1 (ppm)

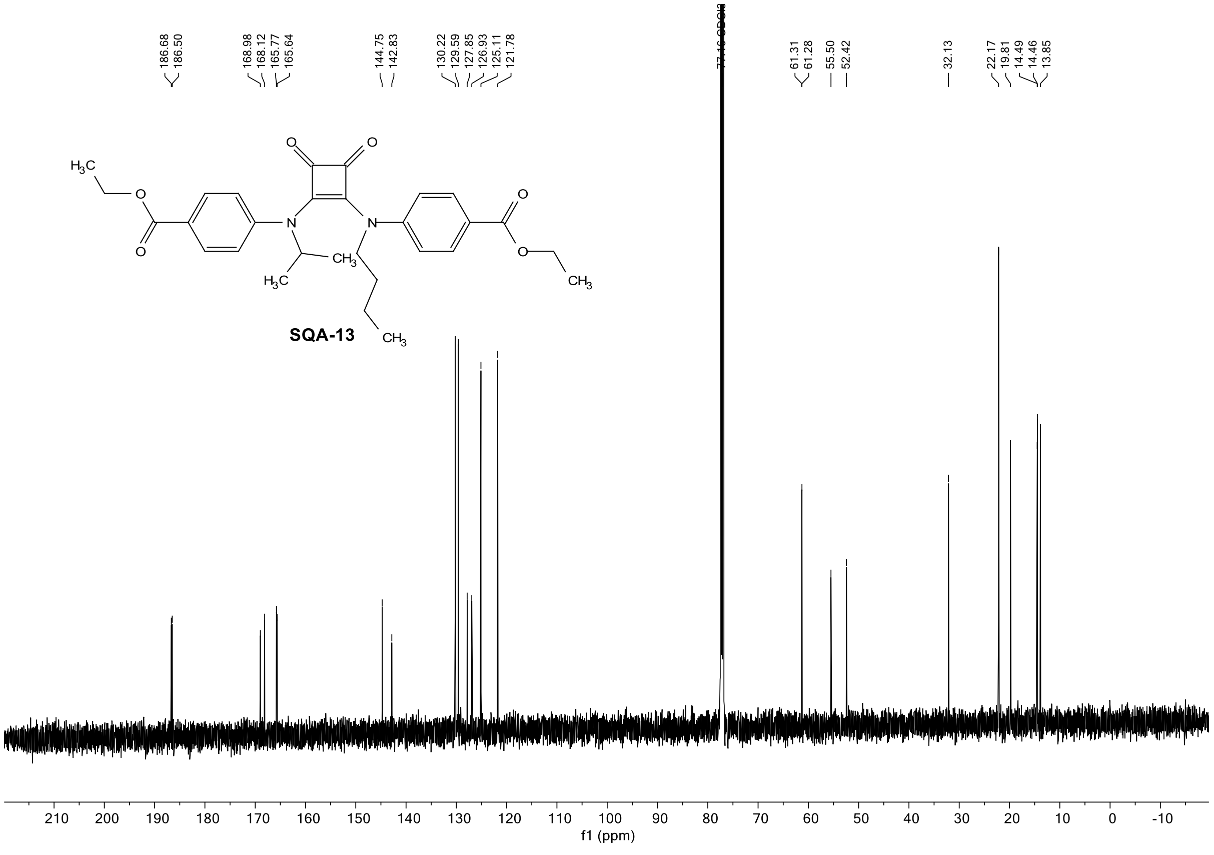
186.68
186.50
168.98
168.12
165.77
165.64
144.75
142.83
130.22
129.59
127.85
126.93
125.11
121.78
61.31
61.28
55.50
52.42
32.13
22.17
19.81
14.49
14.46
13.85
SQA-13
f1 (ppm)

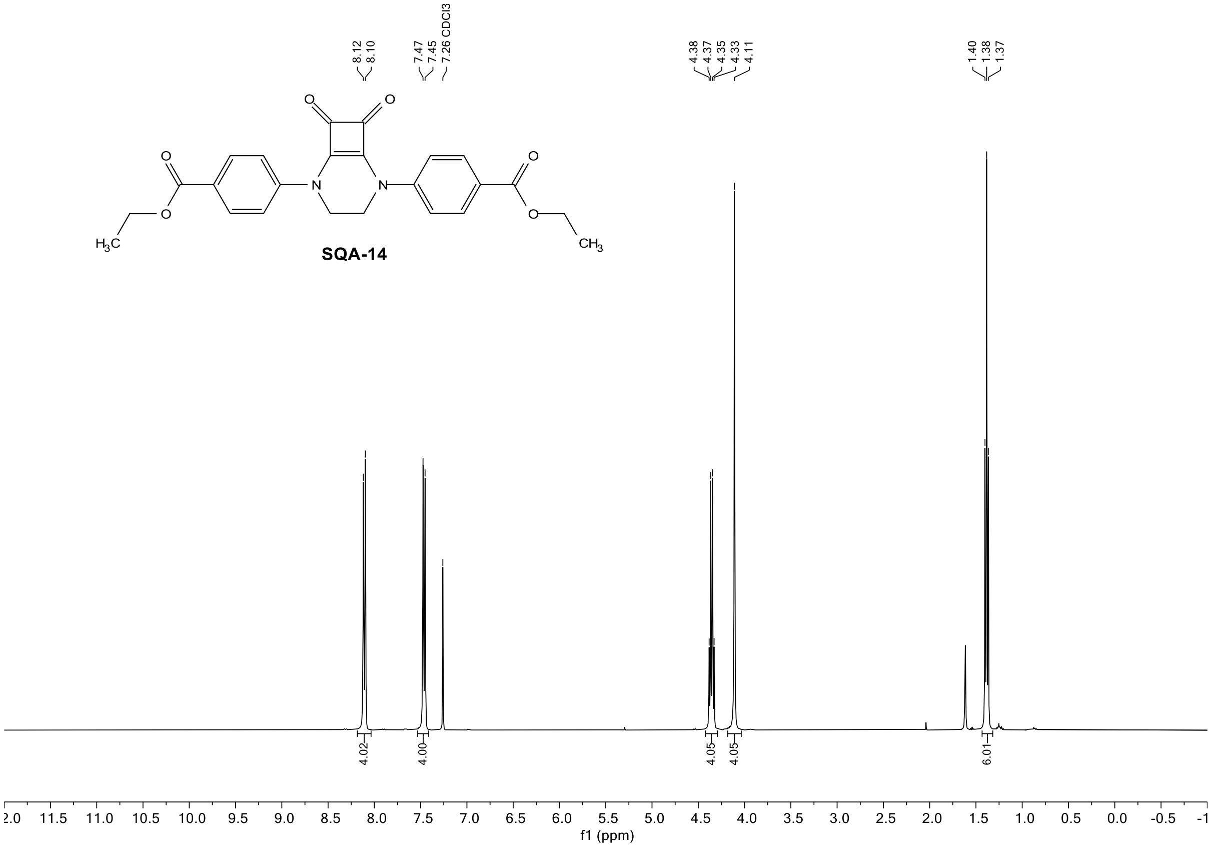
H3C
CH3
SQA-14
8.12
8.10
7.47
7.45
7.26 CDCl3
4.38
4.37
4.35
4.33
4.11
1.40
1.38
1.37
4.02
4.00
4.05
4.05
6.01
f1 (ppm)

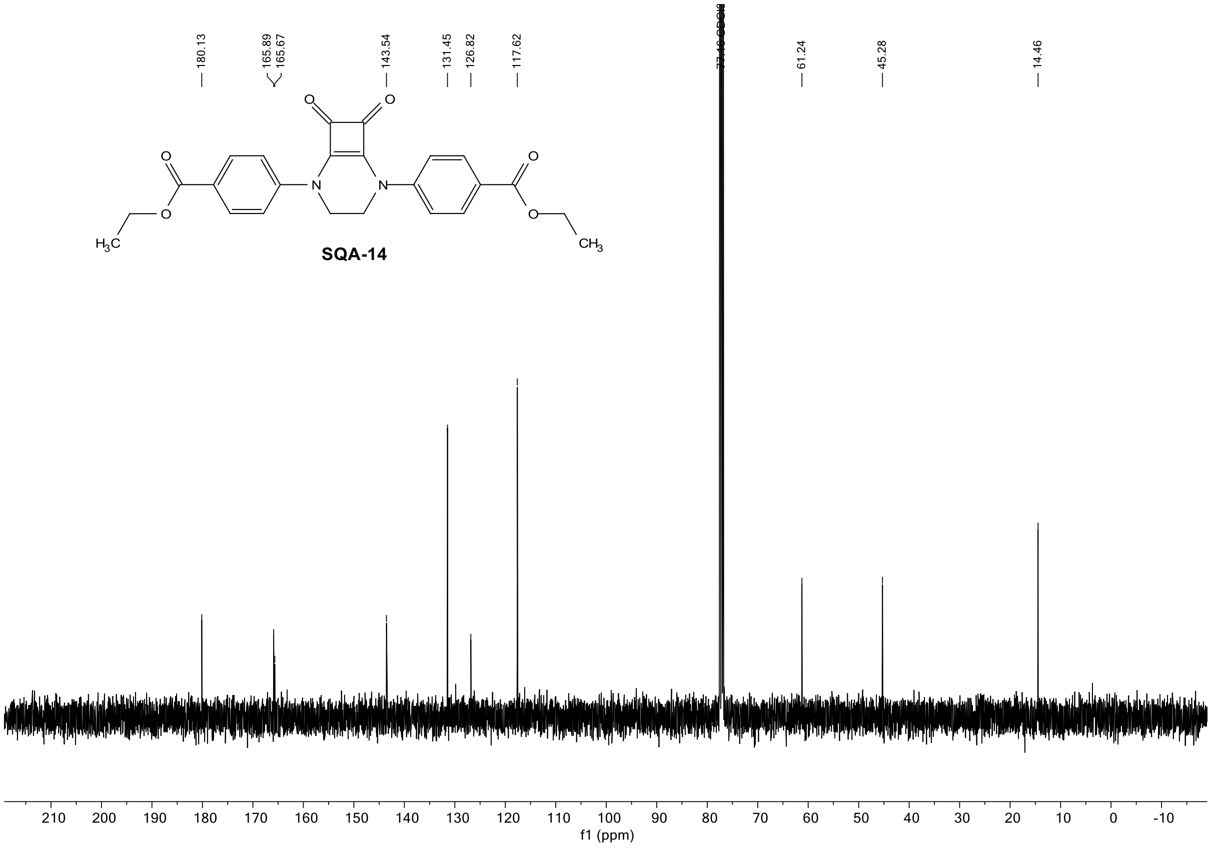
H3C
CH3
SQA-14
180.13
165.89
165.67
143.54
131.45
126.82
117.62
61.24
45.28
14.46
f1 (ppm)

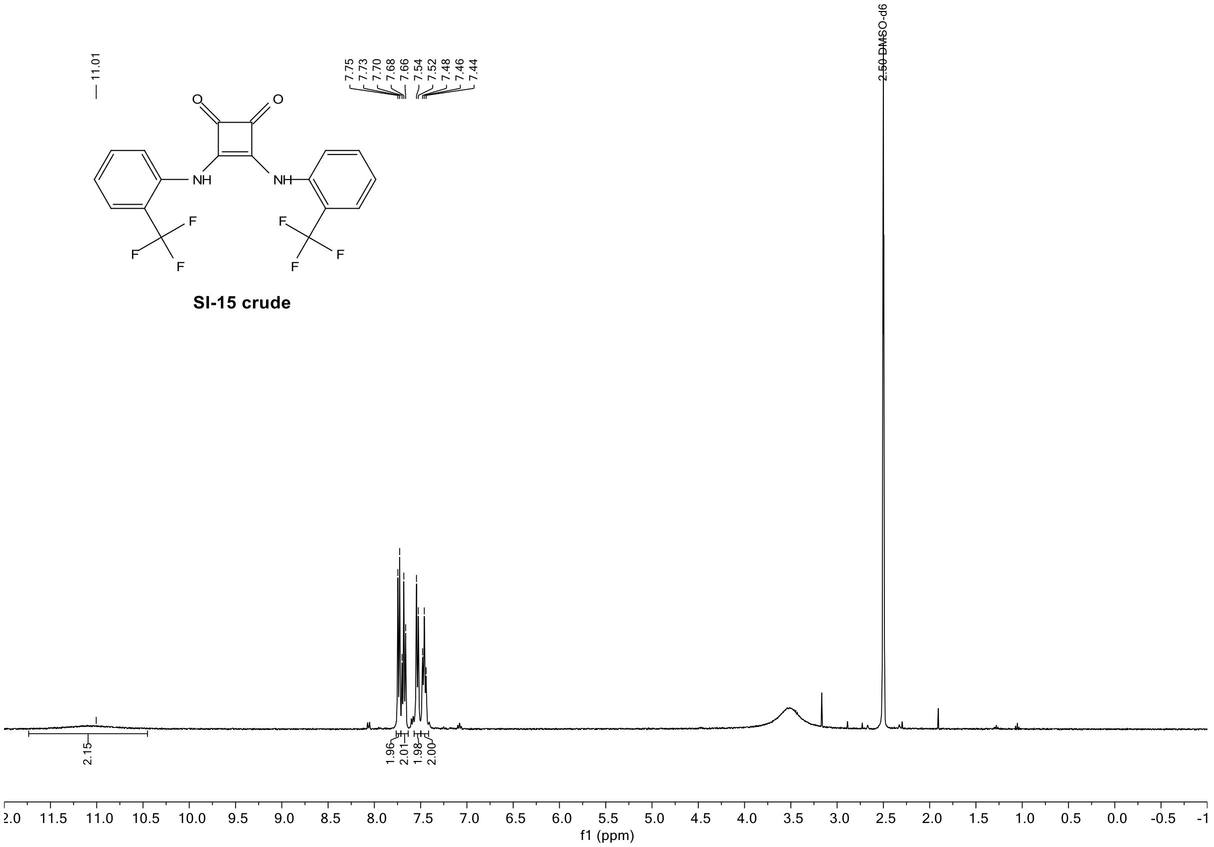

11.01
7.75
7.73
7.70
7.68
7.66
7.54
7.52
7.48
7.46
7.44
2.50 DMSO-d6
O
O
NH
NH
F
F
F
F
F
F
2.15
1.96
2.01
1.98
2.00
f1 (ppm)

SI-15 crude

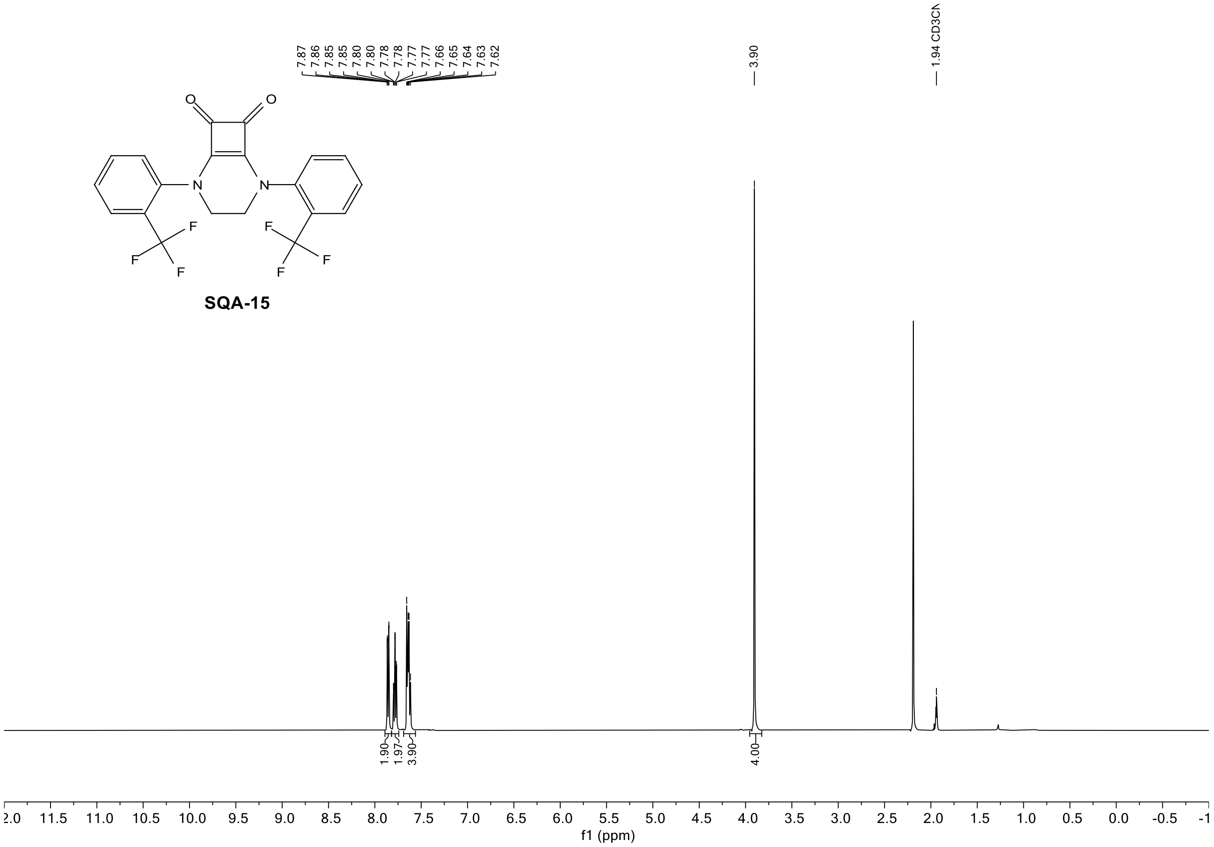
7.87
7.86
7.85
7.85
7.80
7.80
7.78
7.78
7.77
7.77
7.66
7.65
7.64
7.63
7.62
3.90
1.94 CD3CN
SQA-15
1.90
1.97
3.90
4.00
f1 (ppm)

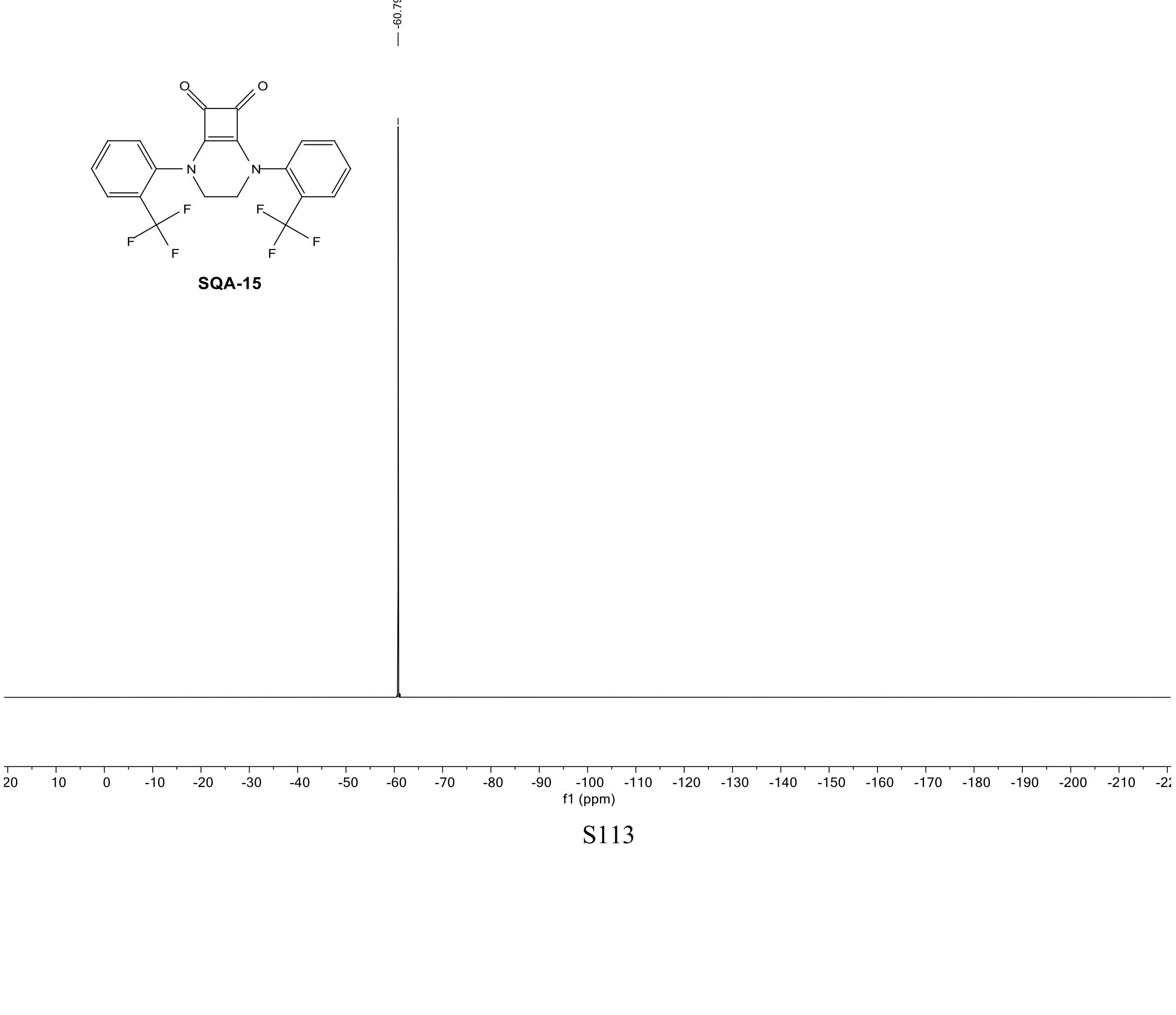
-60.79
SQA-15
f1 (ppm)

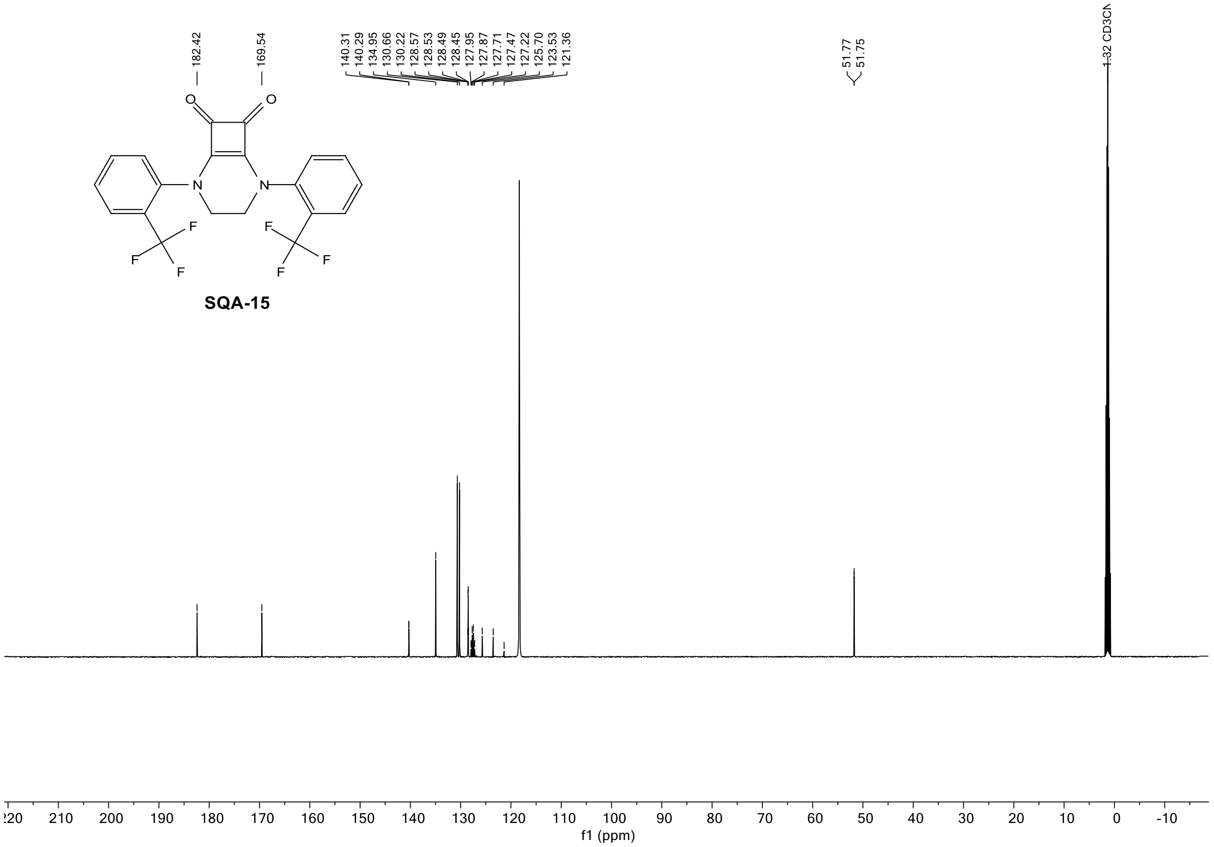
182.42
169.54
140.31
140.29
134.95
130.66
130.22
128.57
128.53
128.49
128.45
127.95
127.87
127.71
127.47
127.22
125.70
123.53
121.36
51.77
51.75
1.32 CD3CN
SQA-15
f1 (ppm)

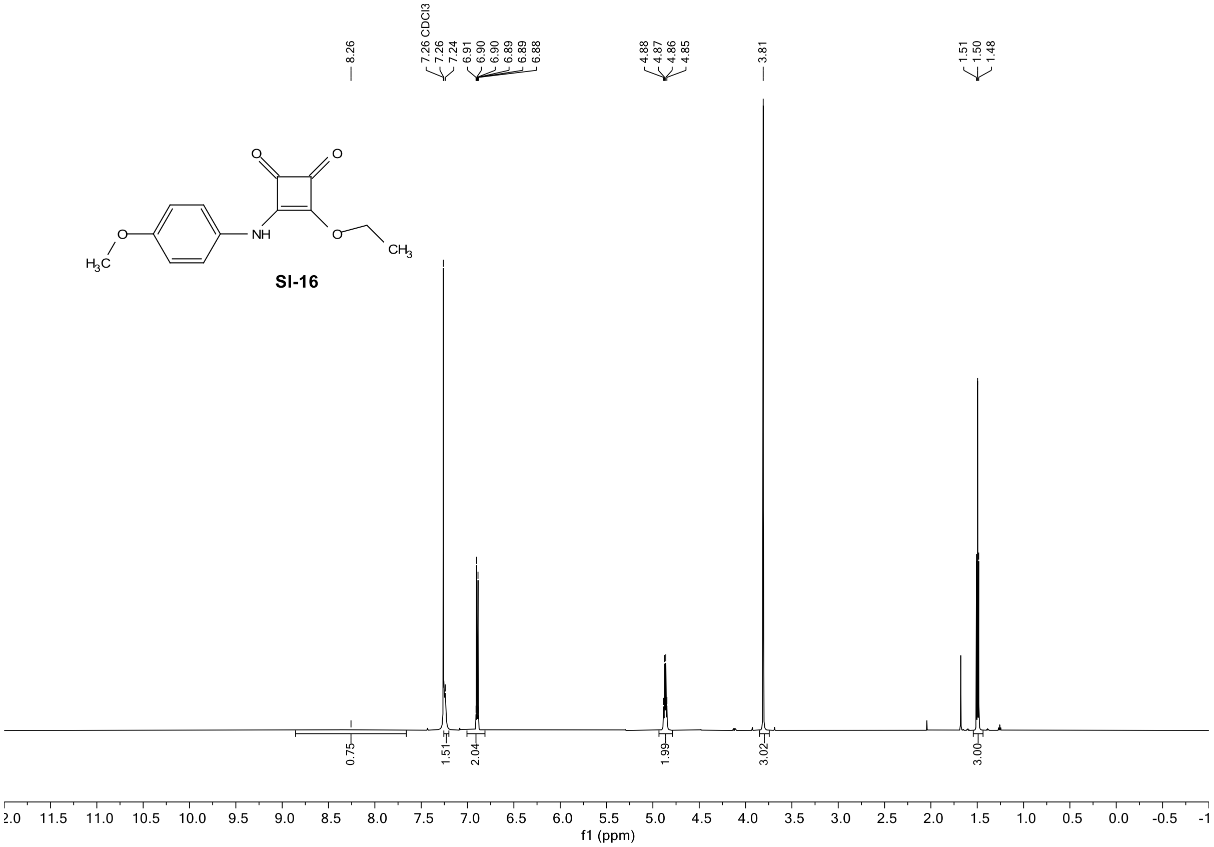
8.26
7.26 CDCl3
7.26
7.24
6.91
6.90
6.90
6.89
6.89
6.88
4.88
4.87
4.86
4.85
3.81
1.51
1.50
1.48
0.75
1.51
2.04
1.99
3.02
3.00
O
O
O
NH
O
CH3
H3C
SI-16
f1 (ppm)

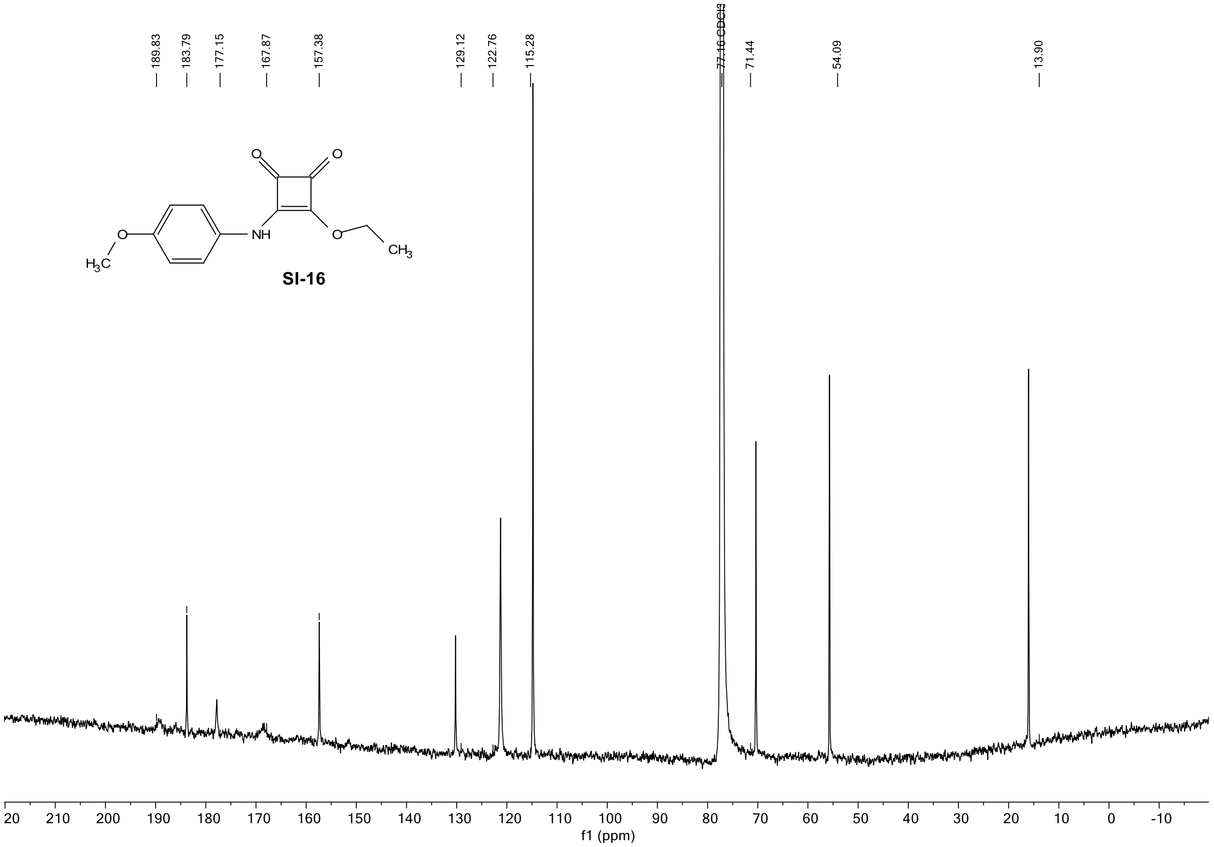
189.83
183.79
177.15
167.87
157.38
129.12
122.76
115.28
77.16 CDCl3
71.44
54.09
13.90
O
O
O
NH
O
CH3
H3C
SI-16
f1 (ppm)

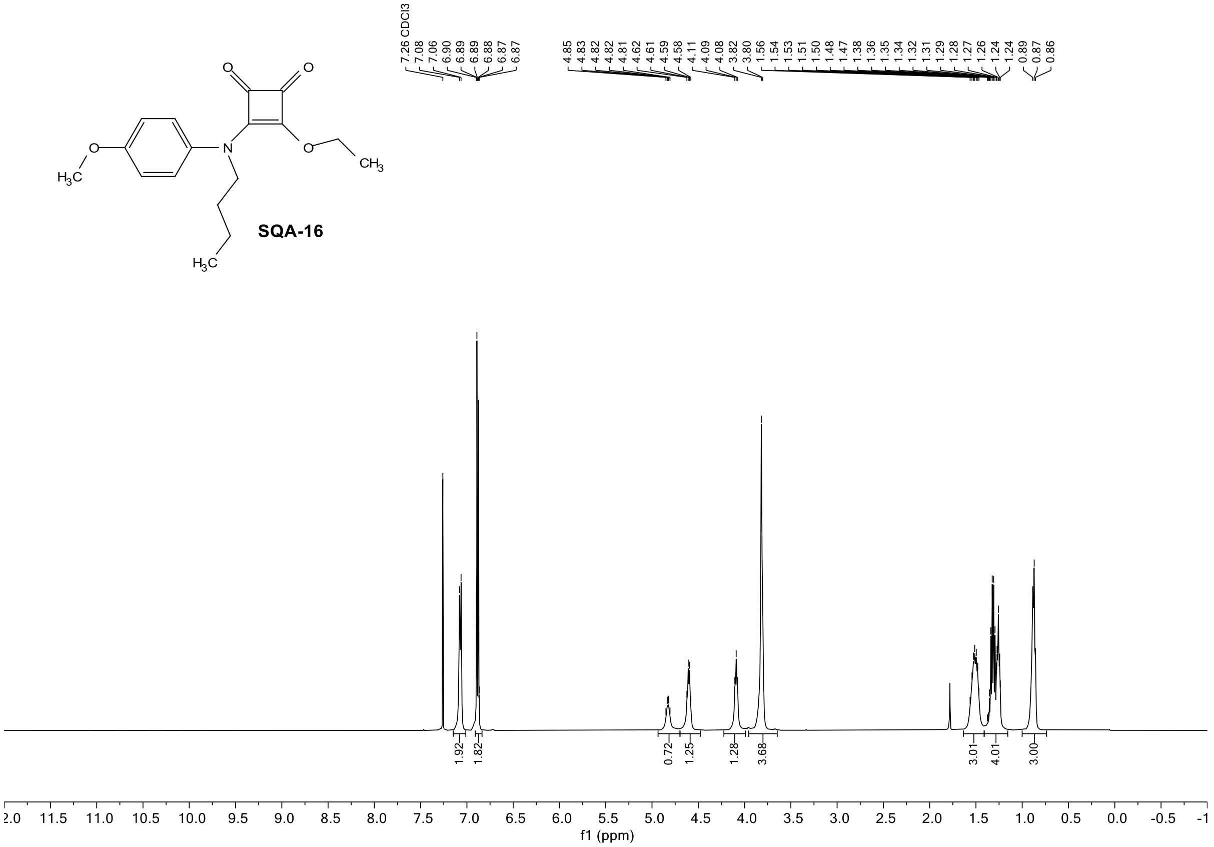
SQA-16
7.26 CDCl3
7.08
7.06
6.90
6.89
6.89
6.88
6.87
6.87
4.85
4.83
4.82
4.82
4.81
4.62
4.61
4.59
4.58
4.11
4.09
4.08
3.82
3.80
1.56
1.54
1.53
1.51
1.50
1.48
1.47
1.38
1.36
1.35
1.34
1.32
1.31
1.29
1.28
1.27
1.26
1.24
1.24
0.89
0.87
0.86
1.92
1.82
0.72
1.25
1.28
3.68
3.01
4.01
3.00
f1 (ppm)

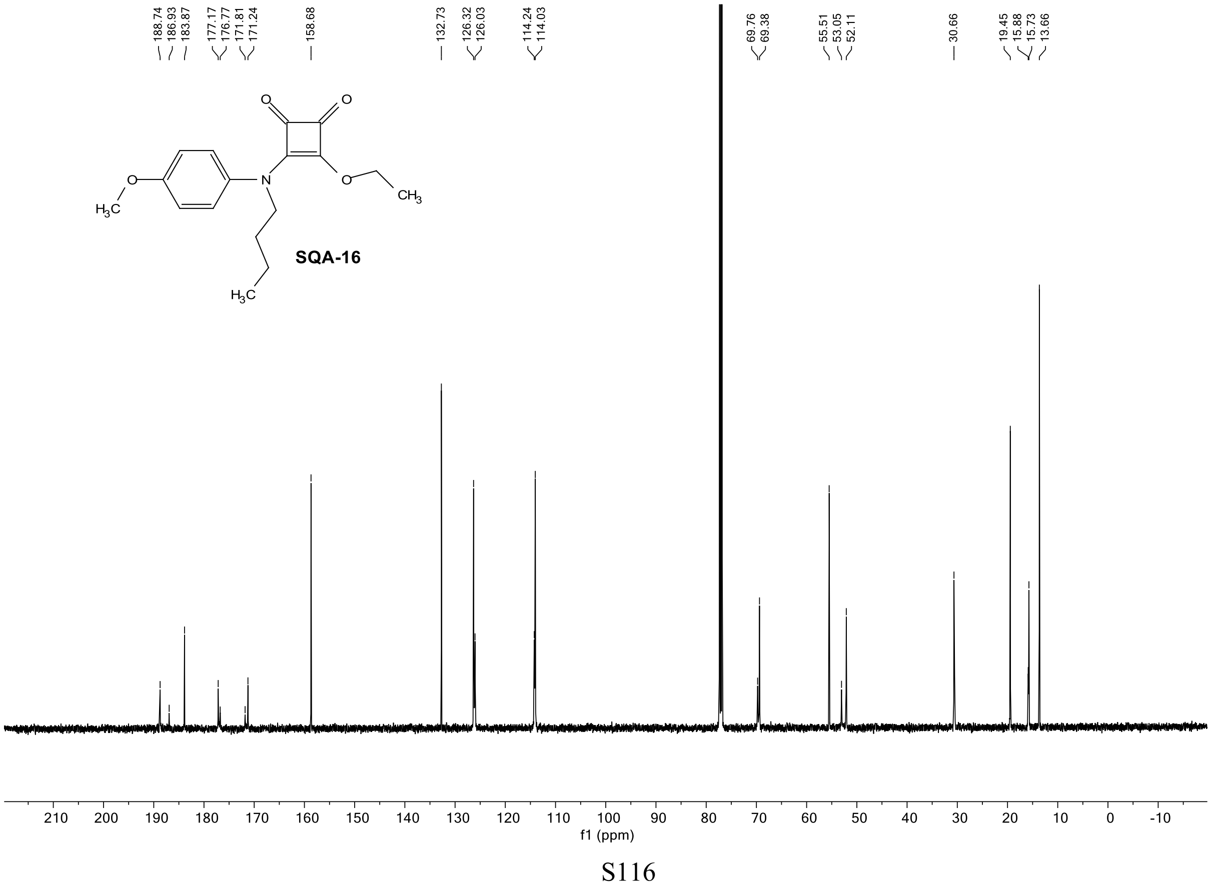
188.74
186.93
183.87
177.17
176.77
171.81
171.24
158.68
132.73
126.32
126.03
114.24
114.03
69.76
69.38
55.51
53.05
52.11
30.66
19.45
15.88
15.73
13.66
SQA-16
f1 (ppm)

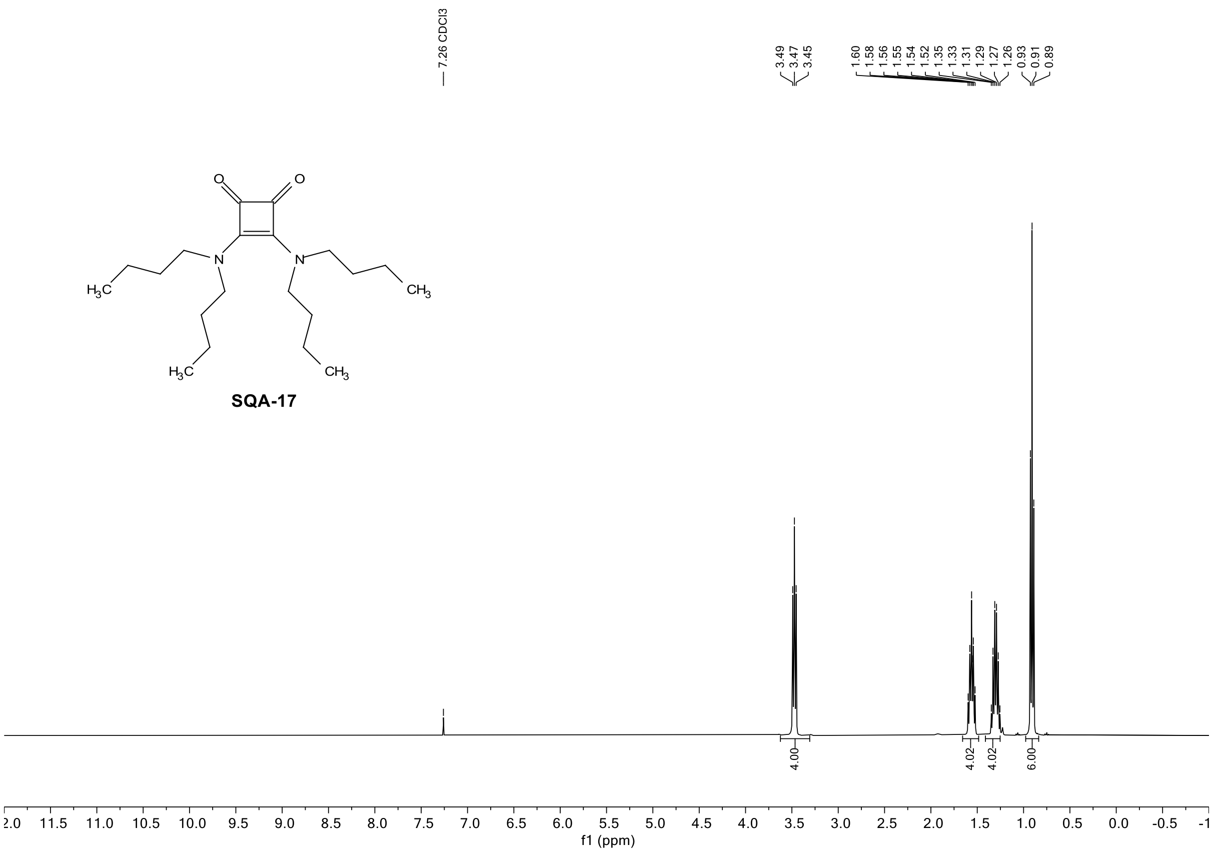
7.26 CDCl3
3.49
3.47
3.45
1.60
1.58
1.56
1.55
1.54
1.52
1.35
1.33
1.31
1.29
1.27
1.26
0.93
0.91
0.89
O
O
N
N
H$_3$C
CH$_3$
H$_3$C
CH$_3$
SQA-17
4.00
4.02
4.02
6.00
f1 (ppm)

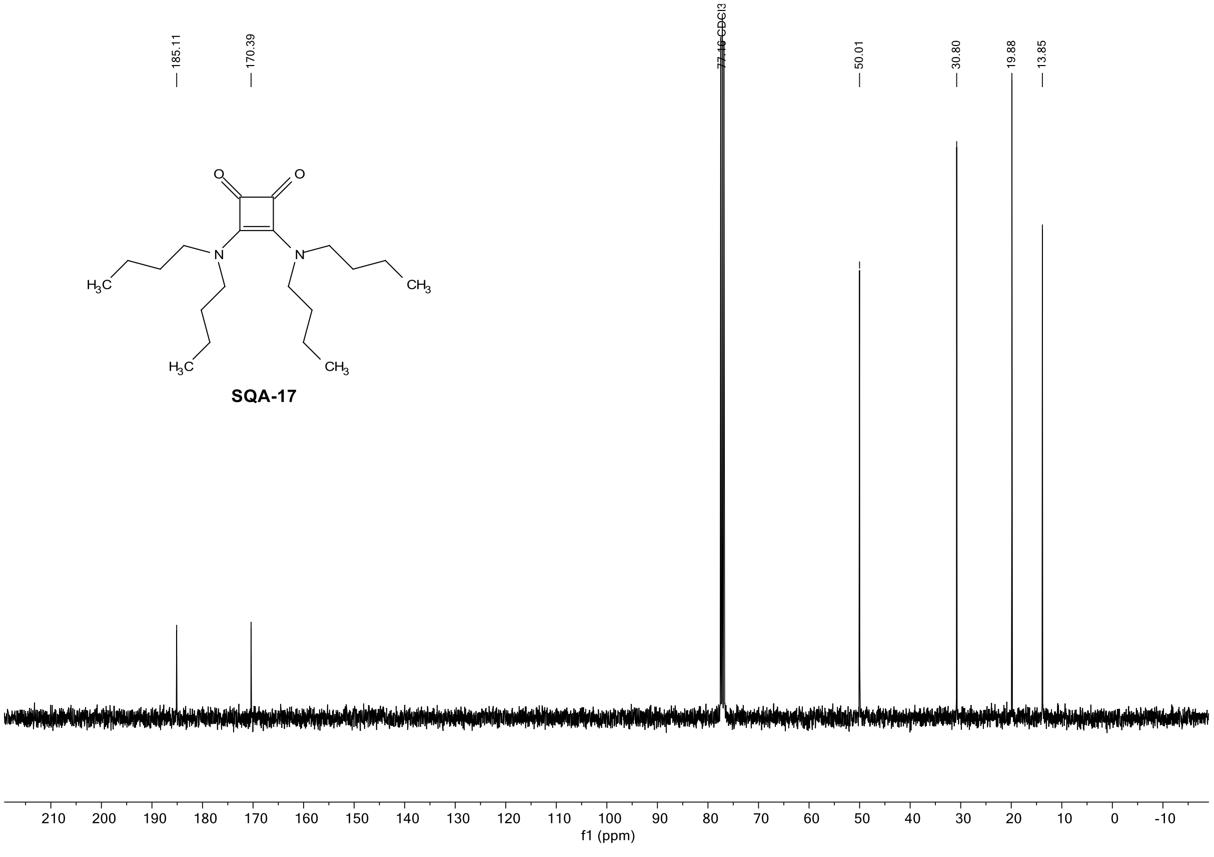
185.11
170.39
77.16 CDCl3
50.01
30.80
19.88
13.85
O
O
N
N
H$_3$C
CH$_3$
H$_3$C
CH$_3$
SQA-17
f1 (ppm)

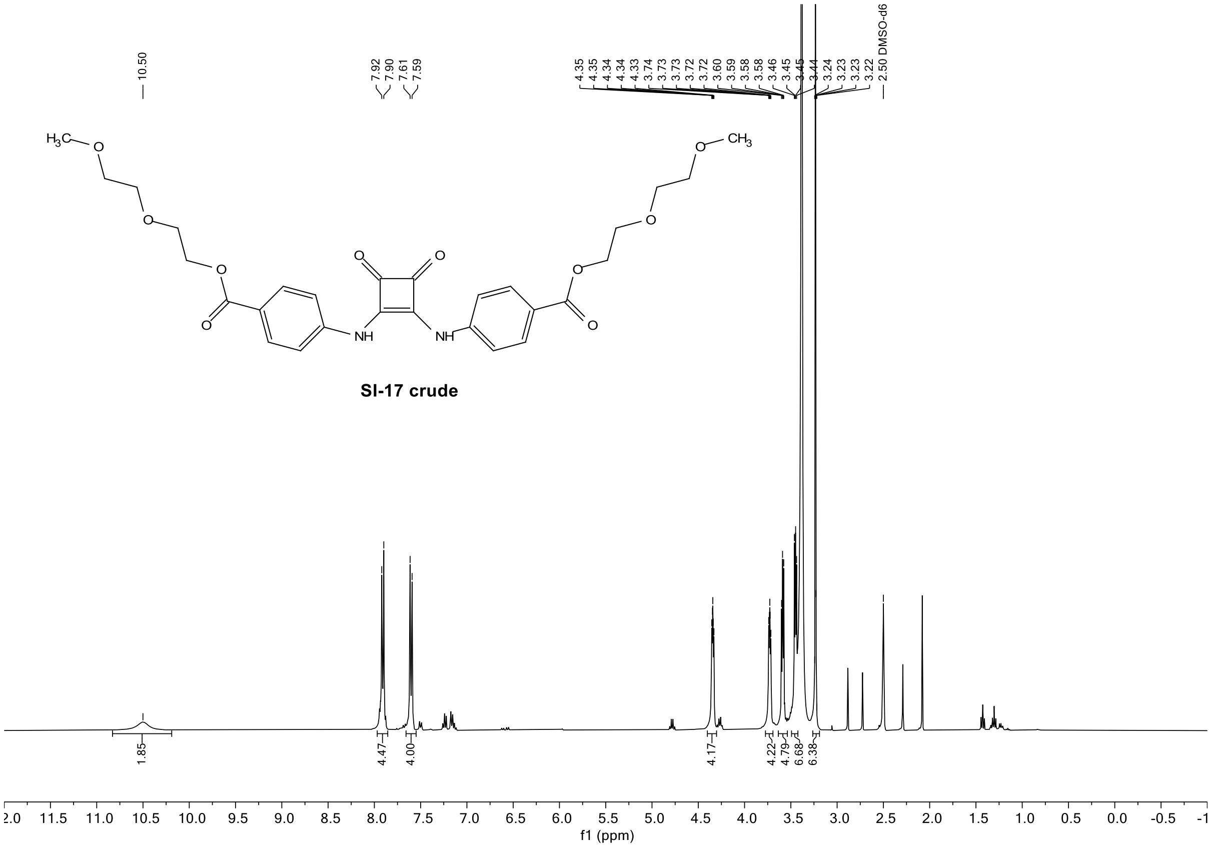
10.50
7.92
7.90
7.61
7.59
4.35
4.35
4.34
4.34
4.33
3.74
3.73
3.73
3.72
3.72
3.60
3.59
3.58
3.58
3.46
3.45
3.45
3.44
3.24
3.23
3.23
3.22
2.50 DMSO-d6
SI-17 crude
1.85
4.47
4.00
4.17
4.22
4.79
6.68
6.38
f1 (ppm)

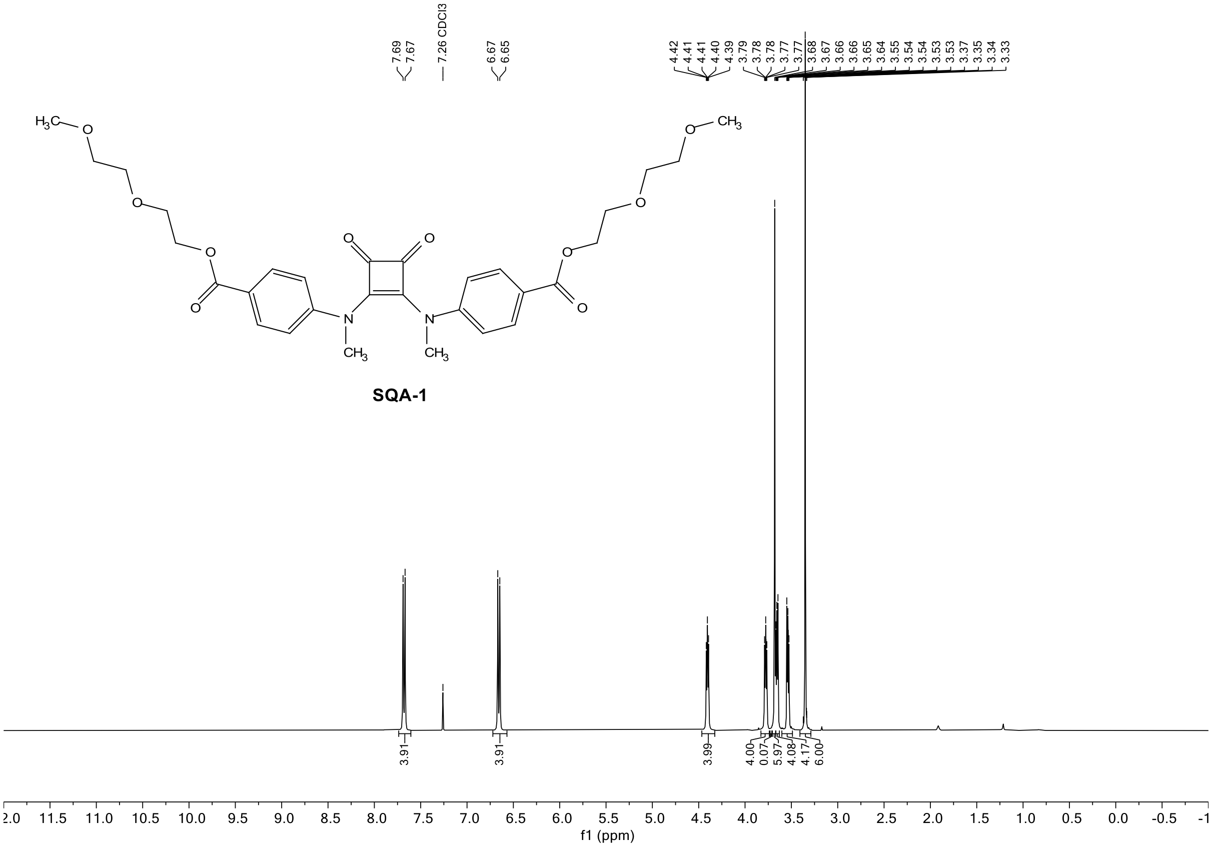
7.69
7.67
7.26 CDCl3
6.67
6.65
4.42
4.41
4.41
4.40
4.39
3.79
3.78
3.78
3.77
3.77
3.68
3.67
3.66
3.66
3.65
3.64
3.55
3.54
3.54
3.53
3.53
3.37
3.35
3.34
3.33
SQA-1
3.91
3.91
3.99
4.00
0.07
5.97
4.08
4.17
6.00
f1 (ppm)

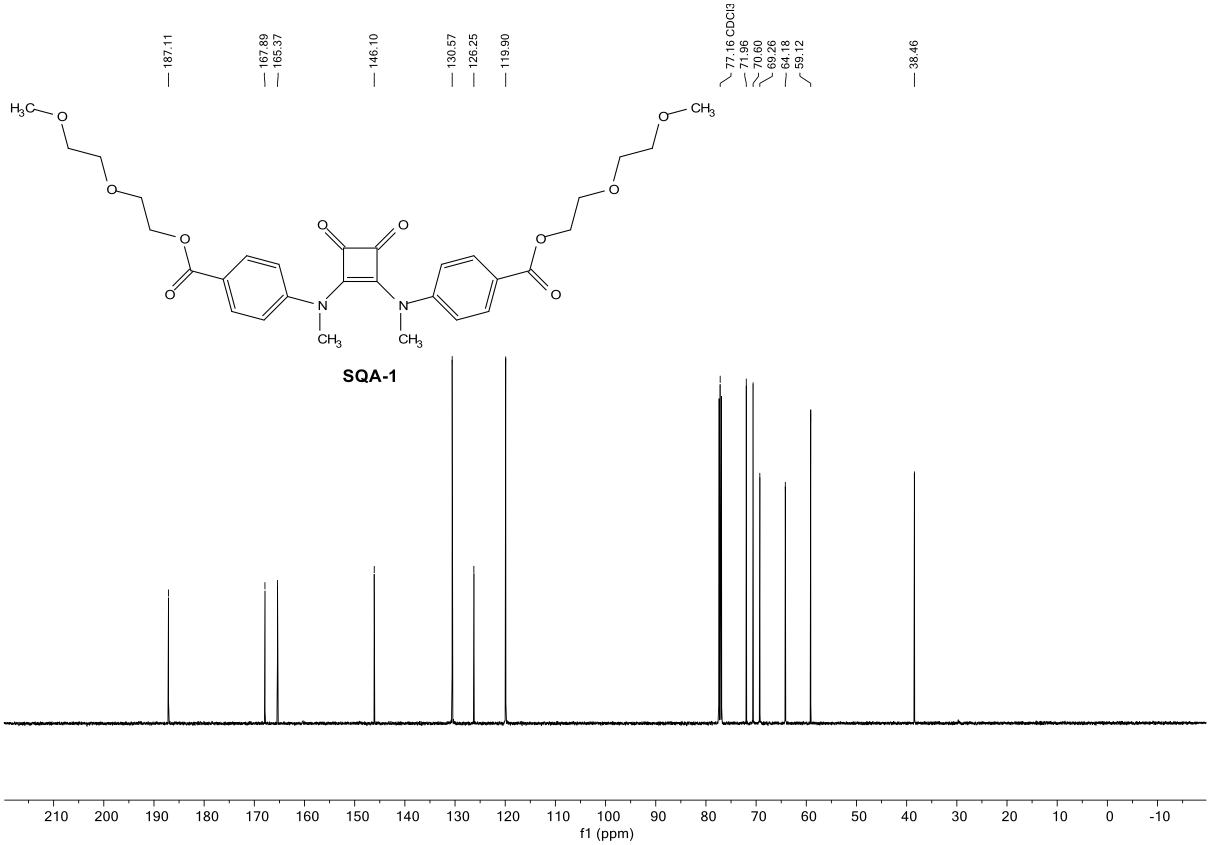
187.11
167.89
165.37
146.10
130.57
126.25
119.90
77.16 CDCl3
71.96
70.60
69.26
64.18
59.12
38.46
SQA-1
f1 (ppm)

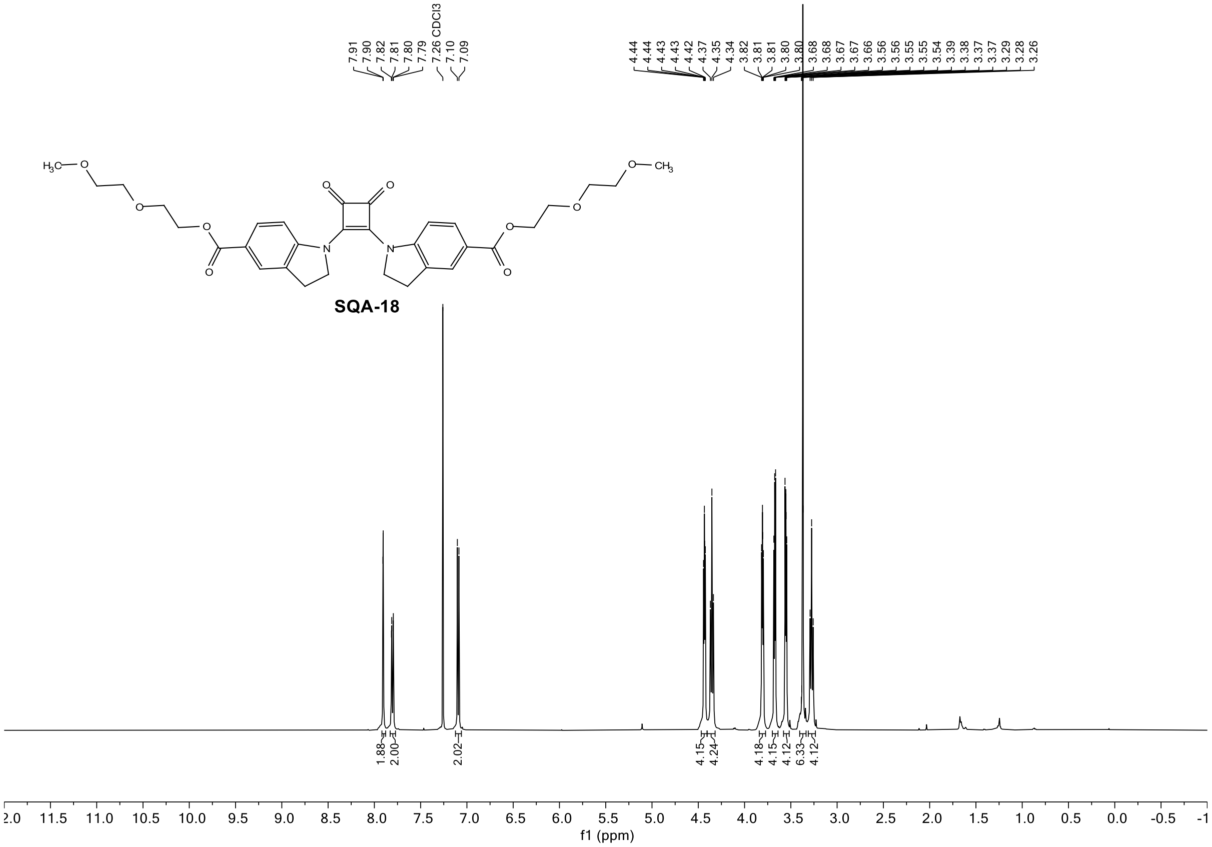
7.91
7.90
7.82
7.81
7.80
7.79
7.26 CDCl3
7.10
7.09
4.44
4.44
4.43
4.43
4.42
4.37
4.35
4.34
3.82
3.81
3.81
3.80
3.80
3.68
3.68
3.67
3.67
3.66
3.56
3.56
3.55
3.55
3.54
3.39
3.38
3.37
3.37
3.29
3.28
3.26
SQA-18
1.88
2.00
2.02
4.15
4.24
4.18
4.15
4.12
6.33
4.12
f1 (ppm)

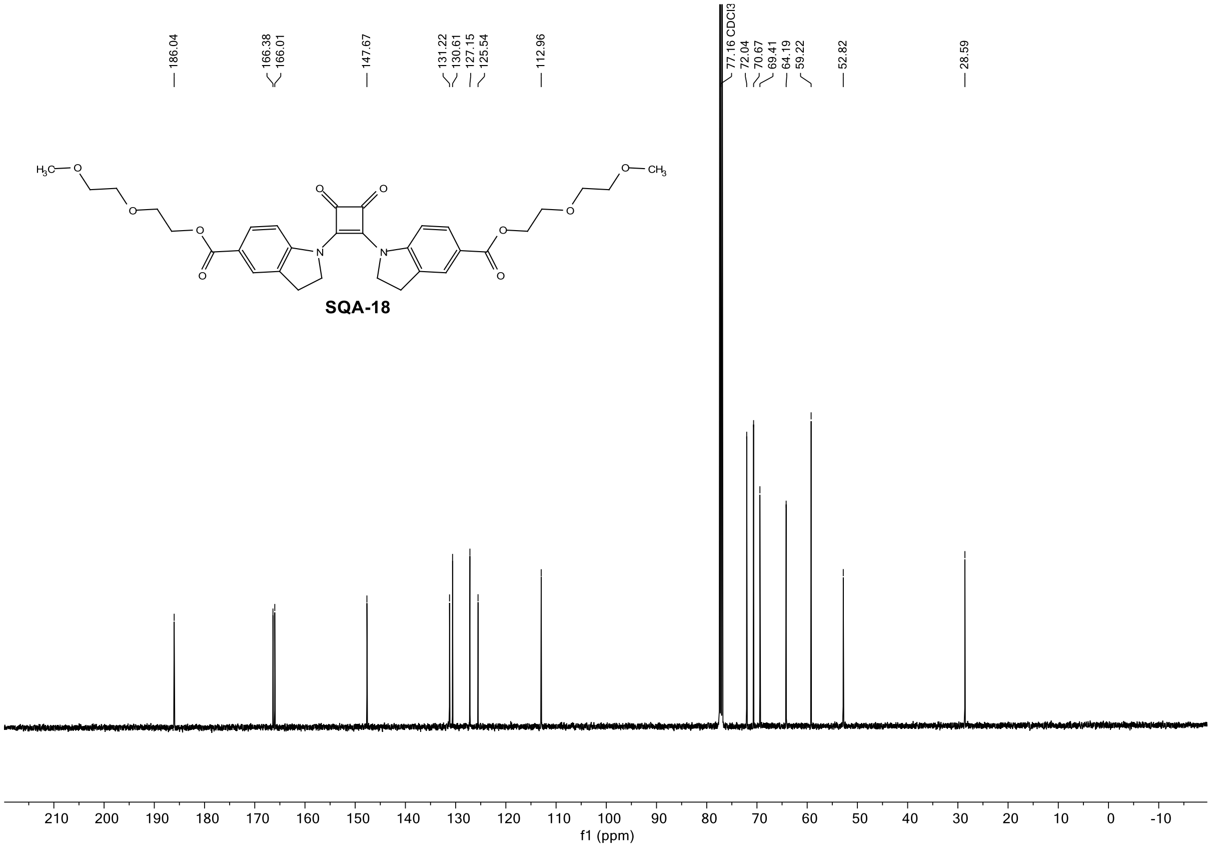
186.04
166.38
166.01
147.67
131.22
130.61
127.15
125.54
112.96
77.16 CDCl3
72.04
70.67
69.41
64.19
59.22
52.82
28.59
SQA-18
f1 (ppm)

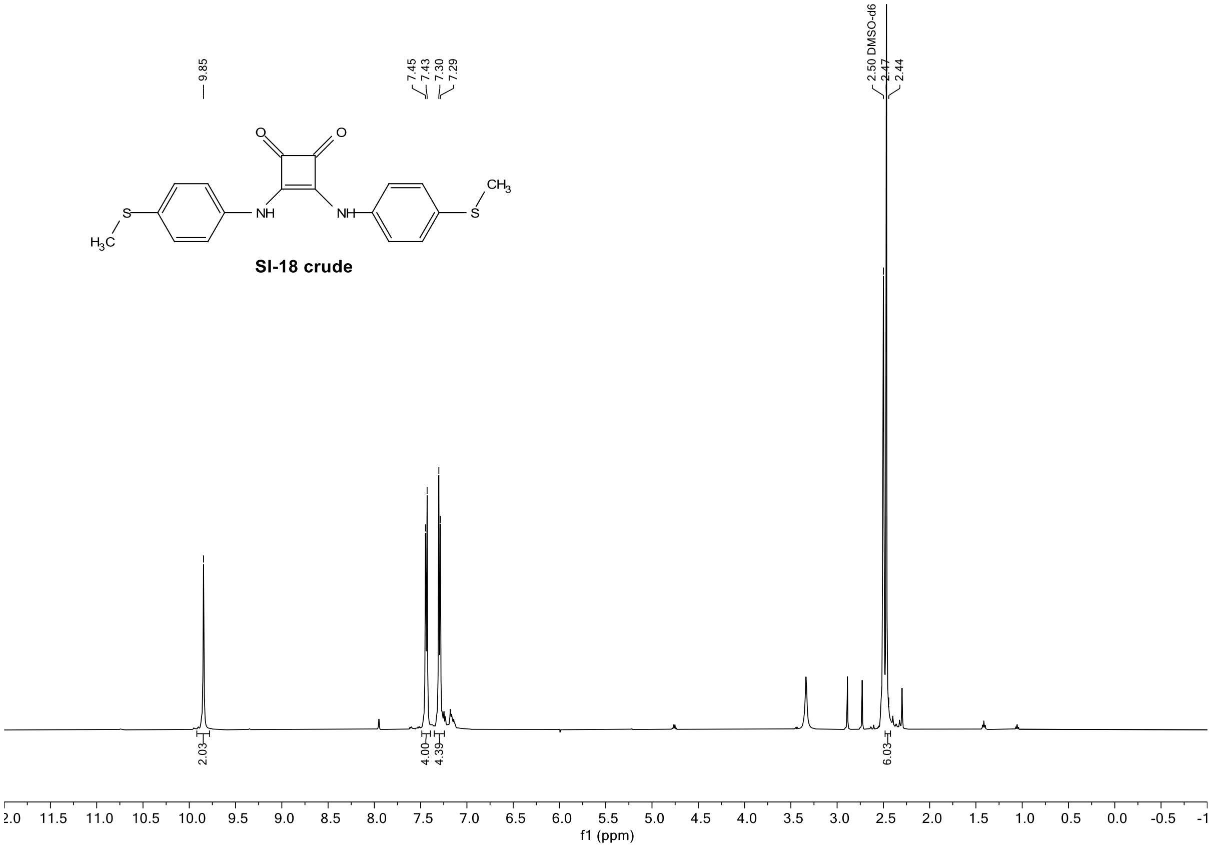
9.85
7.45
7.43
7.30
7.29
2.50 DMSO-d6
2.47
2.44
SI-18 crude
2.03
4.00
4.39
6.03
f1 (ppm)

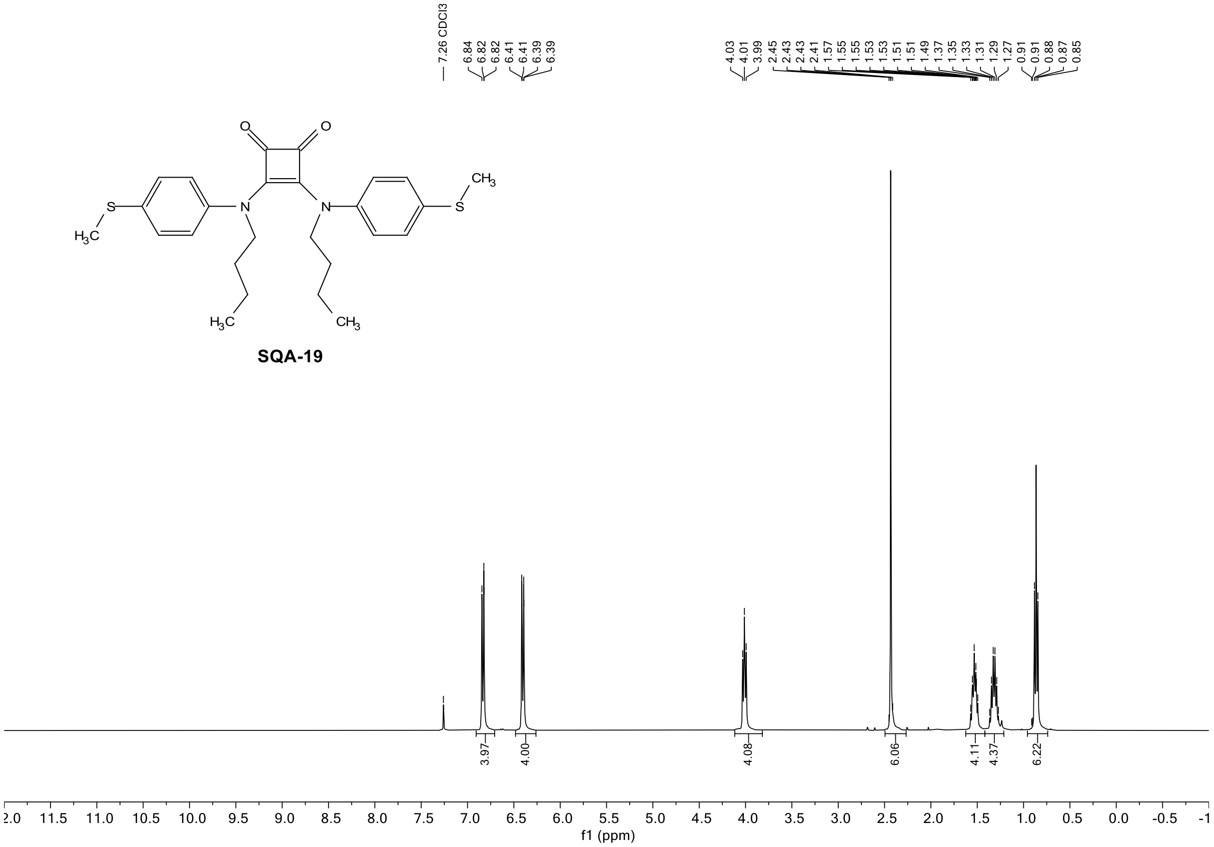
7.26 CDCl3
6.84
6.82
6.82
6.41
6.41
6.39
6.39
4.03
4.01
3.99
2.45
2.43
2.43
2.41
1.57
1.55
1.55
1.53
1.53
1.51
1.51
1.49
1.37
1.35
1.33
1.31
1.29
1.27
0.91
0.91
0.88
0.87
0.85
SQA-19
3.97
4.00
4.08
6.06
4.11
4.37
6.22
f1 (ppm)

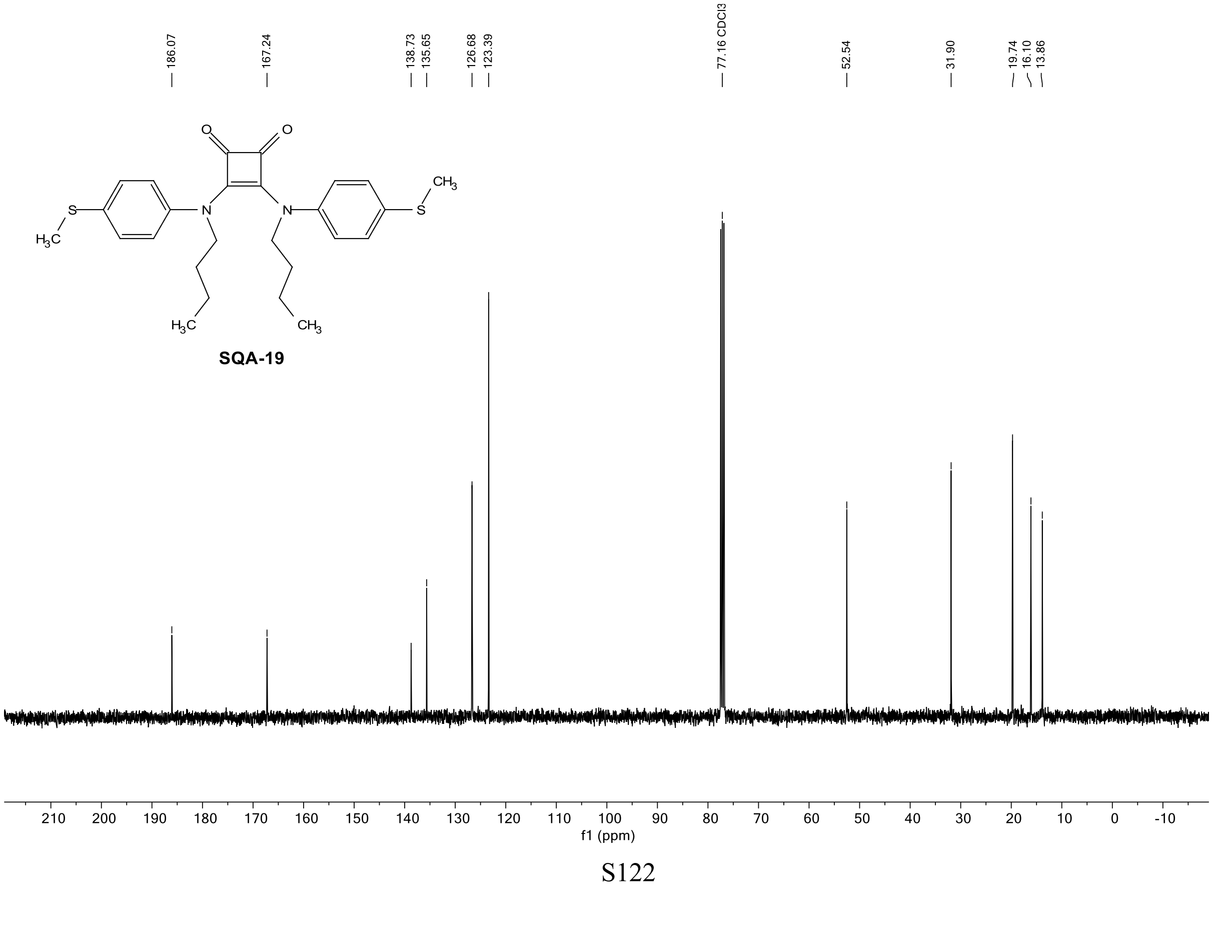
186.07
167.24
138.73
135.65
126.68
123.39
77.16 CDCl3
52.54
31.90
19.74
16.10
13.86
SQA-19
f1 (ppm)

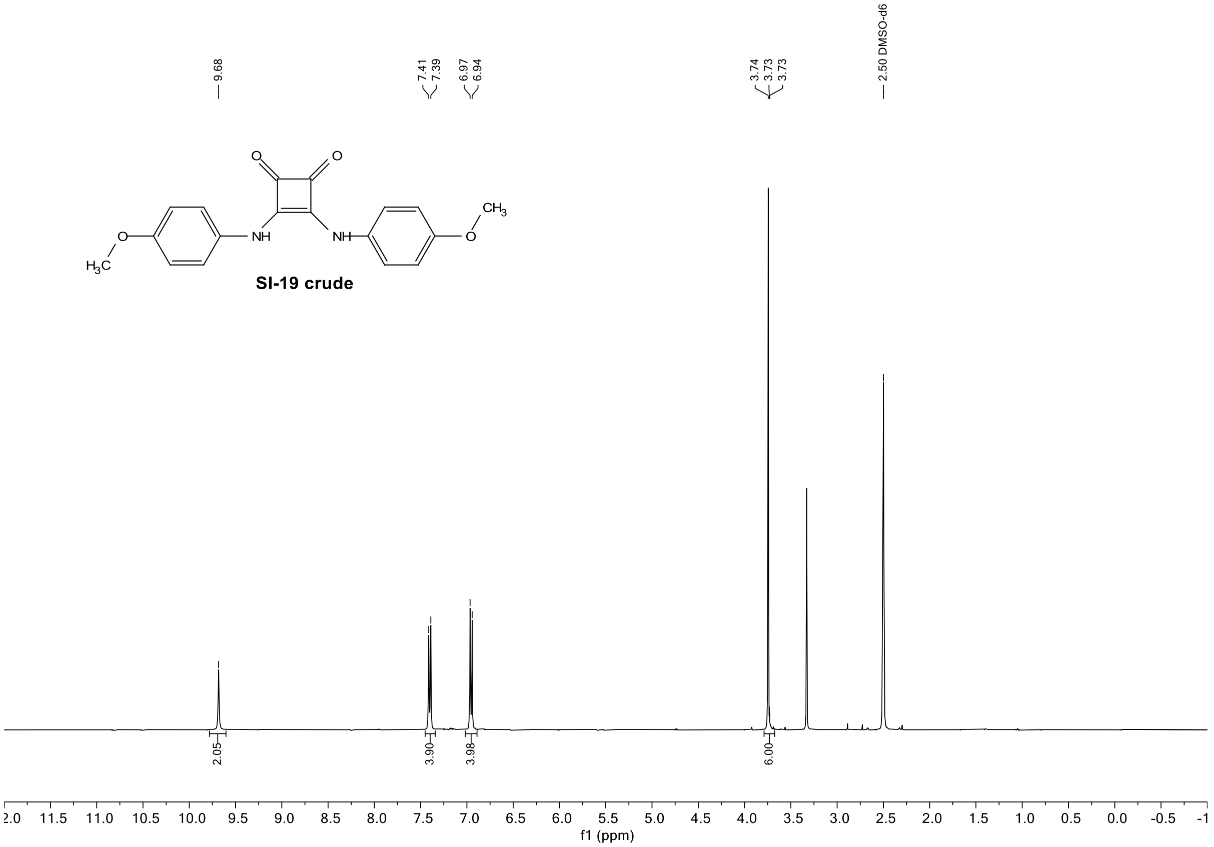
9.68
7.41
7.39
6.97
6.94
3.74
3.73
3.73
2.50 DMSO-d6
2.05
3.90
3.98
6.00
f1 (ppm)

SI-19 crude

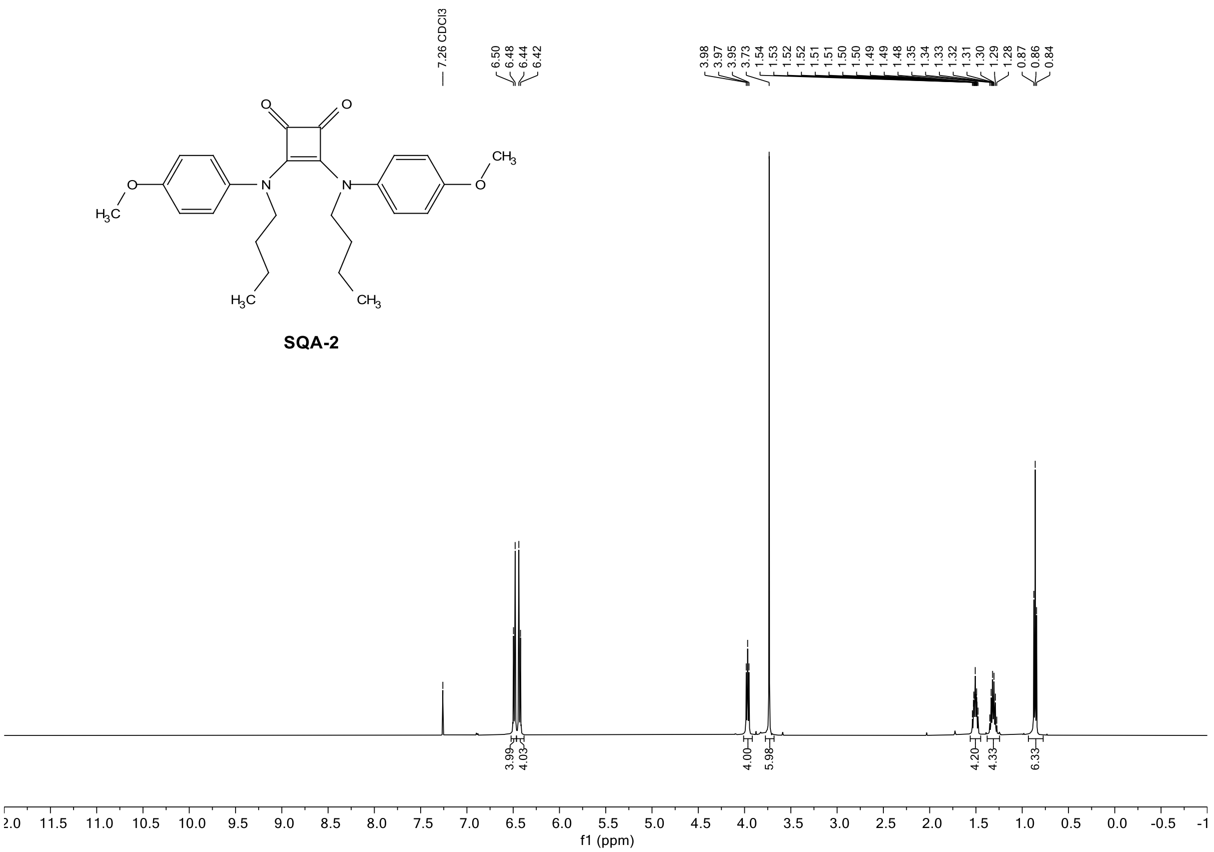
SQA-2
7.26 CDCl3
6.50
6.48
6.44
6.42
3.98
3.97
3.95
3.73
1.54
1.53
1.52
1.52
1.51
1.50
1.50
1.49
1.49
1.48
1.35
1.34
1.33
1.32
1.31
1.30
1.29
1.28
0.87
0.86
0.84
3.99
4.03
4.00
5.98
4.20
4.33
6.33
f1 (ppm)

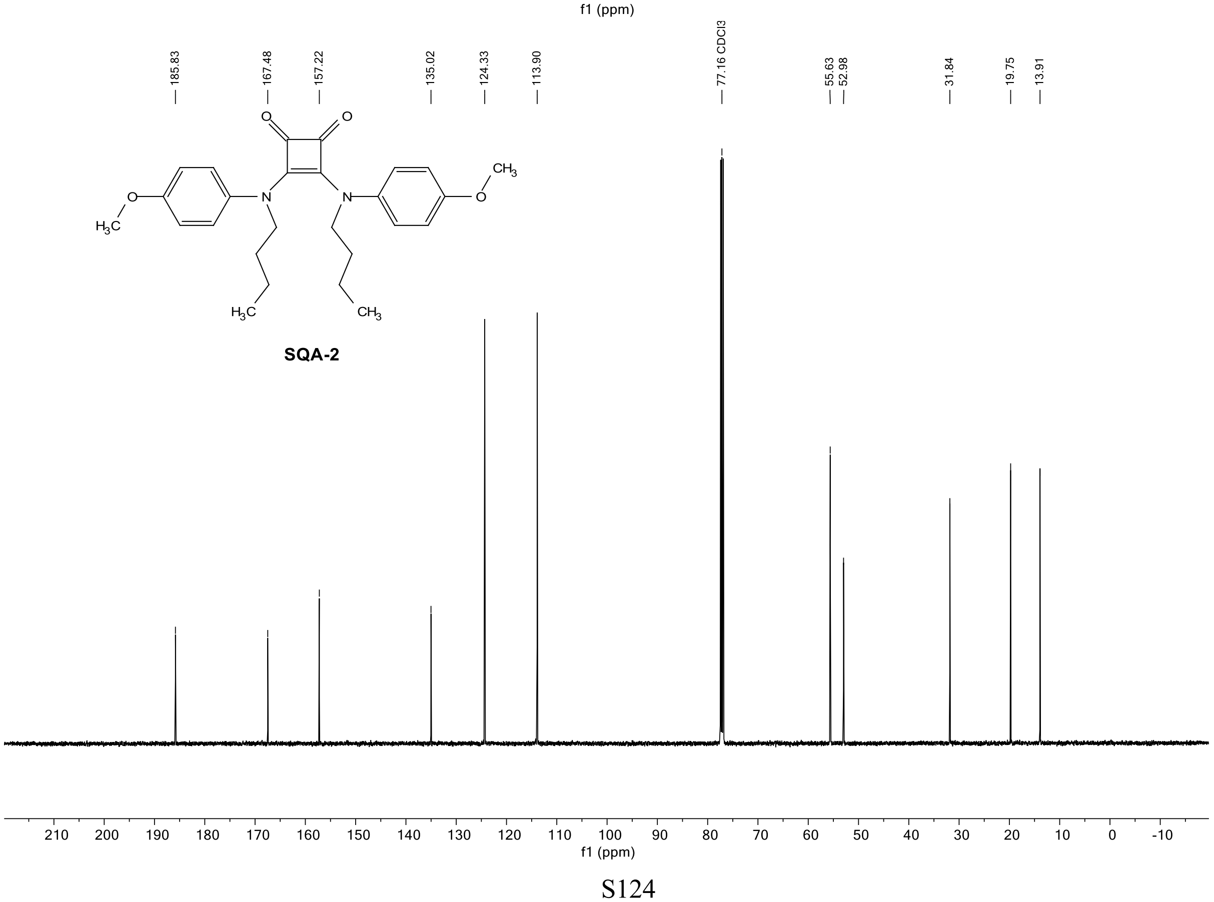
SQA-2
185.83
167.48
157.22
135.02
124.33
113.90
77.16 CDCl3
55.63
52.98
31.84
19.75
13.91
f1 (ppm)

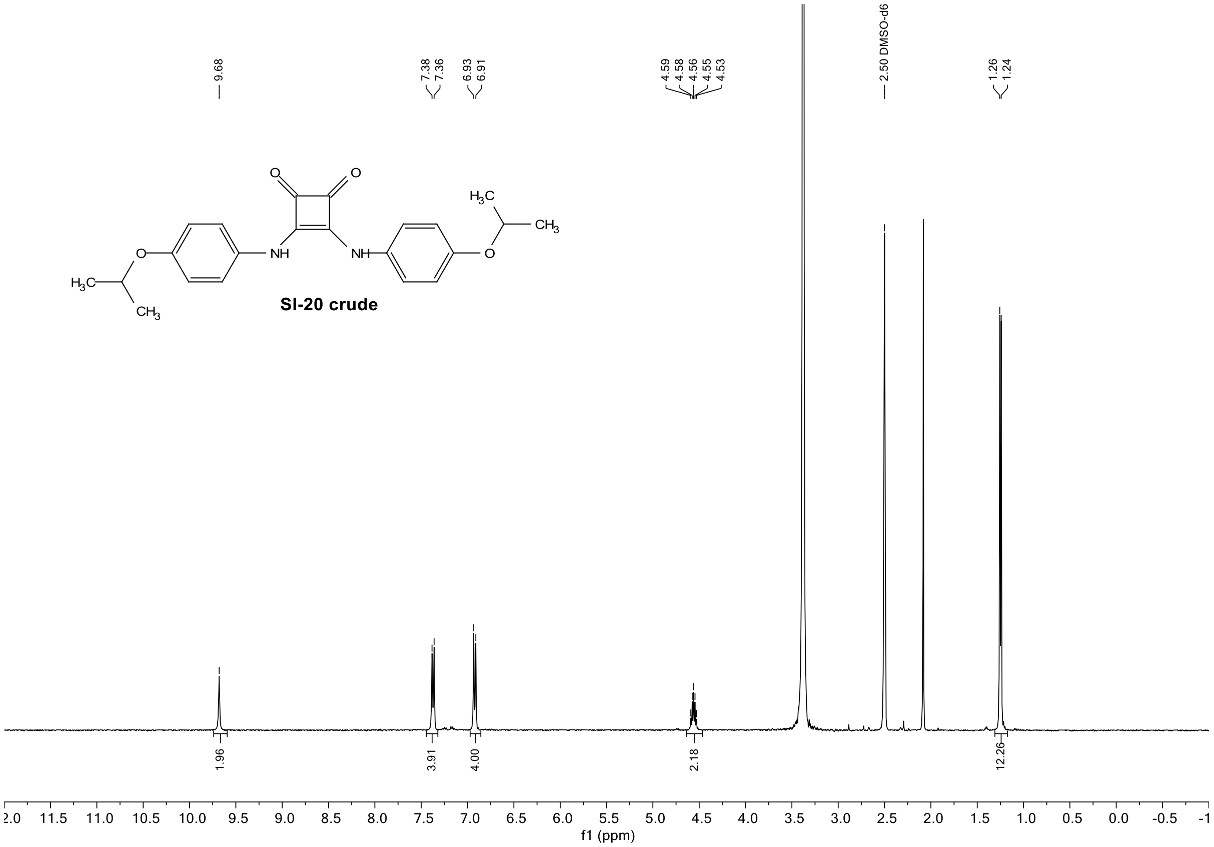
9.68
7.38
7.36
6.93
6.91
4.59
4.58
4.56
4.55
4.53
2.50 DMSO-d6
1.26
1.24
H3C
CH3
O
NH
SI-20 crude
1.96
3.91
4.00
2.18
12.26
f1 (ppm)

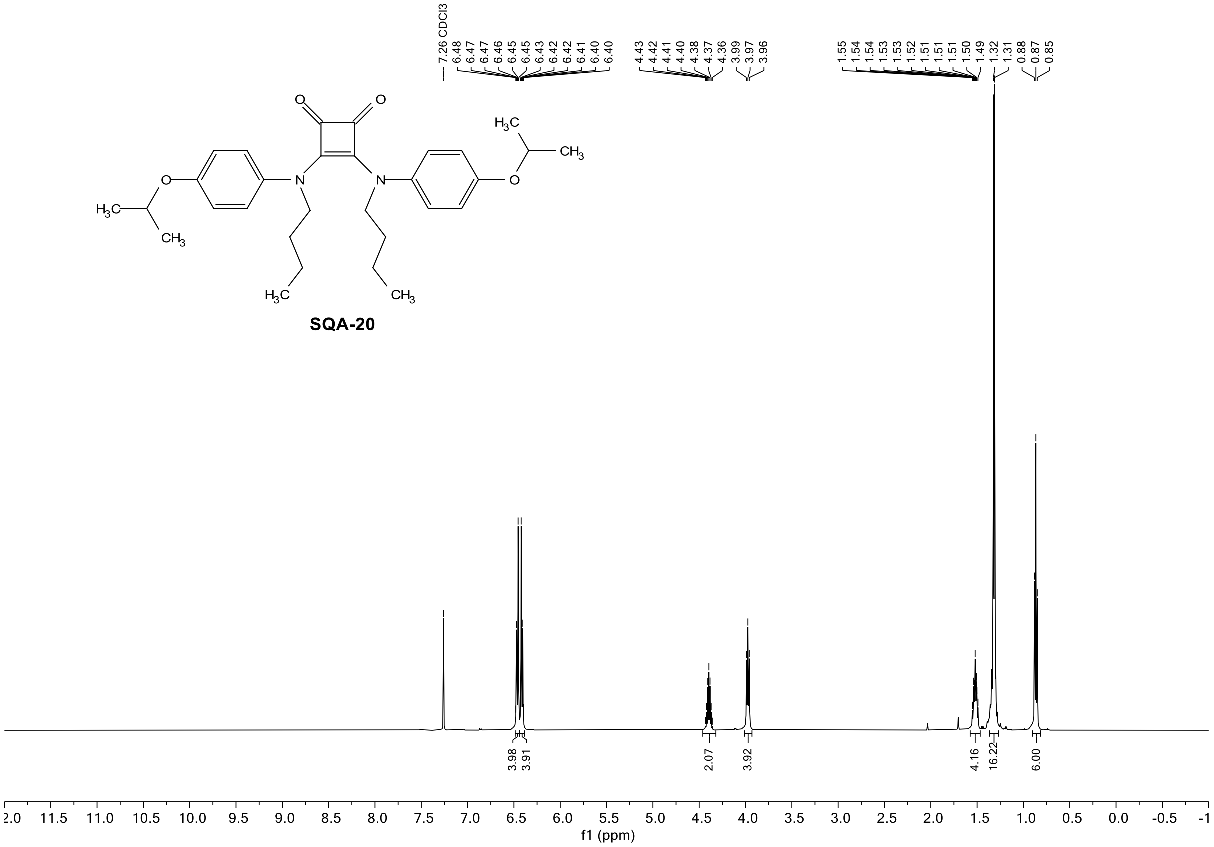
7.26 CDCl3
6.48
6.47
6.47
6.46
6.45
6.45
6.43
6.42
6.42
6.41
6.40
6.40
4.43
4.42
4.41
4.40
4.38
4.37
4.36
3.99
3.97
3.96
1.55
1.54
1.54
1.53
1.53
1.52
1.51
1.51
1.51
1.50
1.49
1.32
1.31
0.88
0.87
0.85
SQA-20
3.98
3.91
2.07
3.92
4.16
16.22
6.00
f1 (ppm)

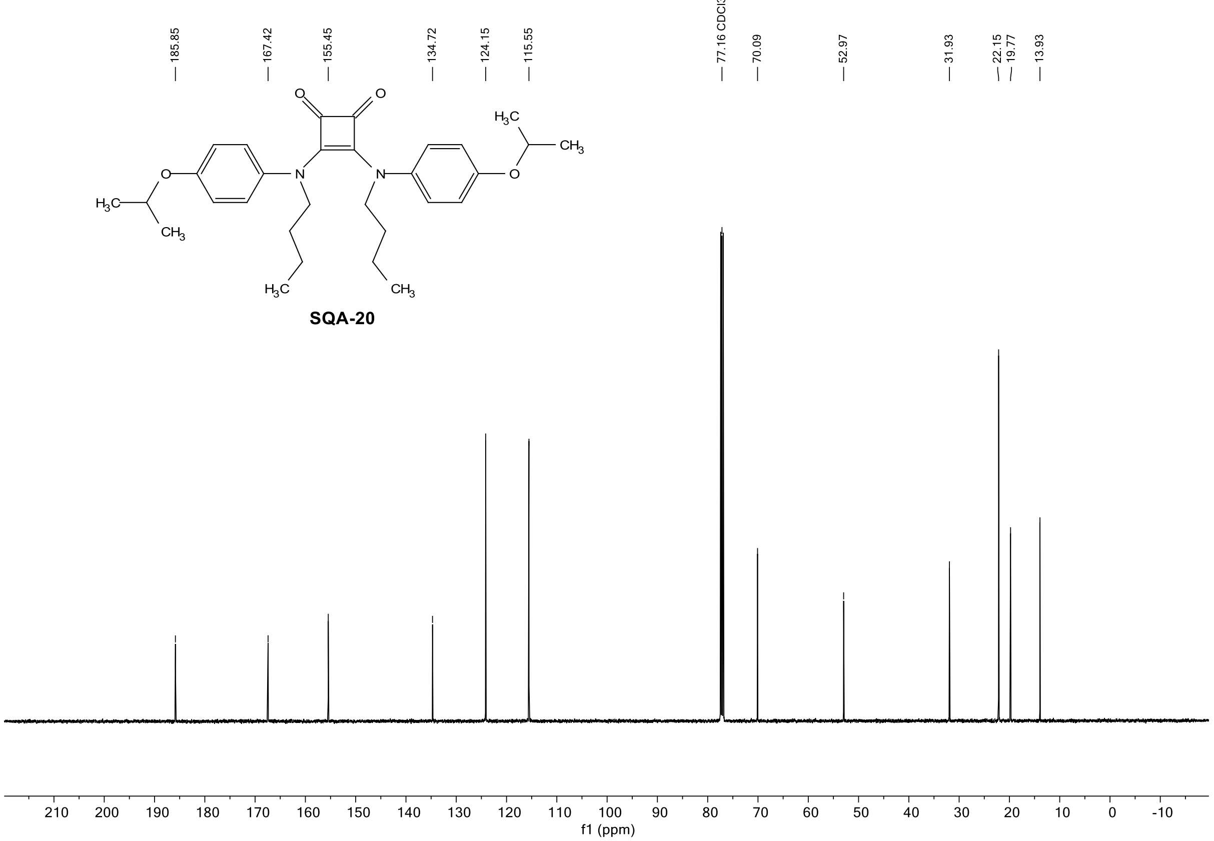
185.85
167.42
155.45
134.72
124.15
115.55
77.16 CDCl3
70.09
52.97
31.93
22.15
19.77
13.93
SQA-20
f1 (ppm)

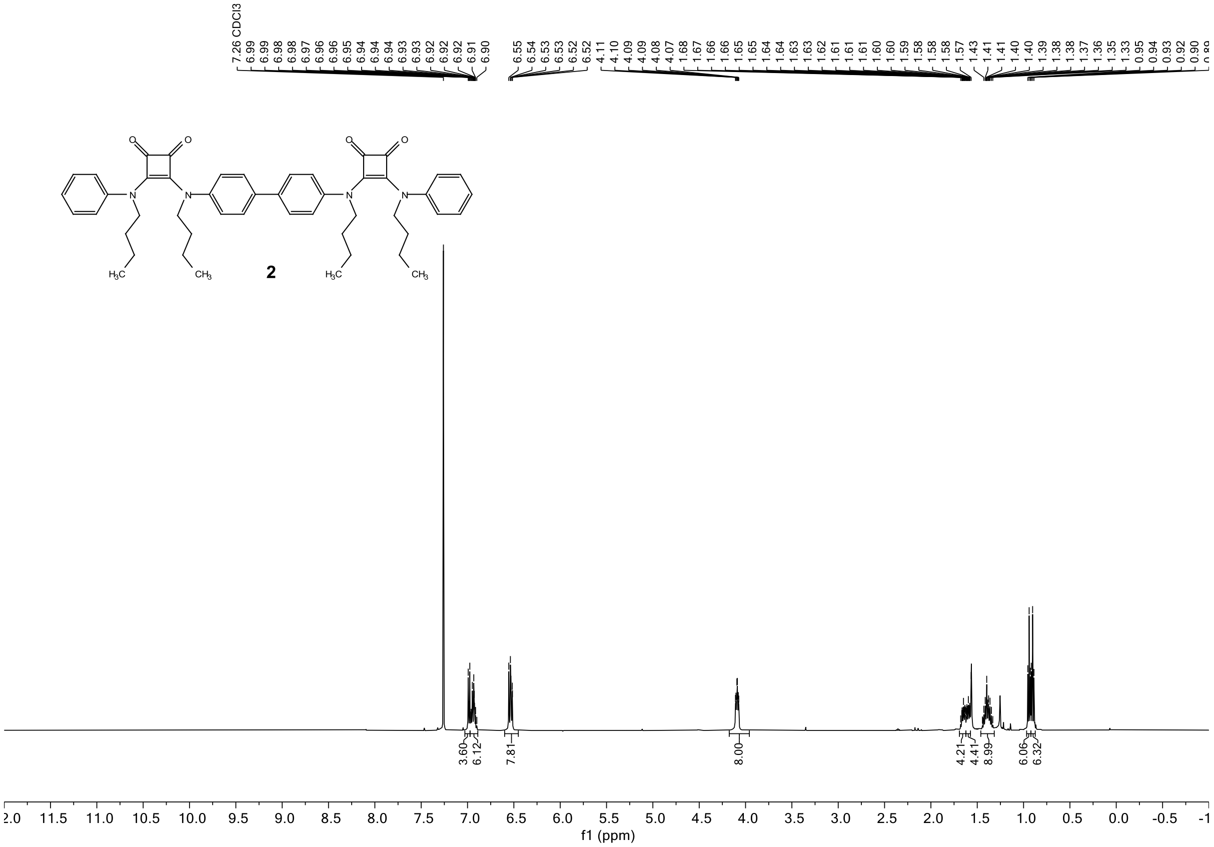
7.26 CDCl3
2
f1 (ppm)

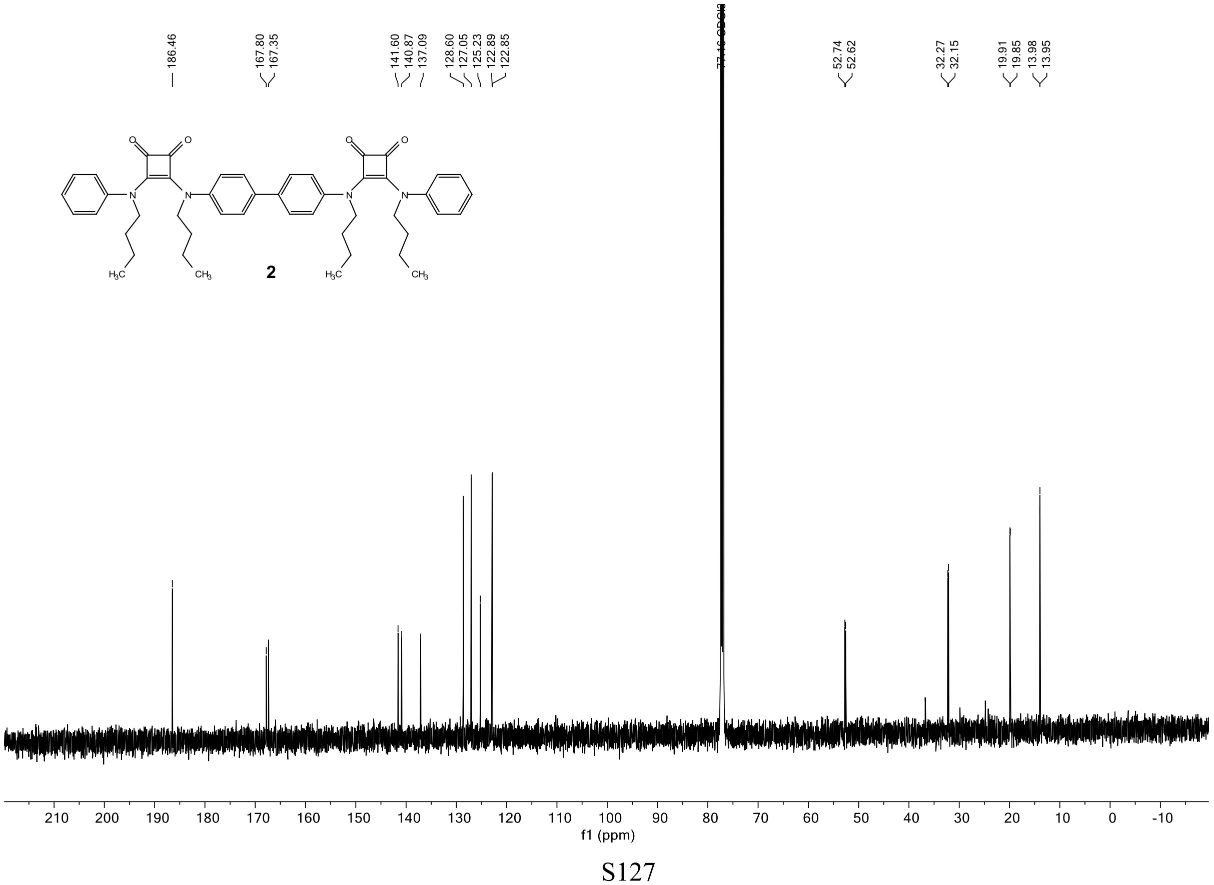
186.46
167.80
167.35
141.60
140.87
137.09
128.60
127.05
125.23
122.89
122.85
52.74
52.62
32.27
32.15
19.91
19.85
13.98
13.95
2
f1 (ppm)

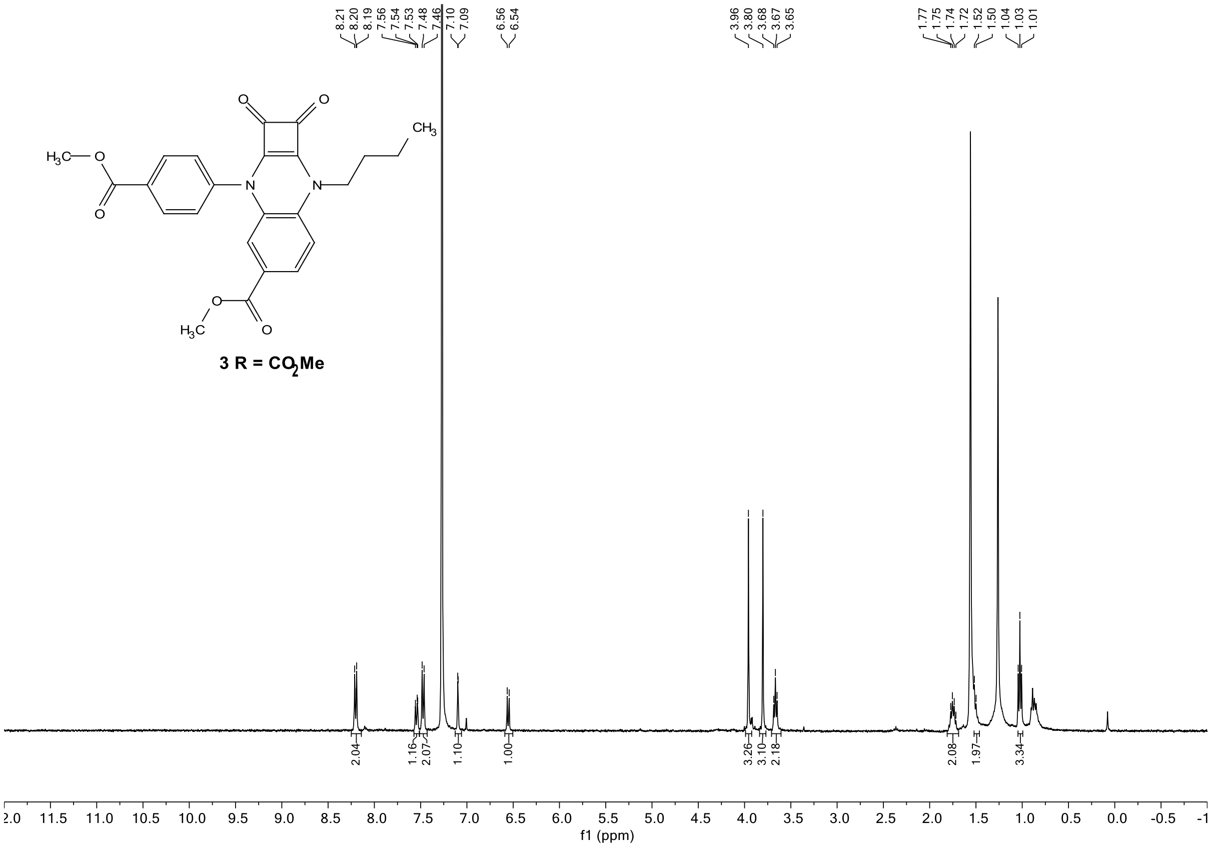
3 R = CO2Me
f1 (ppm)

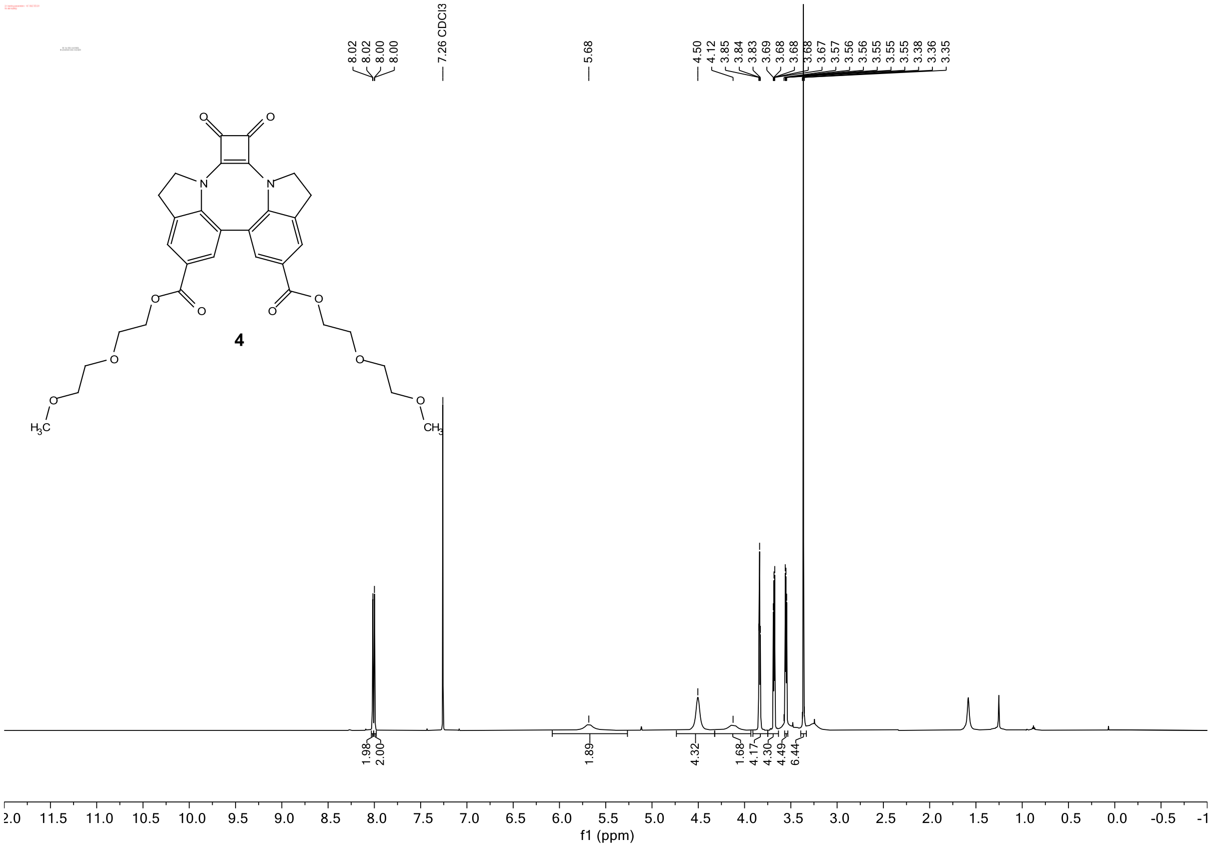

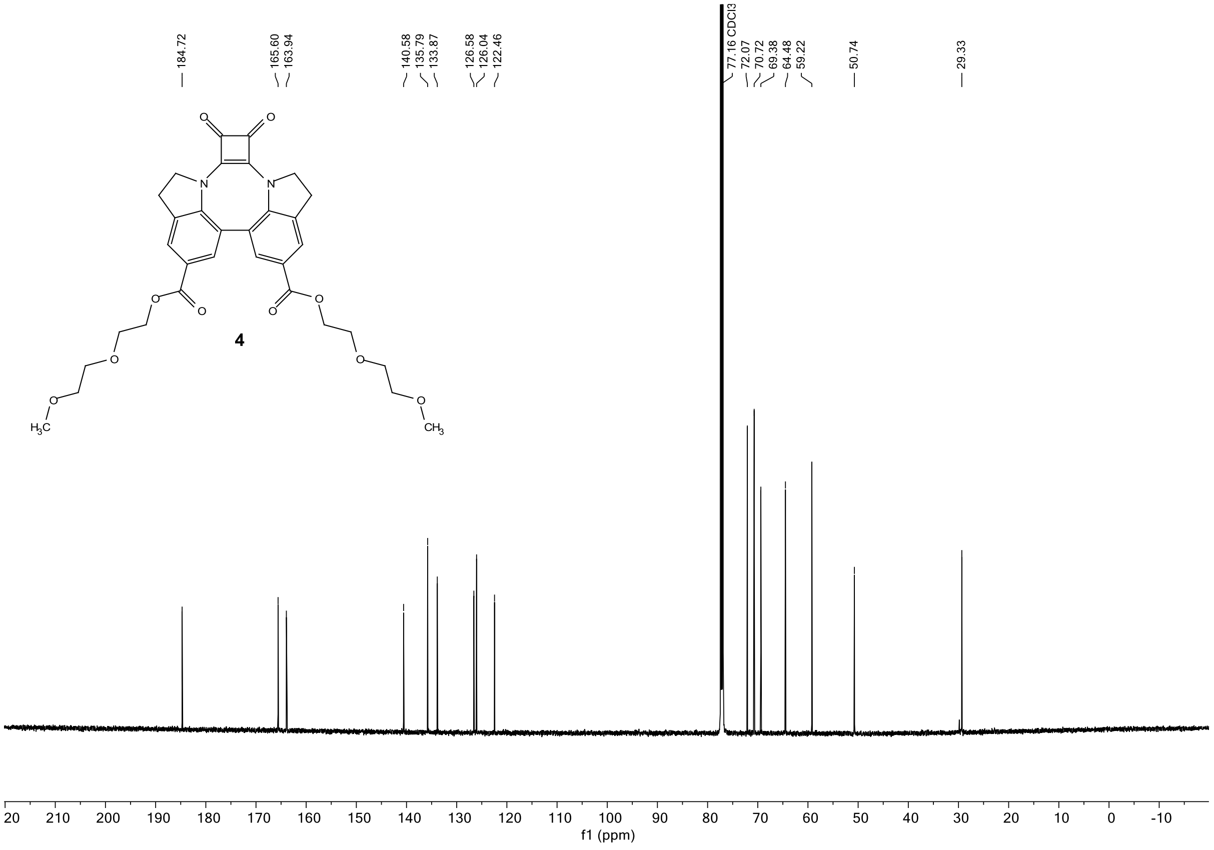

Supplement: Supplementary file 1 — ja3c14776_si_001.pdf [file ja3c14776_si_001.pdf]
